# Supplementary material for: Chromosomal instability promotes cell migration and invasion via EFEMP1 secretion into extracellular vesicles
Source: EMBO J. 2026 Apr 13;45(10):3471–99. doi: 10.1038/s44318-026-00766-4 (PMC13187162; doi:10.1038/s44318-026-00766-4)
Supplement: Supplementary file 4 — Movie EV1 [file 44318_2026_766_MOESM4_ESM.zip › Movie EV1/Legend Movie EV1.docx]

**Movie EV1:** Time-lapse imaging of BT549 recipient cells confirms uptake of fluorescently labelled EVs isolated from CIN-induced BT549 donor cells expressing the EV-specific pHluorin_M153R reporter.
