# Supplementary figures and images for: Chromosomal instability promotes cell migration and invasion via EFEMP1 secretion into extracellular vesicles (part 1 of 2)
Source: EMBO J. 2026 Apr 13;45(10):3471–99. doi: 10.1038/s44318-026-00766-4 (PMC13187162; doi:10.1038/s44318-026-00766-4)

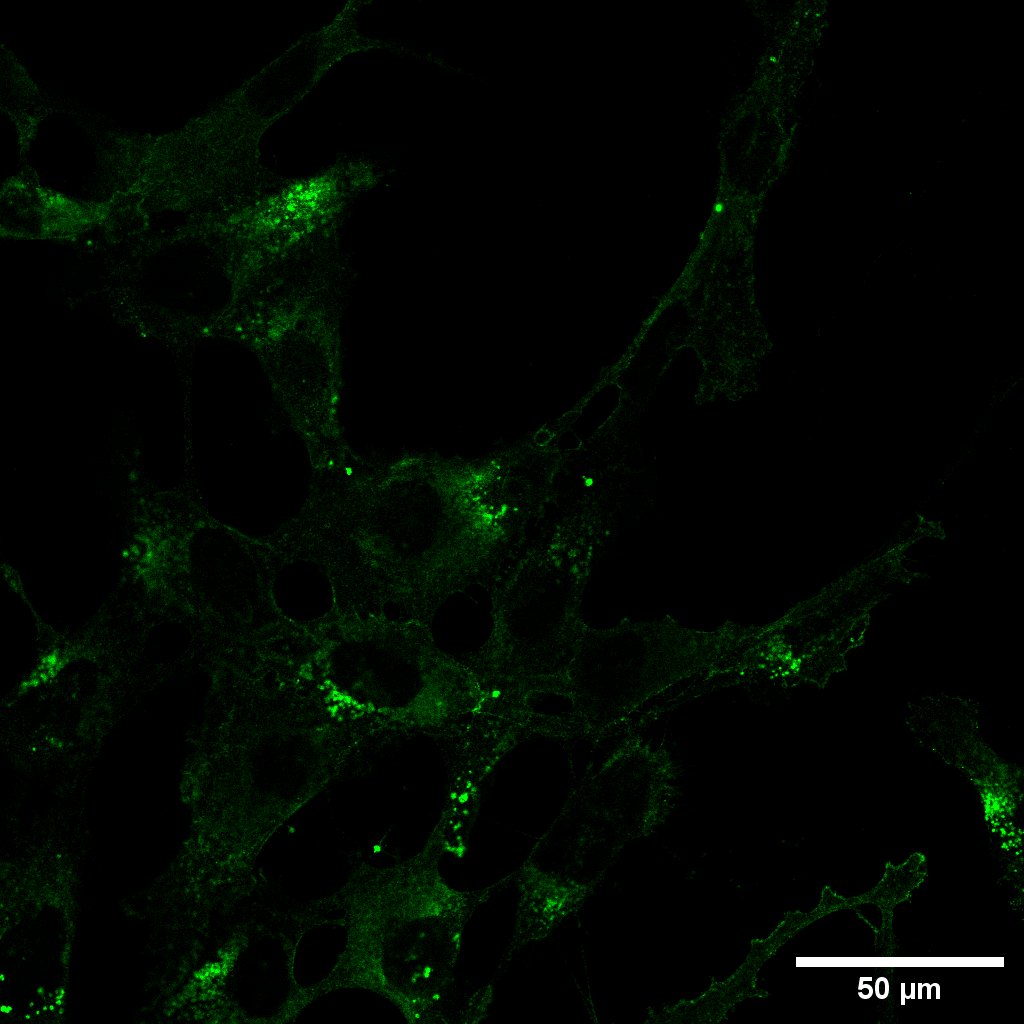

Supplement: Supplementary file 5 — Source data Fig. 1 [file 44318_2026_766_MOESM5_ESM.zip › Figure1/Fig1c/mda dmso/C2-mdamb 231 dmso.lif - Series006.jpg]

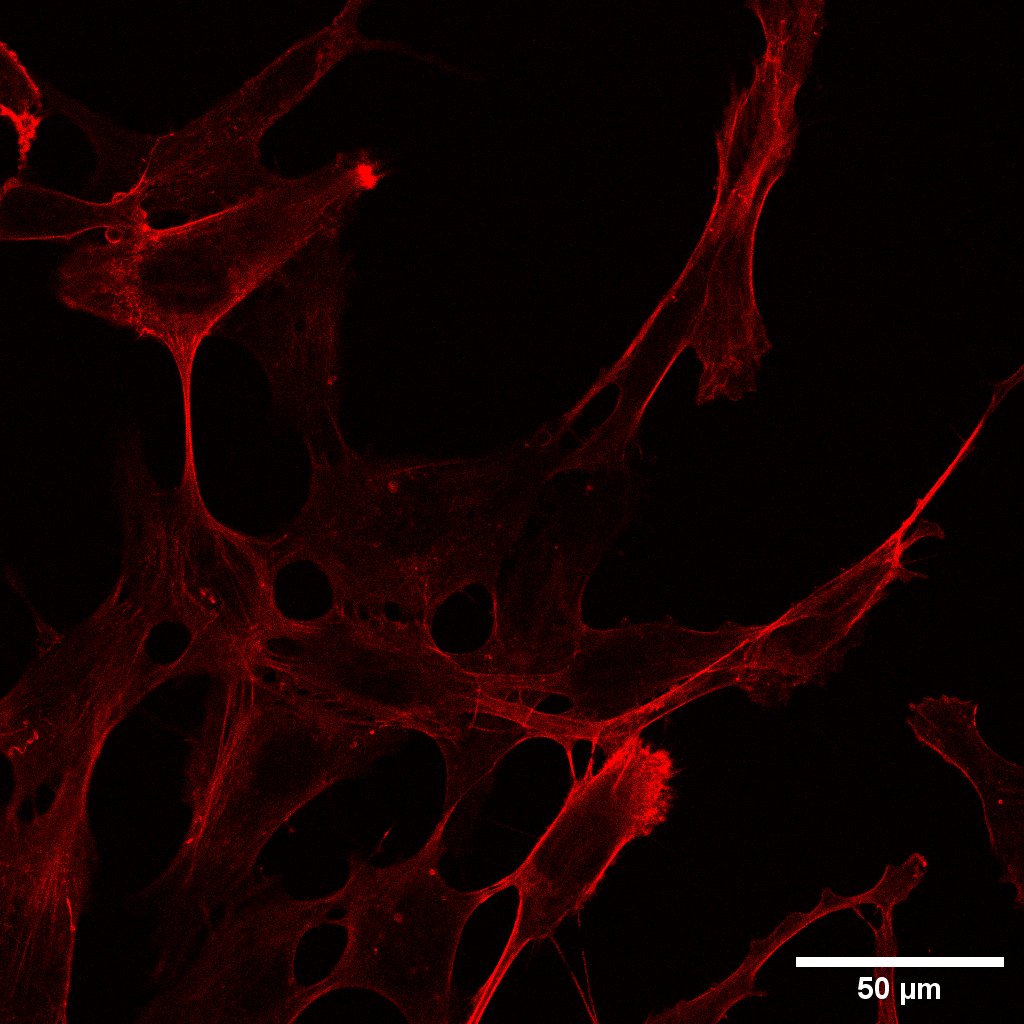

Supplement: Supplementary file 5 — Source data Fig. 1 [file 44318_2026_766_MOESM5_ESM.zip › Figure1/Fig1c/mda dmso/C3-mdamb 231 dmso.lif - Series006.jpg]

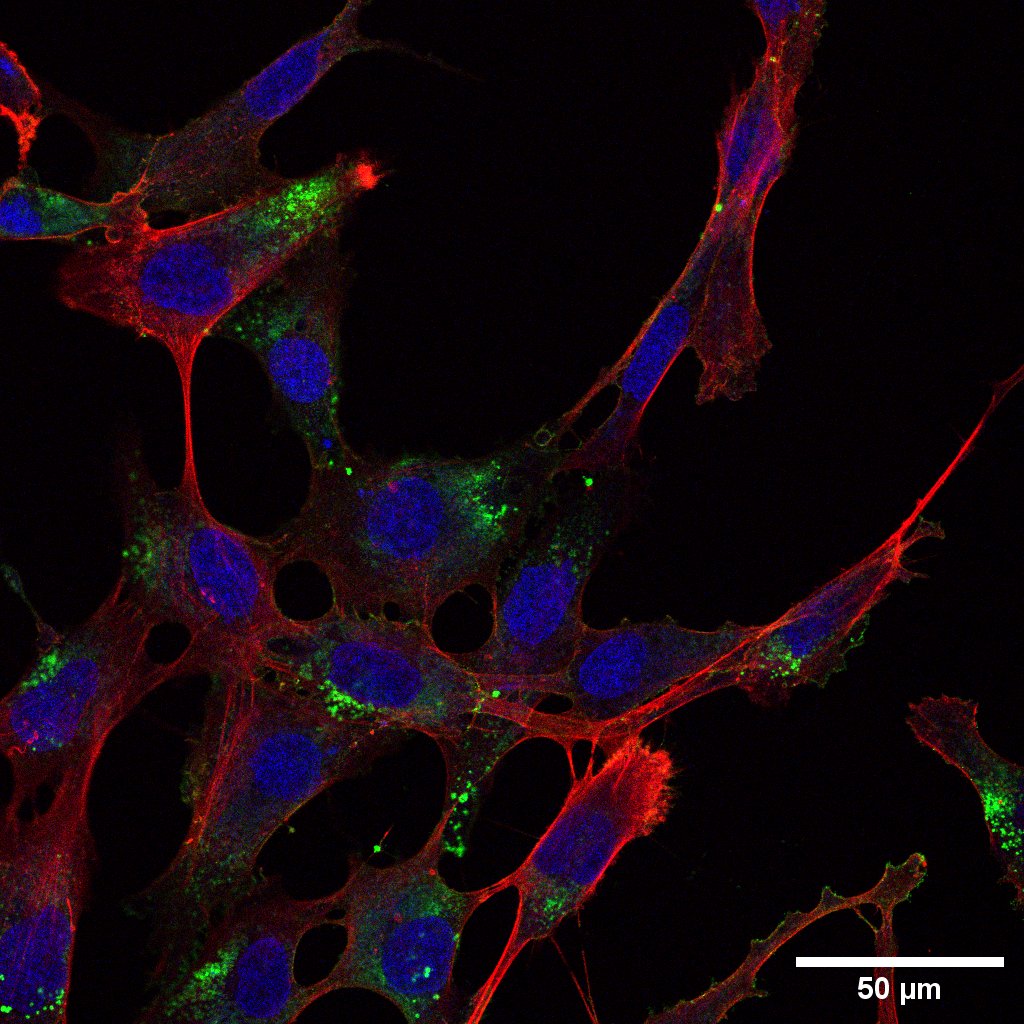

Supplement: Supplementary file 5 — Source data Fig. 1 [file 44318_2026_766_MOESM5_ESM.zip › Figure1/Fig1c/mda dmso/Composite.jpg]

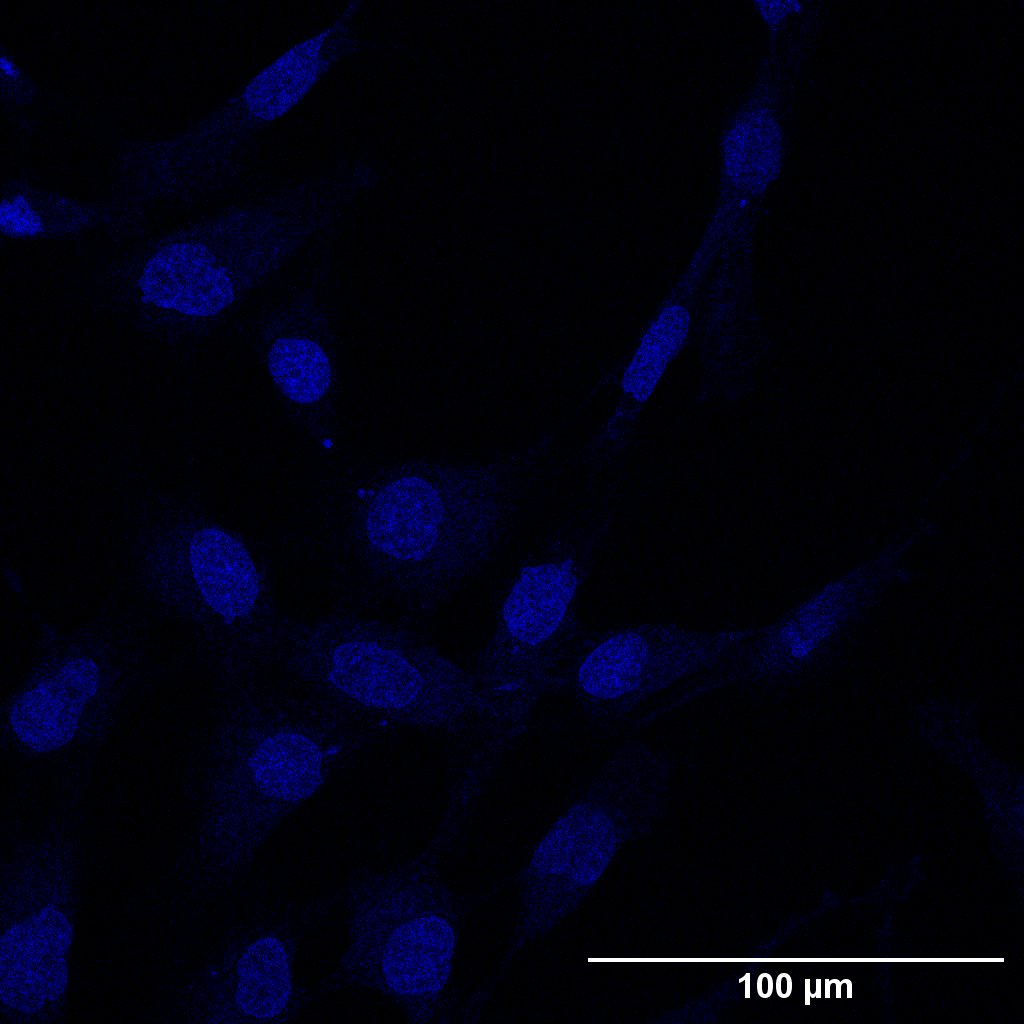

Supplement: Supplementary file 5 — Source data Fig. 1 [file 44318_2026_766_MOESM5_ESM.zip › Figure1/Fig1c/mda dmso/C1-mdamb 231 dmso.lif - Series006.jpg]

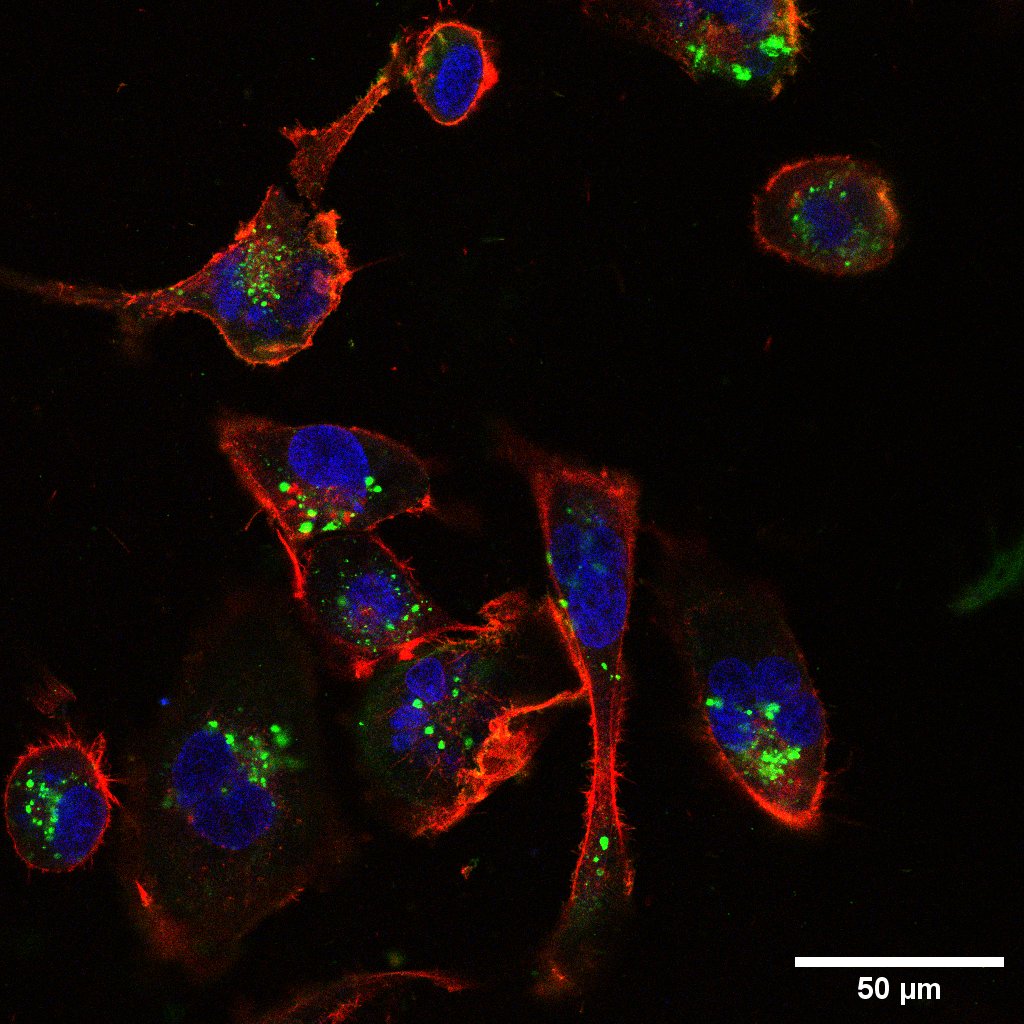

Supplement: Supplementary file 5 — Source data Fig. 1 [file 44318_2026_766_MOESM5_ESM.zip › Figure1/Fig1c/mda rev/mdamb231-rev.lif - Series047.jpg]

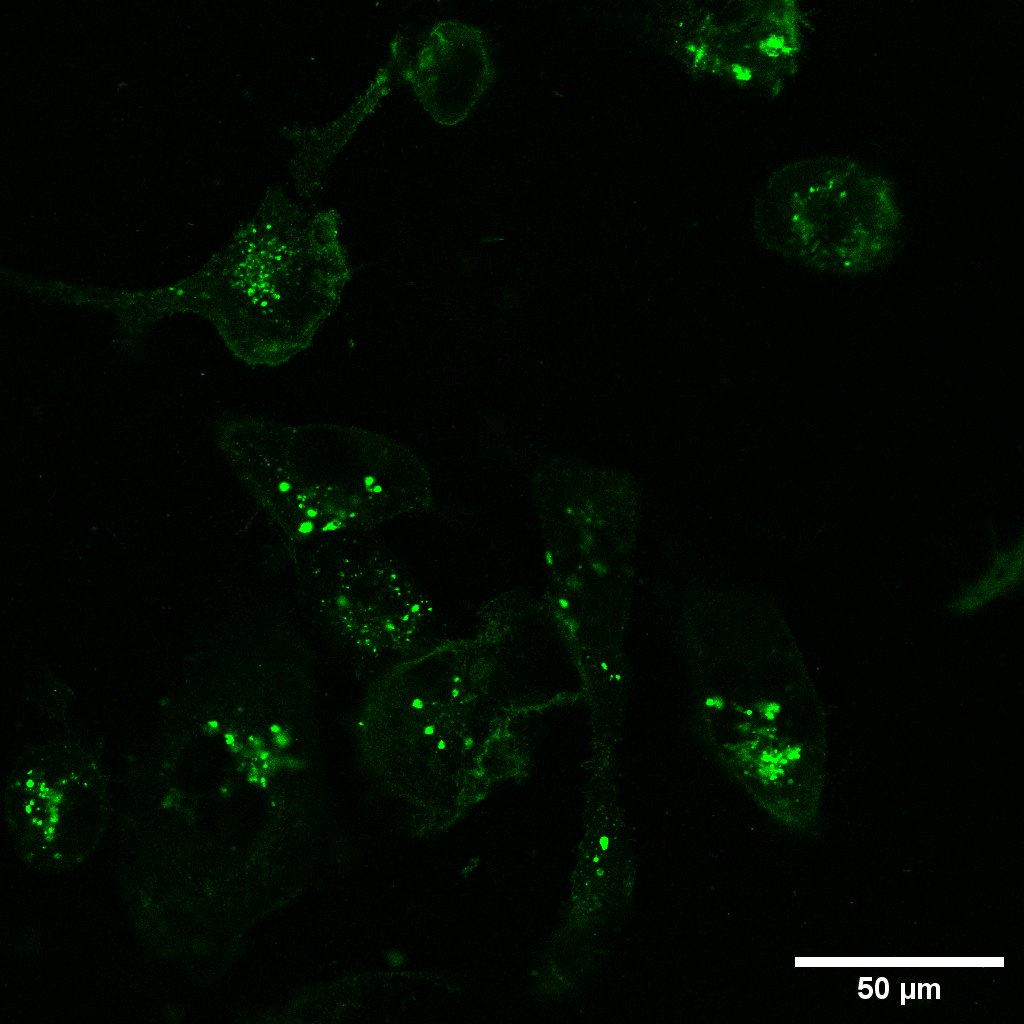

Supplement: Supplementary file 5 — Source data Fig. 1 [file 44318_2026_766_MOESM5_ESM.zip › Figure1/Fig1c/mda rev/C2-mdamb231-rev.lif - Series047.jpg]

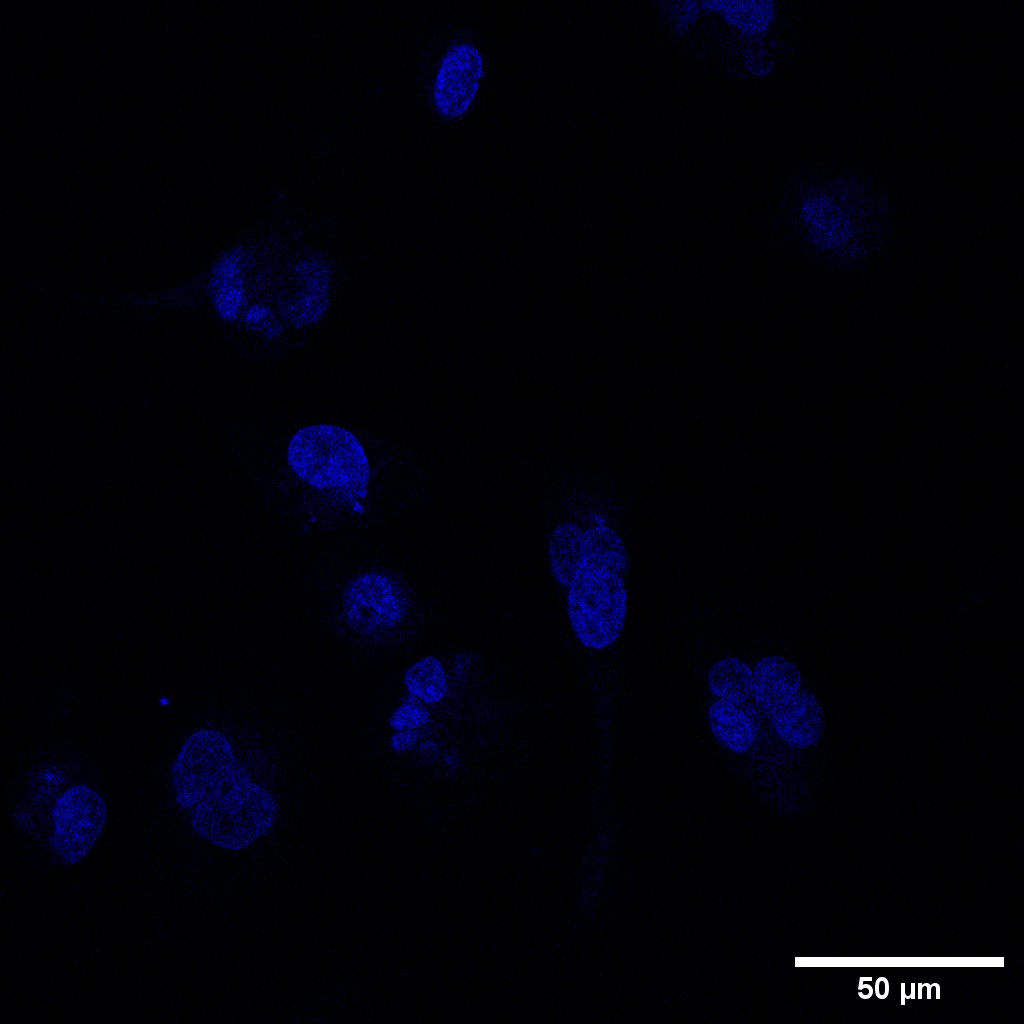

Supplement: Supplementary file 5 — Source data Fig. 1 [file 44318_2026_766_MOESM5_ESM.zip › Figure1/Fig1c/mda rev/C1-mdamb231-rev.lif - Series047.jpg]

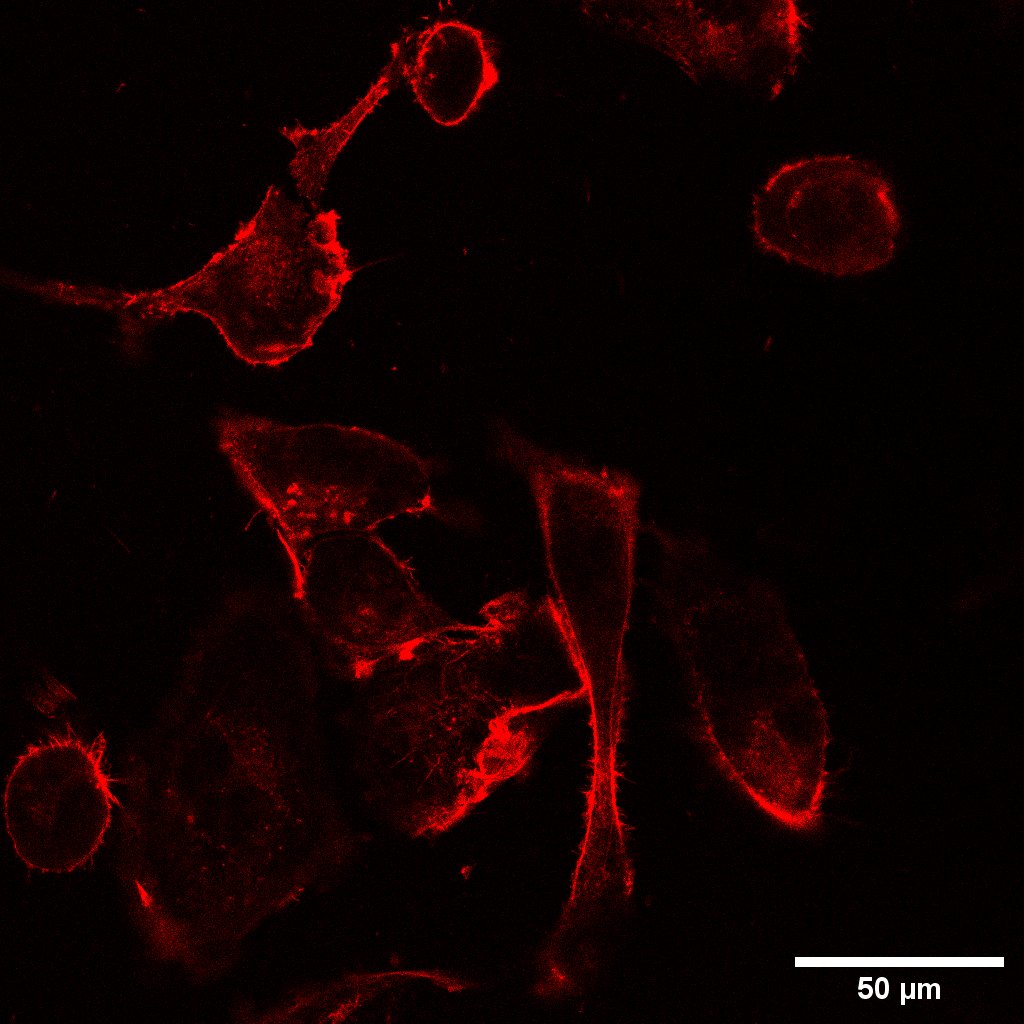

Supplement: Supplementary file 5 — Source data Fig. 1 [file 44318_2026_766_MOESM5_ESM.zip › Figure1/Fig1c/mda rev/C3-mdamb231-rev.lif - Series047.jpg]

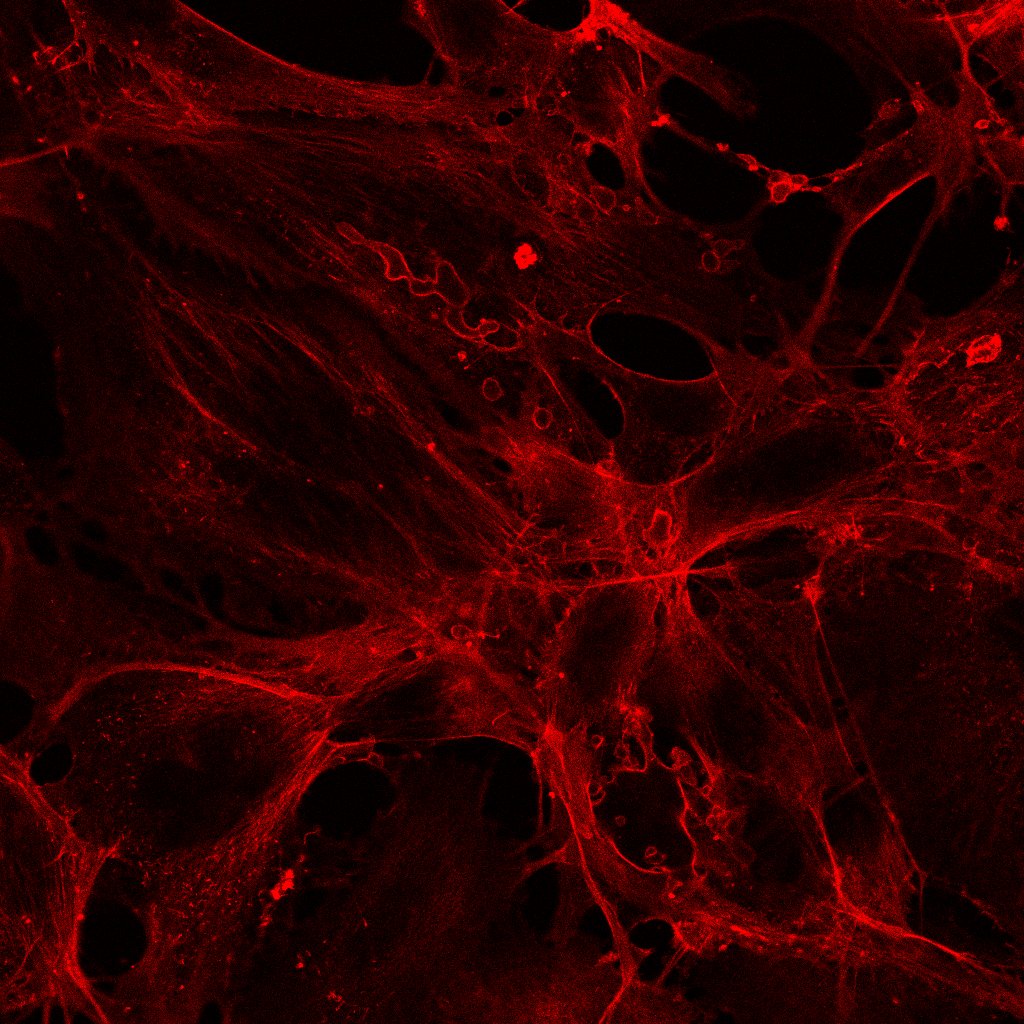

Supplement: Supplementary file 5 — Source data Fig. 1 [file 44318_2026_766_MOESM5_ESM.zip › Figure1/Fig1B/bt549 rev/C3-003.lif - Series006.jpg]

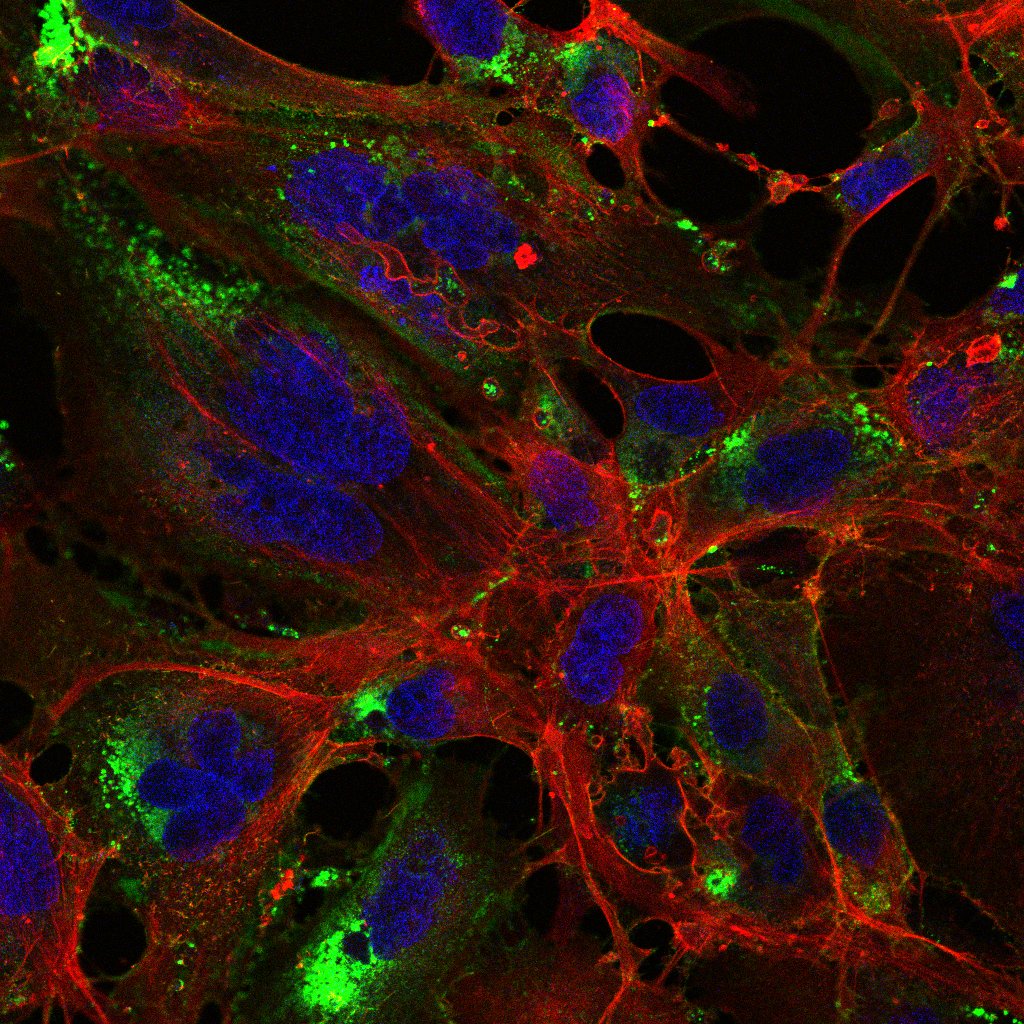

Supplement: Supplementary file 5 — Source data Fig. 1 [file 44318_2026_766_MOESM5_ESM.zip › Figure1/Fig1B/bt549 rev/003.lif - Series006.jpg]

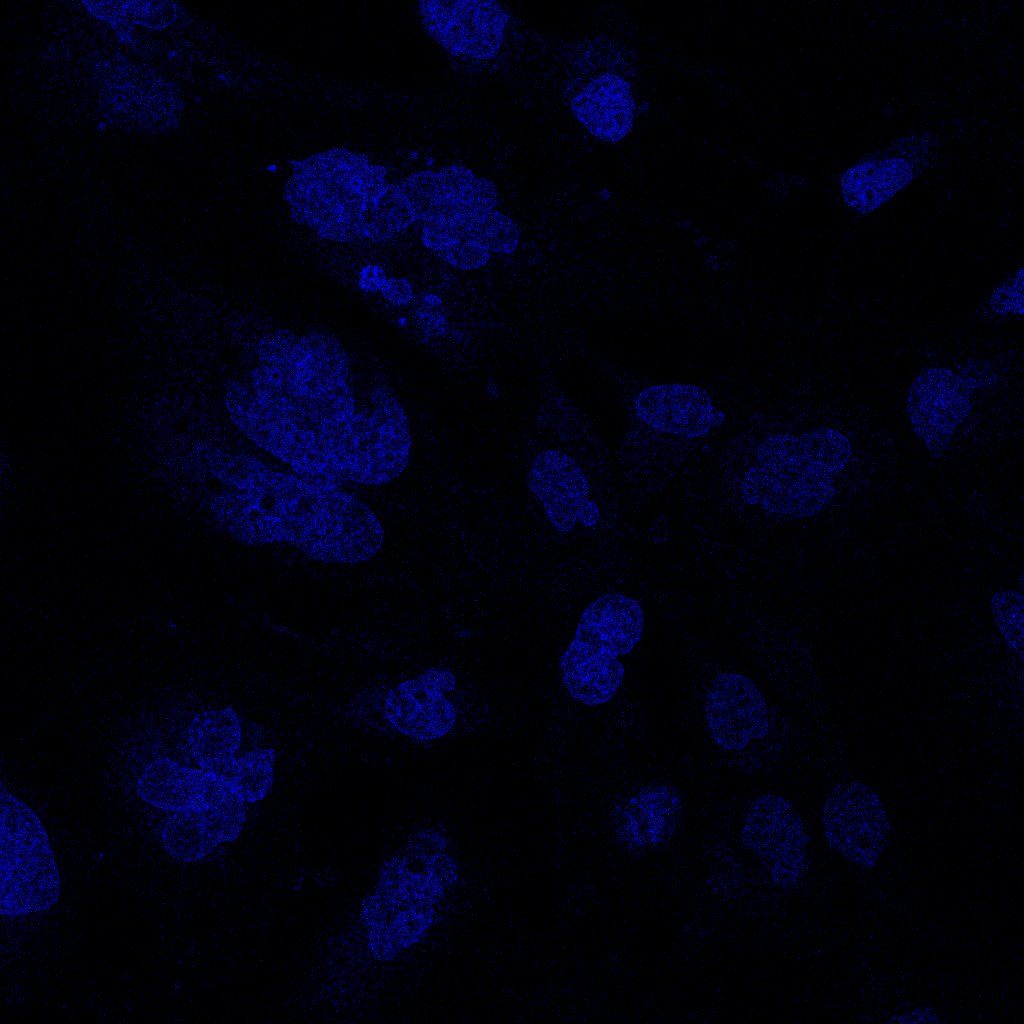

Supplement: Supplementary file 5 — Source data Fig. 1 [file 44318_2026_766_MOESM5_ESM.zip › Figure1/Fig1B/bt549 rev/C1-003.lif - Series006.jpg]

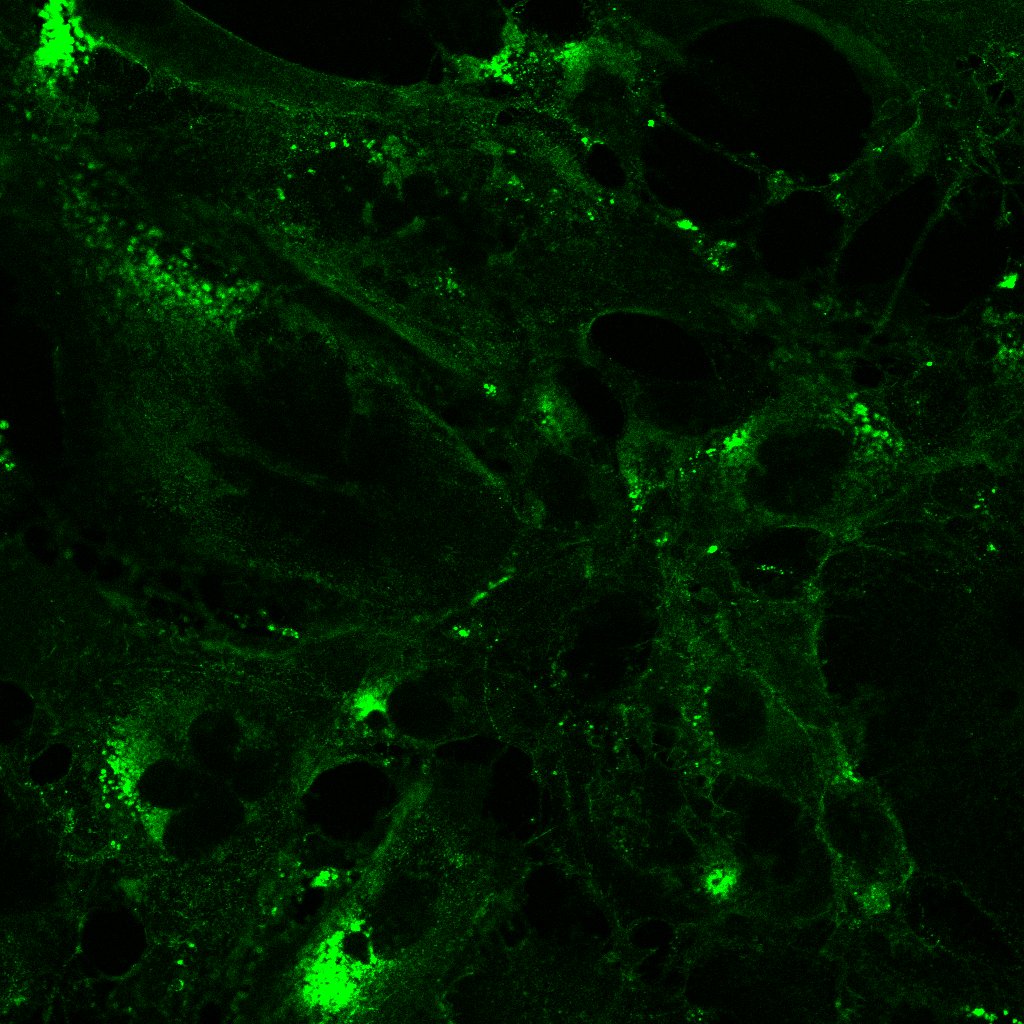

Supplement: Supplementary file 5 — Source data Fig. 1 [file 44318_2026_766_MOESM5_ESM.zip › Figure1/Fig1B/bt549 rev/C2-003.lif - Series006.jpg]

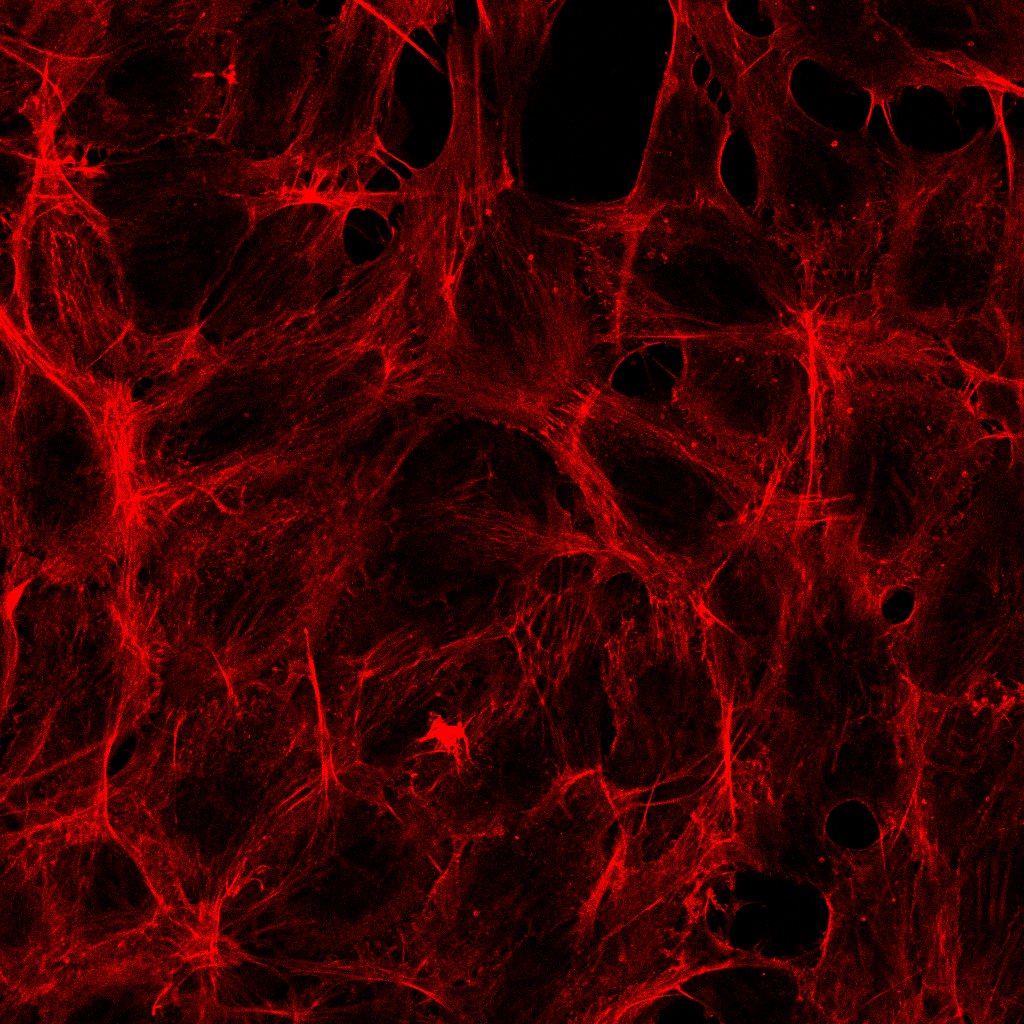

Supplement: Supplementary file 5 — Source data Fig. 1 [file 44318_2026_766_MOESM5_ESM.zip › Figure1/Fig1B/bt549 dmso/C3-Project003.lif - Series003.jpg]

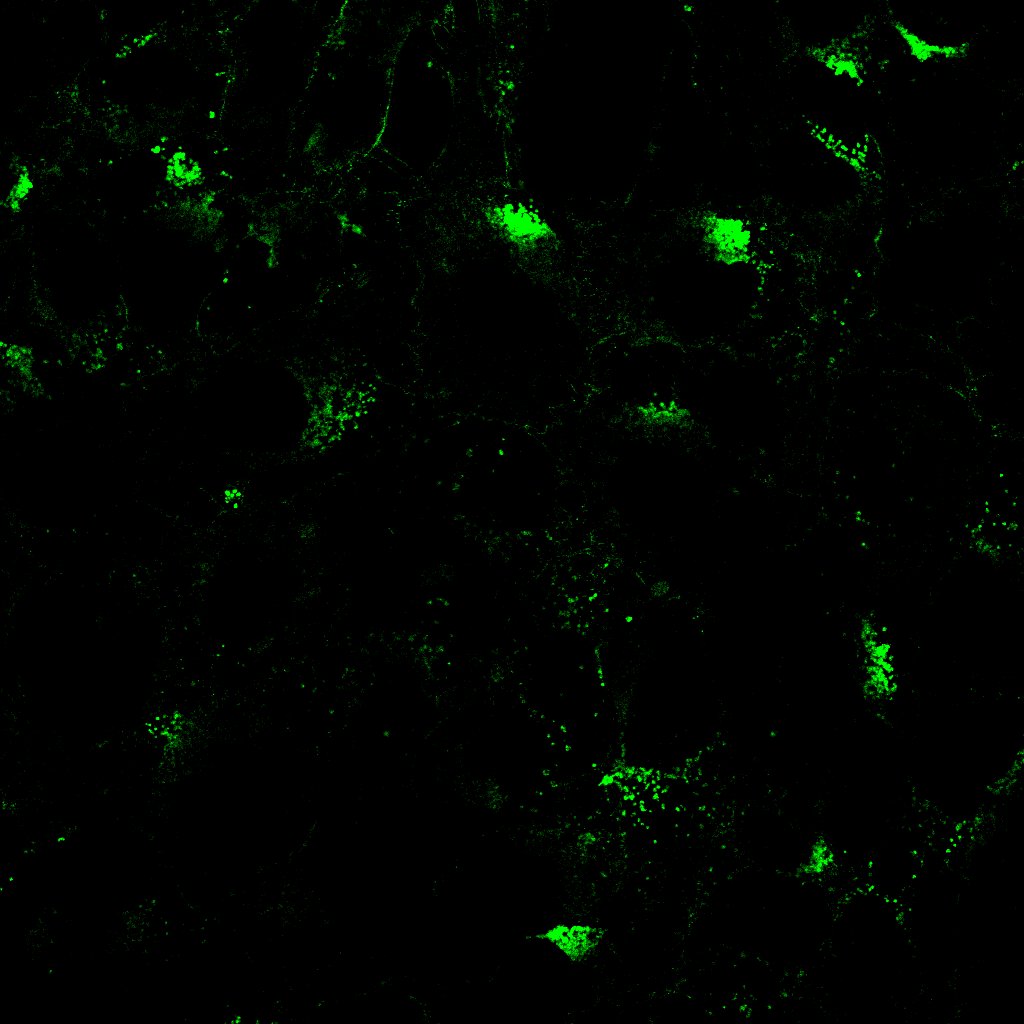

Supplement: Supplementary file 5 — Source data Fig. 1 [file 44318_2026_766_MOESM5_ESM.zip › Figure1/Fig1B/bt549 dmso/C2-Project003.lif - Series003.jpg]

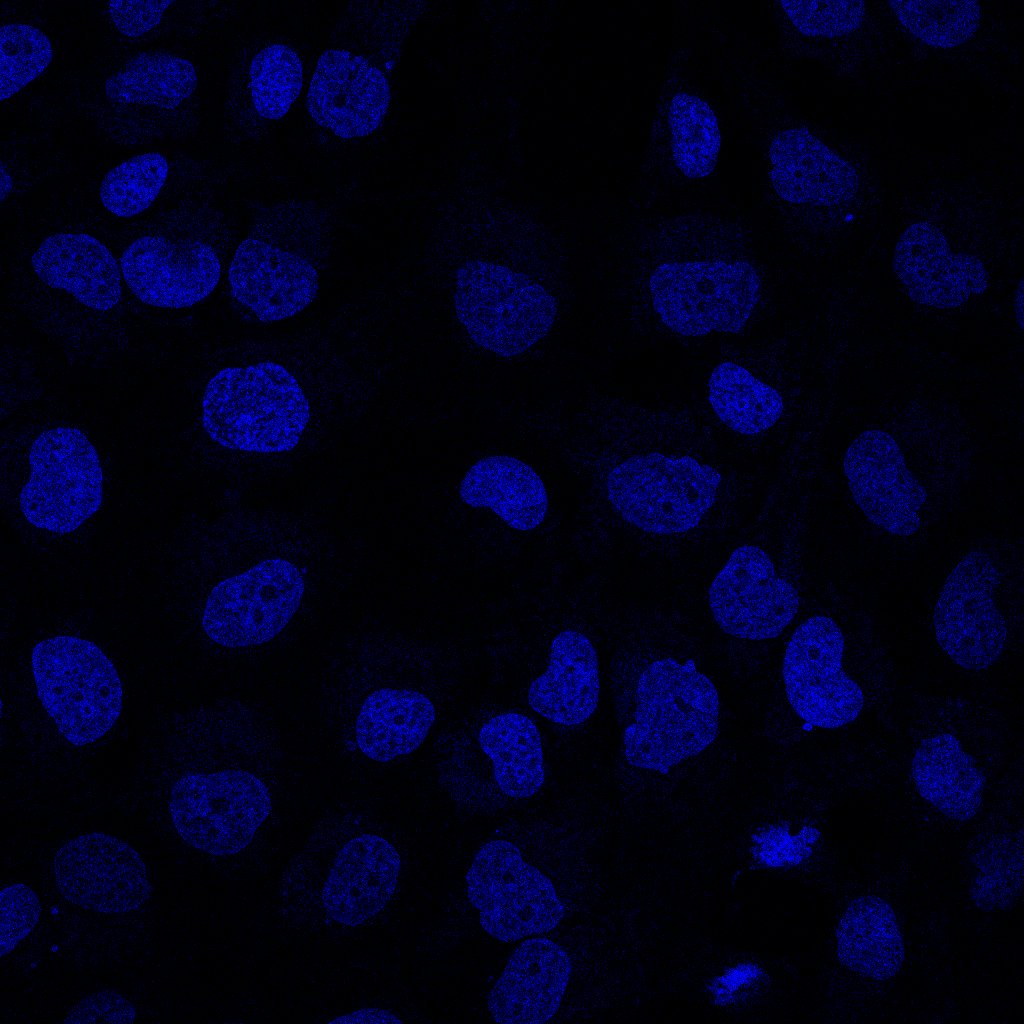

Supplement: Supplementary file 5 — Source data Fig. 1 [file 44318_2026_766_MOESM5_ESM.zip › Figure1/Fig1B/bt549 dmso/C1-Project003.lif - Series003.jpg]

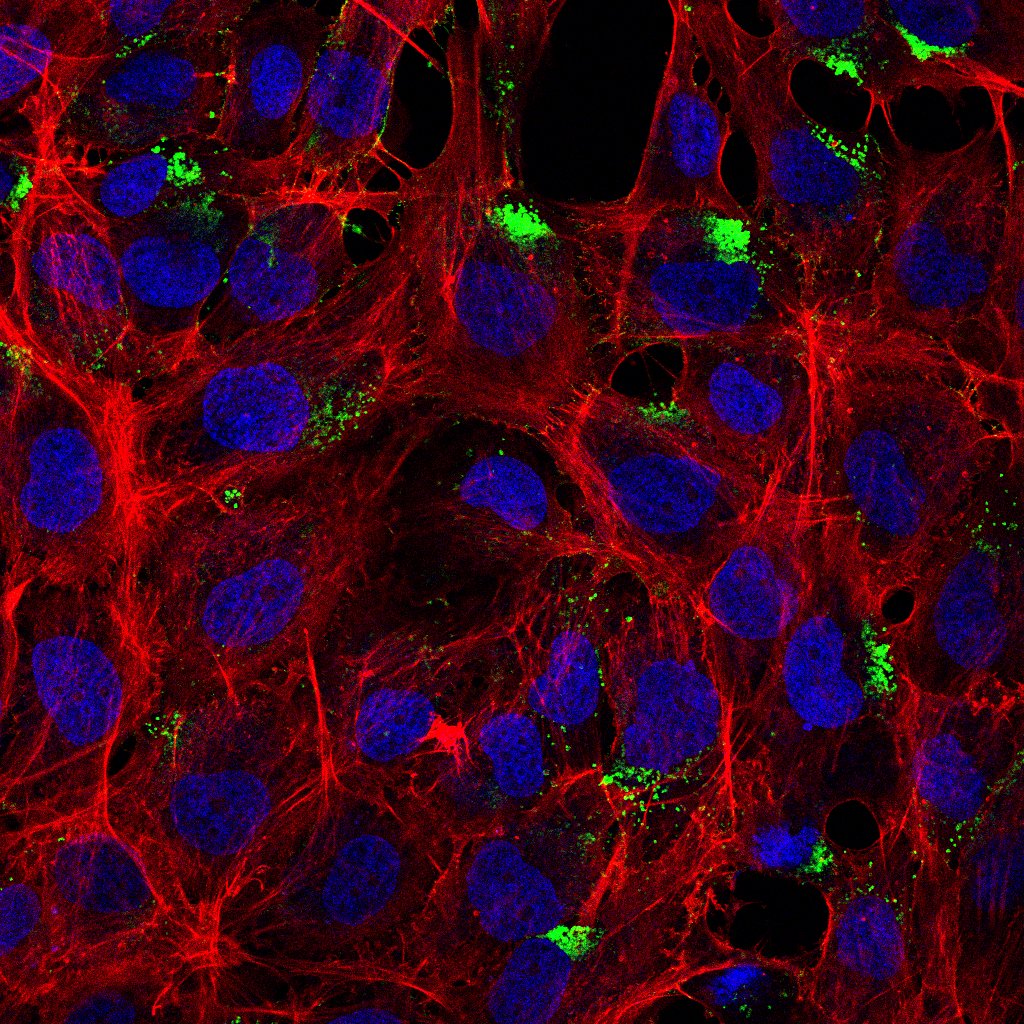

Supplement: Supplementary file 5 — Source data Fig. 1 [file 44318_2026_766_MOESM5_ESM.zip › Figure1/Fig1B/bt549 dmso/Project003.lif - Series003.jpg]

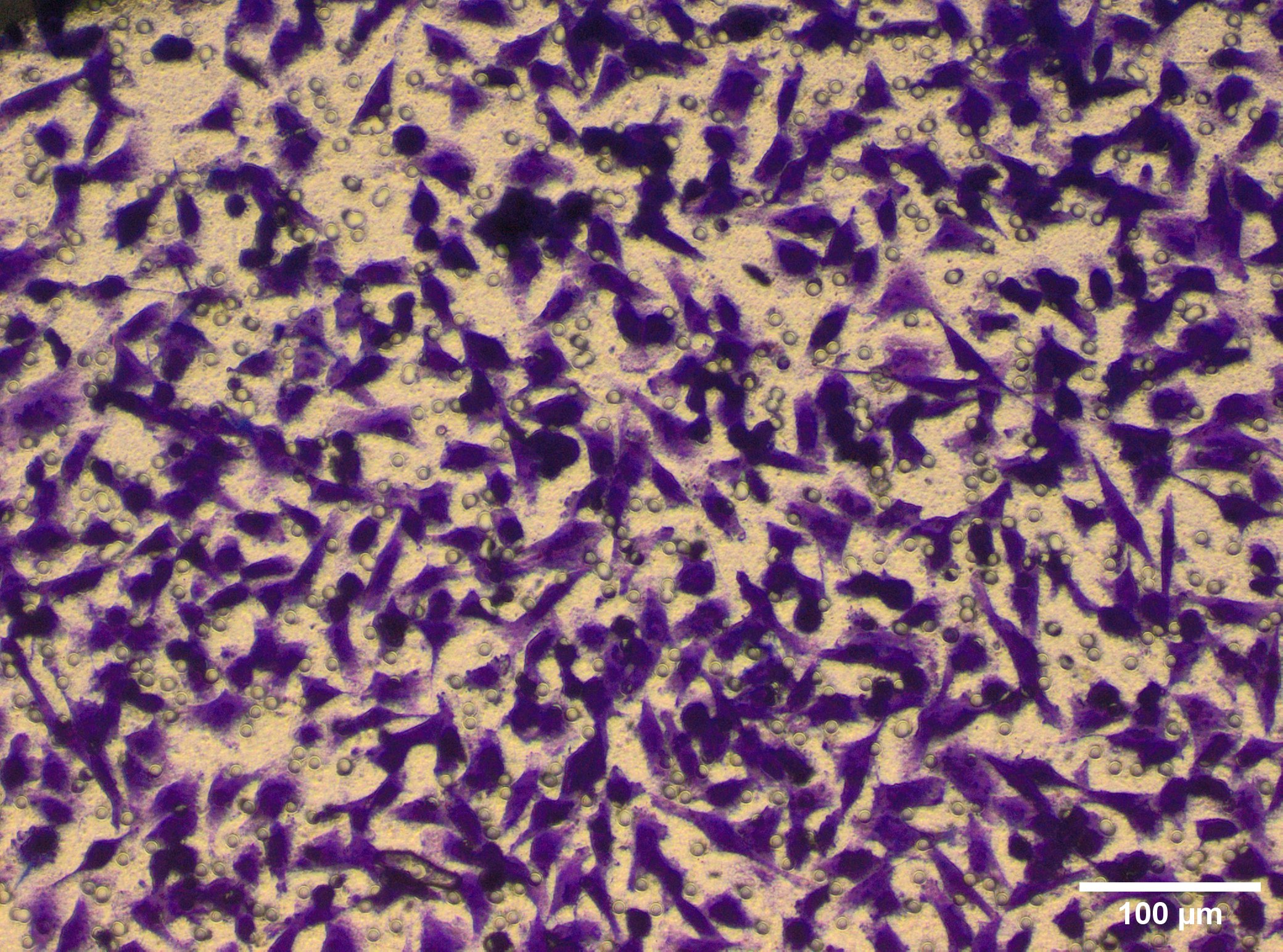

Supplement: Supplementary file 6 — Source data Fig. 2 [file 44318_2026_766_MOESM6_ESM.zip › Figure2/Fig2D/bt rev ev migImage_18052-1.jpg]

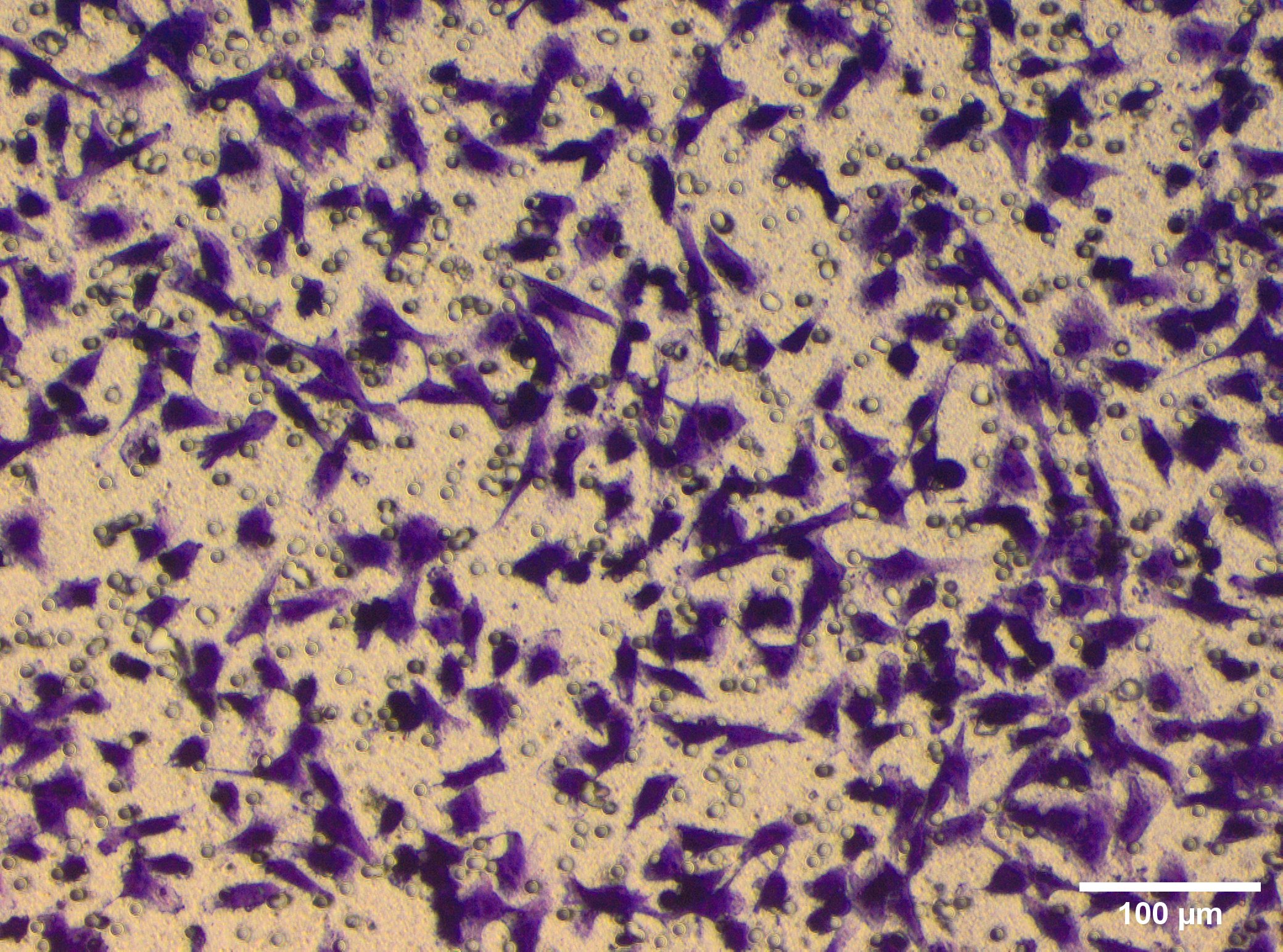

Supplement: Supplementary file 6 — Source data Fig. 2 [file 44318_2026_766_MOESM6_ESM.zip › Figure2/Fig2D/bt dmso ev migraImage_18022-1.jpg]

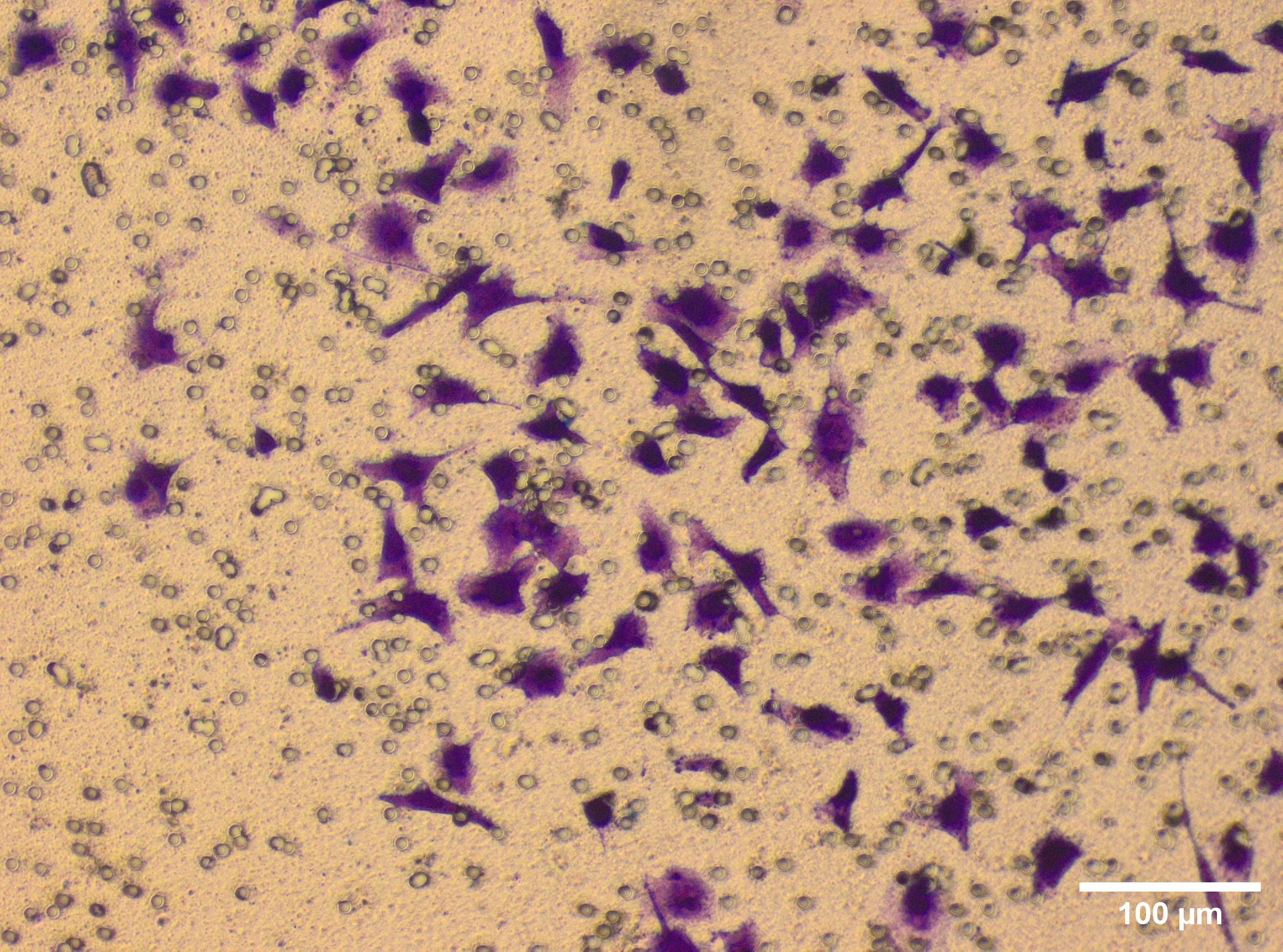

Supplement: Supplementary file 6 — Source data Fig. 2 [file 44318_2026_766_MOESM6_ESM.zip › Figure2/Fig2D/bt dmso ev invImage_18176 94-1.jpg]

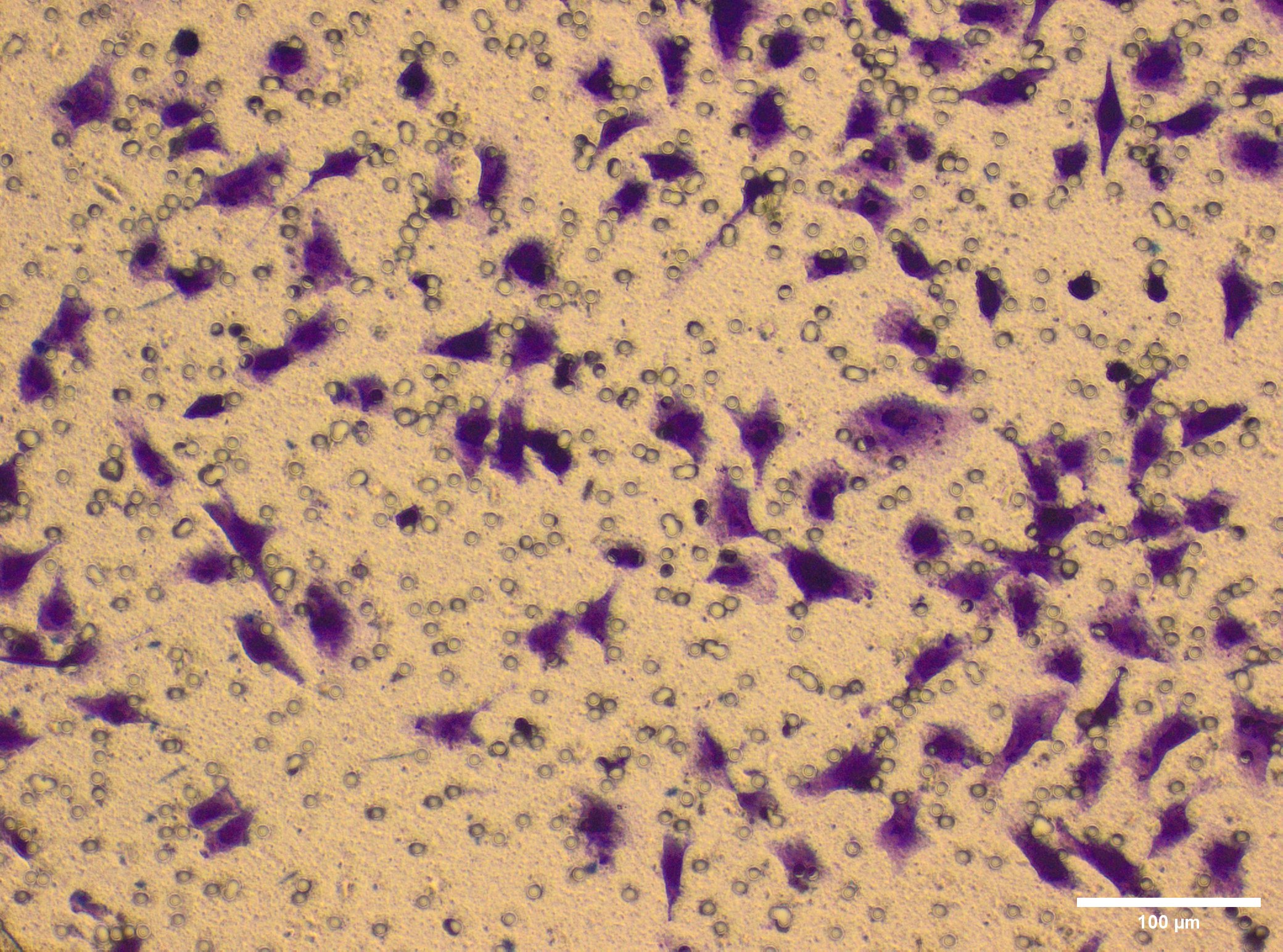

Supplement: Supplementary file 6 — Source data Fig. 2 [file 44318_2026_766_MOESM6_ESM.zip › Figure2/Fig2D/Image_18163pbs invasion bt pbs invasion.jpg]

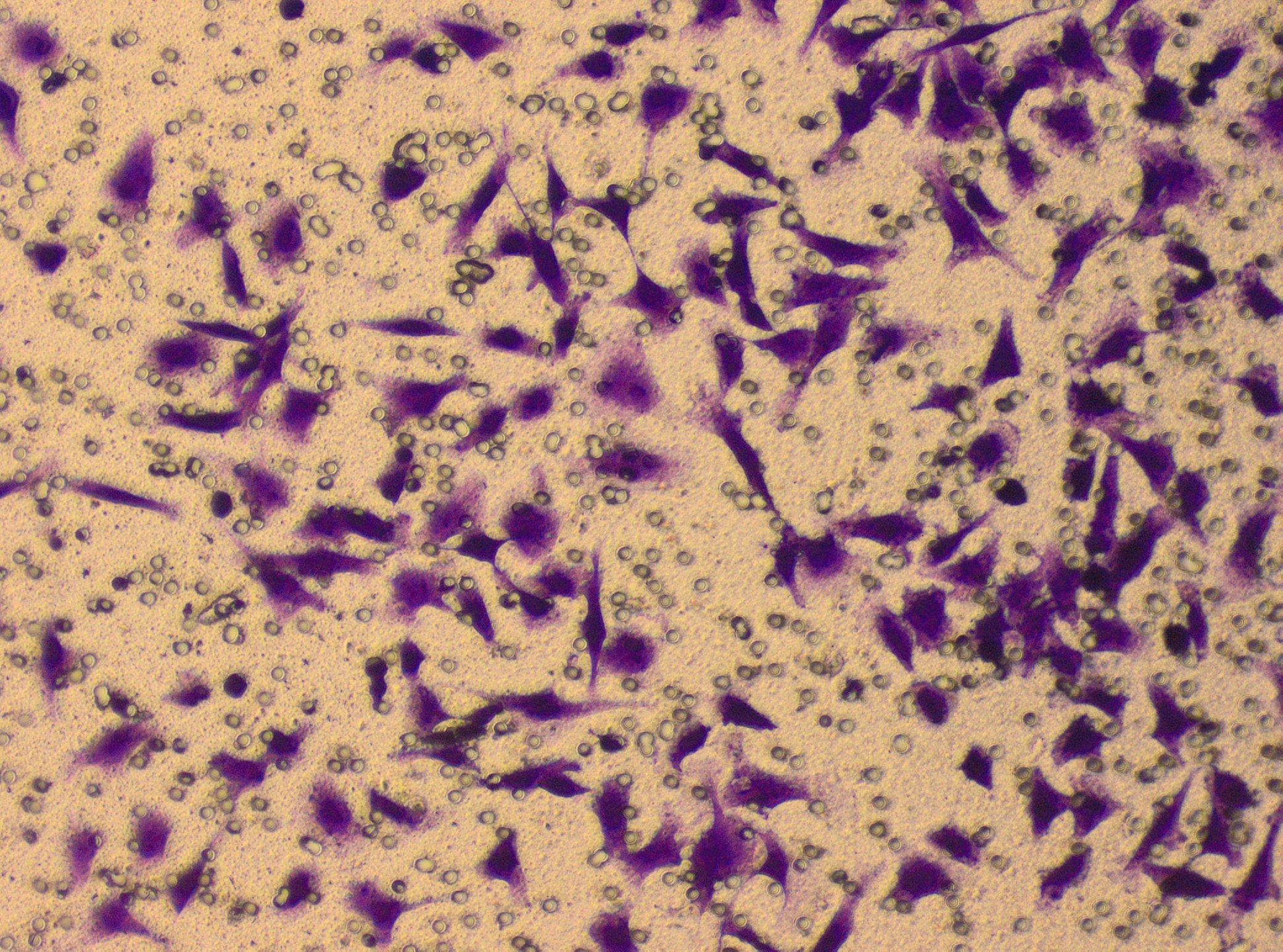

Supplement: Supplementary file 6 — Source data Fig. 2 [file 44318_2026_766_MOESM6_ESM.zip › Figure2/Fig2D/bt rev ev invImage_18182 151.jpg]

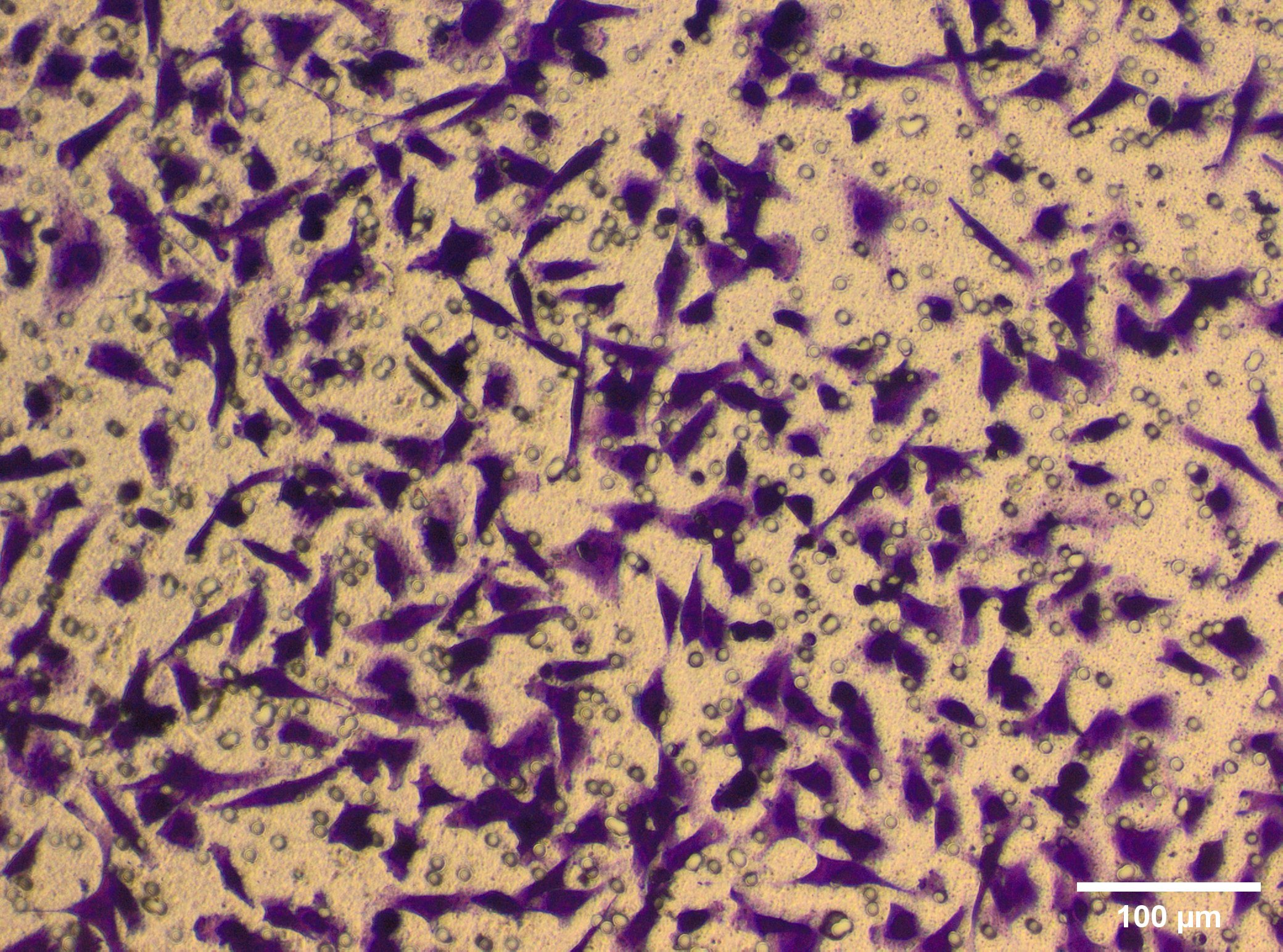

Supplement: Supplementary file 6 — Source data Fig. 2 [file 44318_2026_766_MOESM6_ESM.zip › Figure2/Fig2D/Image_18084pbs migration bt pbs migration.tif]

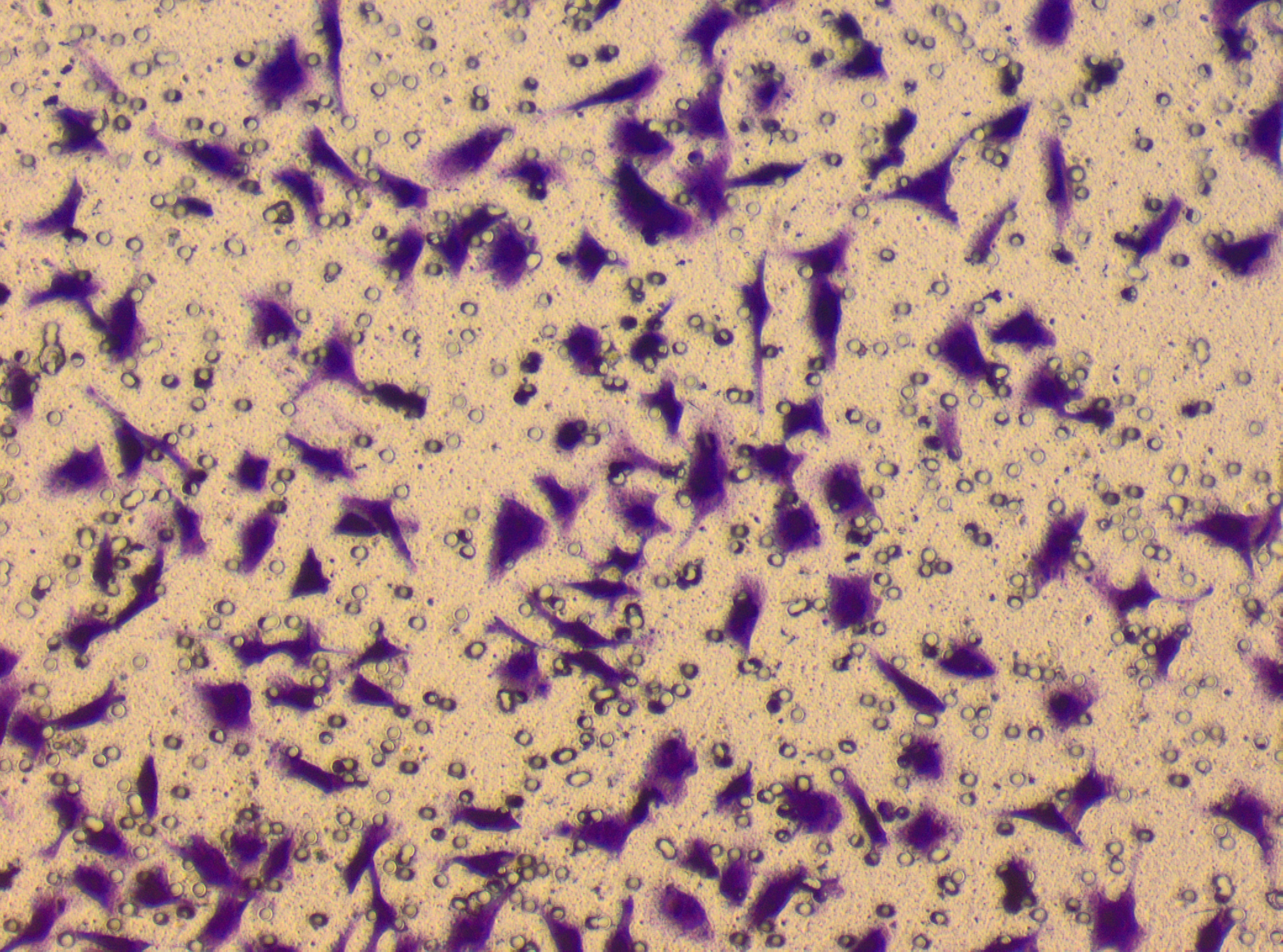

Supplement: Supplementary file 6 — Source data Fig. 2 [file 44318_2026_766_MOESM6_ESM.zip › Figure2/Fig2F/MDA REV EV INVa Image_4467.tif]

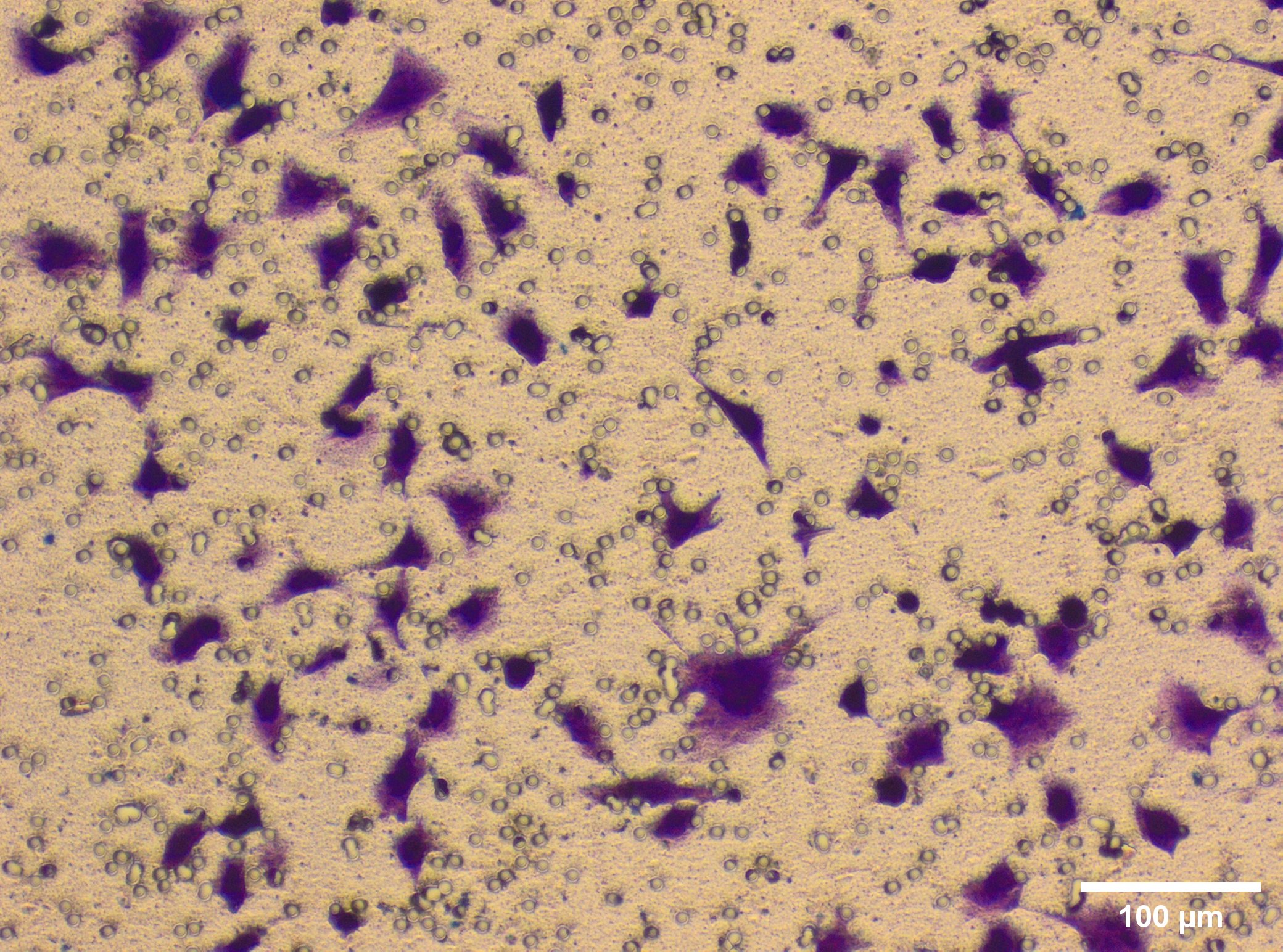

Supplement: Supplementary file 6 — Source data Fig. 2 [file 44318_2026_766_MOESM6_ESM.zip › Figure2/Fig2F/Image_4436 mda pbs invasion.jpg]

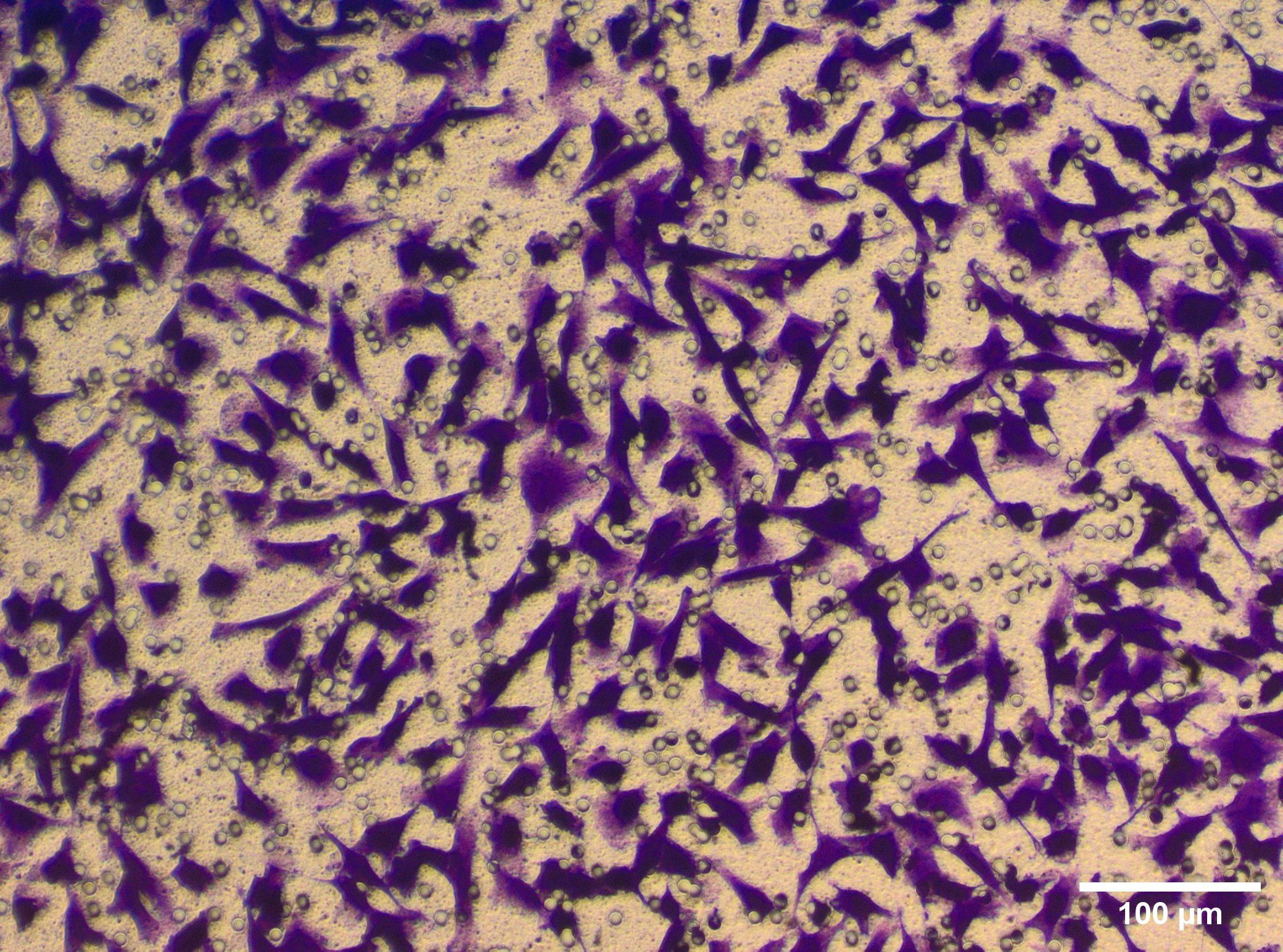

Supplement: Supplementary file 6 — Source data Fig. 2 [file 44318_2026_766_MOESM6_ESM.zip › Figure2/Fig2F/mda rev ev migraImage_4343-1.jpg]

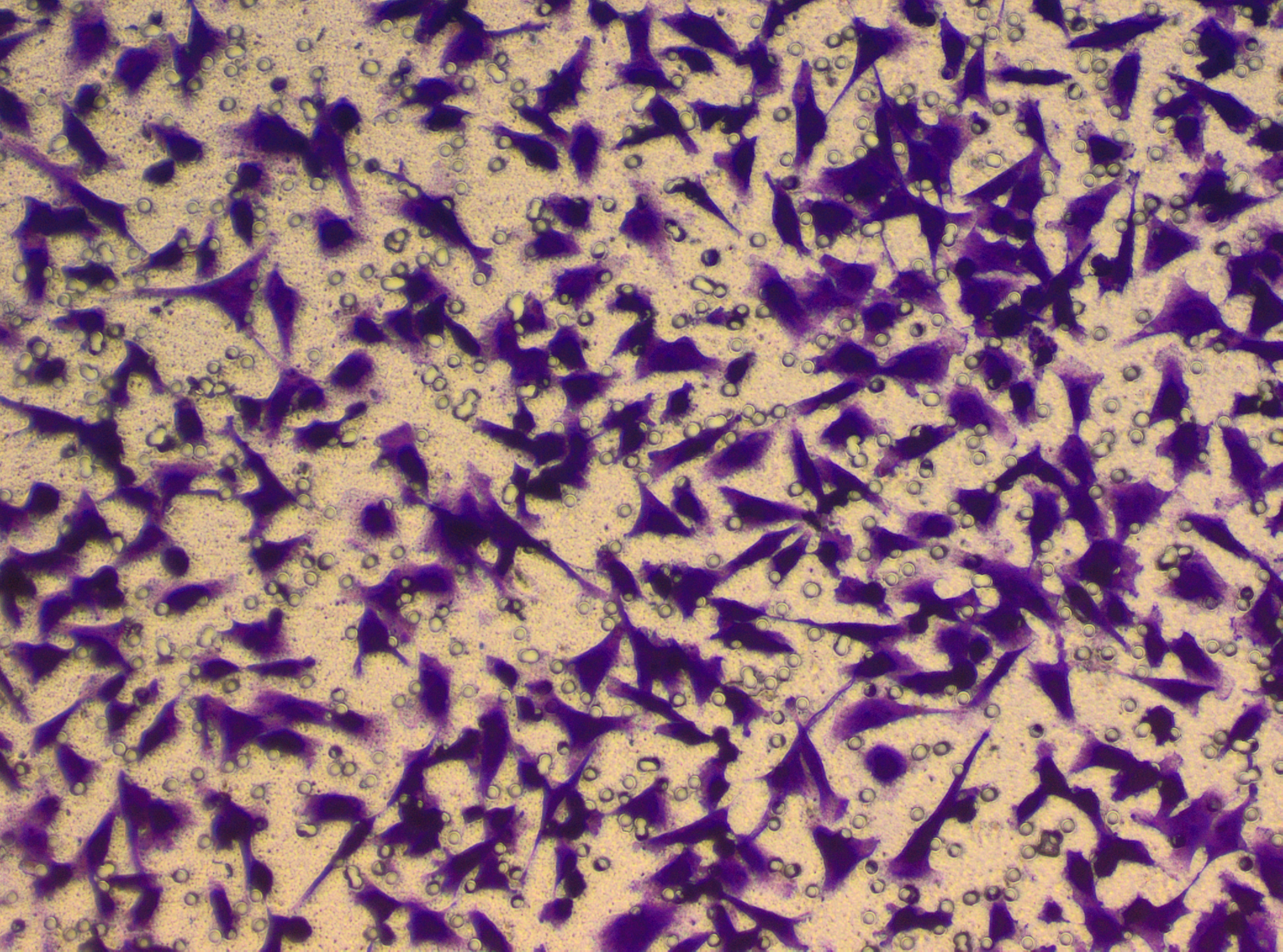

Supplement: Supplementary file 6 — Source data Fig. 2 [file 44318_2026_766_MOESM6_ESM.zip › Figure2/Fig2F/mda dmso ev migrImage_4291.tif]

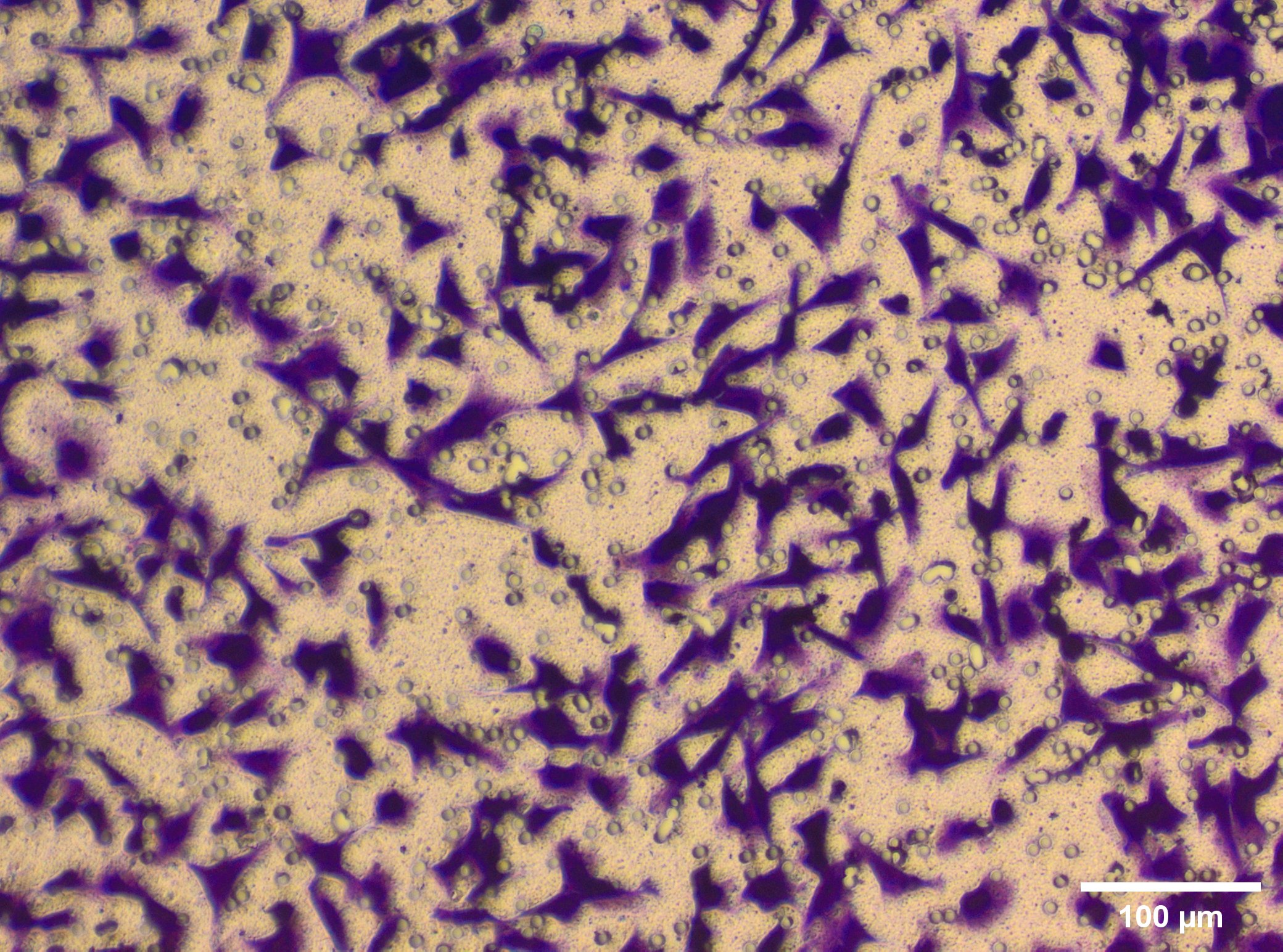

Supplement: Supplementary file 6 — Source data Fig. 2 [file 44318_2026_766_MOESM6_ESM.zip › Figure2/Fig2F/Image_4281 mda pbs mig.jpg]

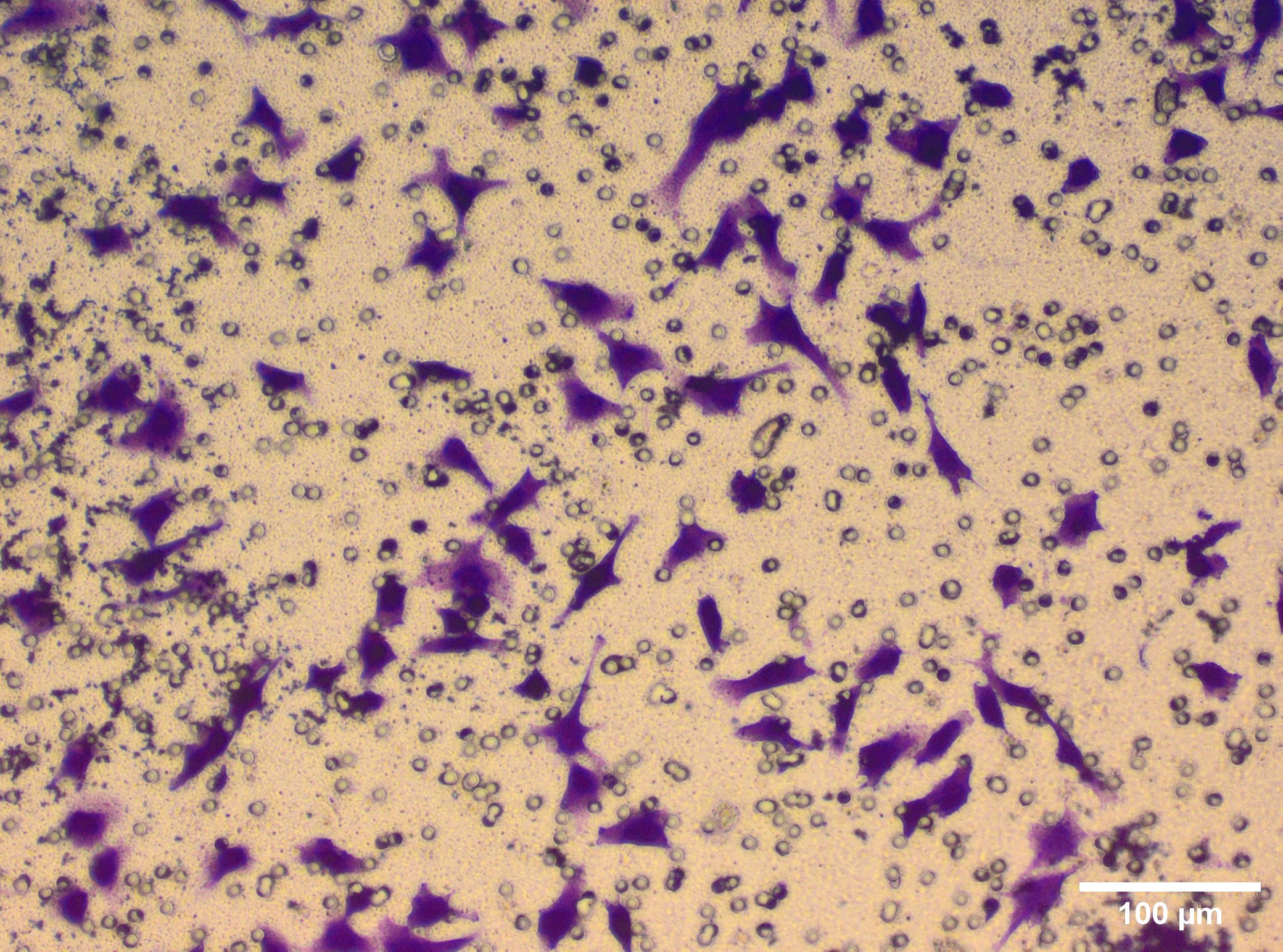

Supplement: Supplementary file 6 — Source data Fig. 2 [file 44318_2026_766_MOESM6_ESM.zip › Figure2/Fig2F/mda dmso ev invImage_4441-1.jpg]

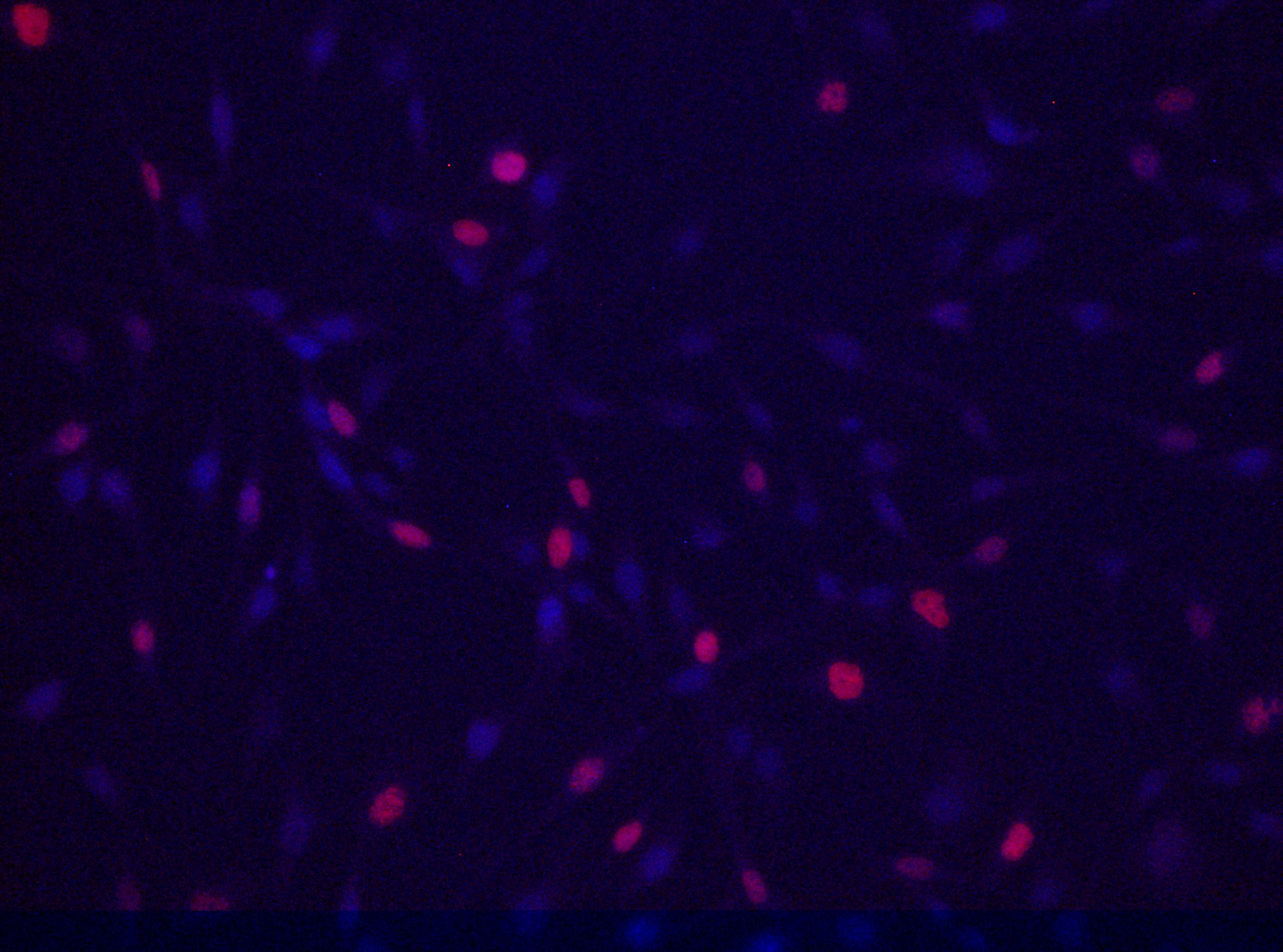

Supplement: Supplementary file 6 — Source data Fig. 2 [file 44318_2026_766_MOESM6_ESM.zip › Figure2/Fig2A/DMSO/Composite.jpg]

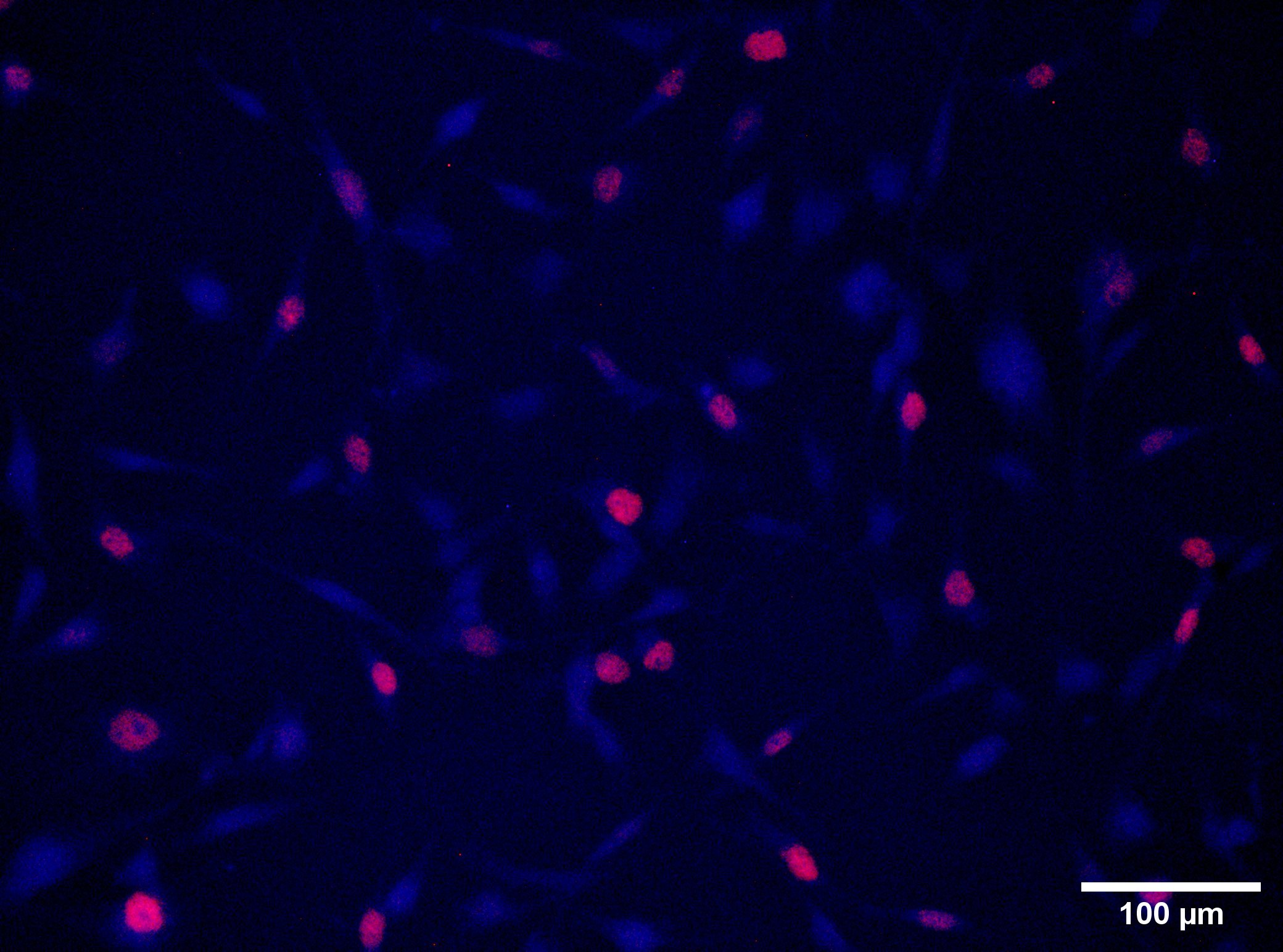

Supplement: Supplementary file 6 — Source data Fig. 2 [file 44318_2026_766_MOESM6_ESM.zip › Figure2/Fig2A/REV/Composite-2.jpg]

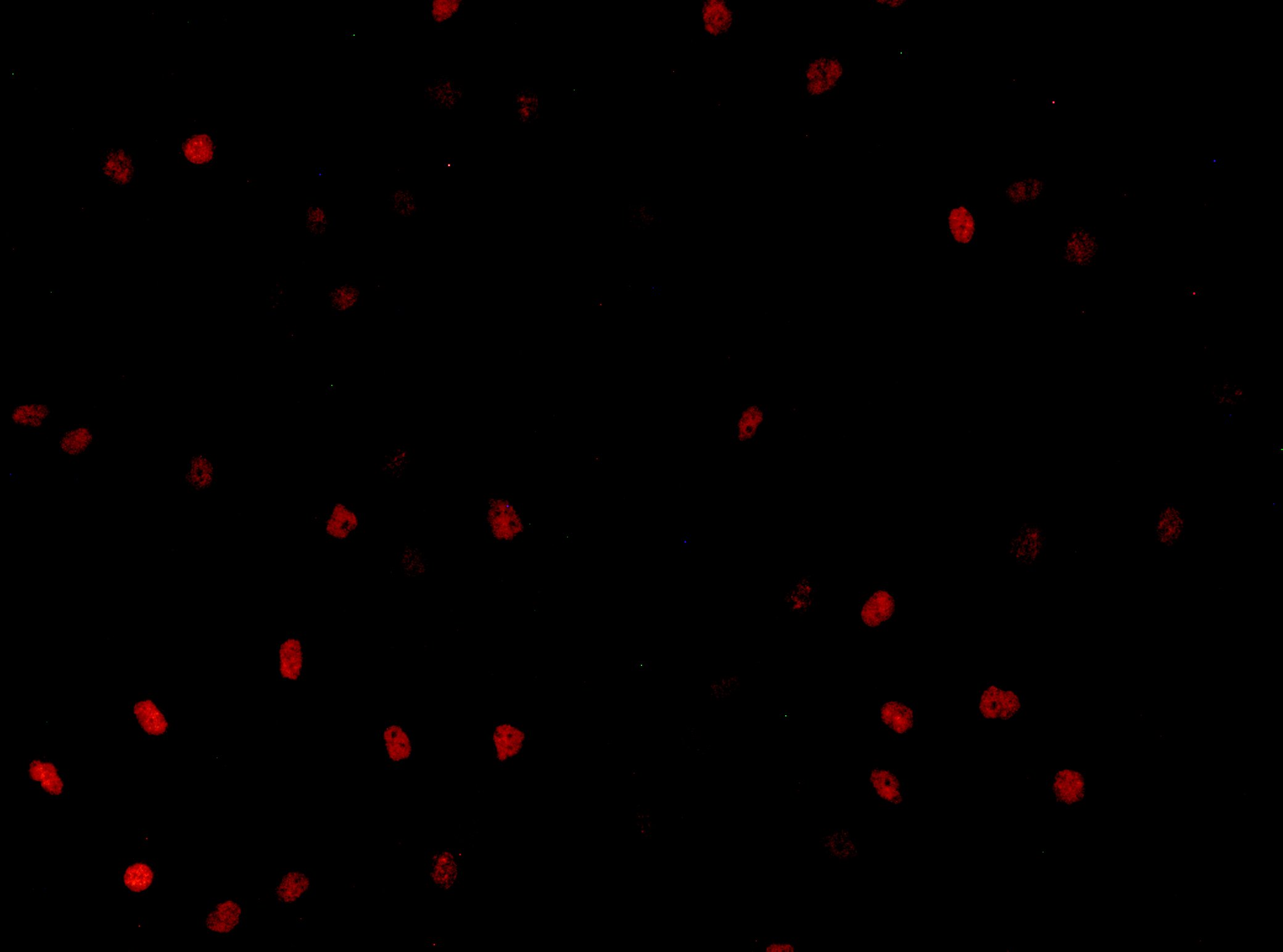

Supplement: Supplementary file 6 — Source data Fig. 2 [file 44318_2026_766_MOESM6_ESM.zip › Figure2/Fig2A/PBS/edu.jpg]

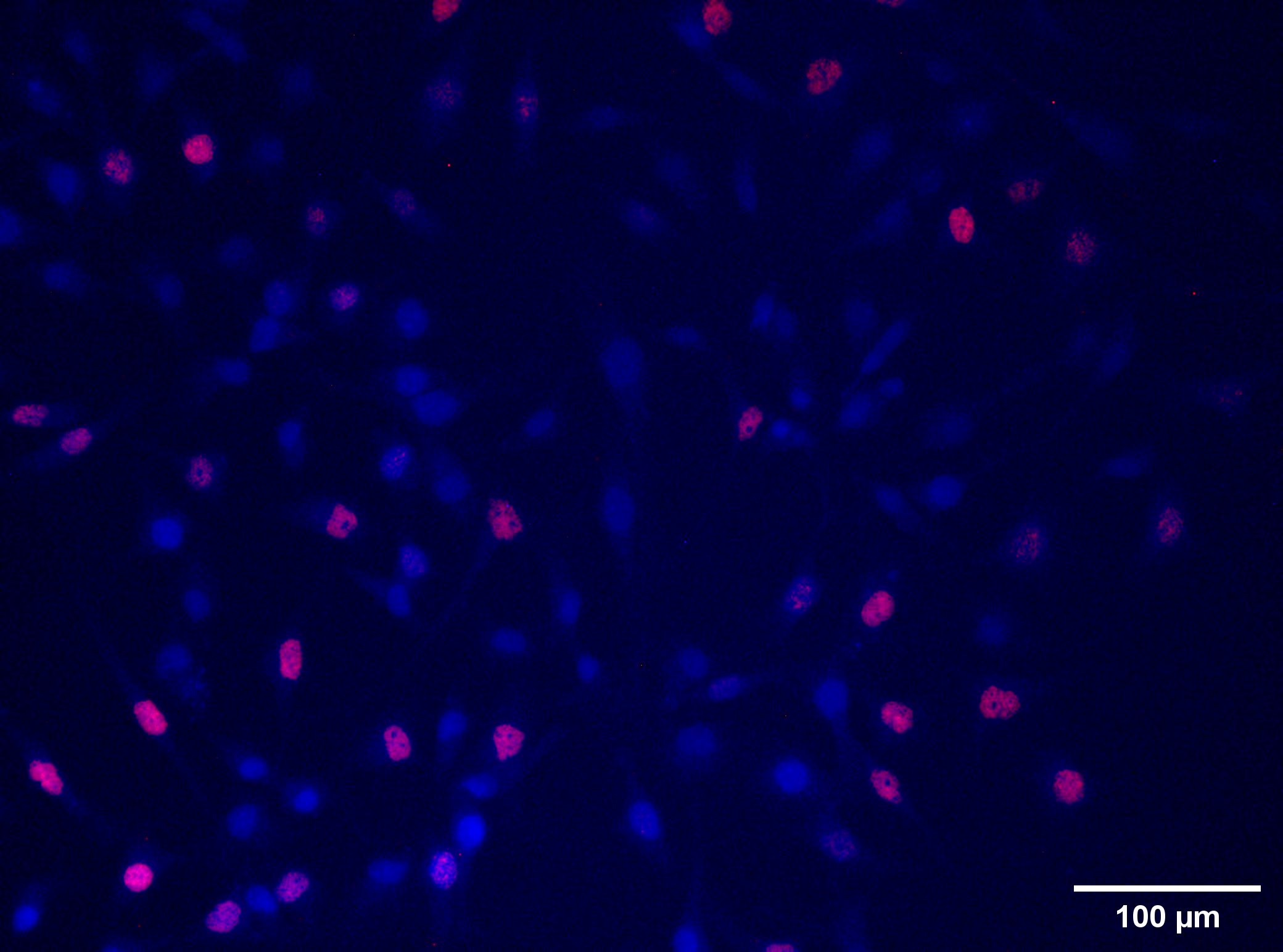

Supplement: Supplementary file 6 — Source data Fig. 2 [file 44318_2026_766_MOESM6_ESM.zip › Figure2/Fig2A/PBS/Composite50.jpg]

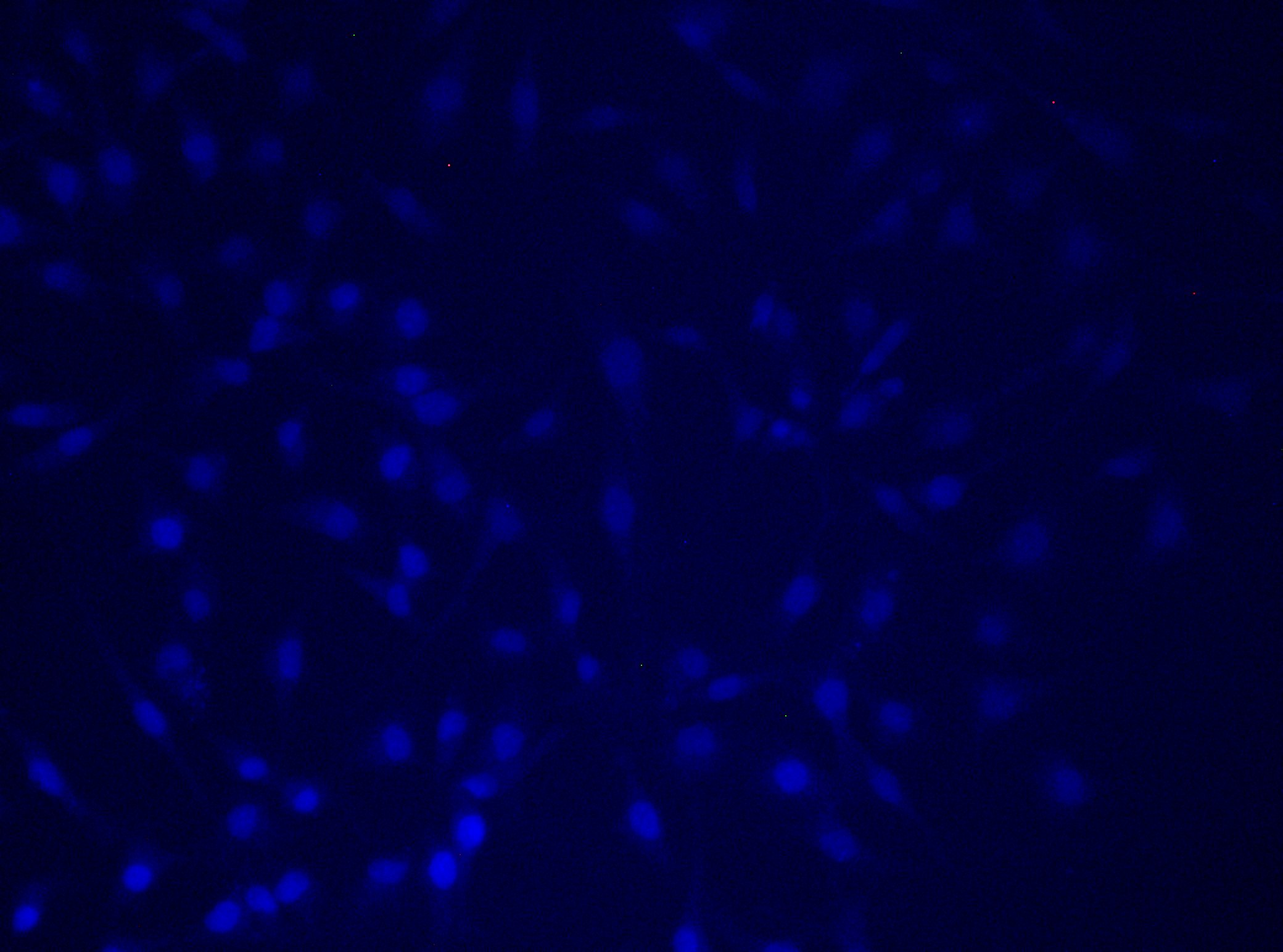

Supplement: Supplementary file 6 — Source data Fig. 2 [file 44318_2026_766_MOESM6_ESM.zip › Figure2/Fig2A/PBS/dapi.jpg]

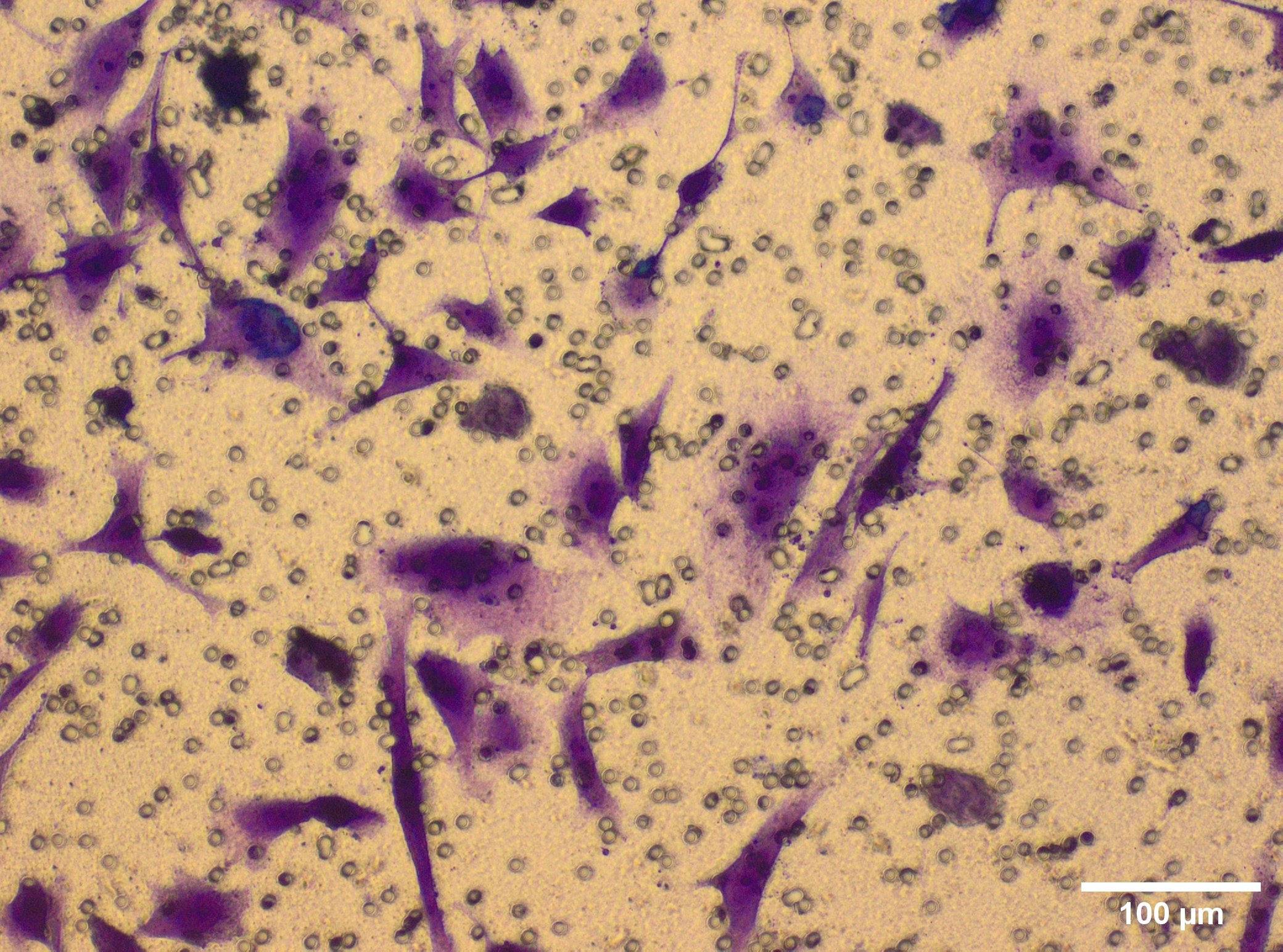

Supplement: Supplementary file 7 — Source data Fig. 3 [file 44318_2026_766_MOESM7_ESM.zip › Figure3/Fig3A/Invasion/vector.jpg]

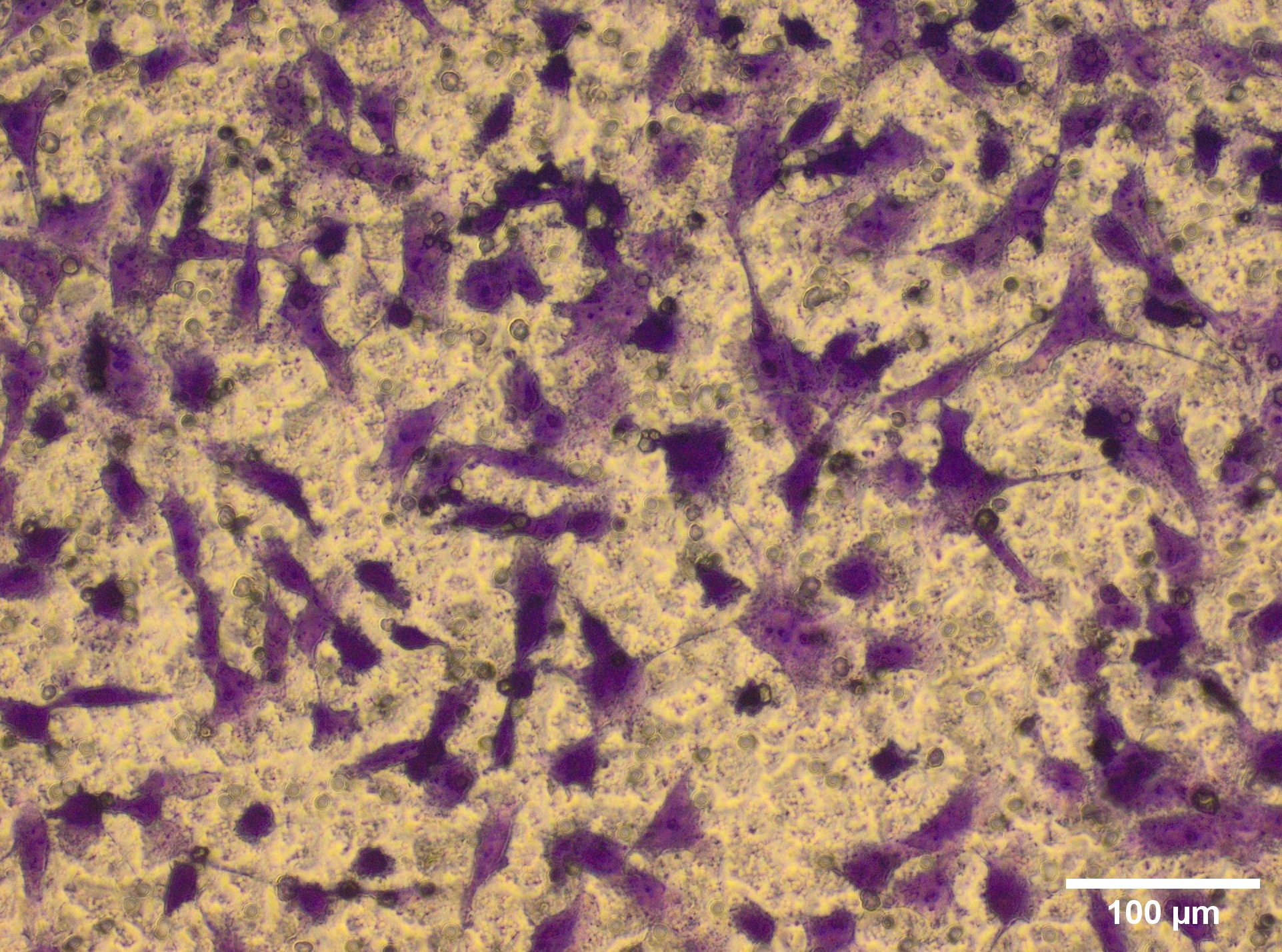

Supplement: Supplementary file 7 — Source data Fig. 3 [file 44318_2026_766_MOESM7_ESM.zip › Figure3/Fig3A/Invasion/EFEMP1.jpg]

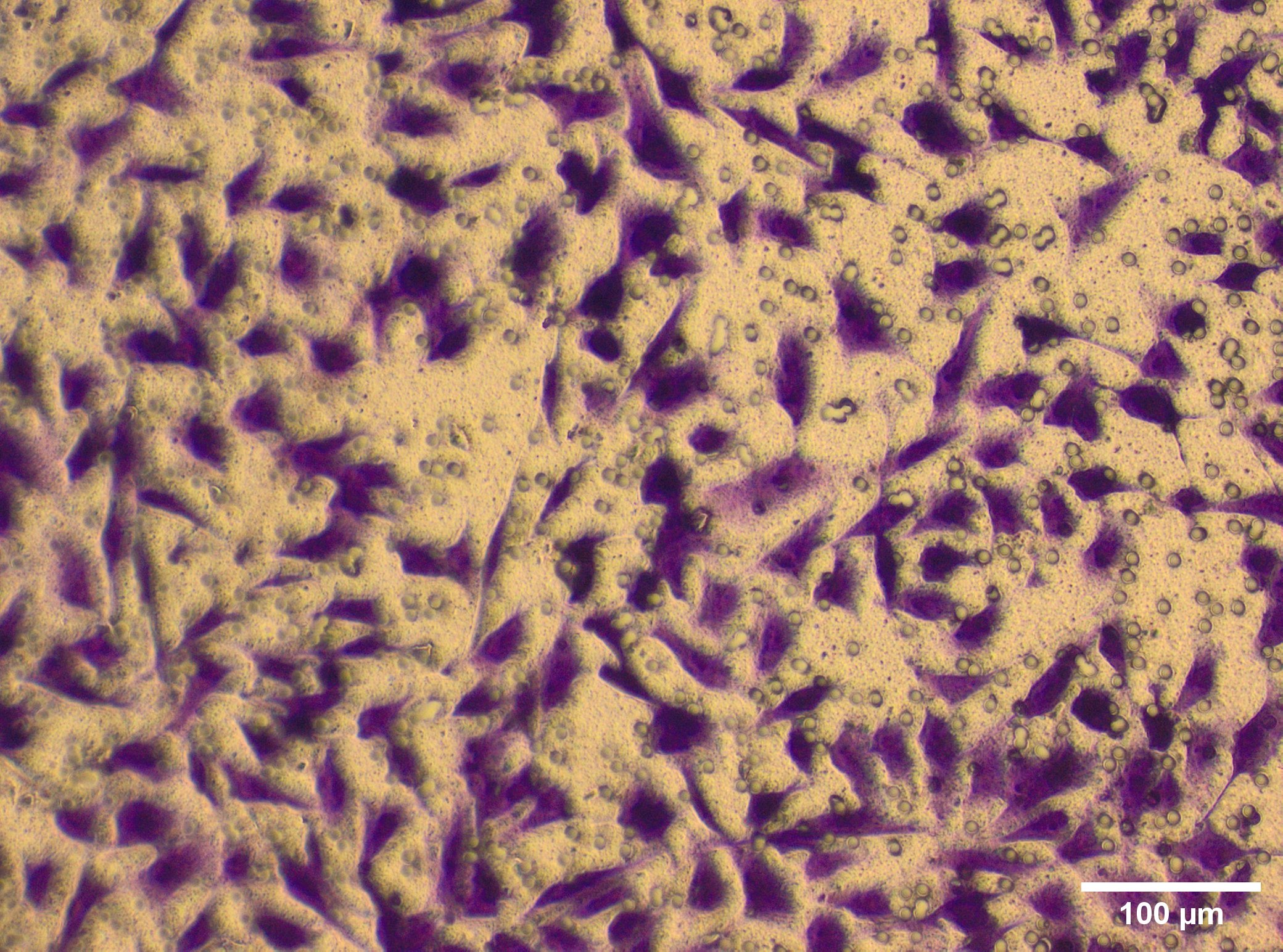

Supplement: Supplementary file 7 — Source data Fig. 3 [file 44318_2026_766_MOESM7_ESM.zip › Figure3/Fig3A/migration/vector.jpg]

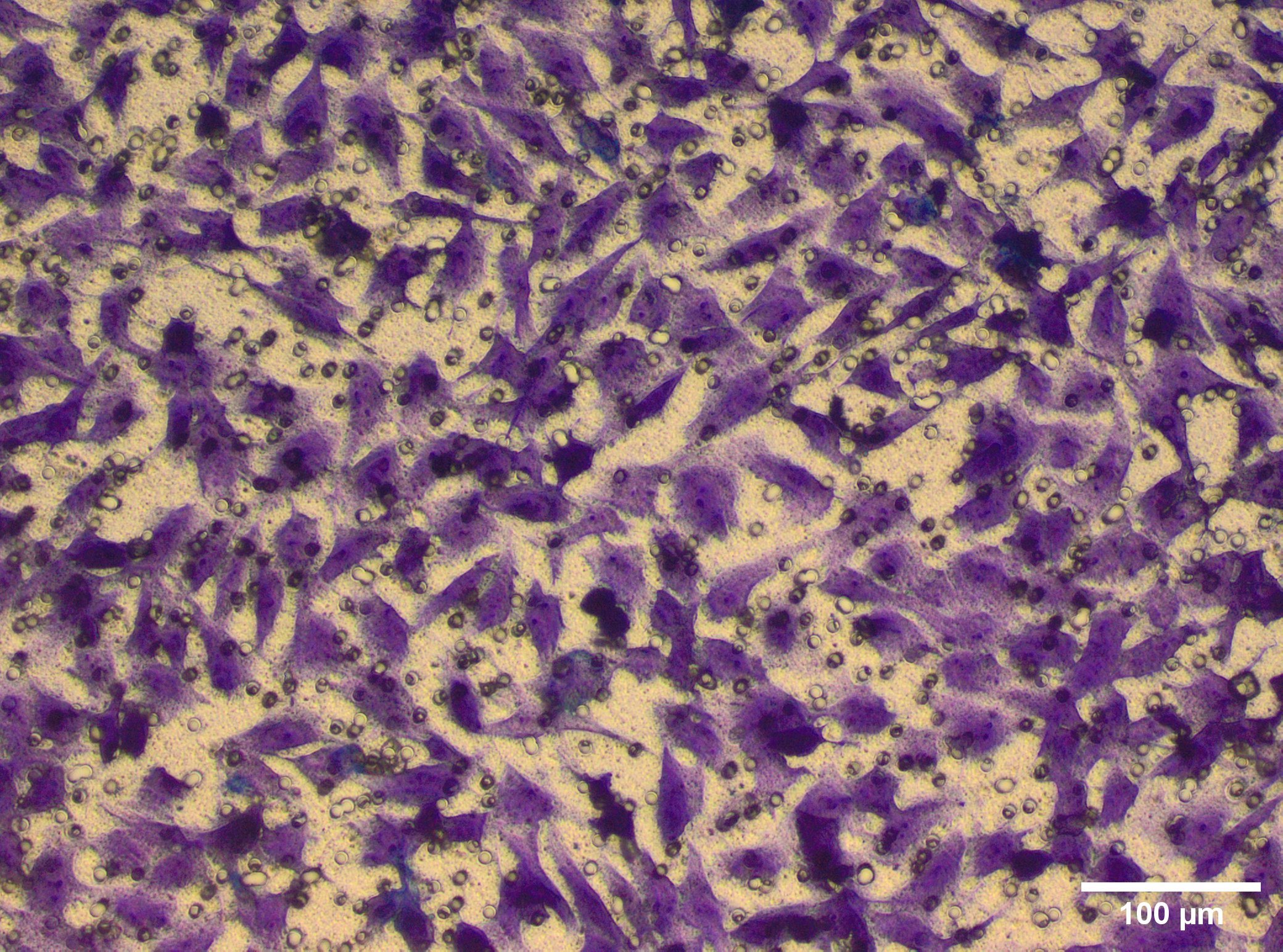

Supplement: Supplementary file 7 — Source data Fig. 3 [file 44318_2026_766_MOESM7_ESM.zip › Figure3/Fig3A/migration/EFEMP1.jpg]

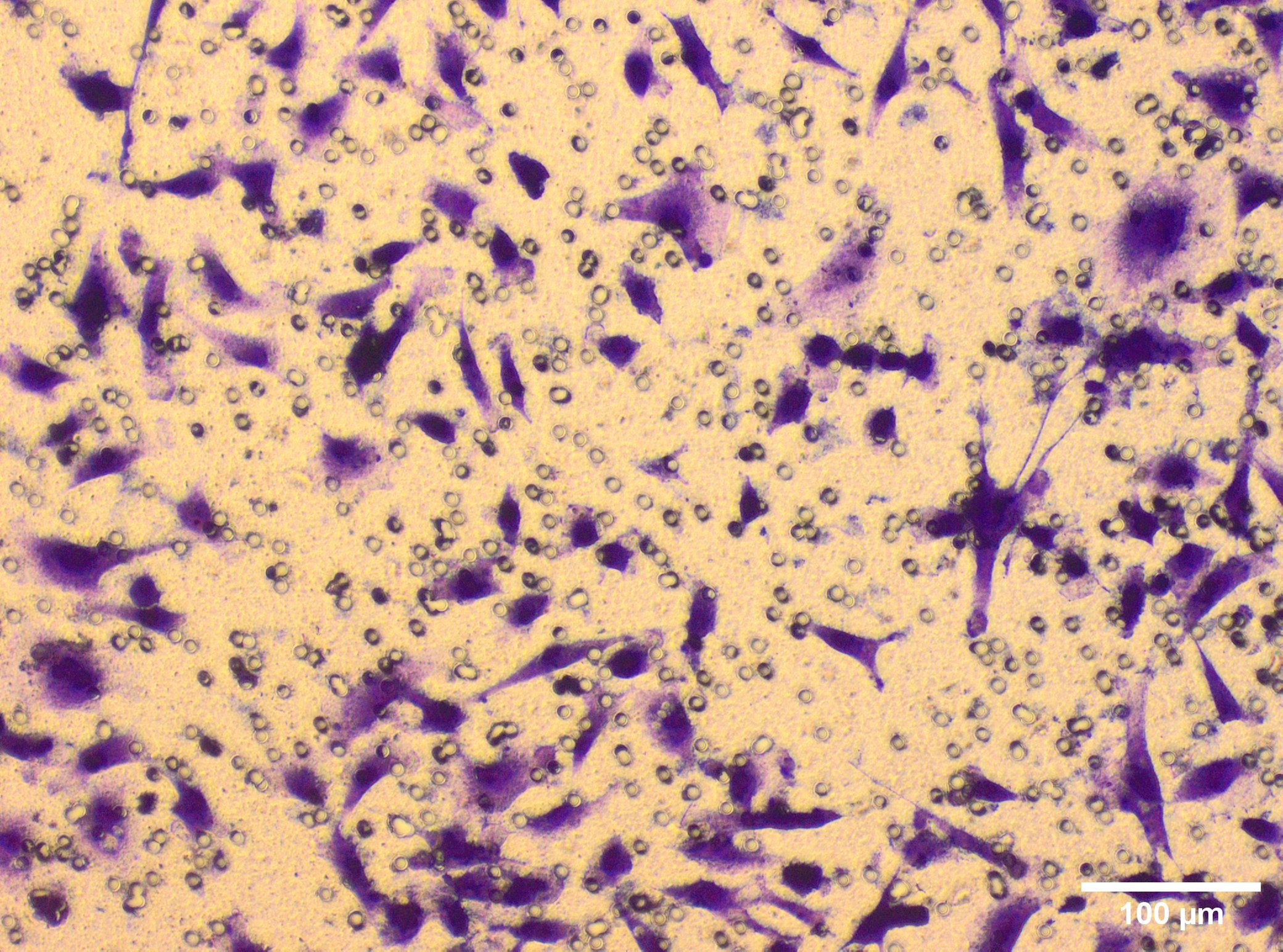

Supplement: Supplementary file 7 — Source data Fig. 3 [file 44318_2026_766_MOESM7_ESM.zip › Figure3/Fig3C/Invasion/shrna no.jpg]

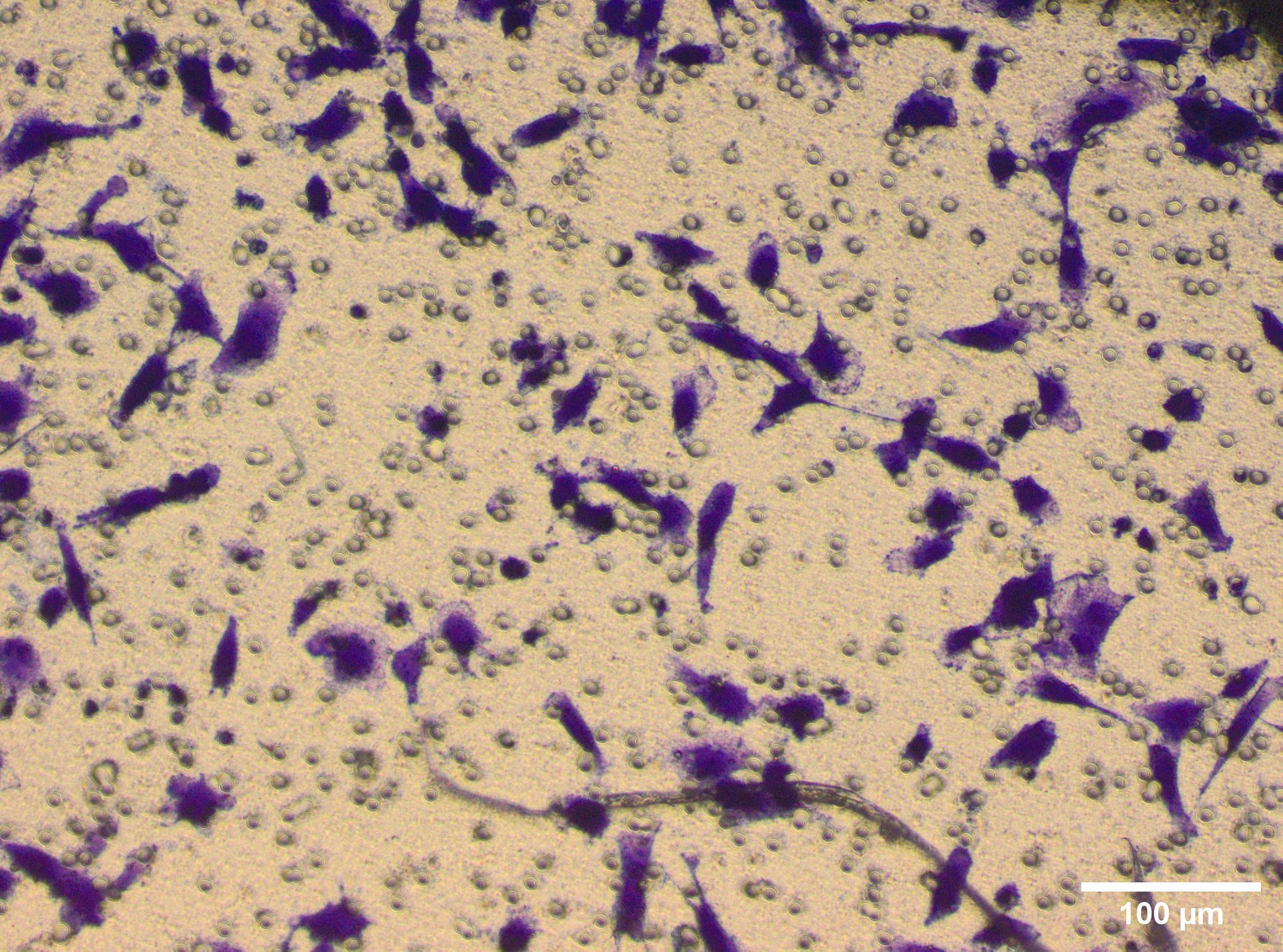

Supplement: Supplementary file 7 — Source data Fig. 3 [file 44318_2026_766_MOESM7_ESM.zip › Figure3/Fig3C/Invasion/shrna dox.jpg]

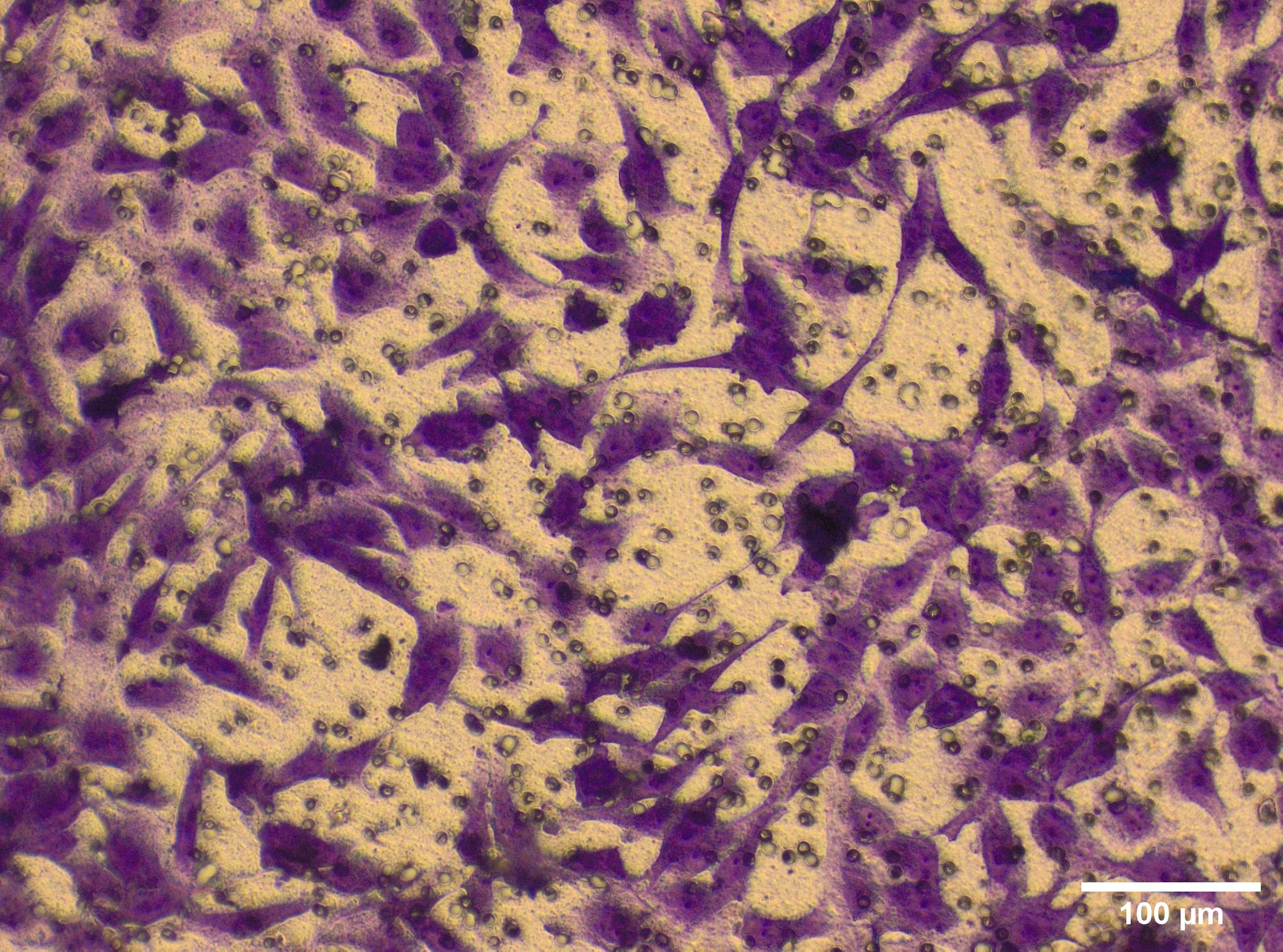

Supplement: Supplementary file 7 — Source data Fig. 3 [file 44318_2026_766_MOESM7_ESM.zip › Figure3/Fig3C/migration/shrna no.jpg]

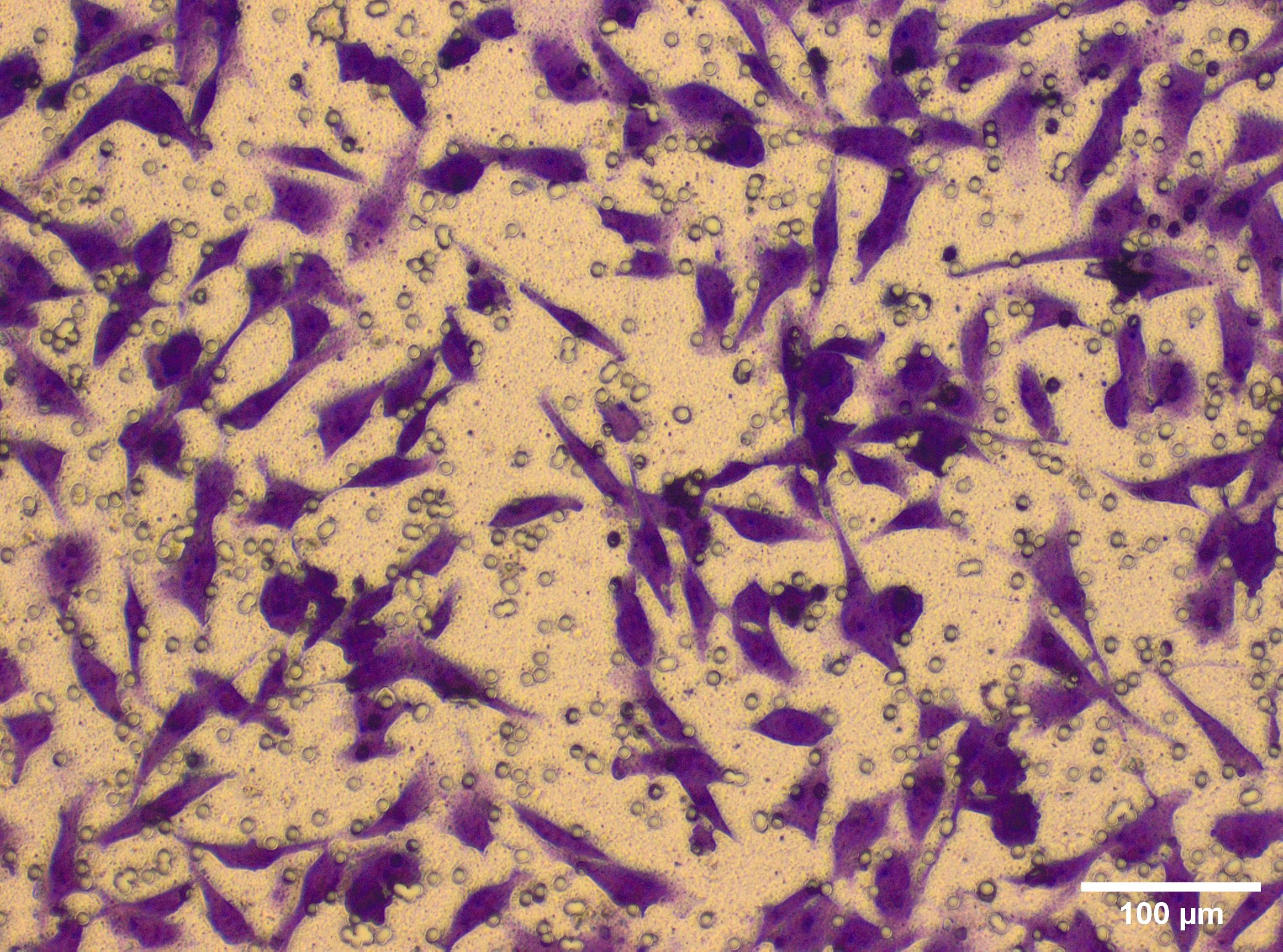

Supplement: Supplementary file 7 — Source data Fig. 3 [file 44318_2026_766_MOESM7_ESM.zip › Figure3/Fig3C/migration/shrna dox.jpg]

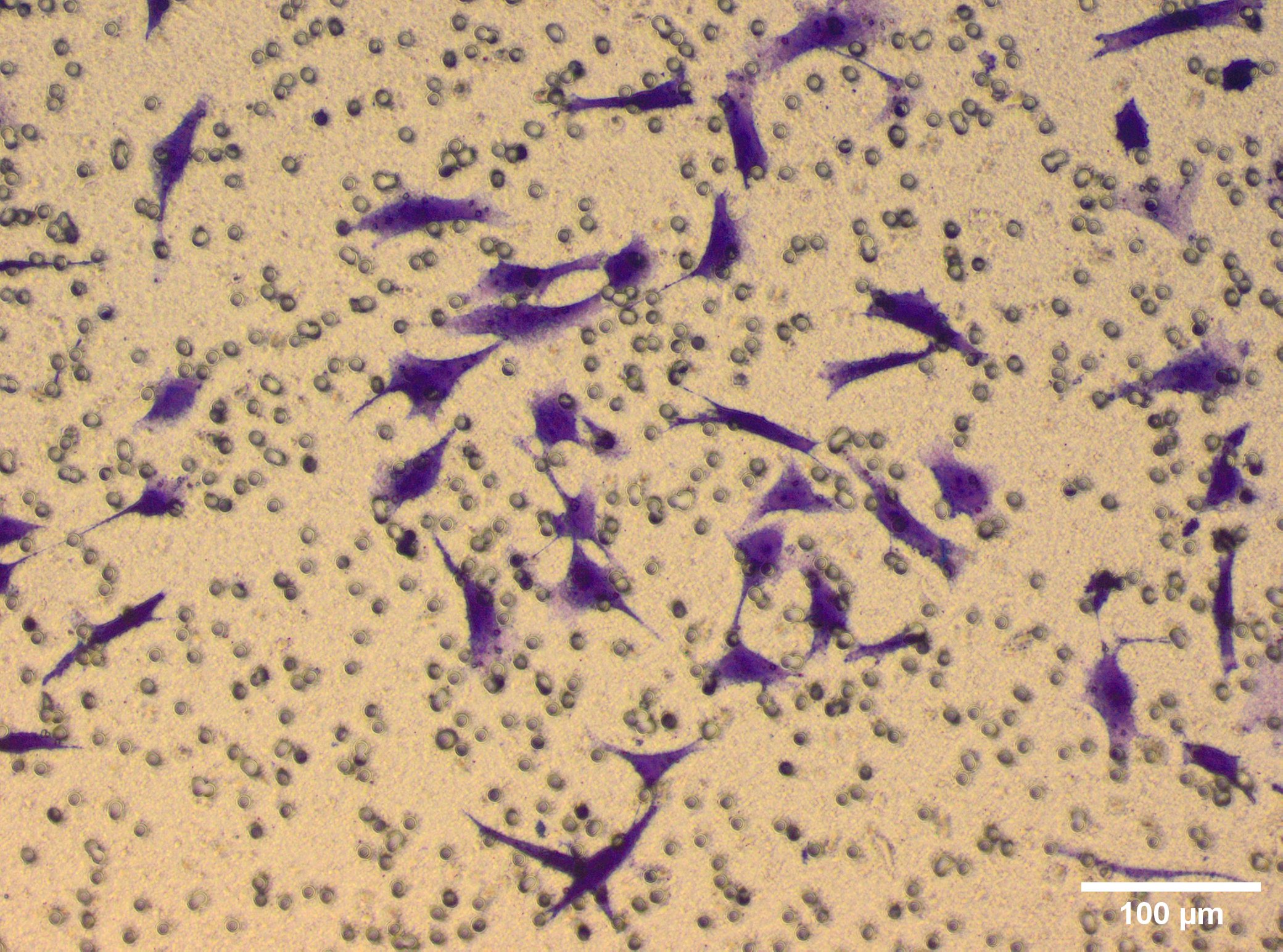

Supplement: Supplementary file 7 — Source data Fig. 3 [file 44318_2026_766_MOESM7_ESM.zip › Figure3/Fig3E/Invasion/shrna dox dmso.jpg]

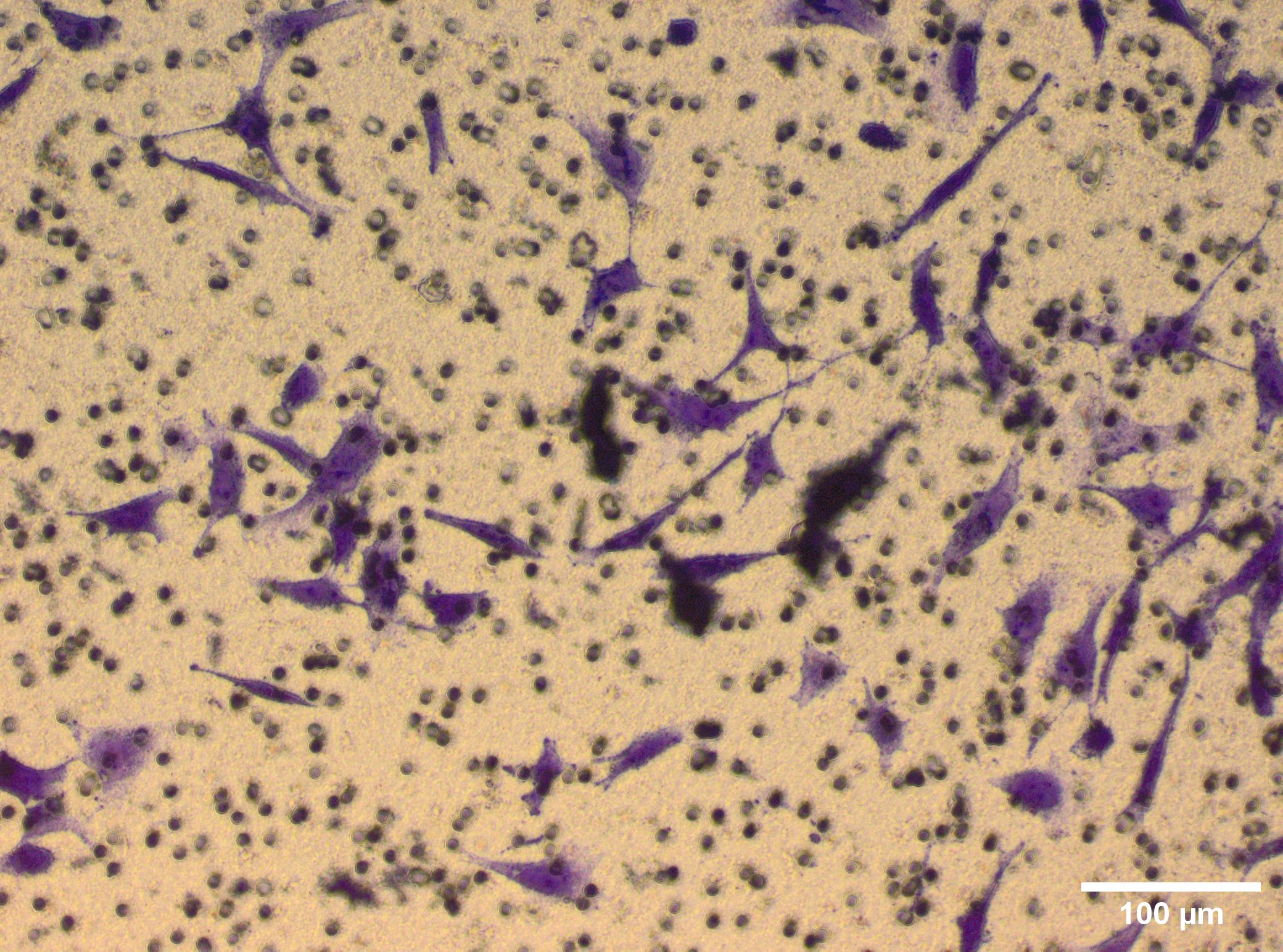

Supplement: Supplementary file 7 — Source data Fig. 3 [file 44318_2026_766_MOESM7_ESM.zip › Figure3/Fig3E/Invasion/shrna dox rev.jpg]

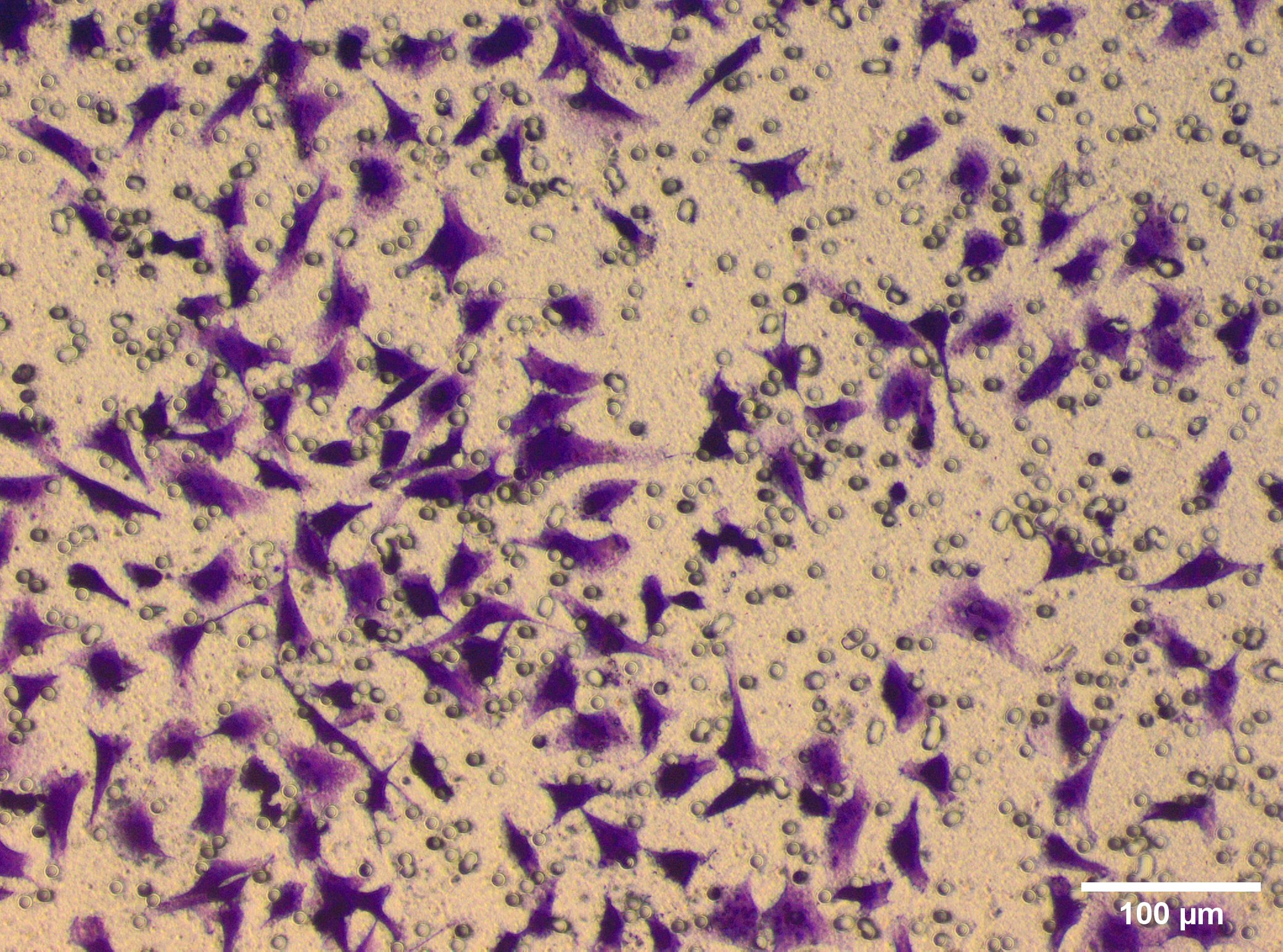

Supplement: Supplementary file 7 — Source data Fig. 3 [file 44318_2026_766_MOESM7_ESM.zip › Figure3/Fig3E/Invasion/rev.jpg]

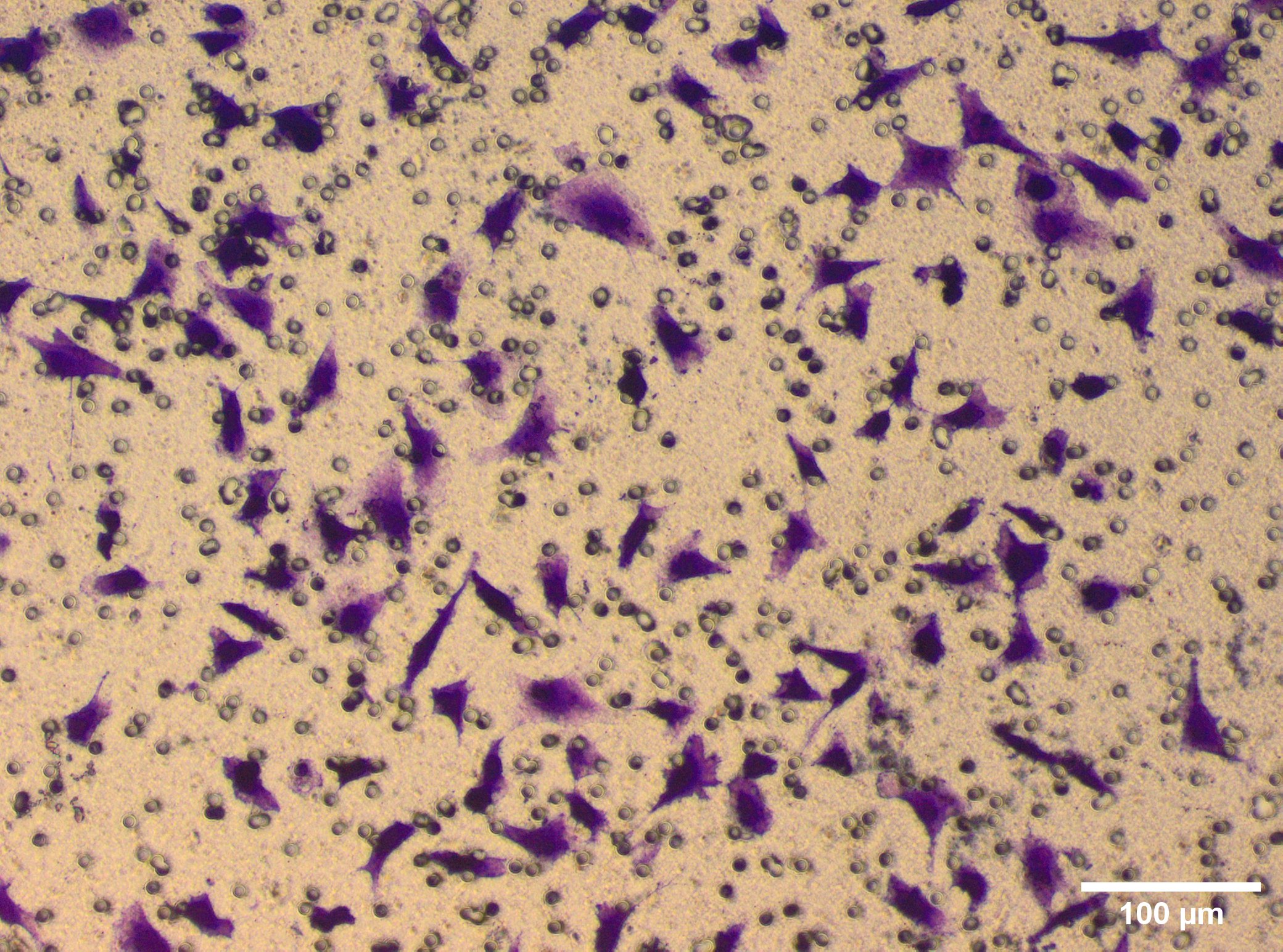

Supplement: Supplementary file 7 — Source data Fig. 3 [file 44318_2026_766_MOESM7_ESM.zip › Figure3/Fig3E/Invasion/dmso.jpg]

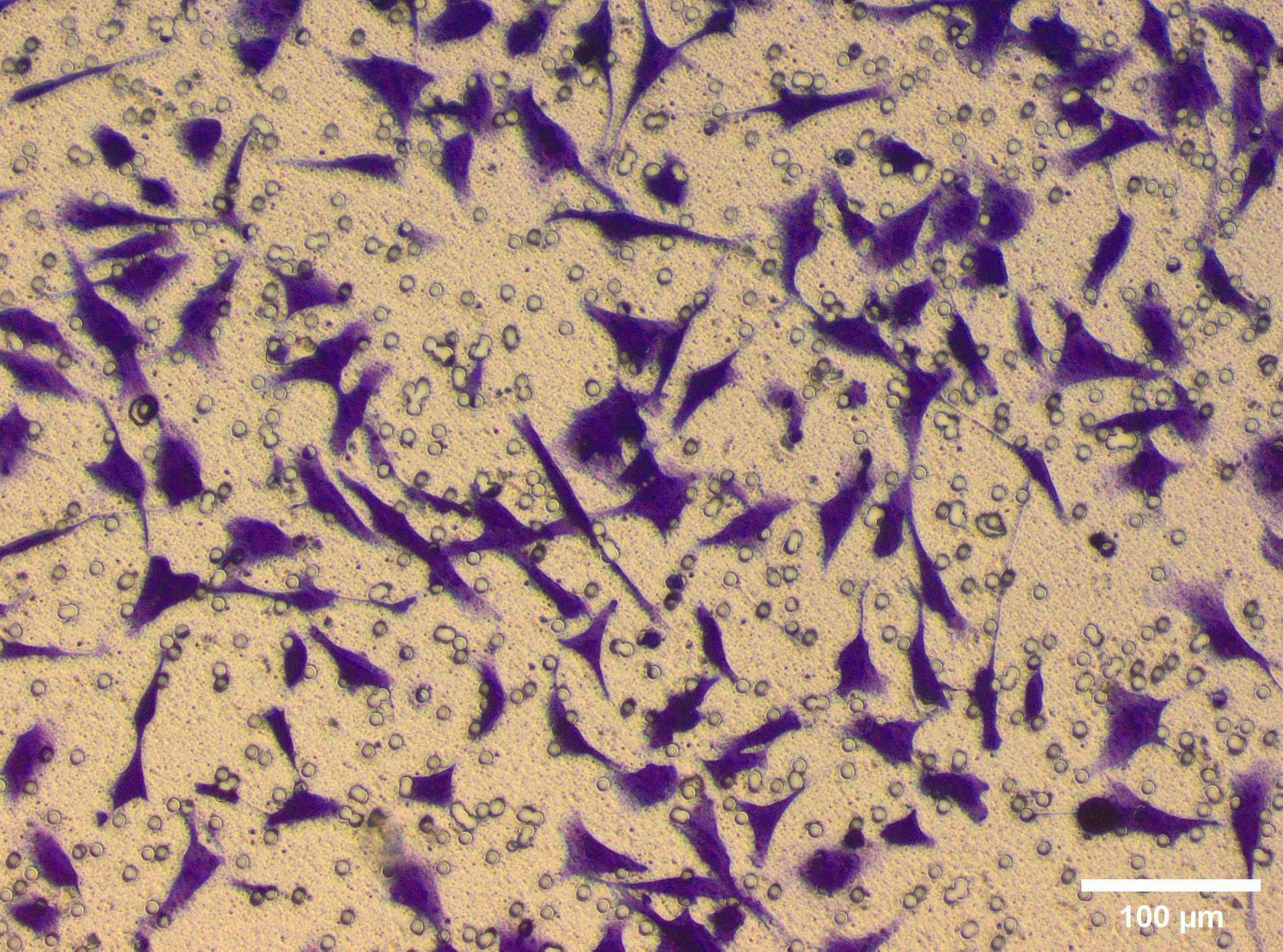

Supplement: Supplementary file 7 — Source data Fig. 3 [file 44318_2026_766_MOESM7_ESM.zip › Figure3/Fig3E/migration/shRNA dox dmso.jpg]

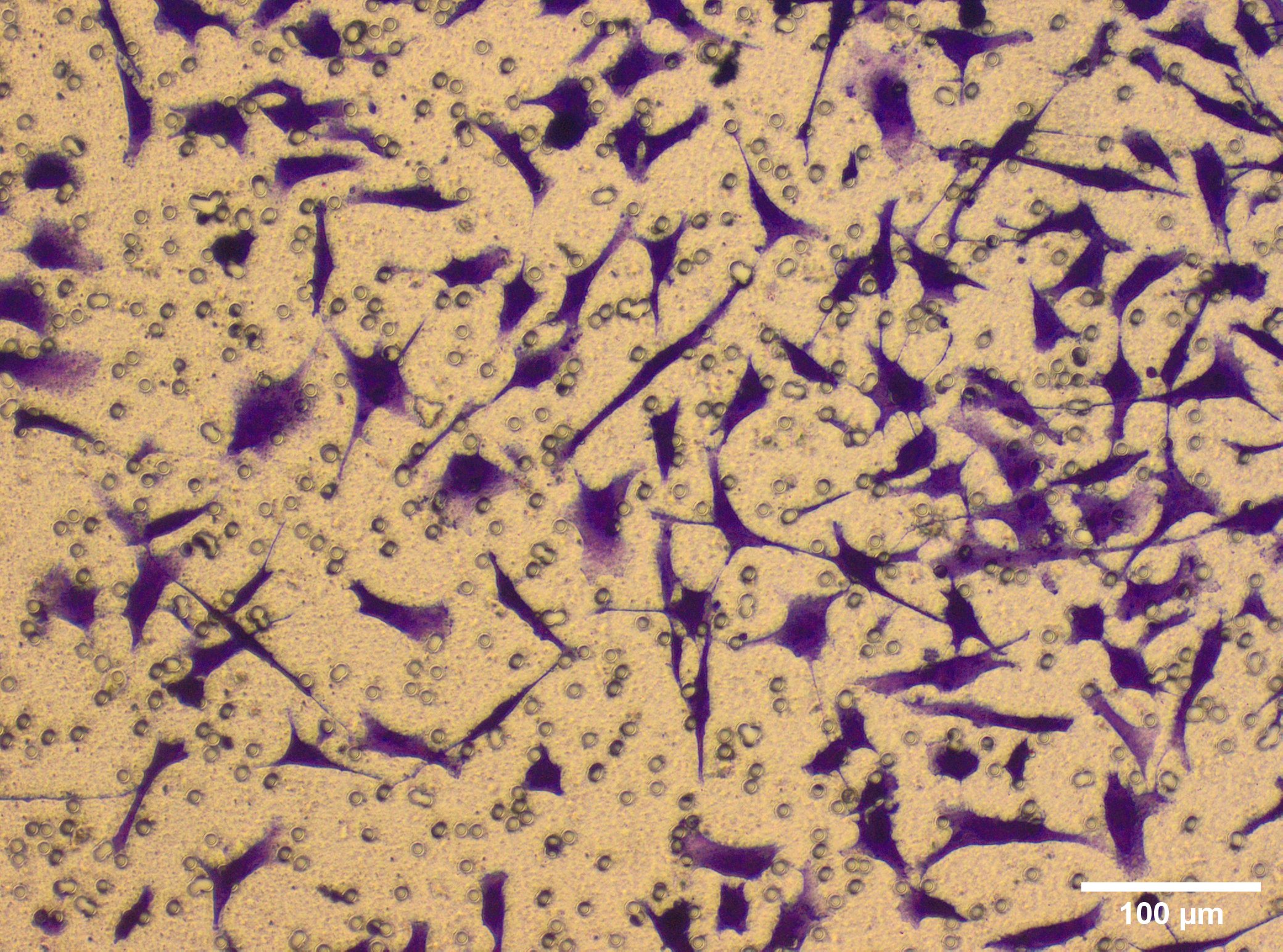

Supplement: Supplementary file 7 — Source data Fig. 3 [file 44318_2026_766_MOESM7_ESM.zip › Figure3/Fig3E/migration/shrna dox rev.jpg]

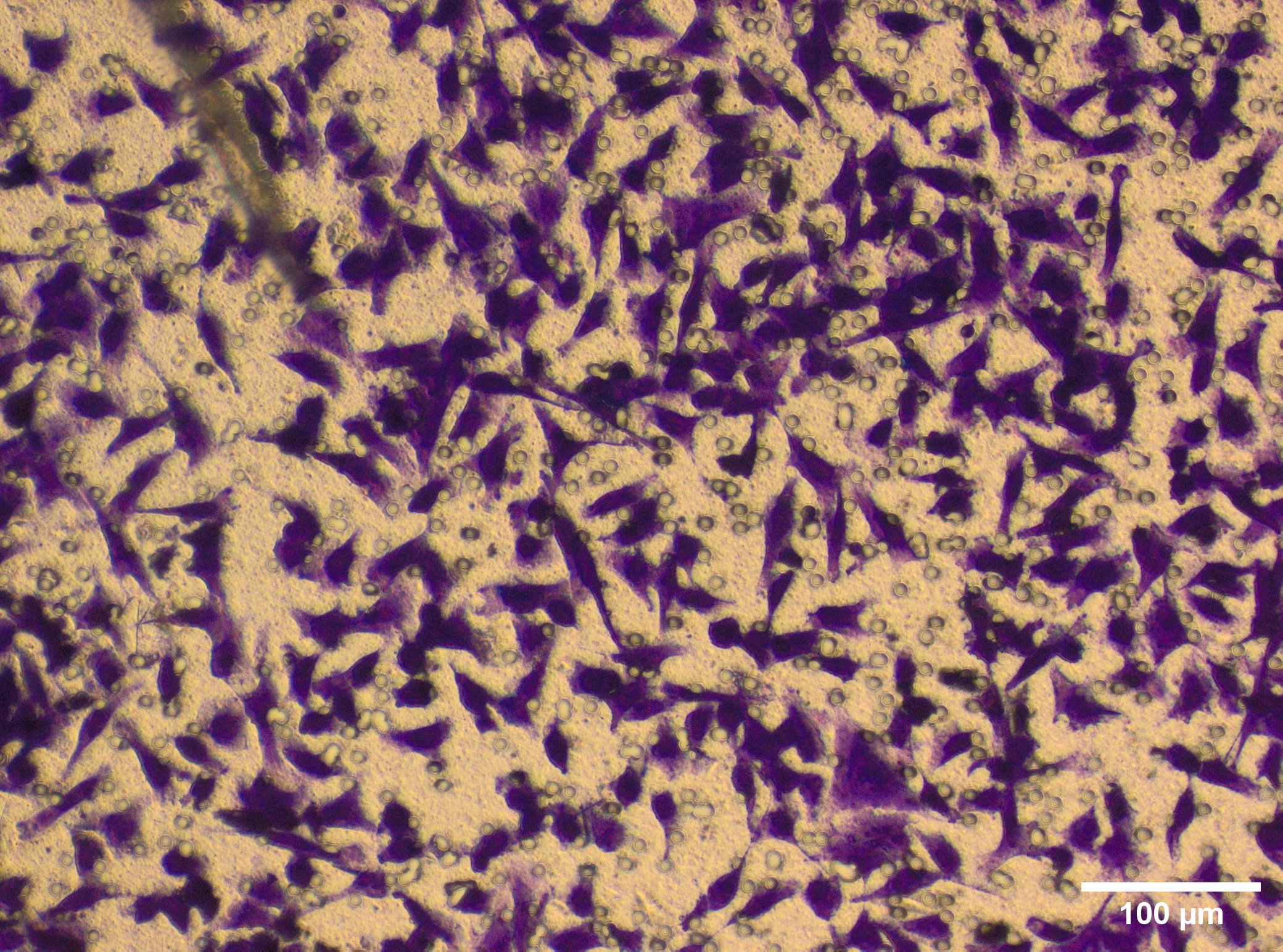

Supplement: Supplementary file 7 — Source data Fig. 3 [file 44318_2026_766_MOESM7_ESM.zip › Figure3/Fig3E/migration/rev.jpg]

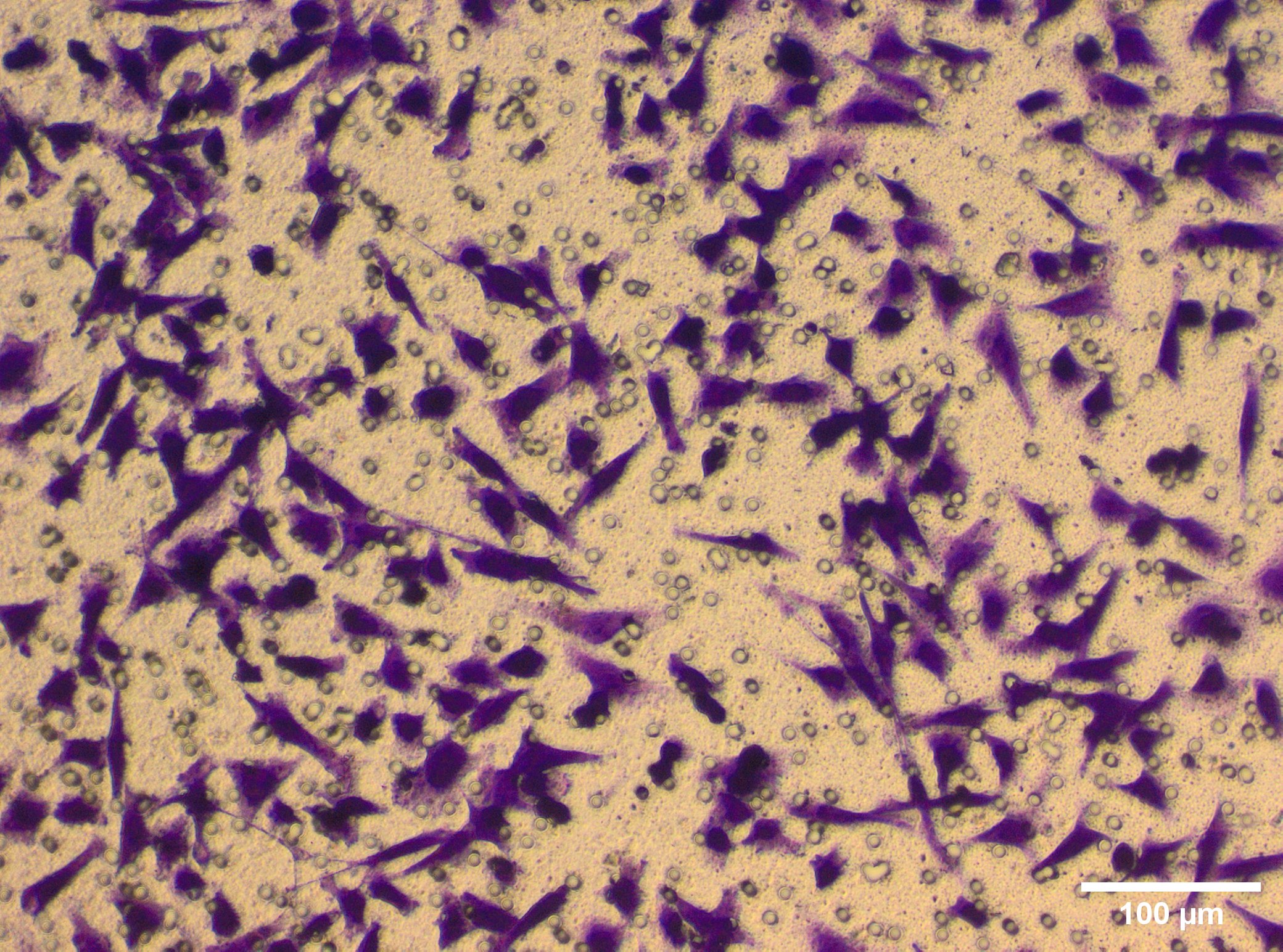

Supplement: Supplementary file 7 — Source data Fig. 3 [file 44318_2026_766_MOESM7_ESM.zip › Figure3/Fig3E/migration/dmso.jpg]

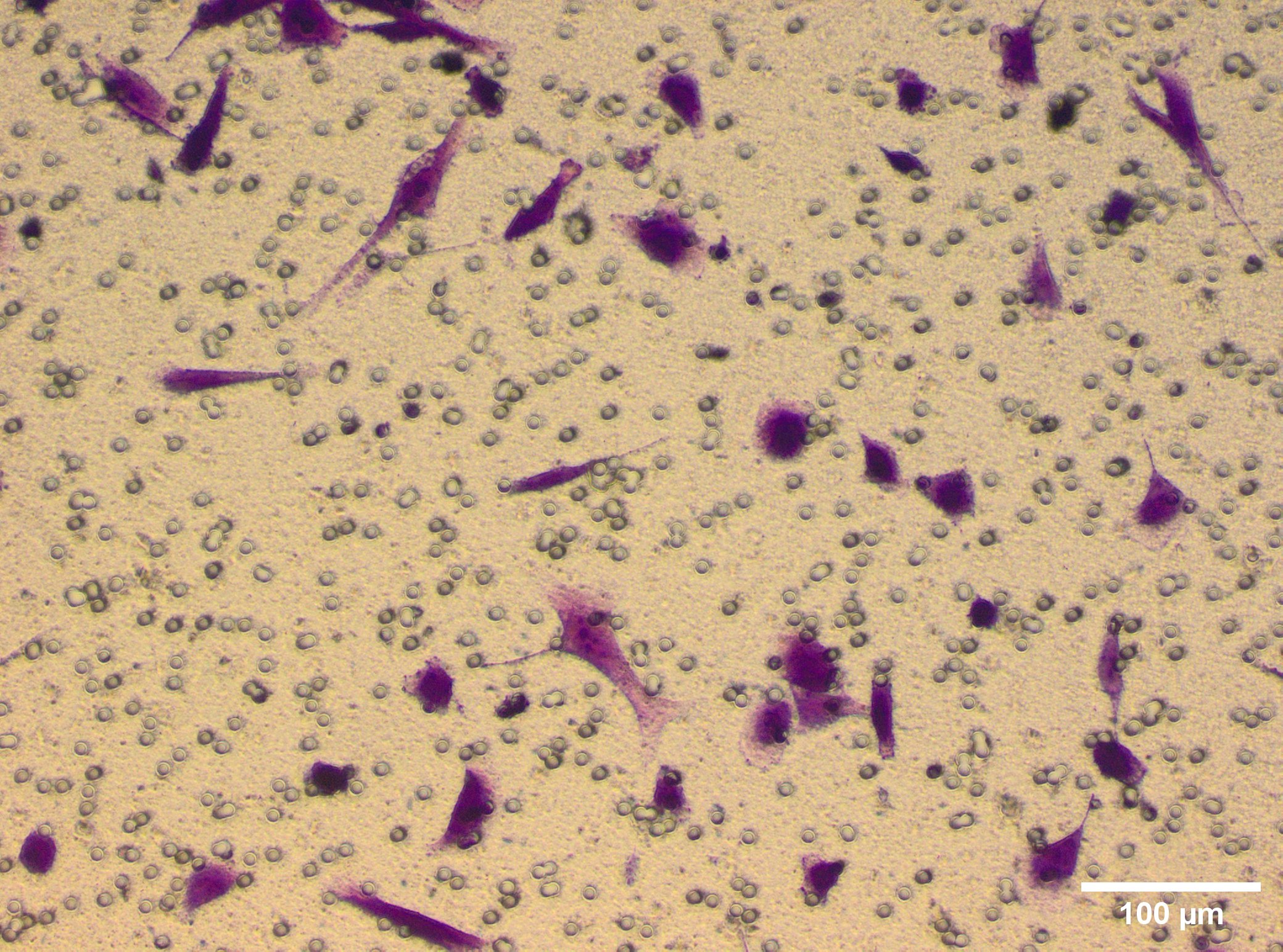

Supplement: Supplementary file 8 — Source data Fig. 4 [file 44318_2026_766_MOESM8_ESM.zip › Figure4/Fig4F/Invasion/stat1 vector.jpg]

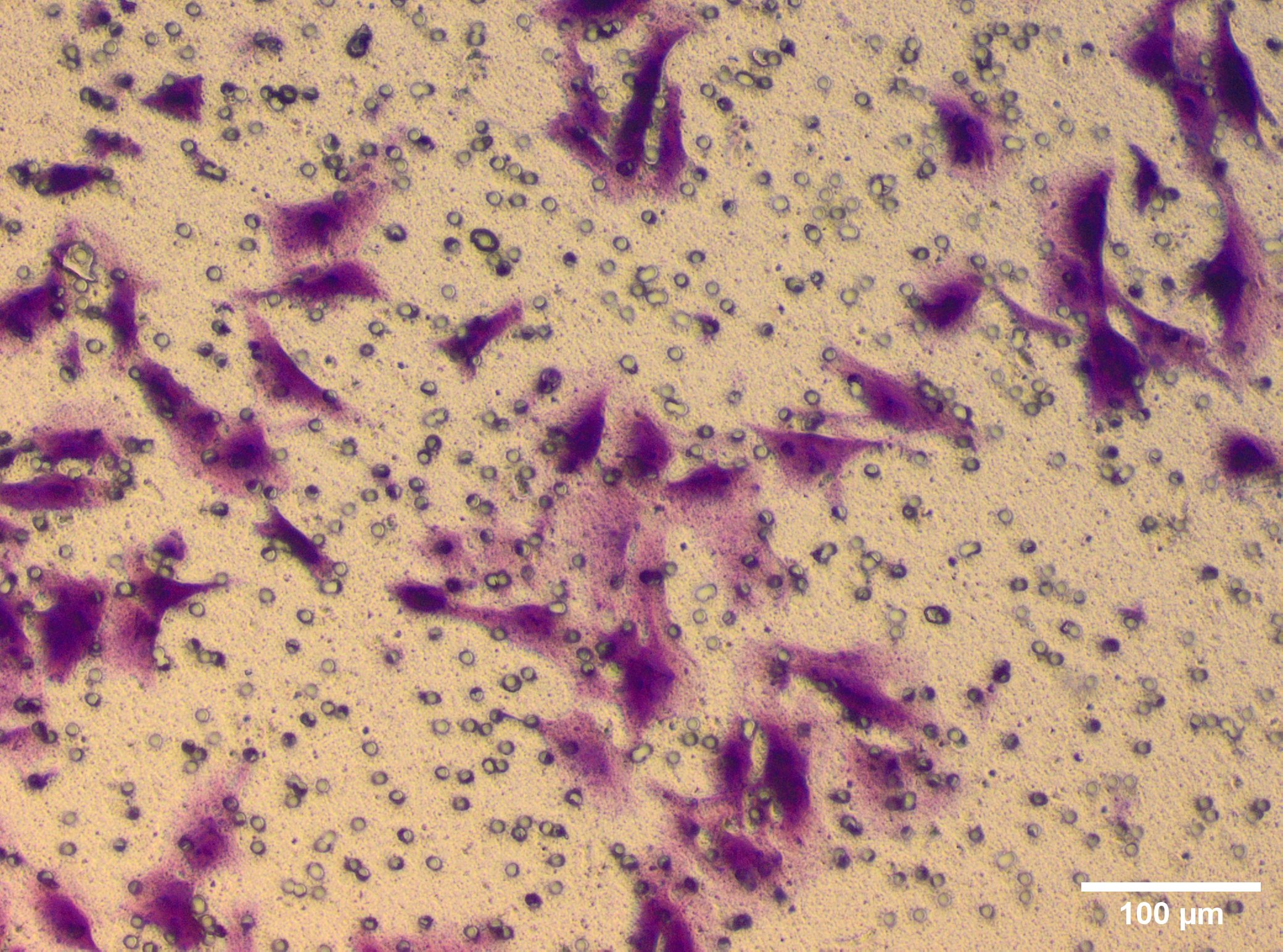

Supplement: Supplementary file 8 — Source data Fig. 4 [file 44318_2026_766_MOESM8_ESM.zip › Figure4/Fig4F/Invasion/stat1 efemp1.jpg]

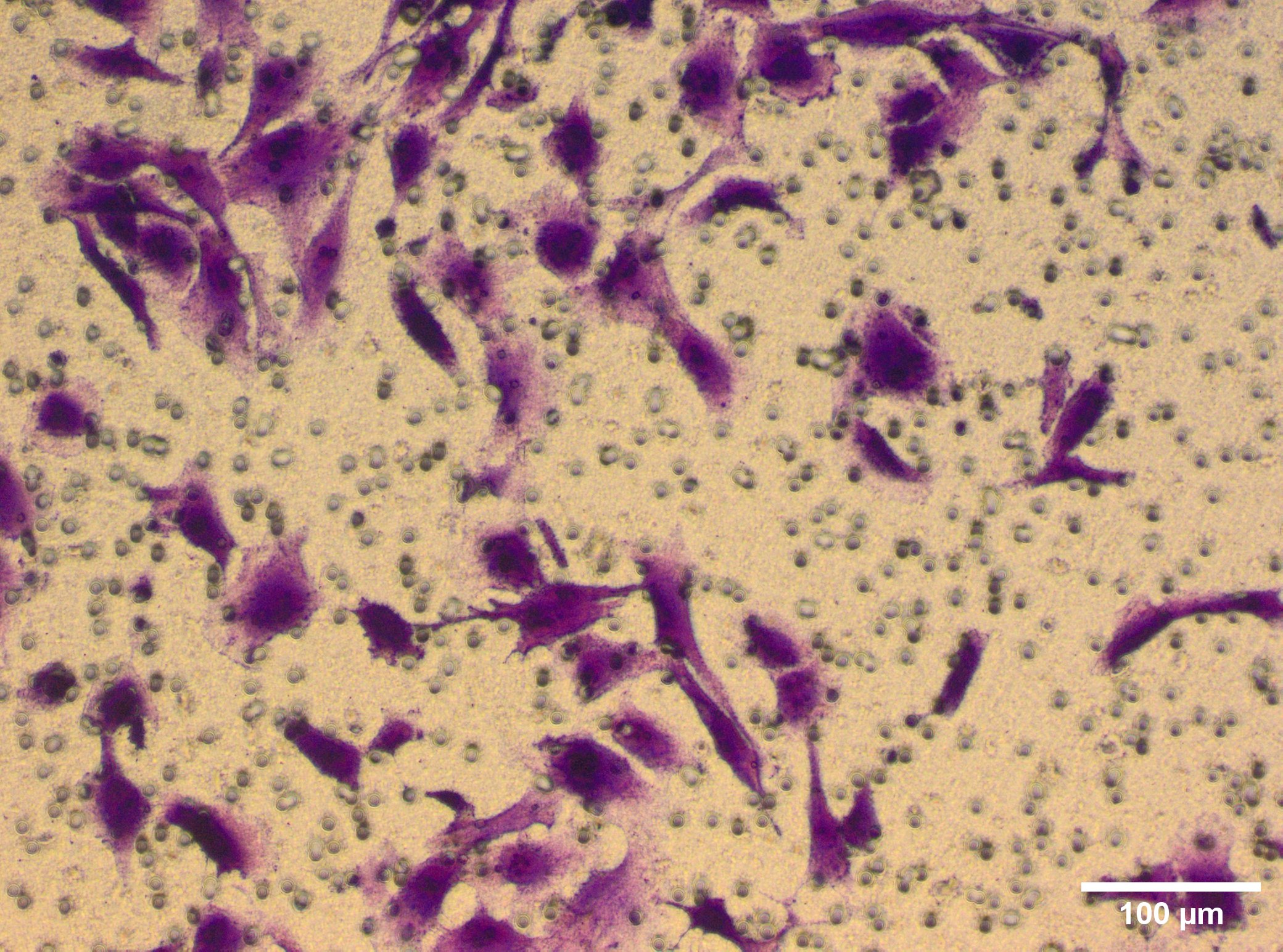

Supplement: Supplementary file 8 — Source data Fig. 4 [file 44318_2026_766_MOESM8_ESM.zip › Figure4/Fig4F/migration/sat1 vector.jpg]

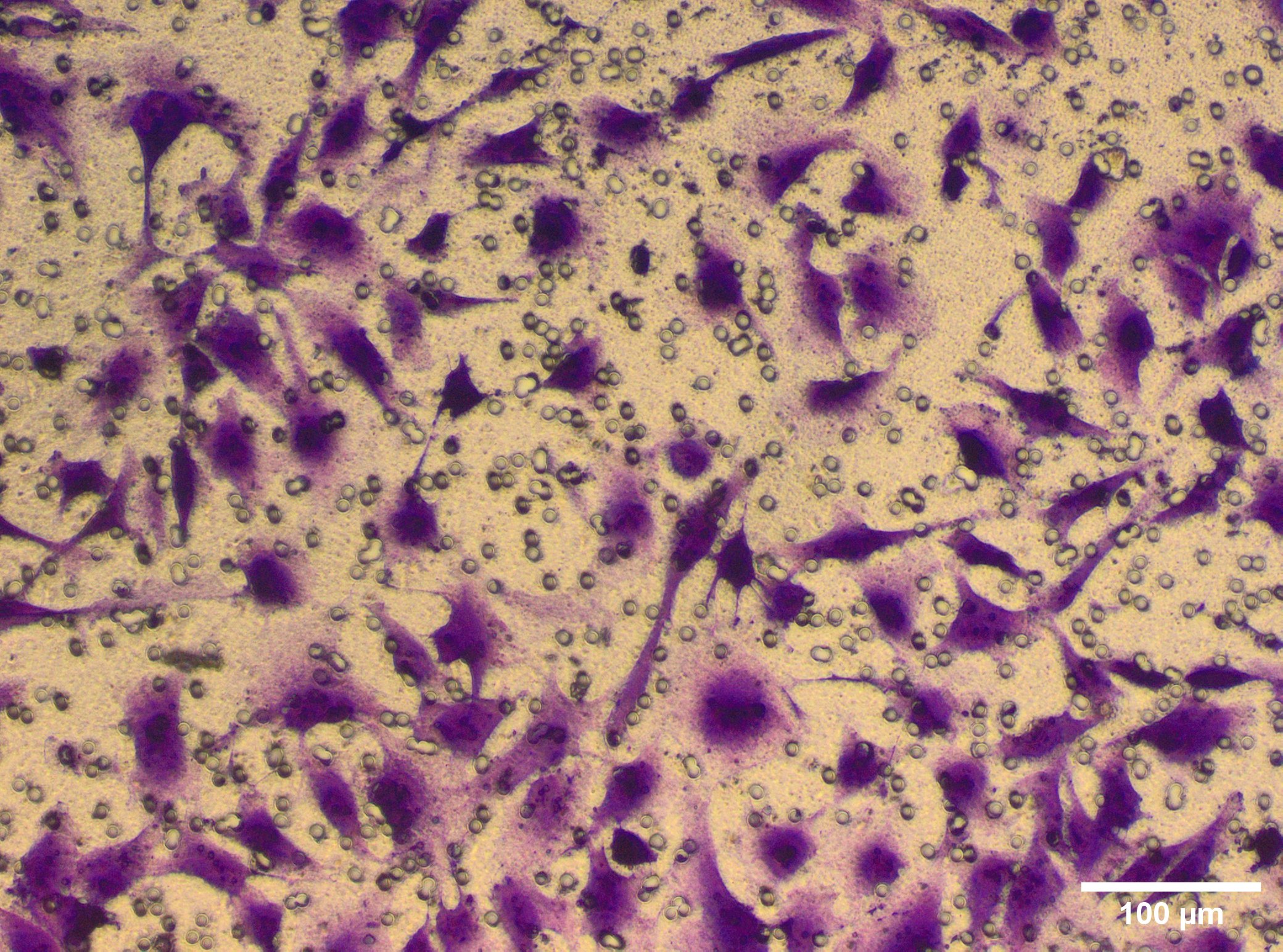

Supplement: Supplementary file 8 — Source data Fig. 4 [file 44318_2026_766_MOESM8_ESM.zip › Figure4/Fig4F/migration/Image_17464stat efe ev mig.jpg]

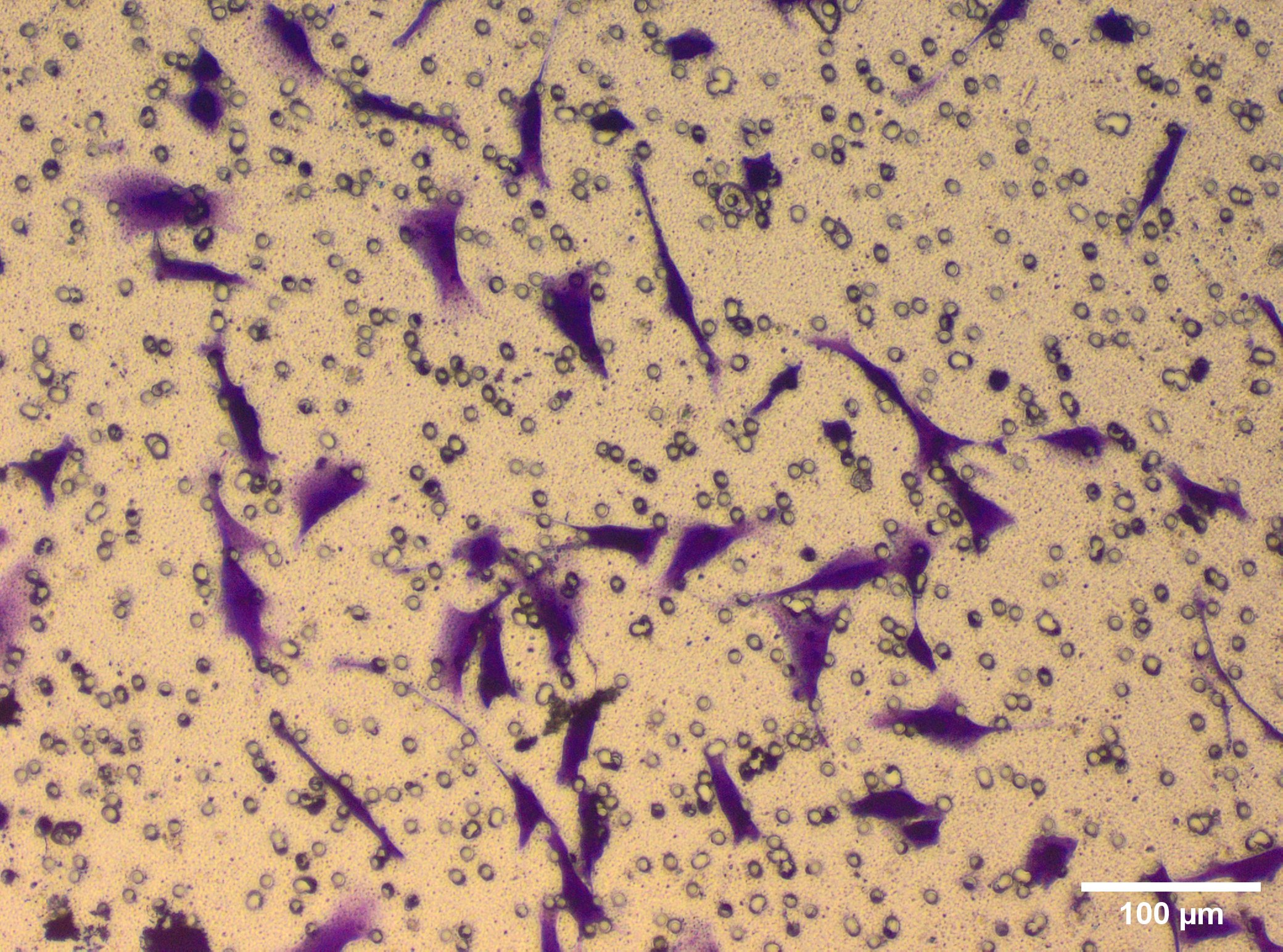

Supplement: Supplementary file 8 — Source data Fig. 4 [file 44318_2026_766_MOESM8_ESM.zip › Figure4/Fig4D/Invasion/stat1 rev.jpg]

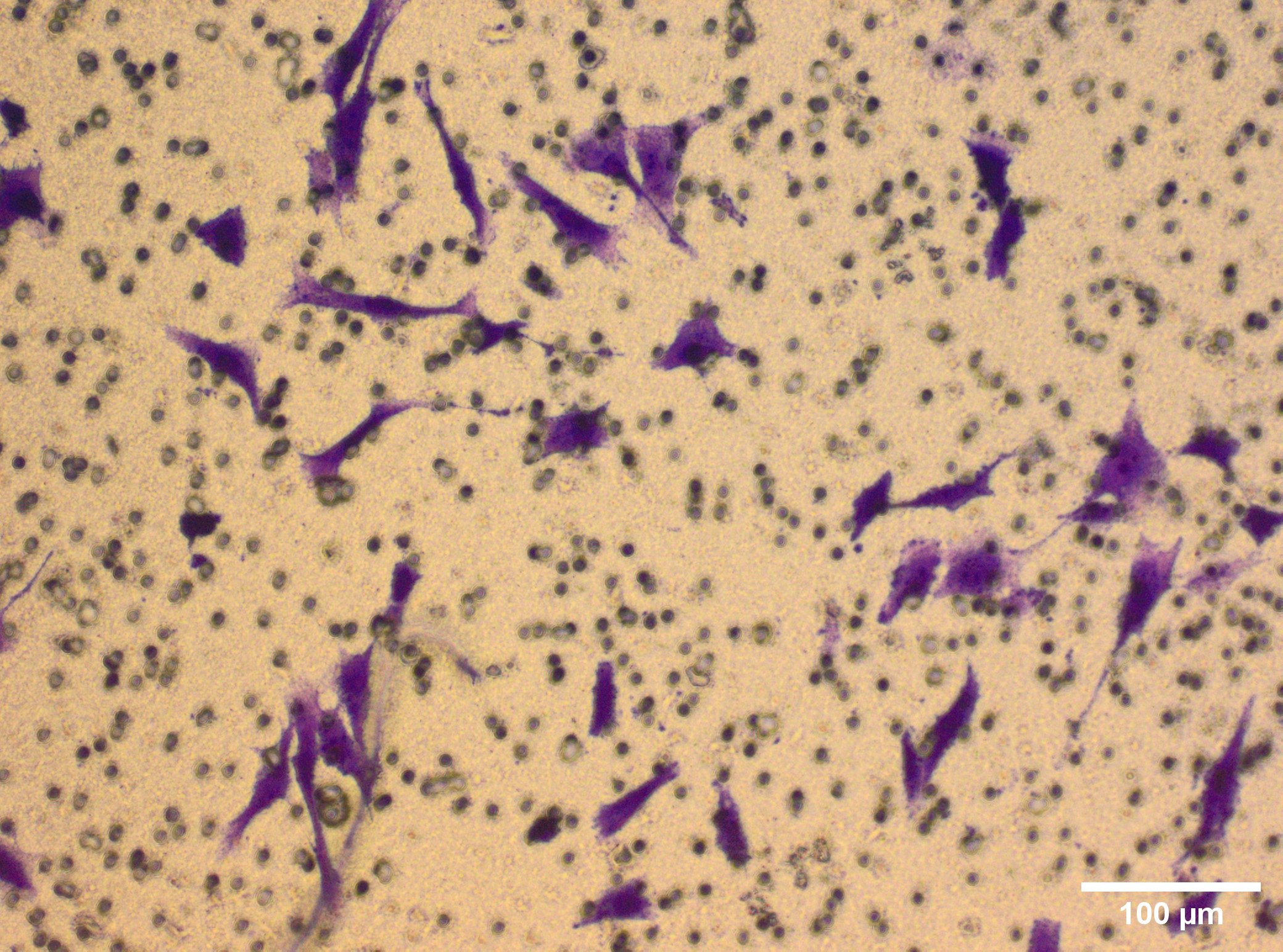

Supplement: Supplementary file 8 — Source data Fig. 4 [file 44318_2026_766_MOESM8_ESM.zip › Figure4/Fig4D/Invasion/stat1 dmso.jpg]

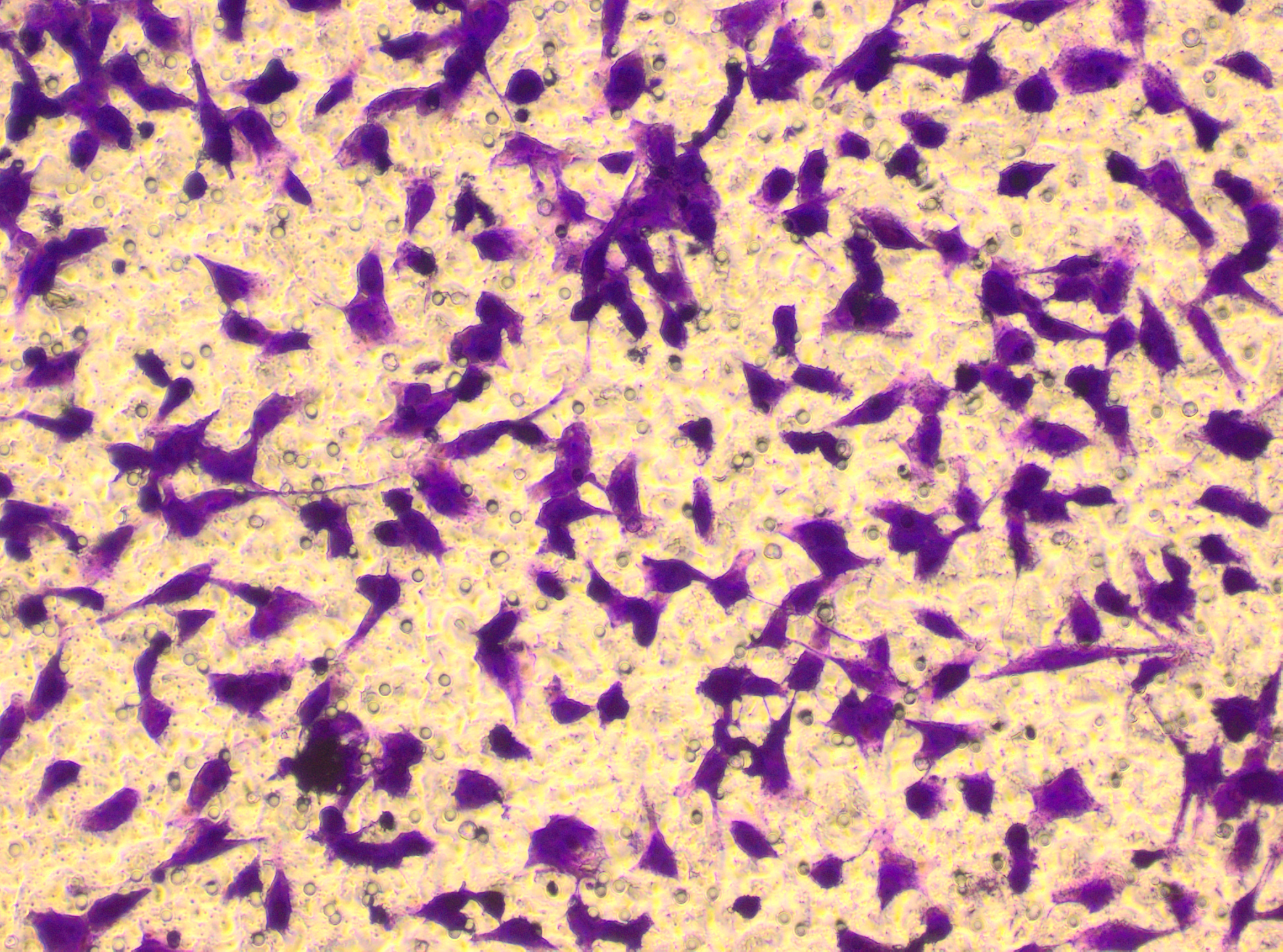

Supplement: Supplementary file 8 — Source data Fig. 4 [file 44318_2026_766_MOESM8_ESM.zip › Figure4/Fig4D/Invasion/rev.tif]

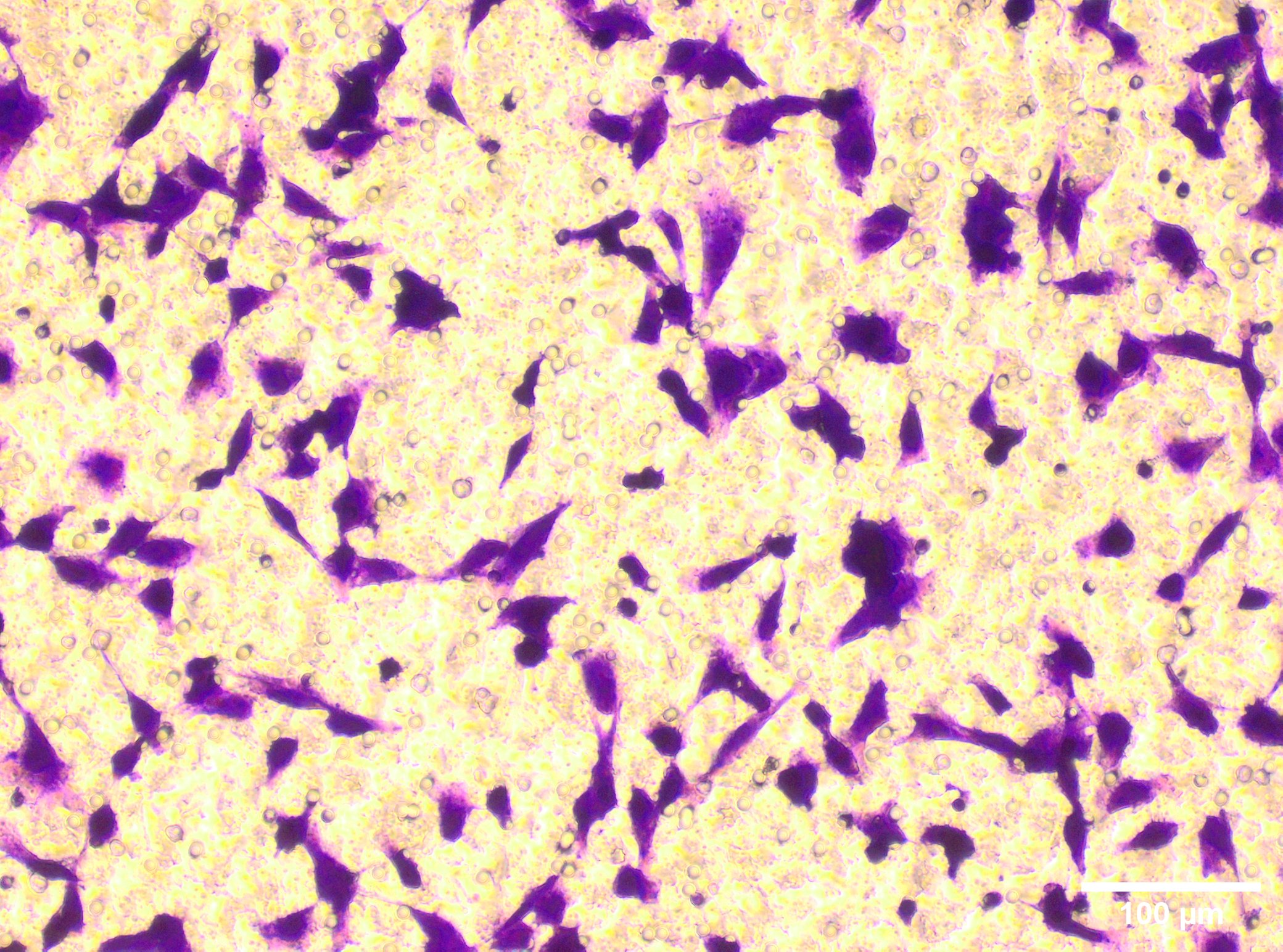

Supplement: Supplementary file 8 — Source data Fig. 4 [file 44318_2026_766_MOESM8_ESM.zip › Figure4/Fig4D/Invasion/dmso.jpg]

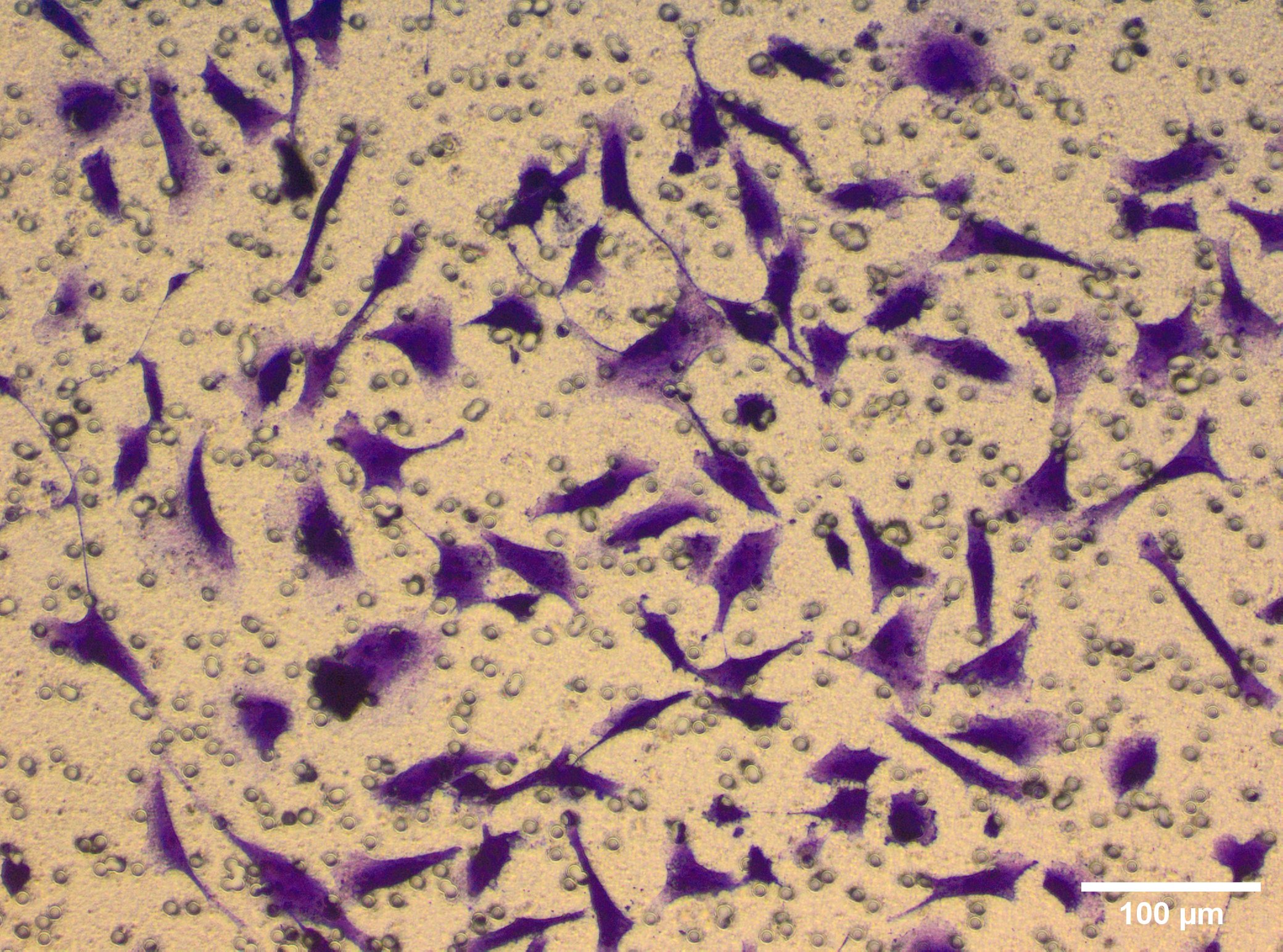

Supplement: Supplementary file 8 — Source data Fig. 4 [file 44318_2026_766_MOESM8_ESM.zip › Figure4/Fig4D/migration/stat1 dmso.jpg]

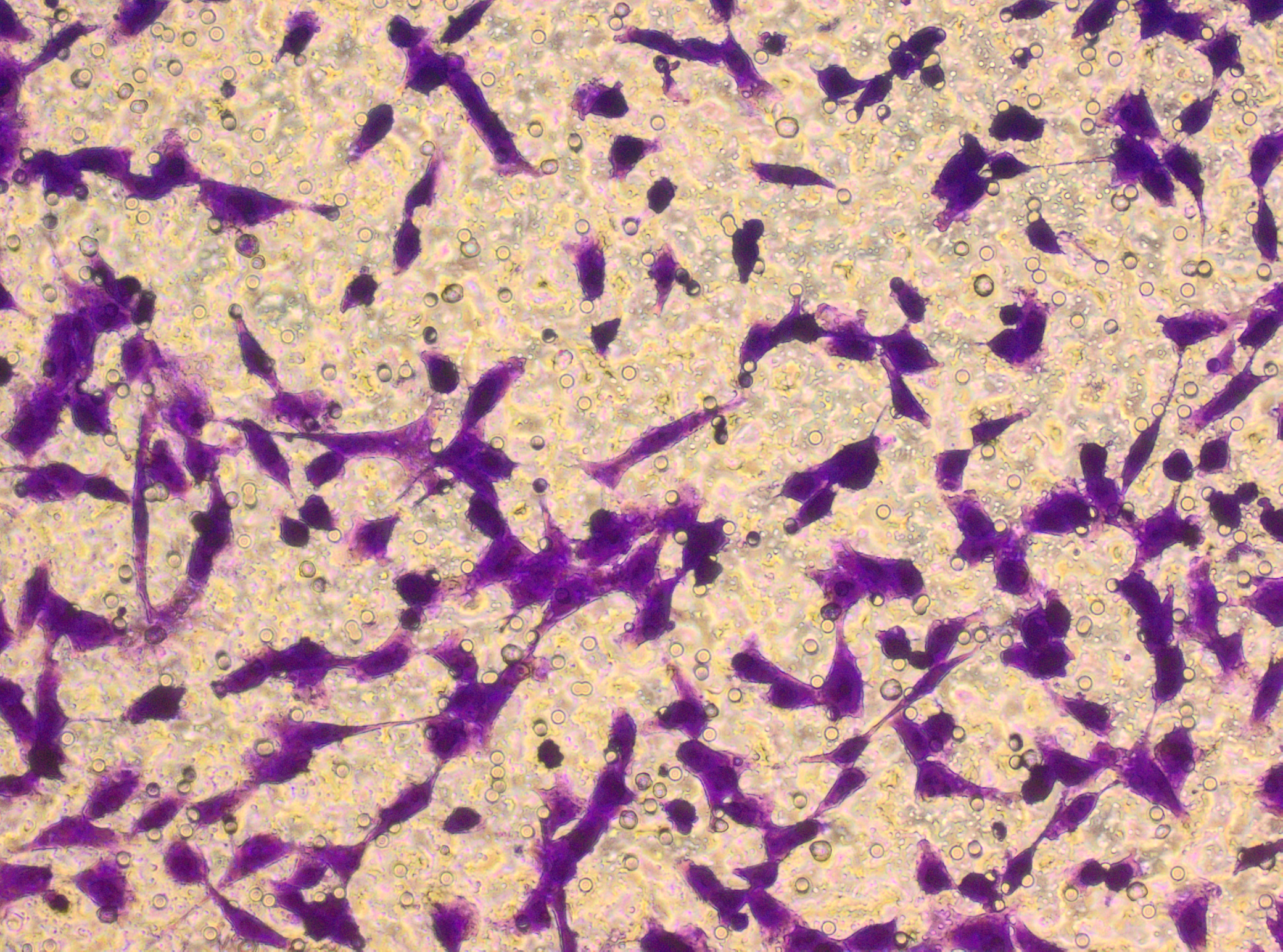

Supplement: Supplementary file 8 — Source data Fig. 4 [file 44318_2026_766_MOESM8_ESM.zip › Figure4/Fig4D/migration/dmso.tif]

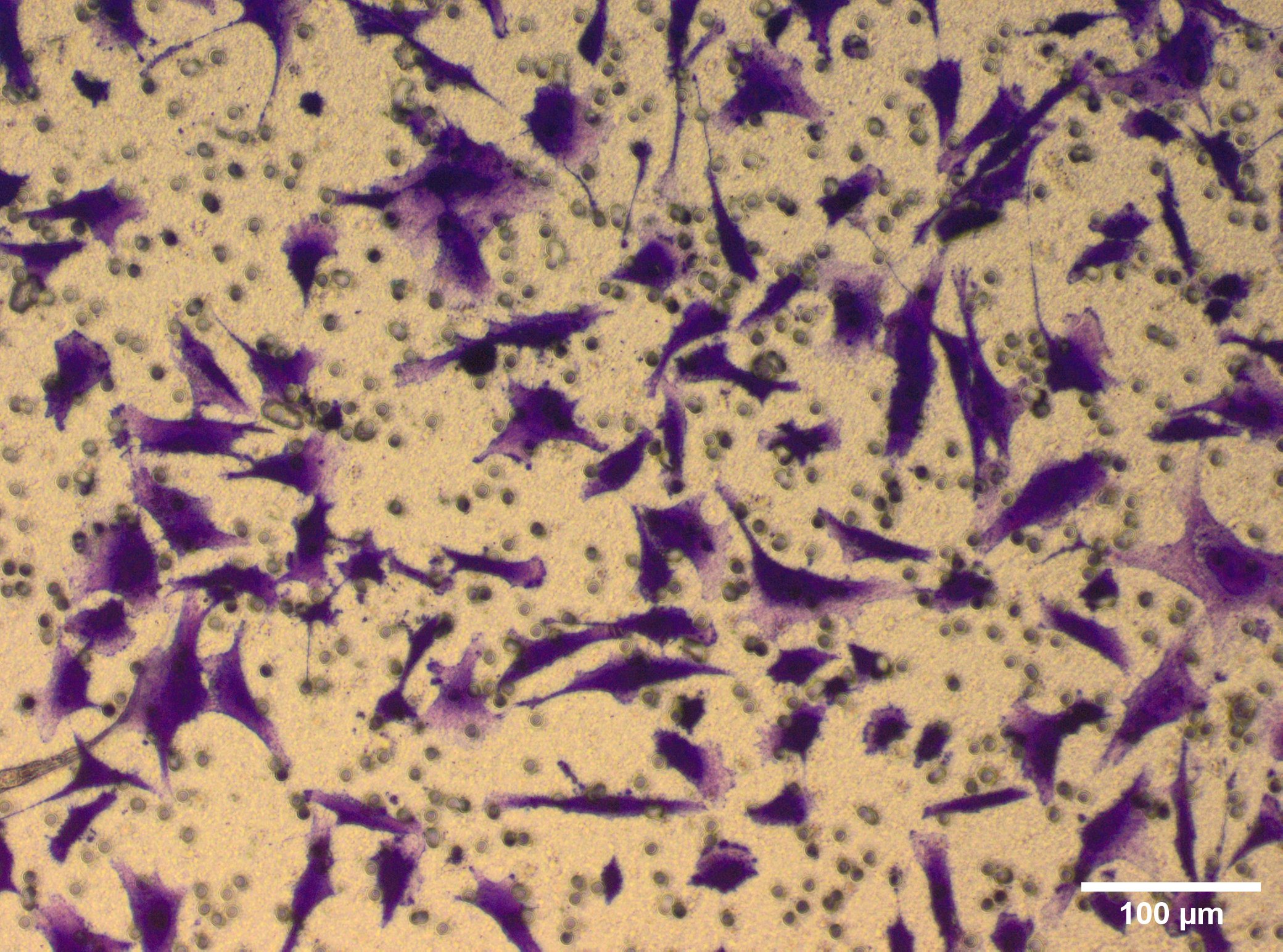

Supplement: Supplementary file 8 — Source data Fig. 4 [file 44318_2026_766_MOESM8_ESM.zip › Figure4/Fig4D/migration/Image_17690sta bt rev ev mig.jpg]

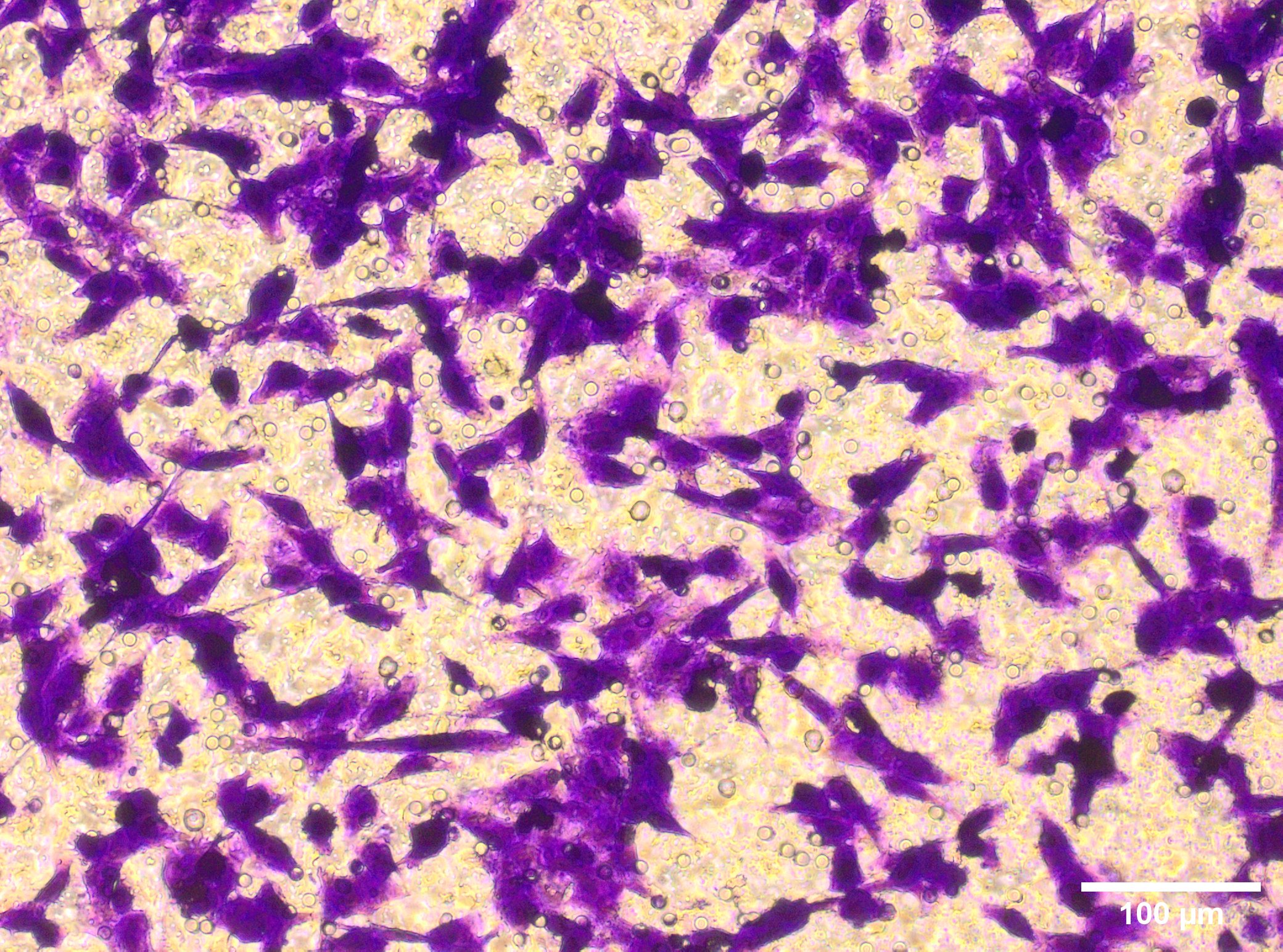

Supplement: Supplementary file 8 — Source data Fig. 4 [file 44318_2026_766_MOESM8_ESM.zip › Figure4/Fig4D/migration/rev.jpg]

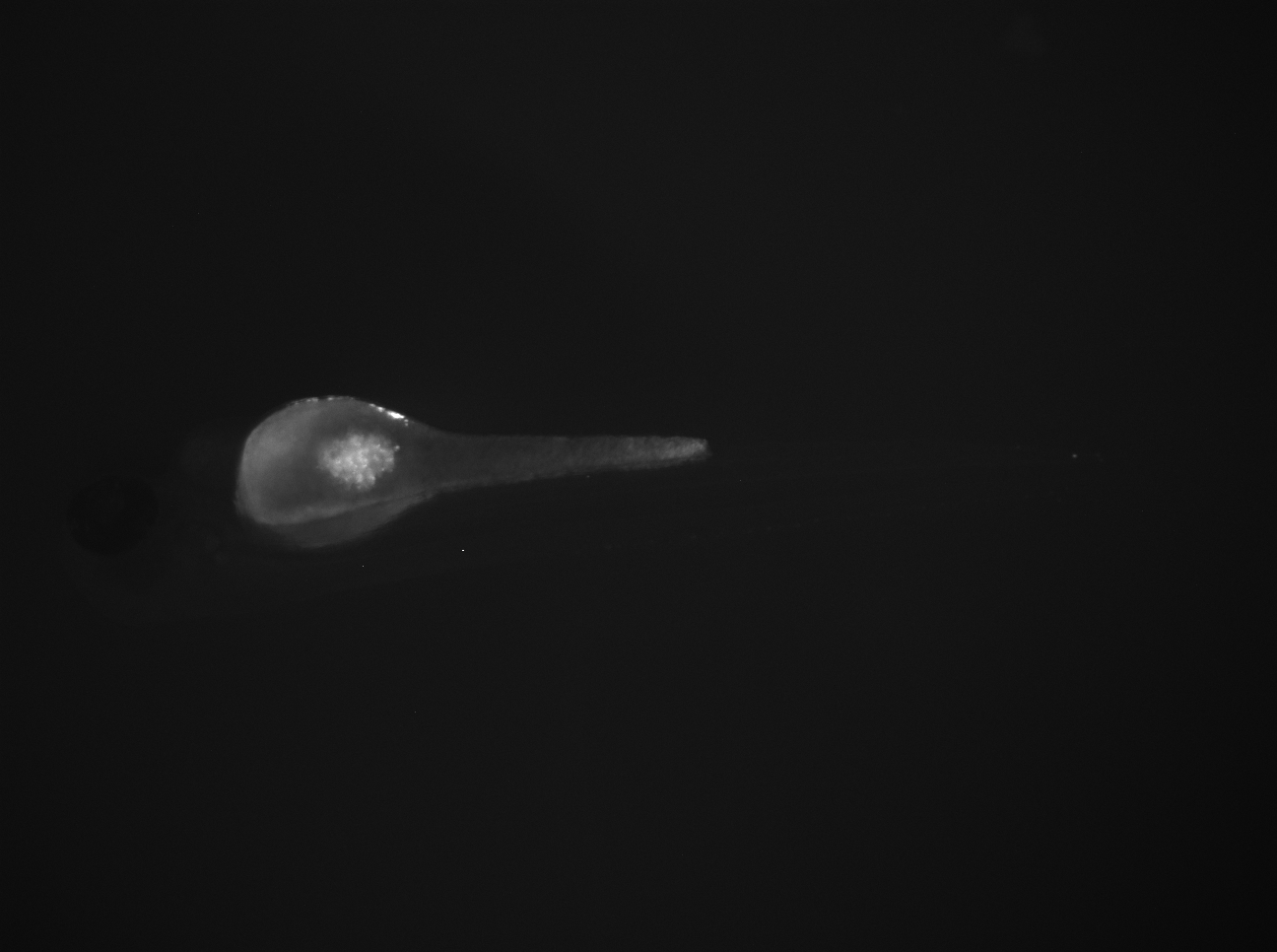

Supplement: Supplementary file 9 — Source data Fig. 5 [file 44318_2026_766_MOESM9_ESM.zip › Figure5/Fig5B/KD/image0028.tif]

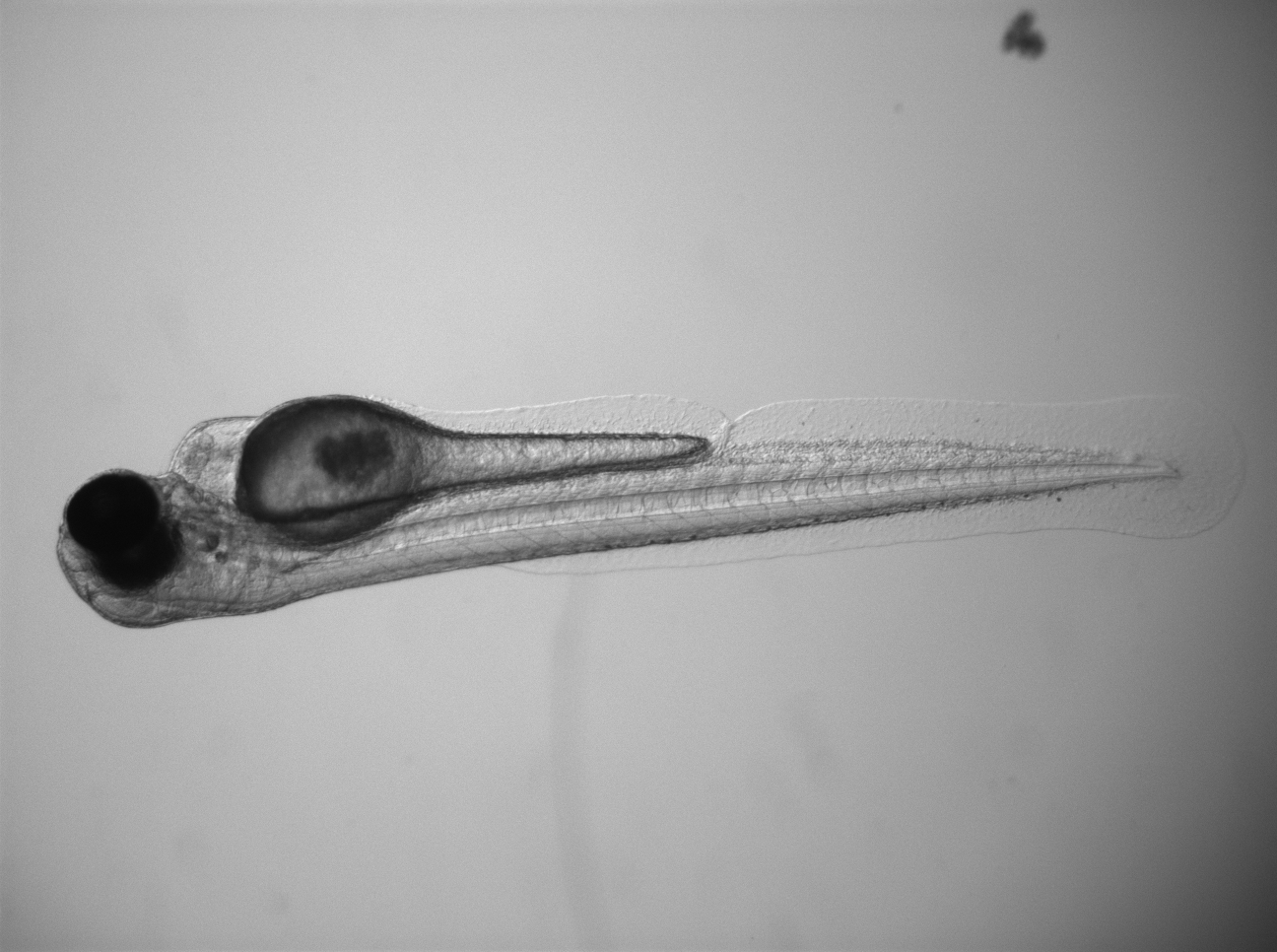

Supplement: Supplementary file 9 — Source data Fig. 5 [file 44318_2026_766_MOESM9_ESM.zip › Figure5/Fig5B/KD/image0027.tif]

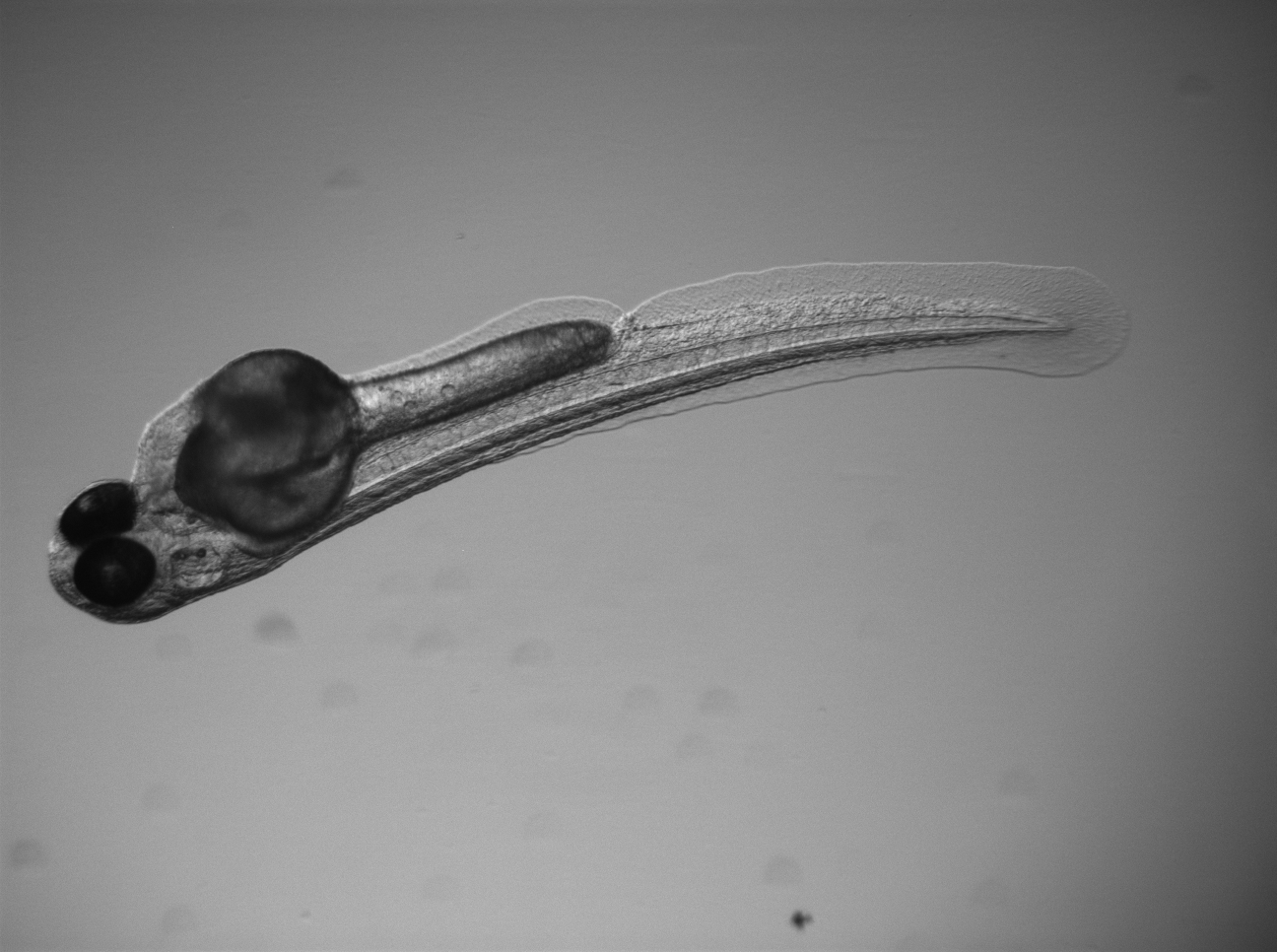

Supplement: Supplementary file 9 — Source data Fig. 5 [file 44318_2026_766_MOESM9_ESM.zip › Figure5/Fig5B/Scramble/image0012.tif]

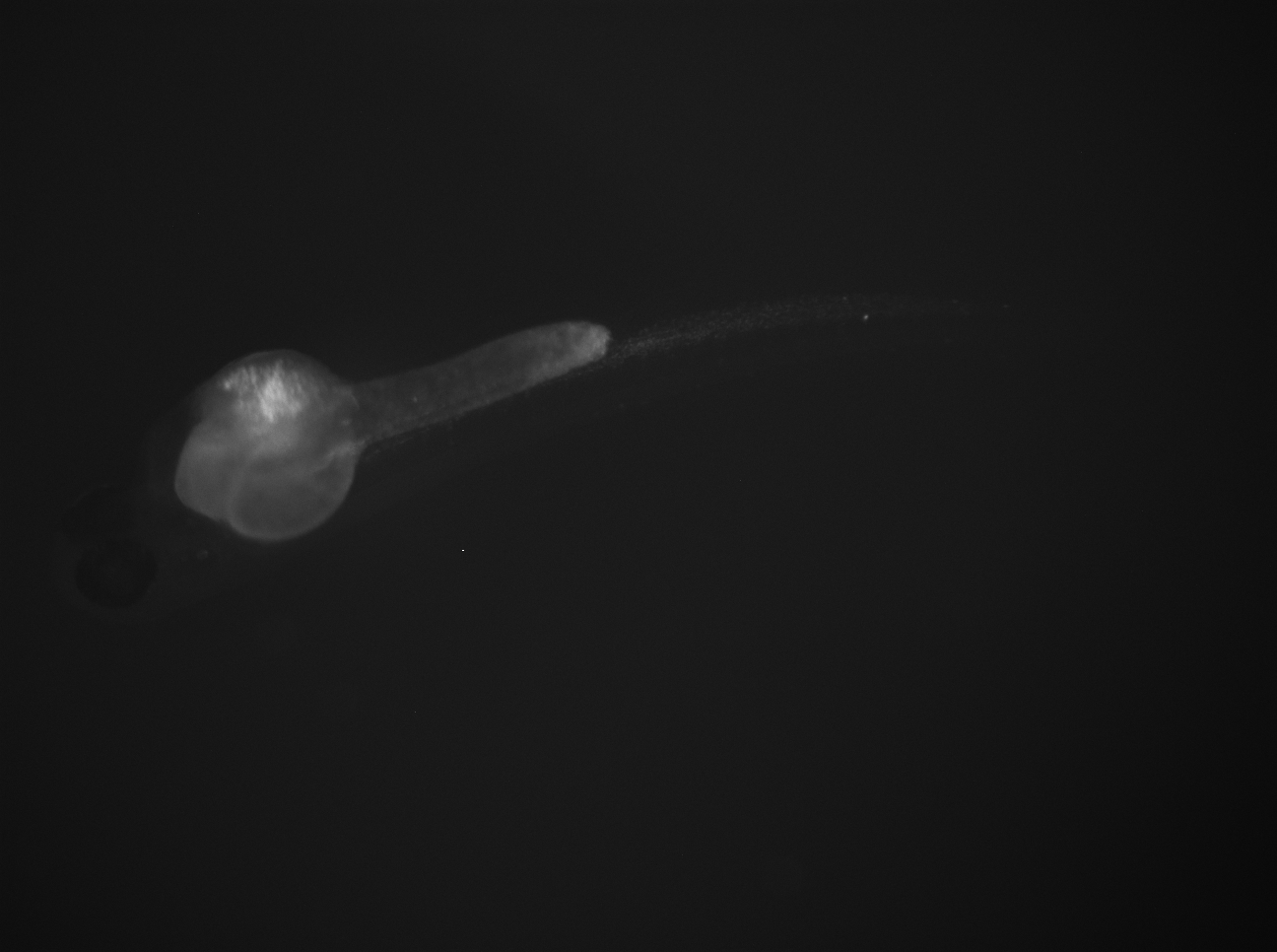

Supplement: Supplementary file 9 — Source data Fig. 5 [file 44318_2026_766_MOESM9_ESM.zip › Figure5/Fig5B/Scramble/image0013.tif]

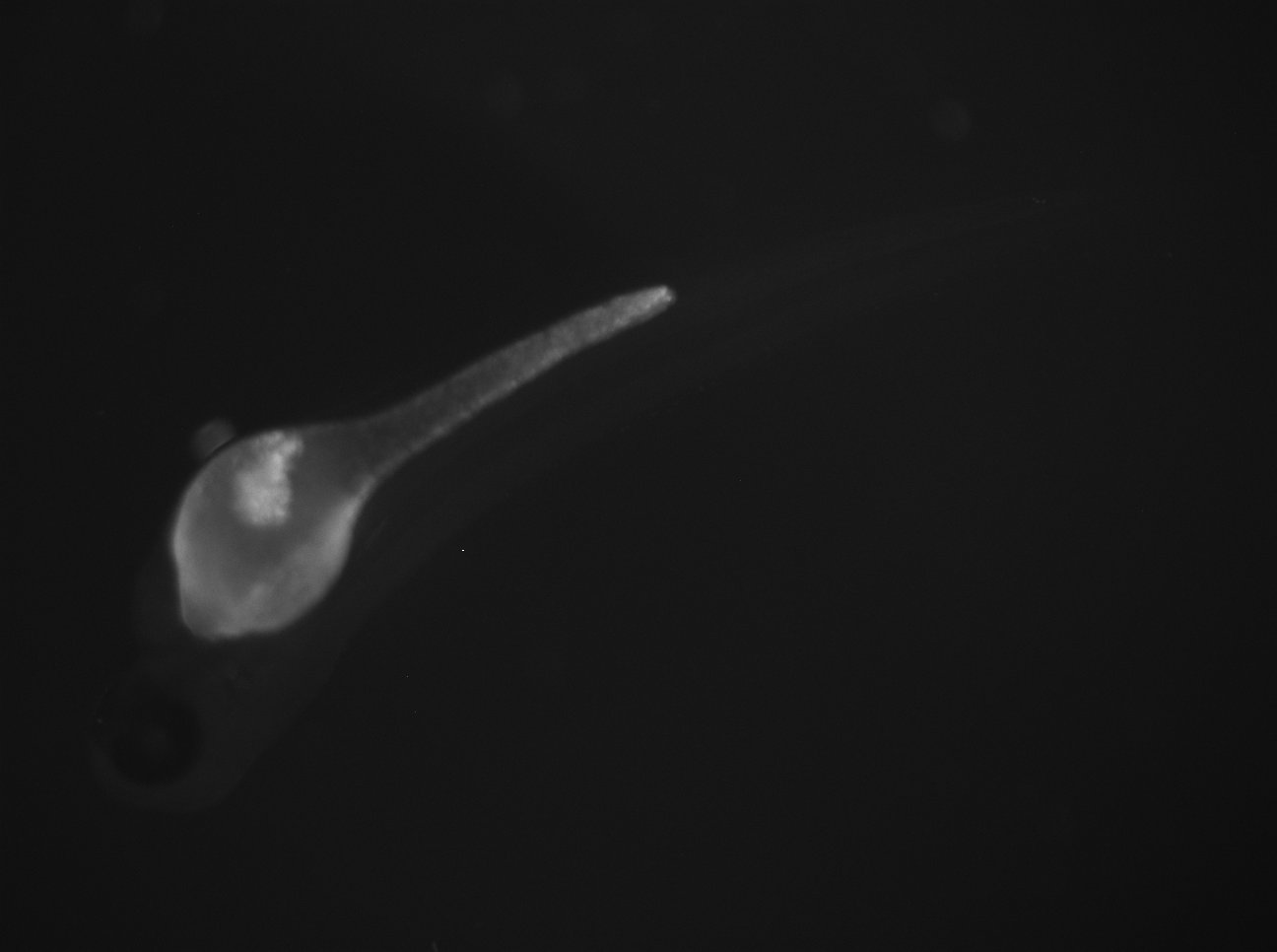

Supplement: Supplementary file 9 — Source data Fig. 5 [file 44318_2026_766_MOESM9_ESM.zip › Figure5/Fig5B/EFEMP1/image0011.tif]

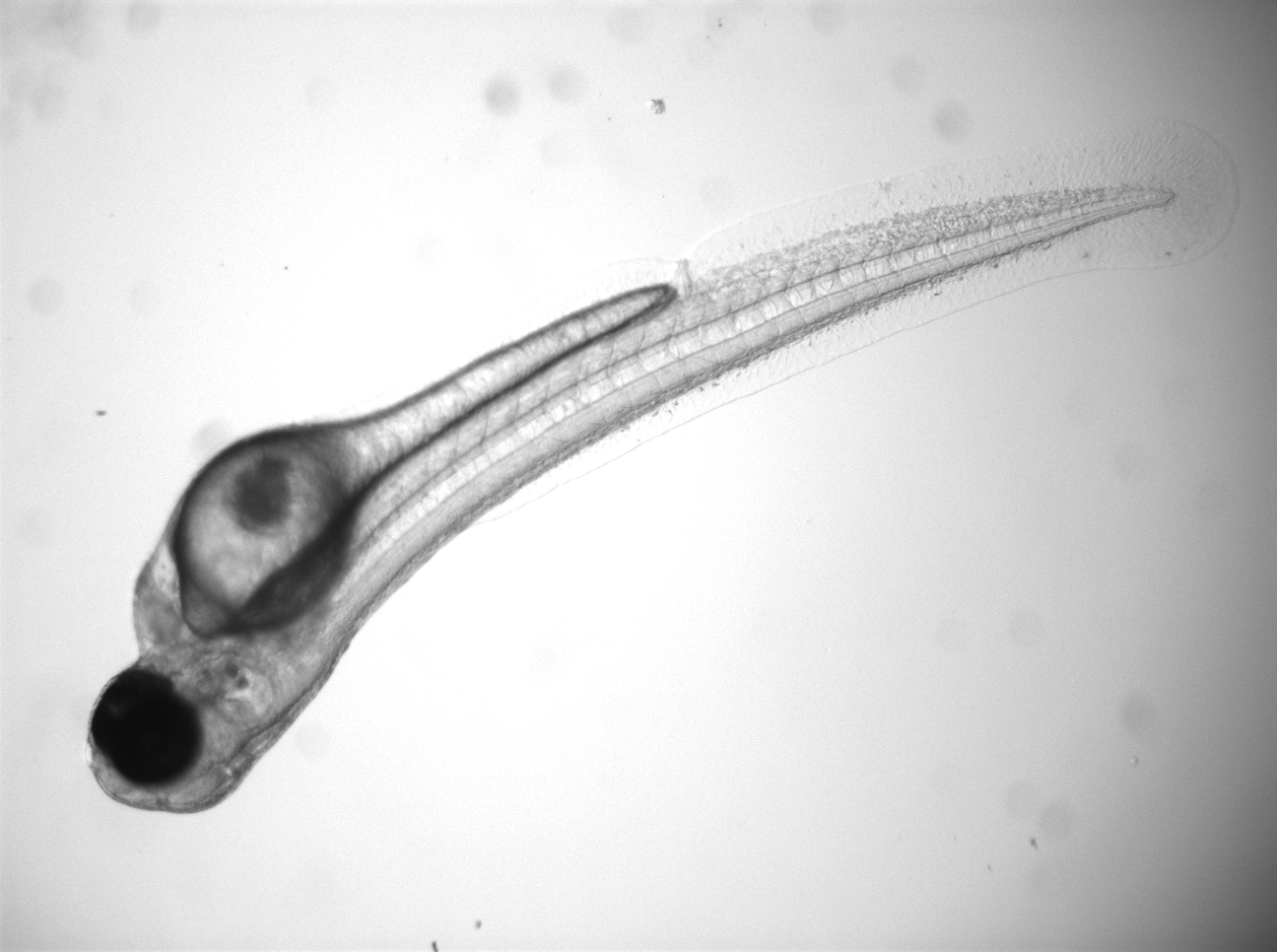

Supplement: Supplementary file 9 — Source data Fig. 5 [file 44318_2026_766_MOESM9_ESM.zip › Figure5/Fig5B/EFEMP1/image0010.tif]

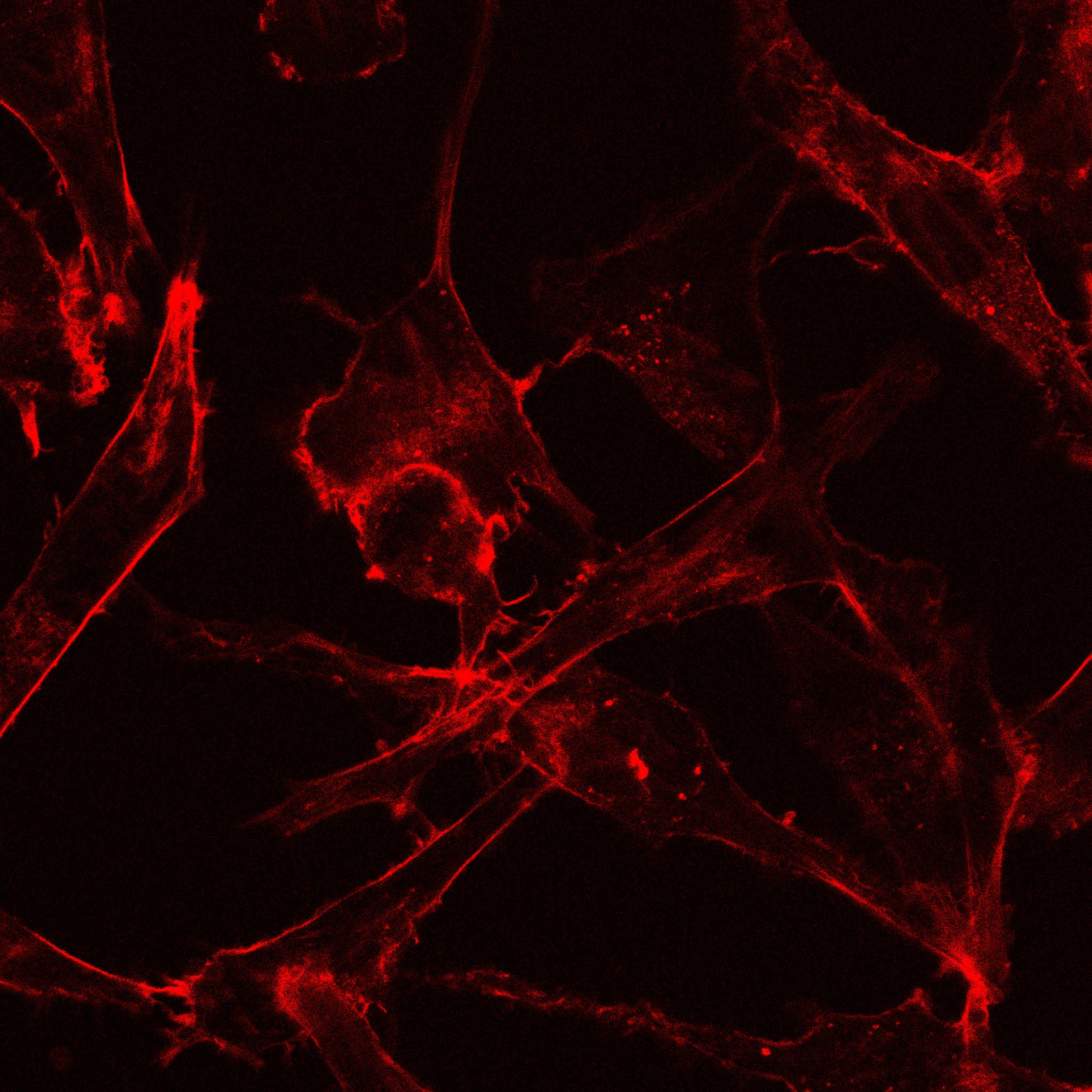

Supplement: Supplementary file 10 — EV Figure Source Data [file 44318_2026_766_MOESM10_ESM.zip › Figure EV1/Fig EV 1E/negative/f-actin.jpg]

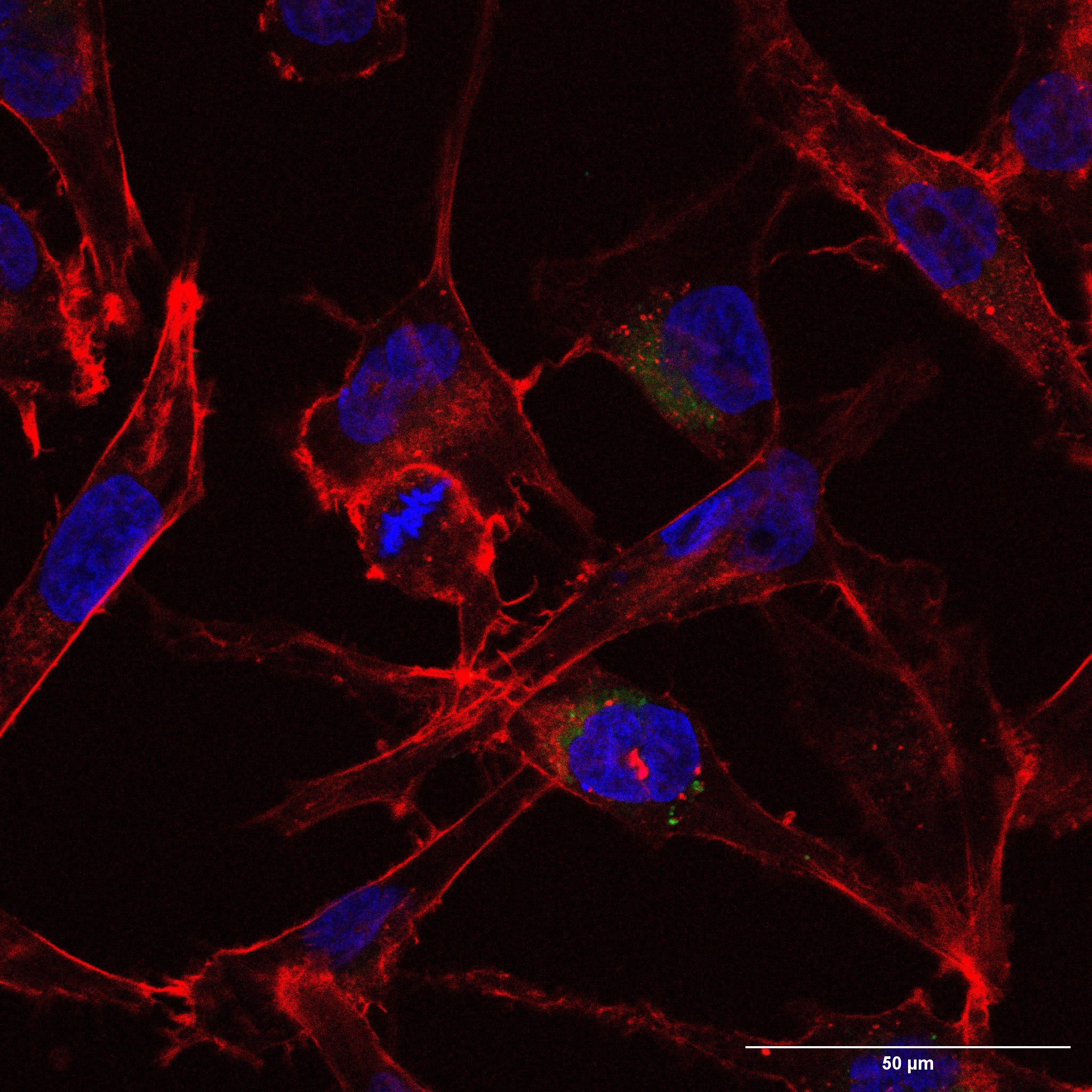

Supplement: Supplementary file 10 — EV Figure Source Data [file 44318_2026_766_MOESM10_ESM.zip › Figure EV1/Fig EV 1E/negative/Composite2.jpg]

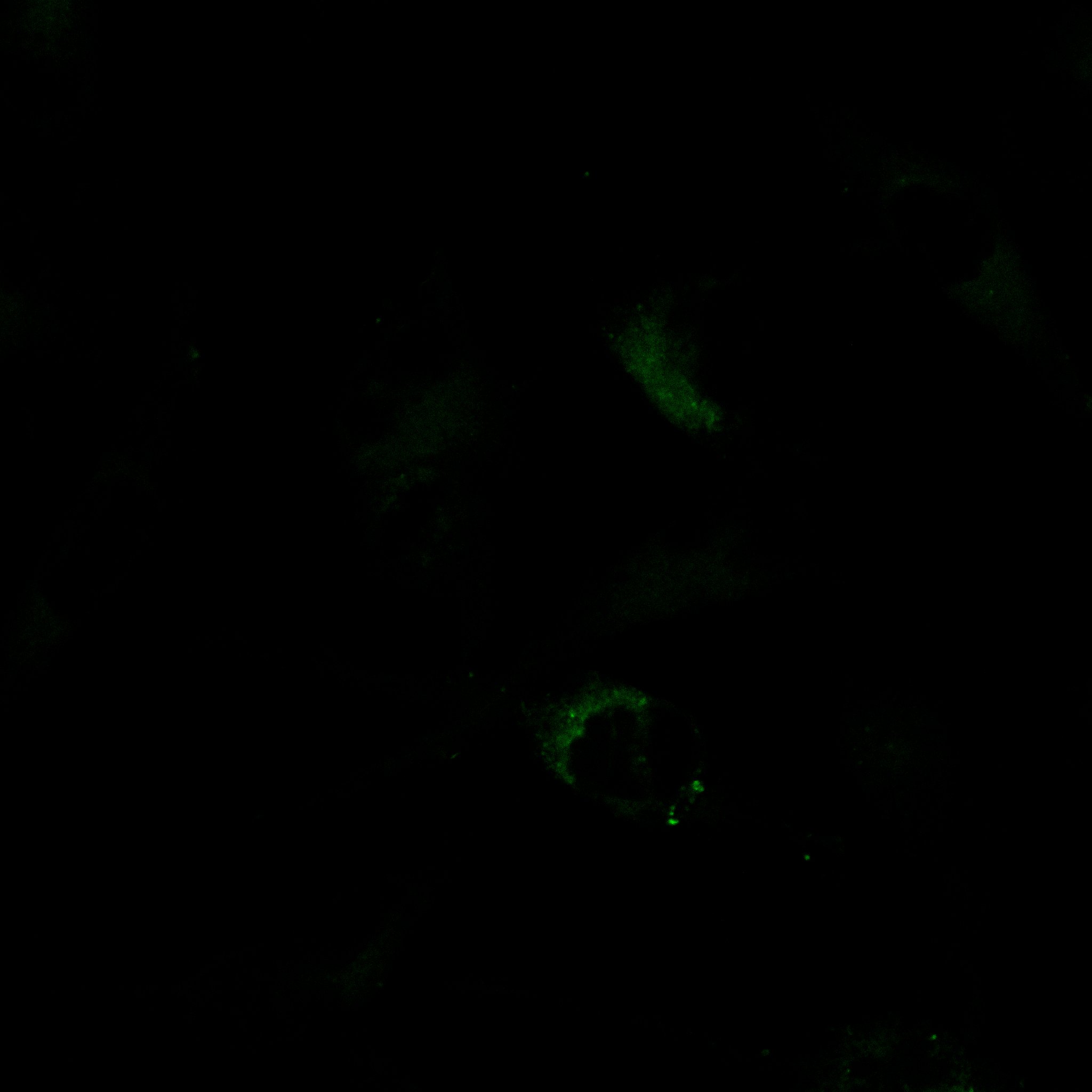

Supplement: Supplementary file 10 — EV Figure Source Data [file 44318_2026_766_MOESM10_ESM.zip › Figure EV1/Fig EV 1E/negative/pkh67.jpg]

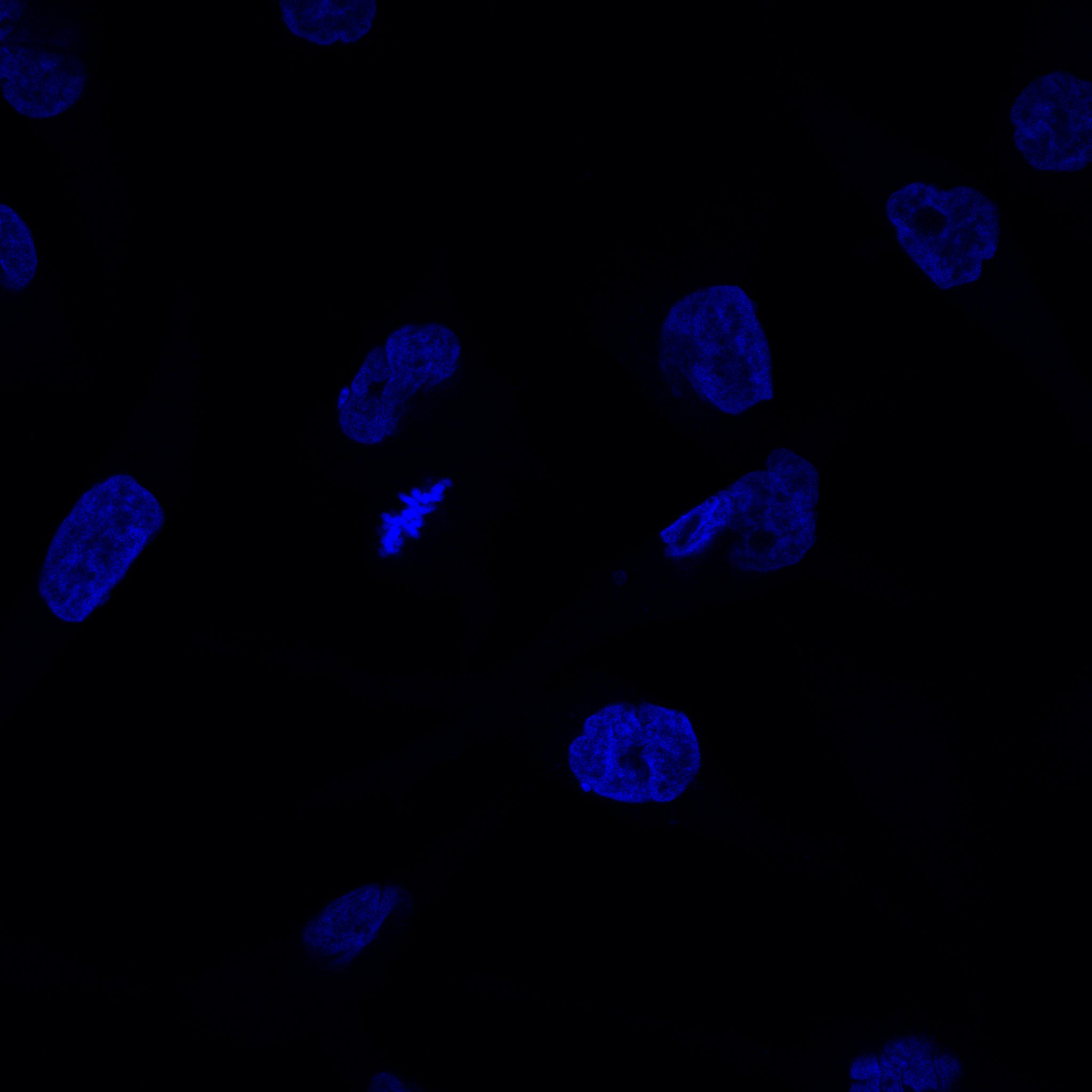

Supplement: Supplementary file 10 — EV Figure Source Data [file 44318_2026_766_MOESM10_ESM.zip › Figure EV1/Fig EV 1E/negative/dapi.jpg]

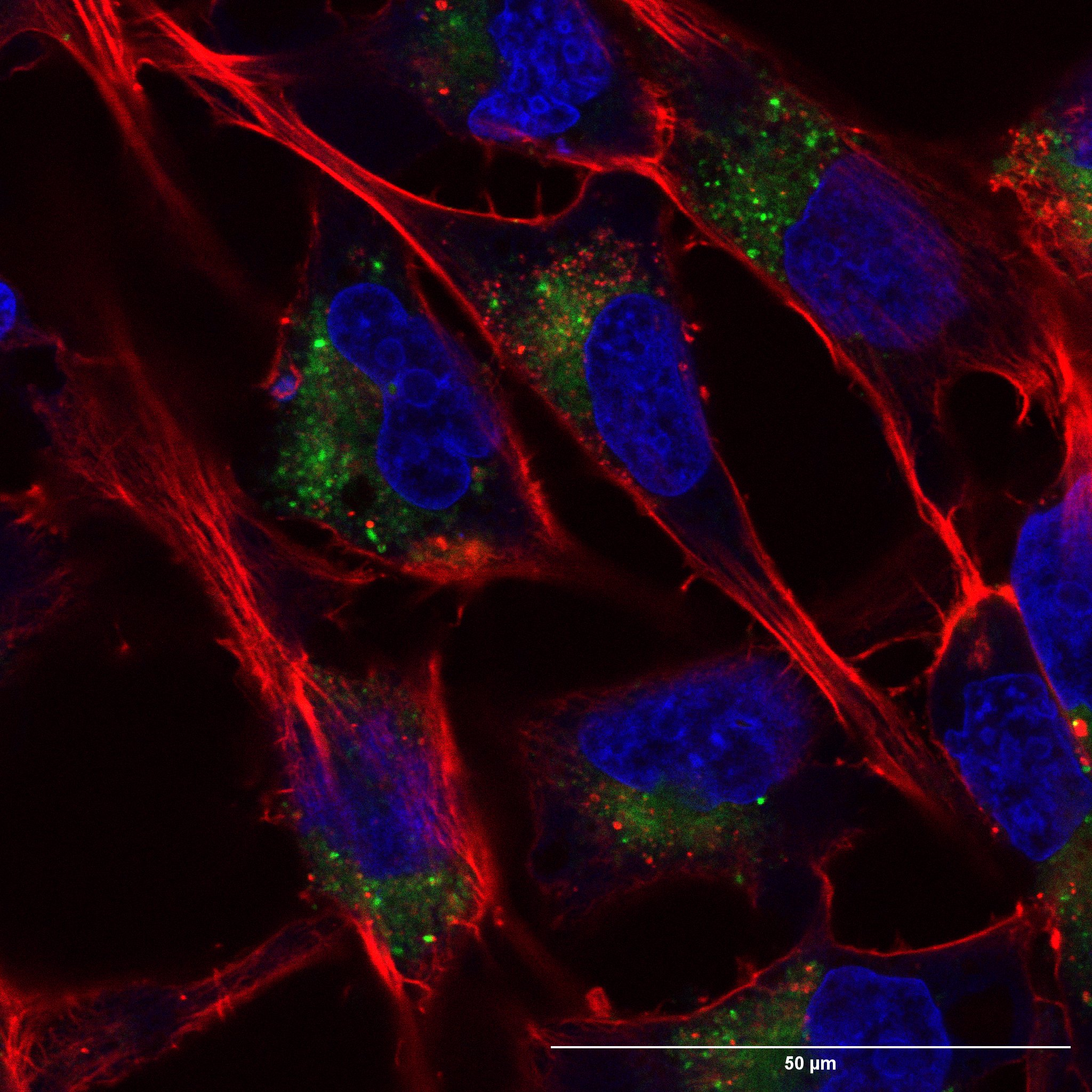

Supplement: Supplementary file 10 — EV Figure Source Data [file 44318_2026_766_MOESM10_ESM.zip › Figure EV1/Fig EV 1E/REV EVs/Composite2.jpg]

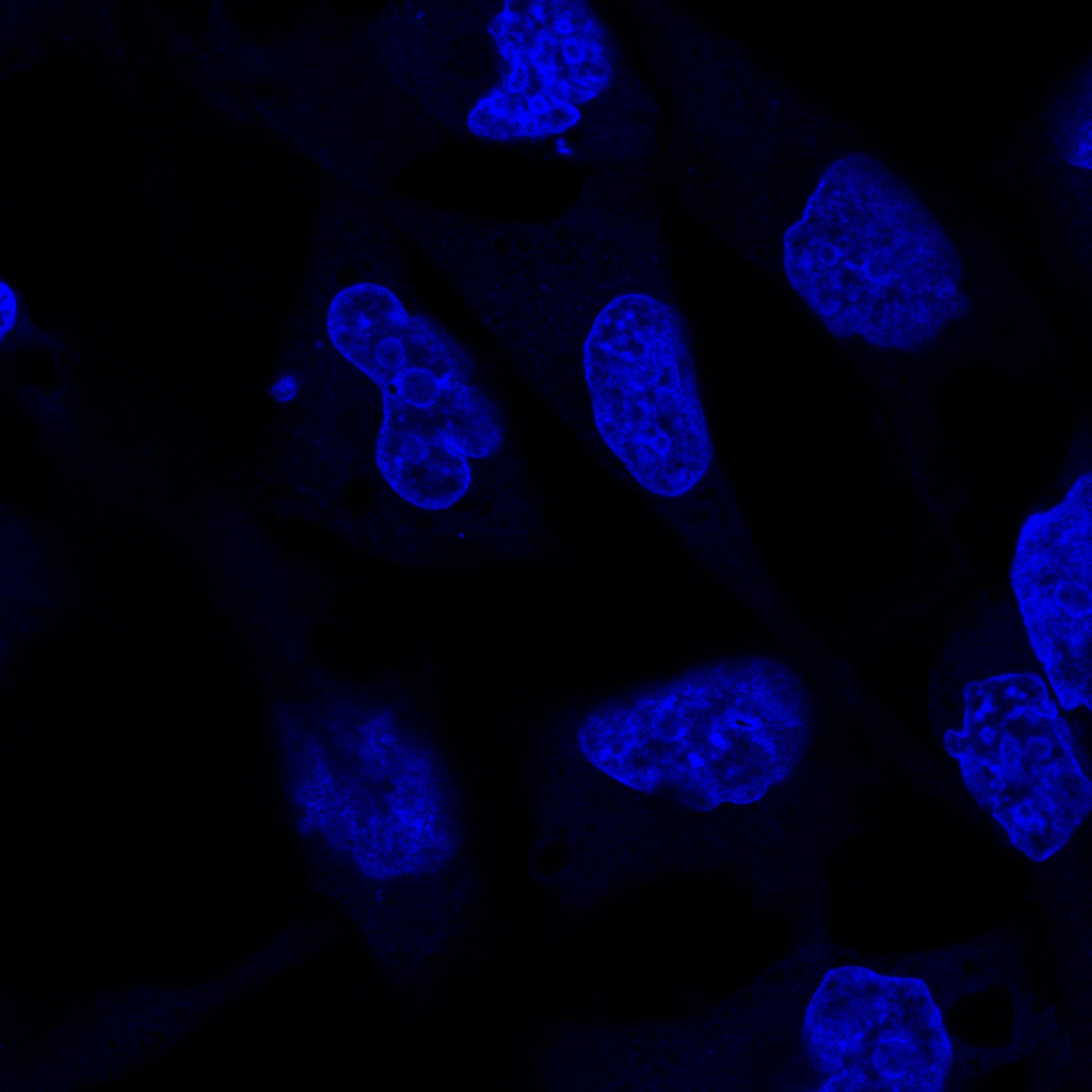

Supplement: Supplementary file 10 — EV Figure Source Data [file 44318_2026_766_MOESM10_ESM.zip › Figure EV1/Fig EV 1E/REV EVs/dapi.jpg]

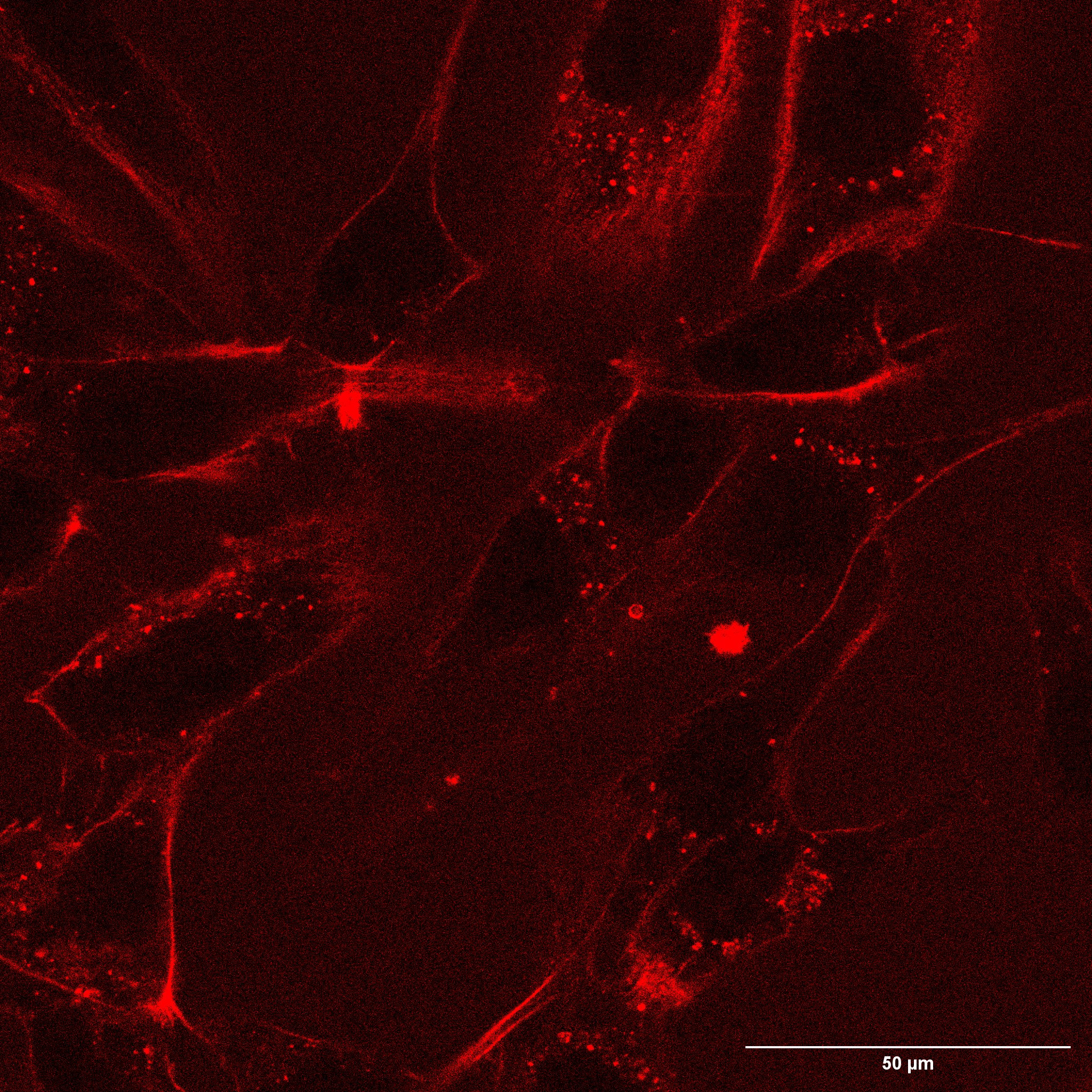

Supplement: Supplementary file 10 — EV Figure Source Data [file 44318_2026_766_MOESM10_ESM.zip › Figure EV1/Fig EV 1E/DMSO EVs/f-actin.jpg]

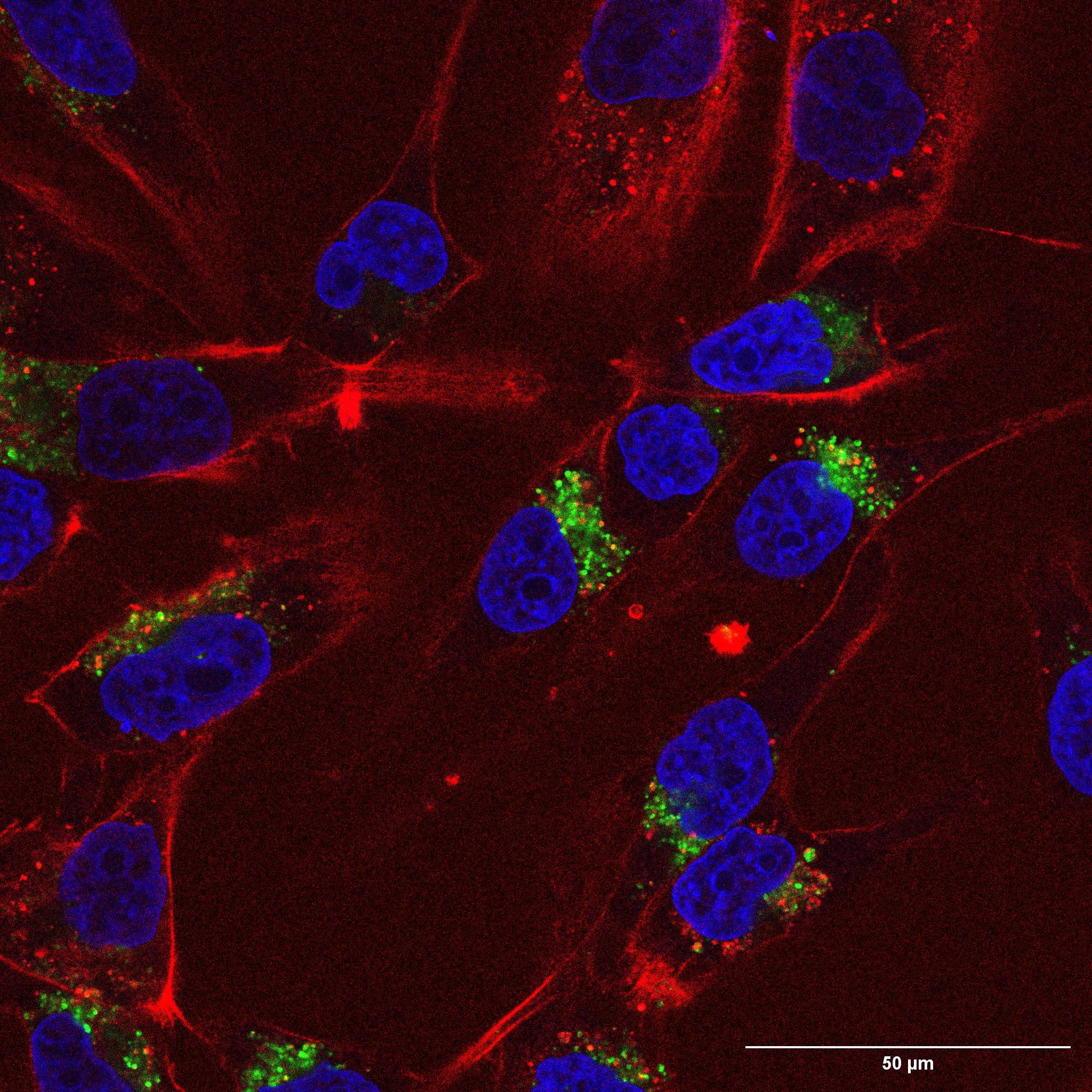

Supplement: Supplementary file 10 — EV Figure Source Data [file 44318_2026_766_MOESM10_ESM.zip › Figure EV1/Fig EV 1E/DMSO EVs/Composite2.jpg]

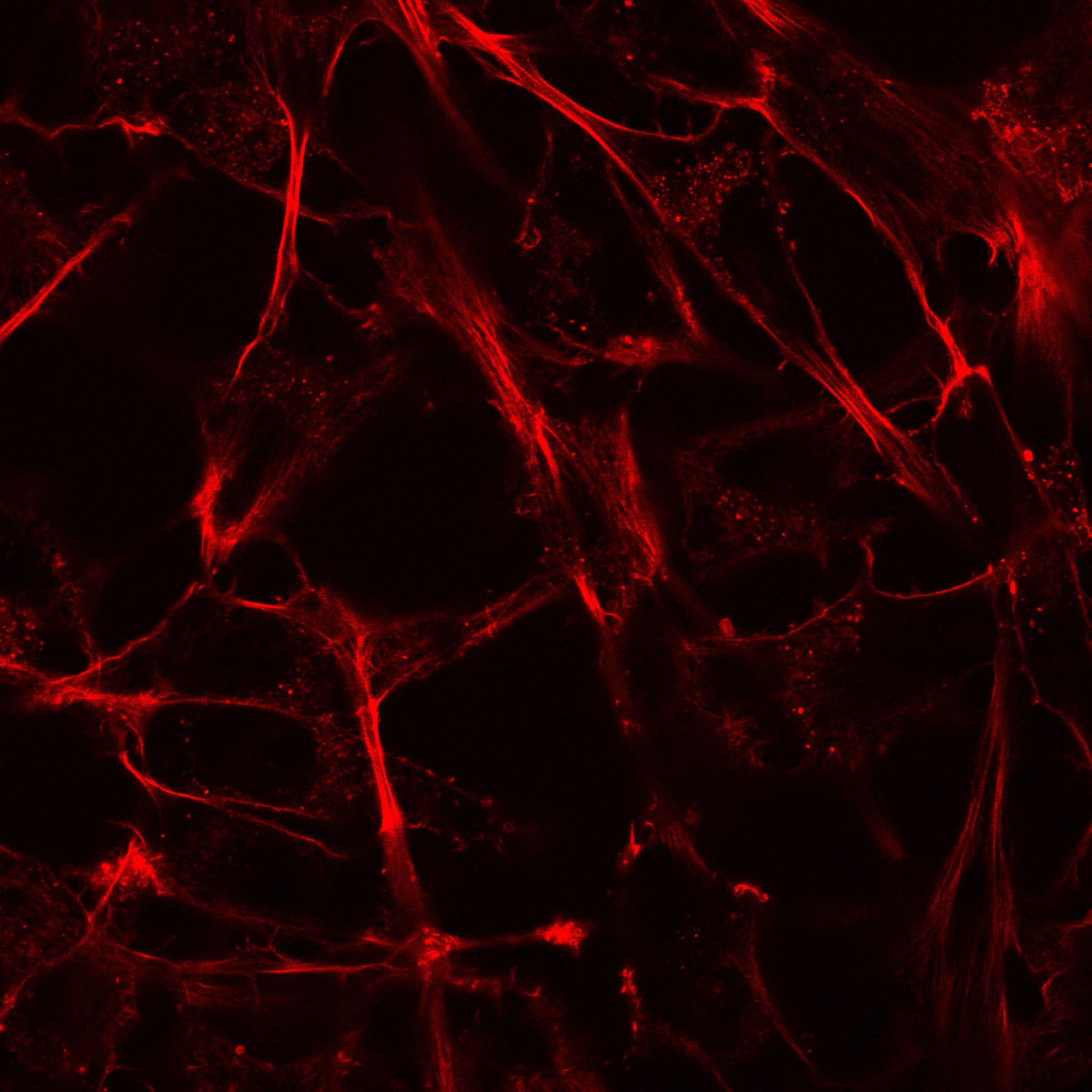

Supplement: Supplementary file 10 — EV Figure Source Data [file 44318_2026_766_MOESM10_ESM.zip › Figure EV1/Fig EV 1E/DMSO 4Γäâ/f-actin.jpg]

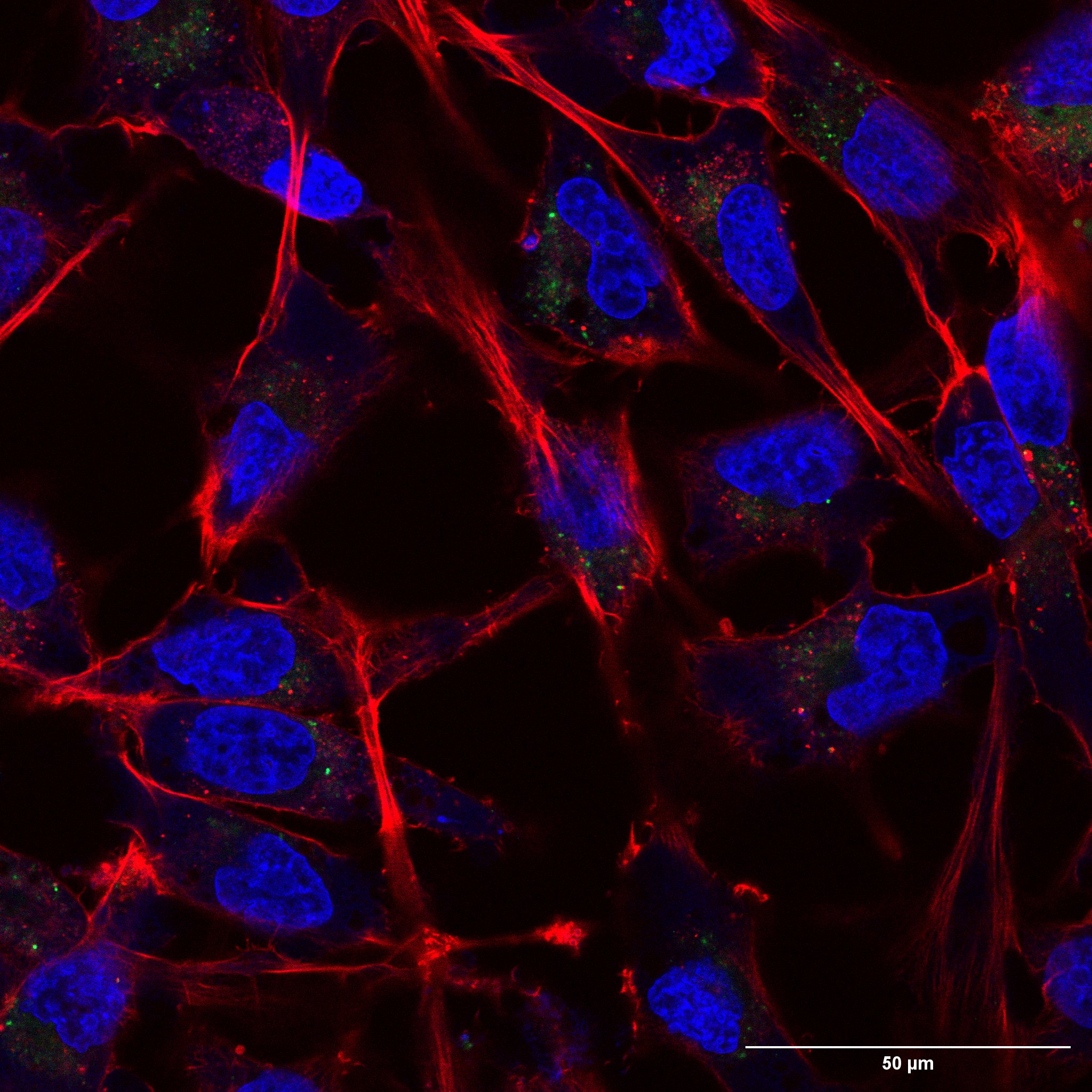

Supplement: Supplementary file 10 — EV Figure Source Data [file 44318_2026_766_MOESM10_ESM.zip › Figure EV1/Fig EV 1E/DMSO 4Γäâ/Composite2.jpg]

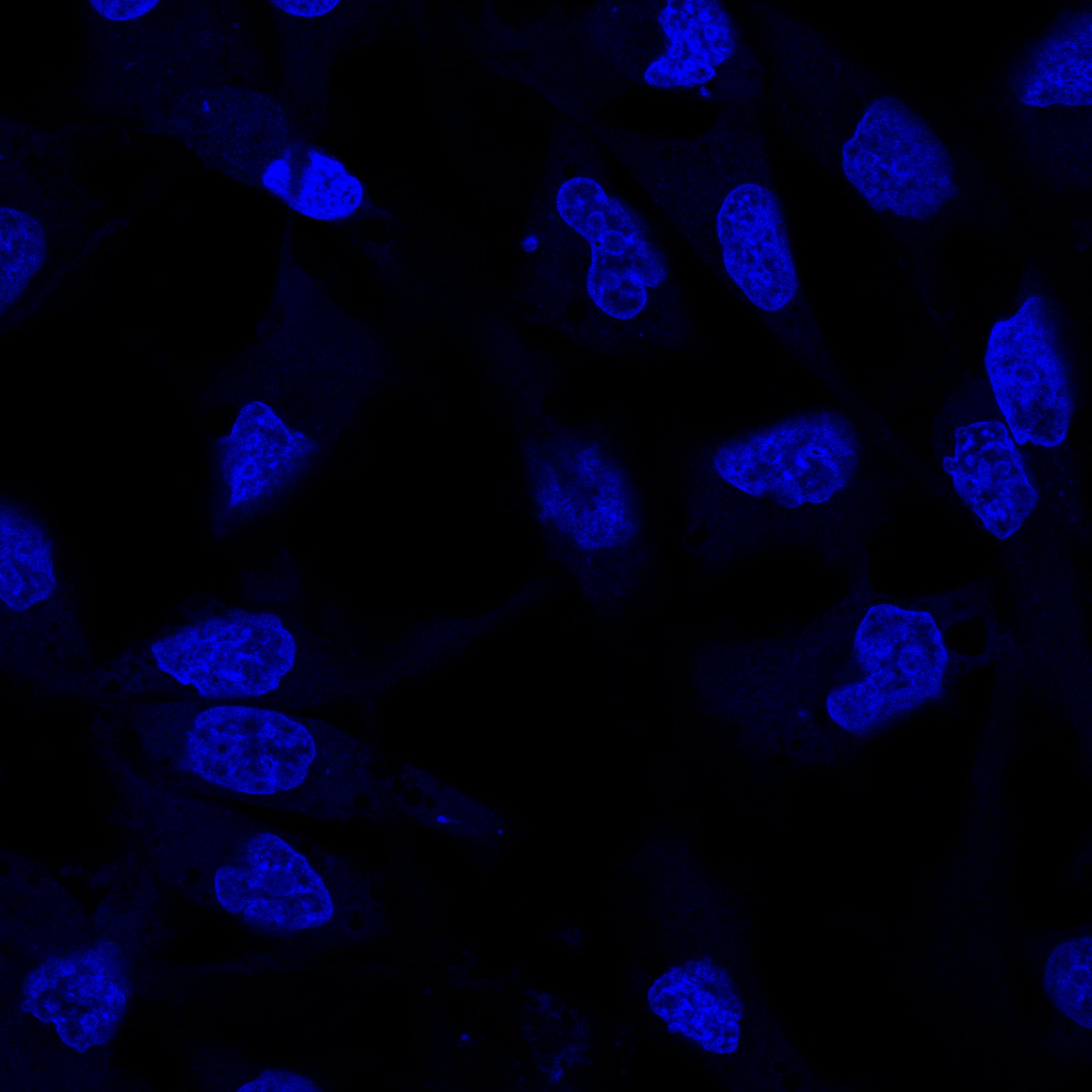

Supplement: Supplementary file 10 — EV Figure Source Data [file 44318_2026_766_MOESM10_ESM.zip › Figure EV1/Fig EV 1E/DMSO 4Γäâ/dapi.jpg]

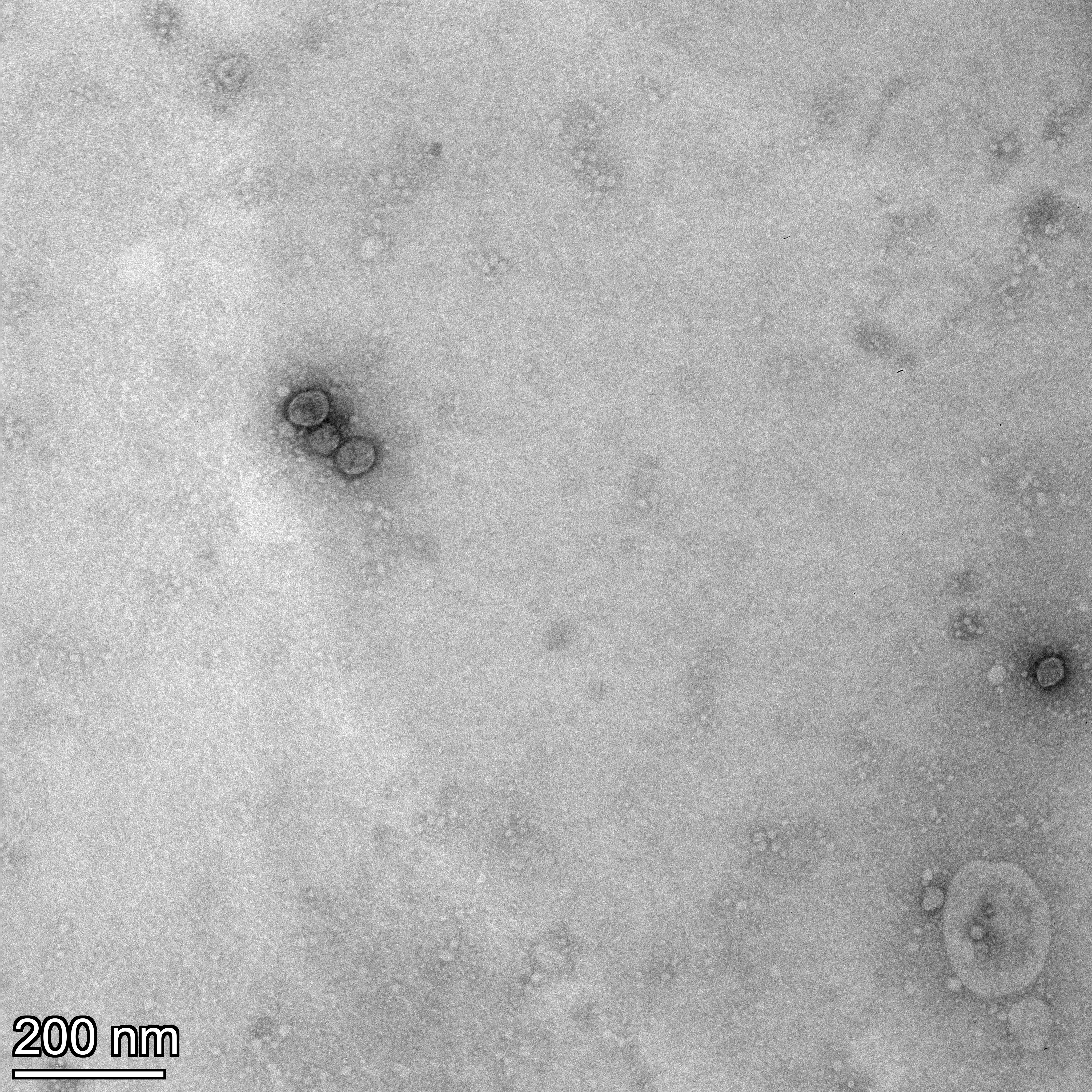

Supplement: Supplementary file 10 — EV Figure Source Data [file 44318_2026_766_MOESM10_ESM.zip › Figure EV1/Fig EV 1A/sup1a/REV EVs1059 sample2 36000 x Ceta.png]

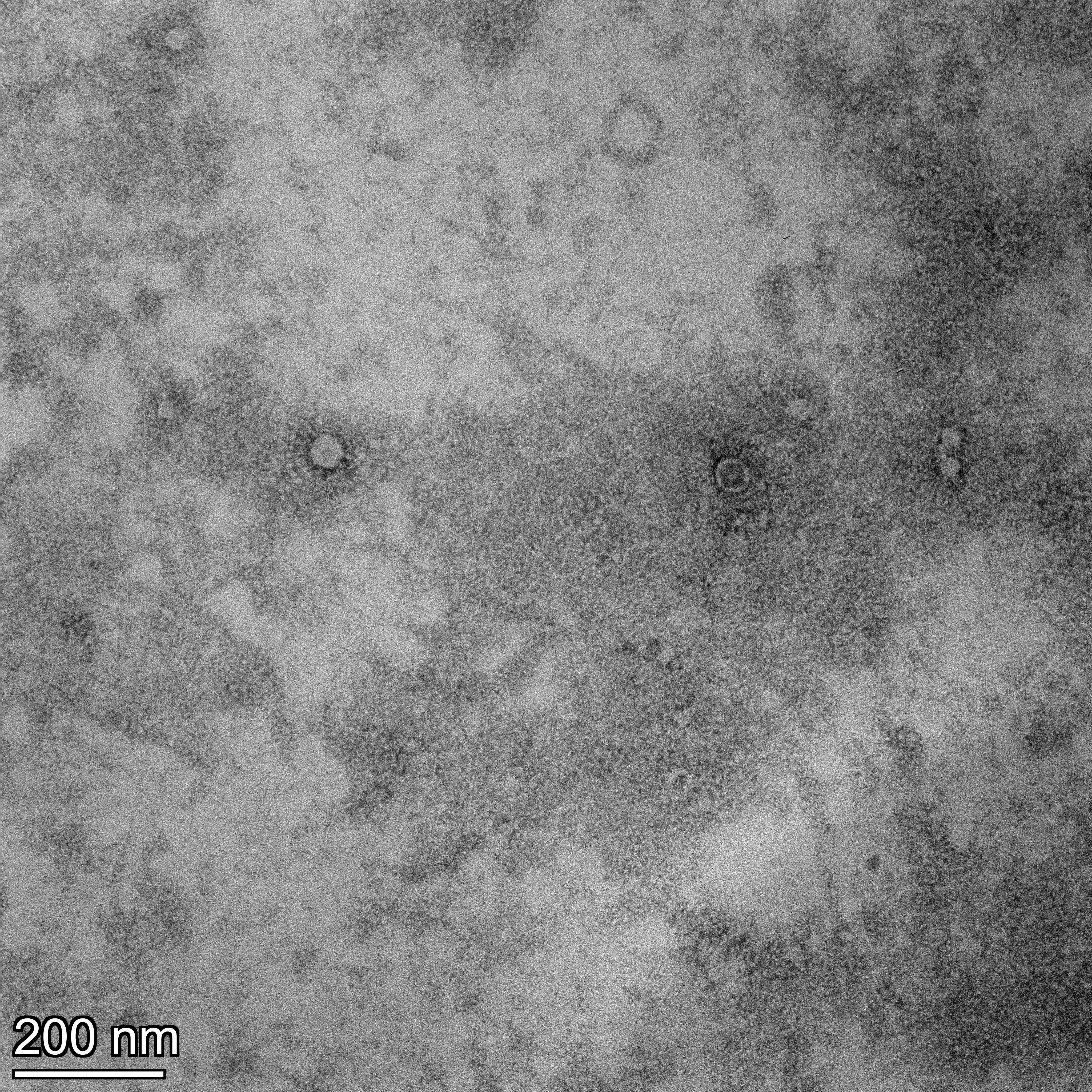

Supplement: Supplementary file 10 — EV Figure Source Data [file 44318_2026_766_MOESM10_ESM.zip › Figure EV1/Fig EV 1A/sup1a/DMSO EVs1208 sample3 36000 x Ceta.png]

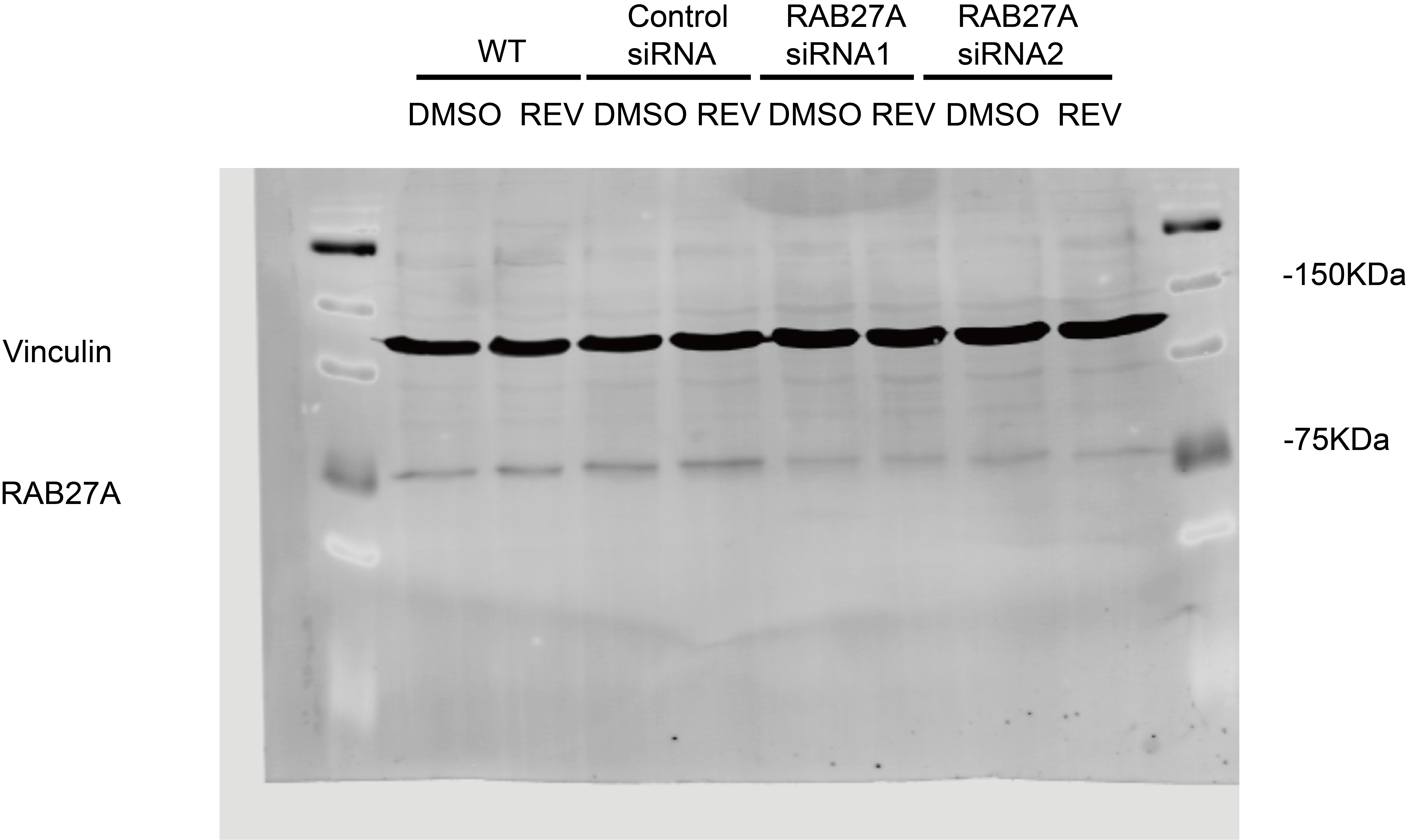

Supplement: Supplementary file 10 — EV Figure Source Data [file 44318_2026_766_MOESM10_ESM.zip › Figure EV2/Fig EV 2I.png]

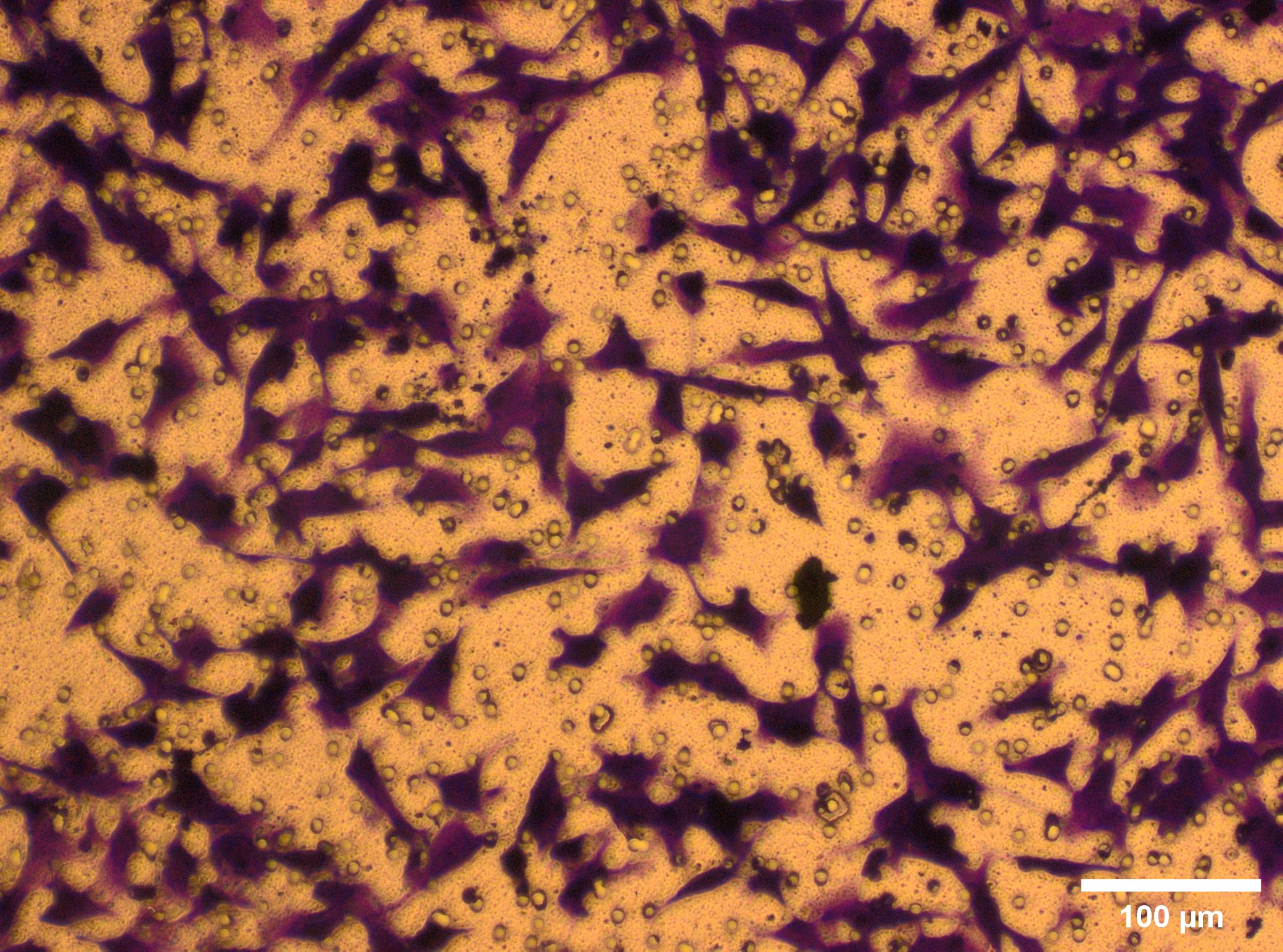

Supplement: Supplementary file 10 — EV Figure Source Data [file 44318_2026_766_MOESM10_ESM.zip › Figure EV2/Fig EV 2K/control siRNA DMSO migra.jpg]

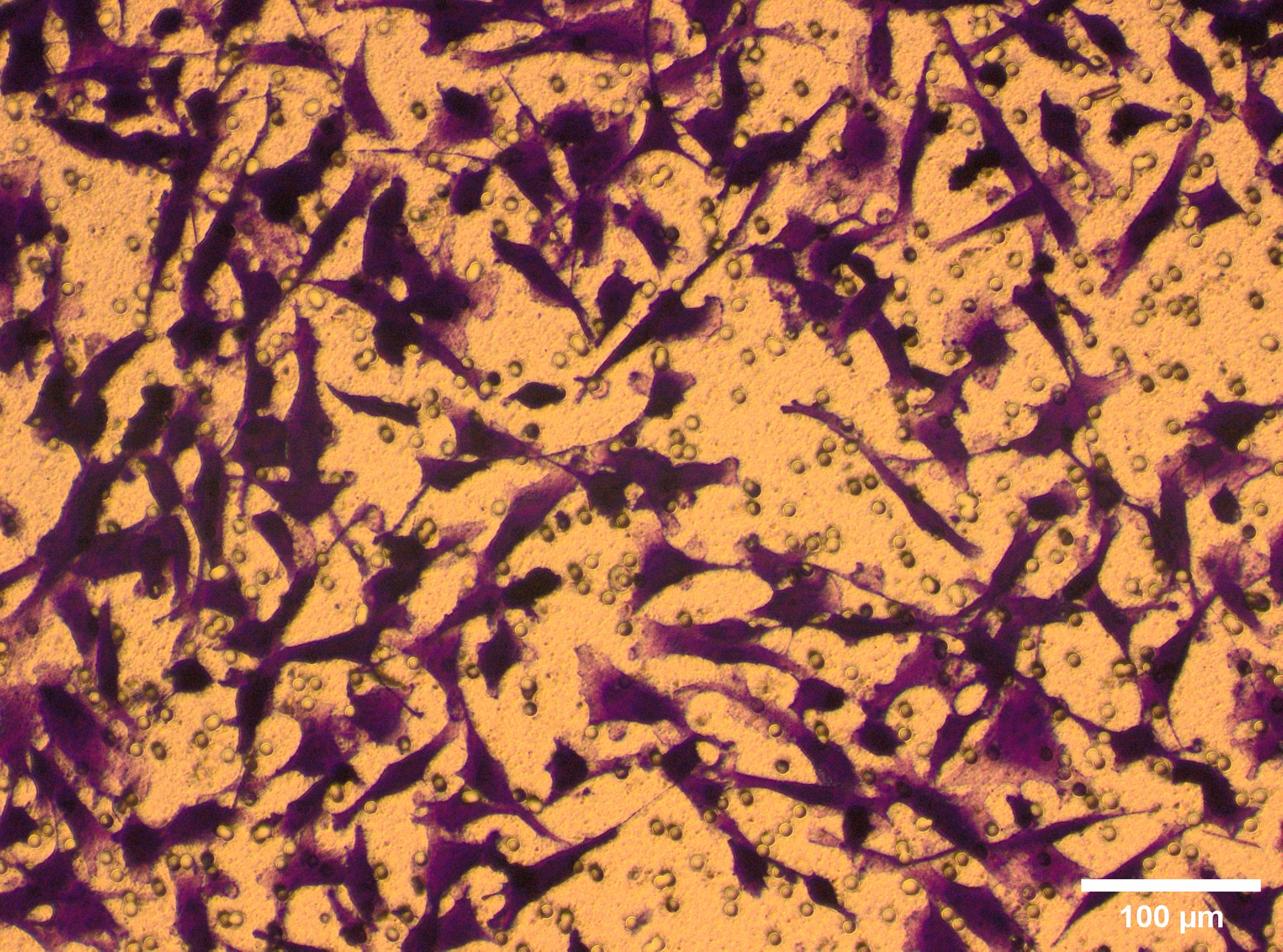

Supplement: Supplementary file 10 — EV Figure Source Data [file 44318_2026_766_MOESM10_ESM.zip › Figure EV2/Fig EV 2K/DMSO WT MIGRA.jpg]

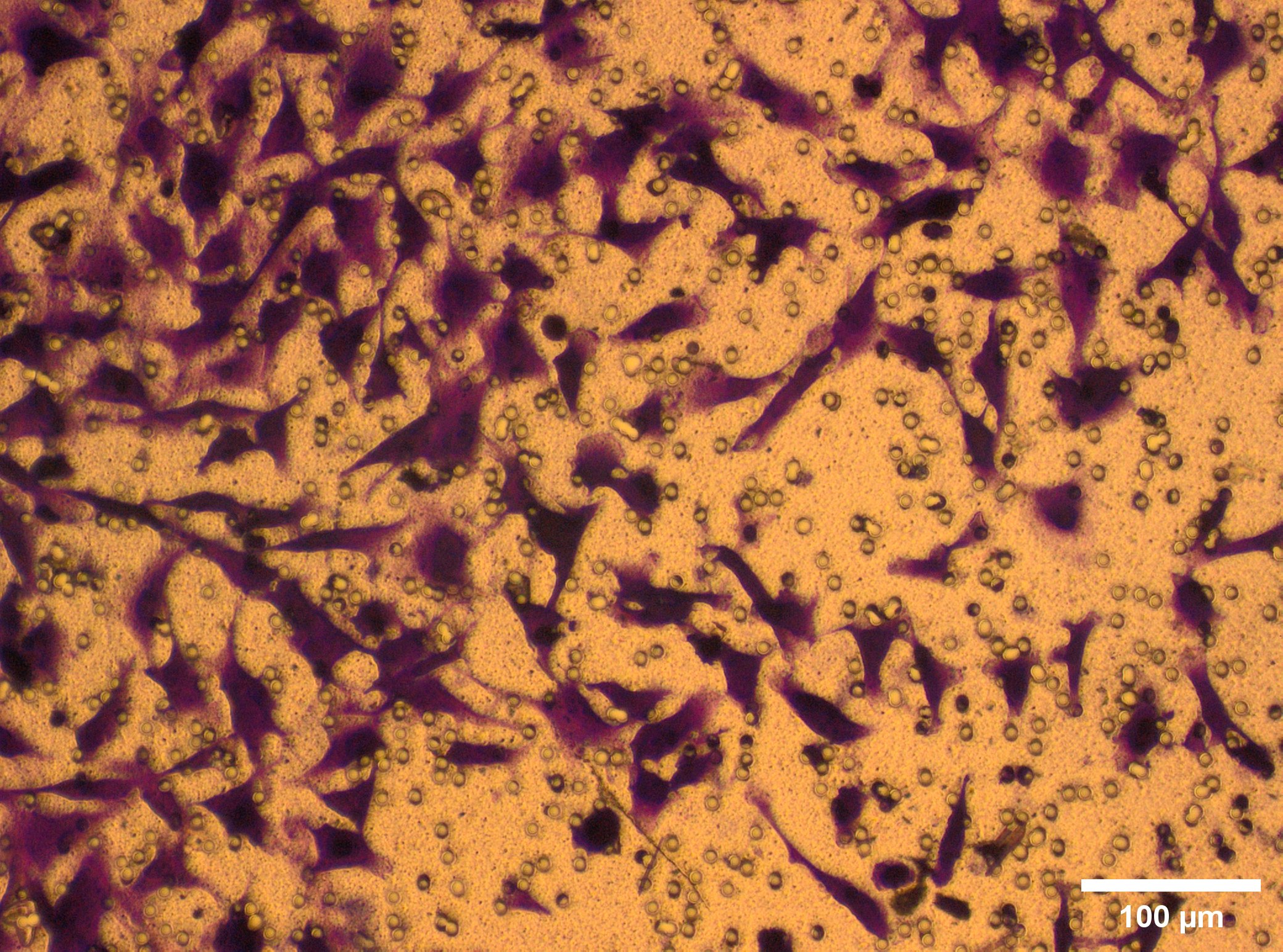

Supplement: Supplementary file 10 — EV Figure Source Data [file 44318_2026_766_MOESM10_ESM.zip › Figure EV2/Fig EV 2K/rab27a siRNA REV.jpg]

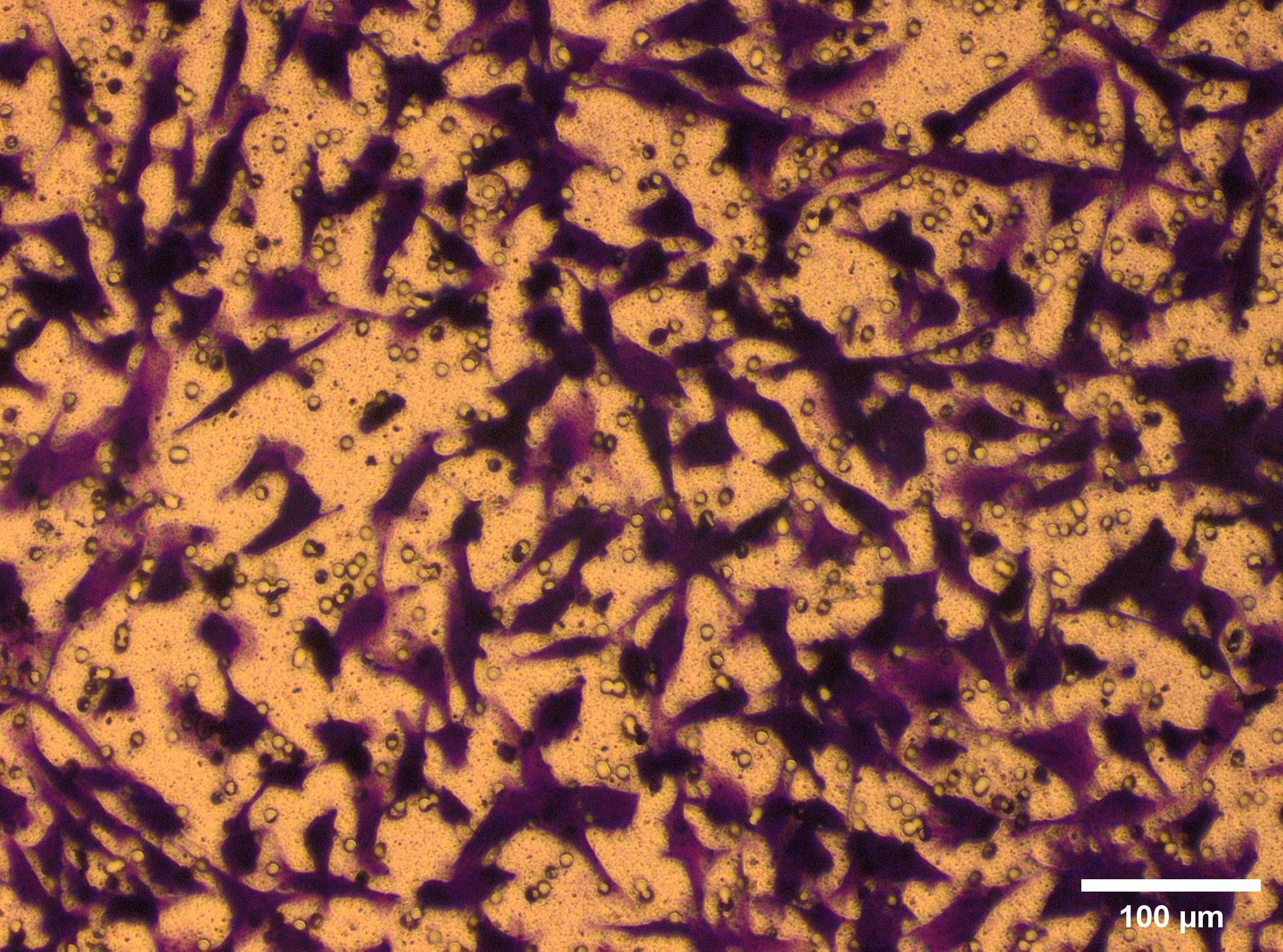

Supplement: Supplementary file 10 — EV Figure Source Data [file 44318_2026_766_MOESM10_ESM.zip › Figure EV2/Fig EV 2K/REV WT MIGRA.jpg]

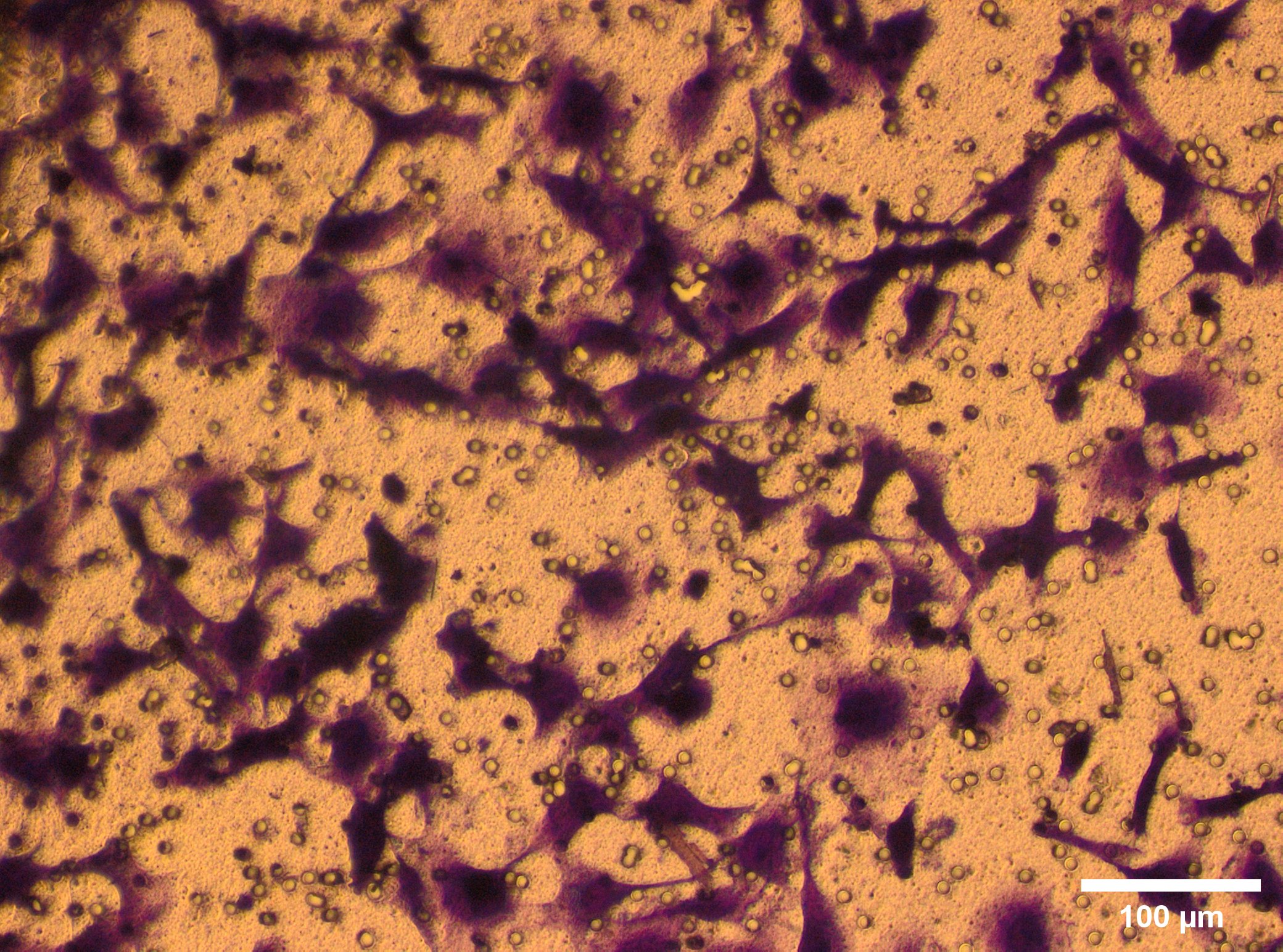

Supplement: Supplementary file 10 — EV Figure Source Data [file 44318_2026_766_MOESM10_ESM.zip › Figure EV2/Fig EV 2K/rab27a sirna dmso.jpg]

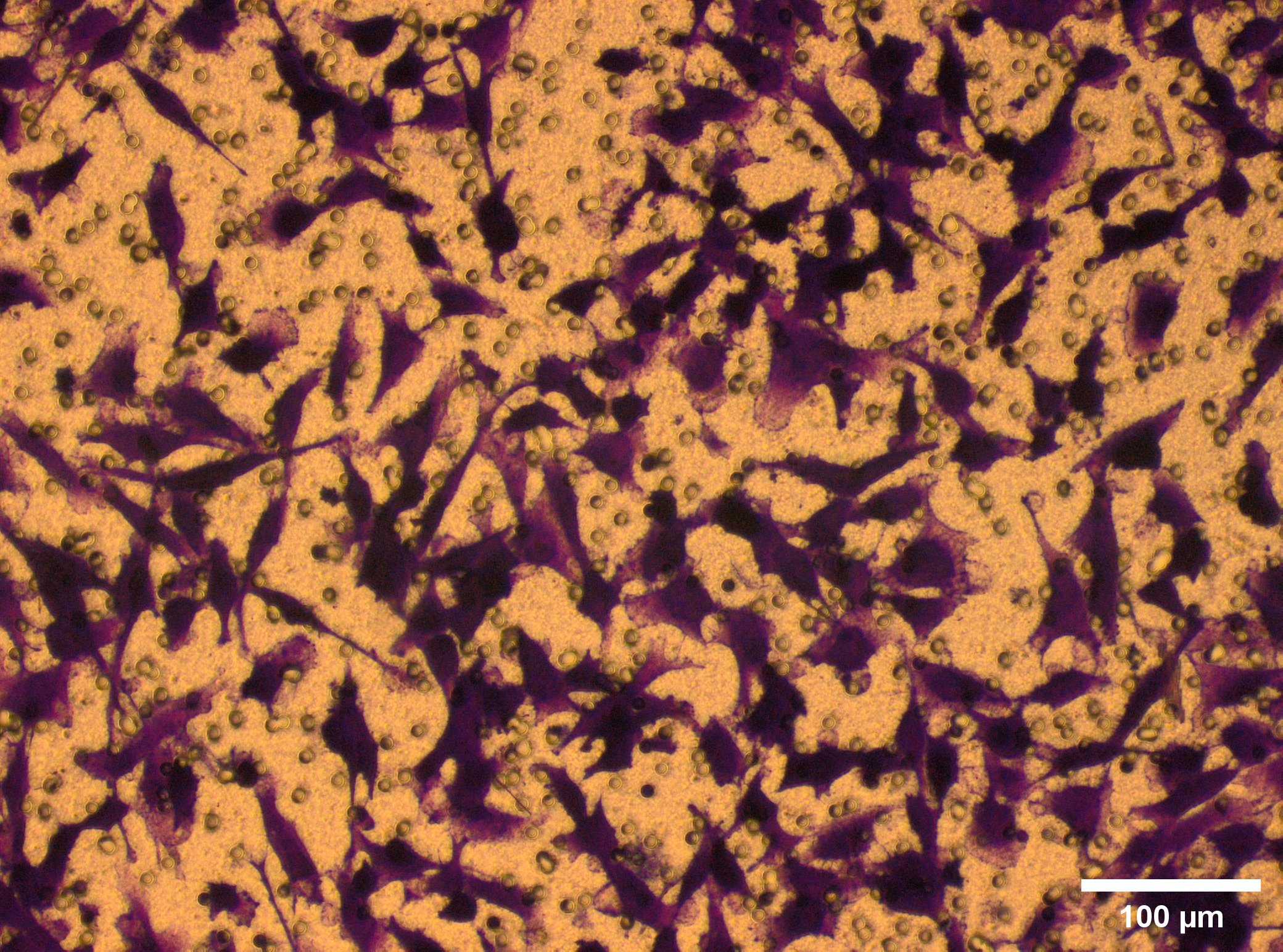

Supplement: Supplementary file 10 — EV Figure Source Data [file 44318_2026_766_MOESM10_ESM.zip › Figure EV2/Fig EV 2K/control siRNA REV migra.jpg]

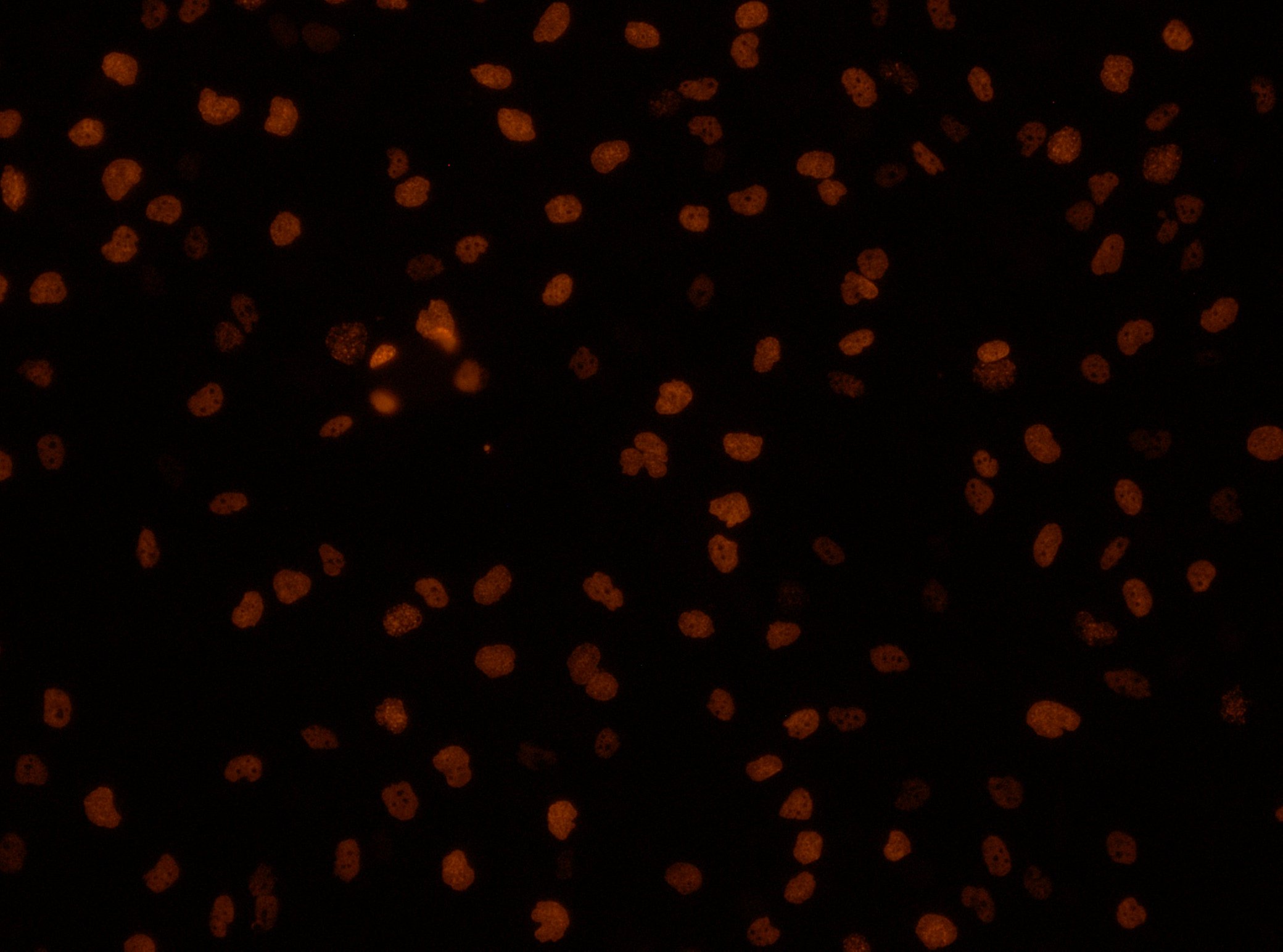

Supplement: Supplementary file 10 — EV Figure Source Data [file 44318_2026_766_MOESM10_ESM.zip › Figure EV2/Fig EV 2C/DMSO/EDU.jpg]

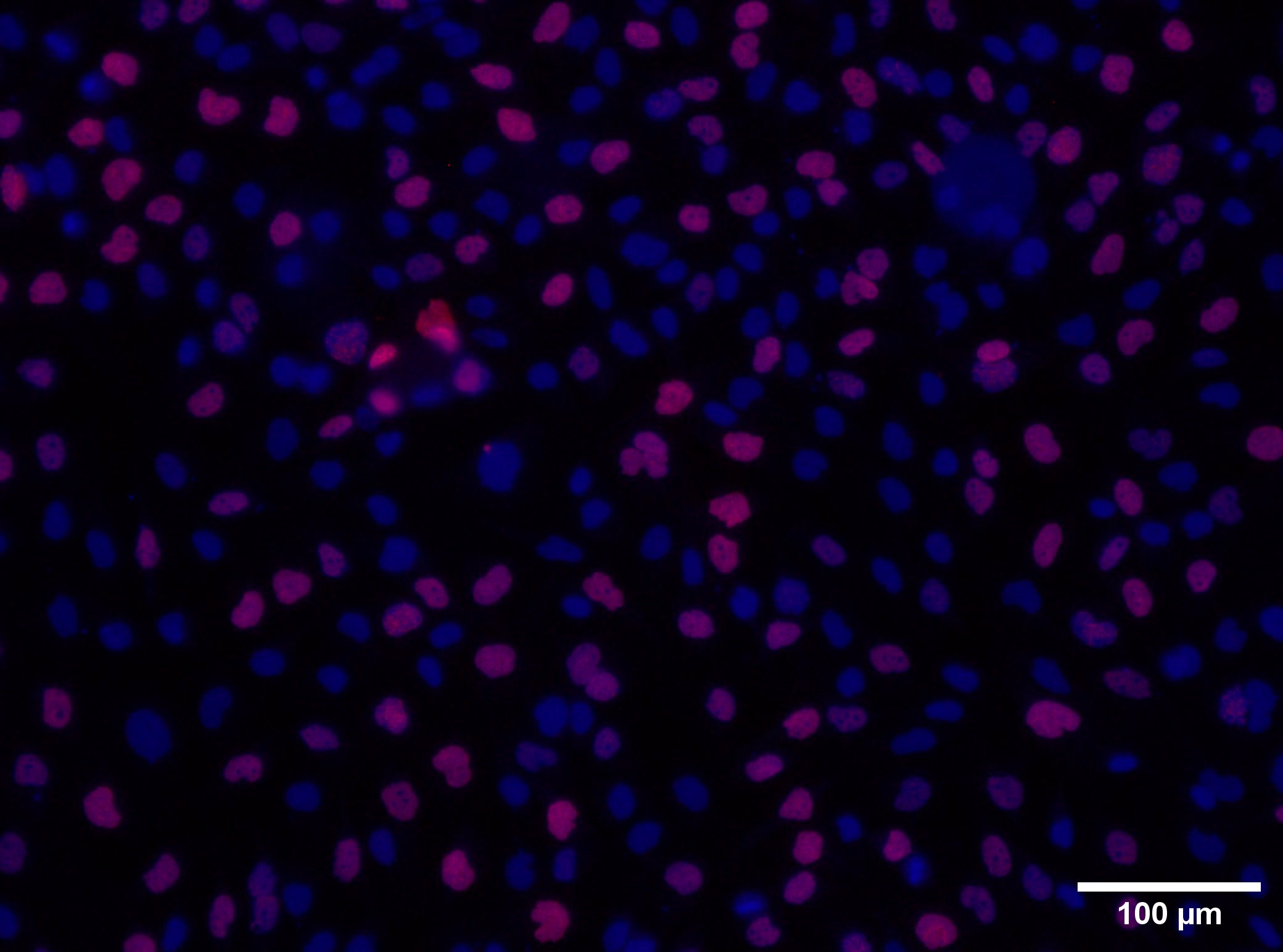

Supplement: Supplementary file 10 — EV Figure Source Data [file 44318_2026_766_MOESM10_ESM.zip › Figure EV2/Fig EV 2C/DMSO/Composite.jpg]

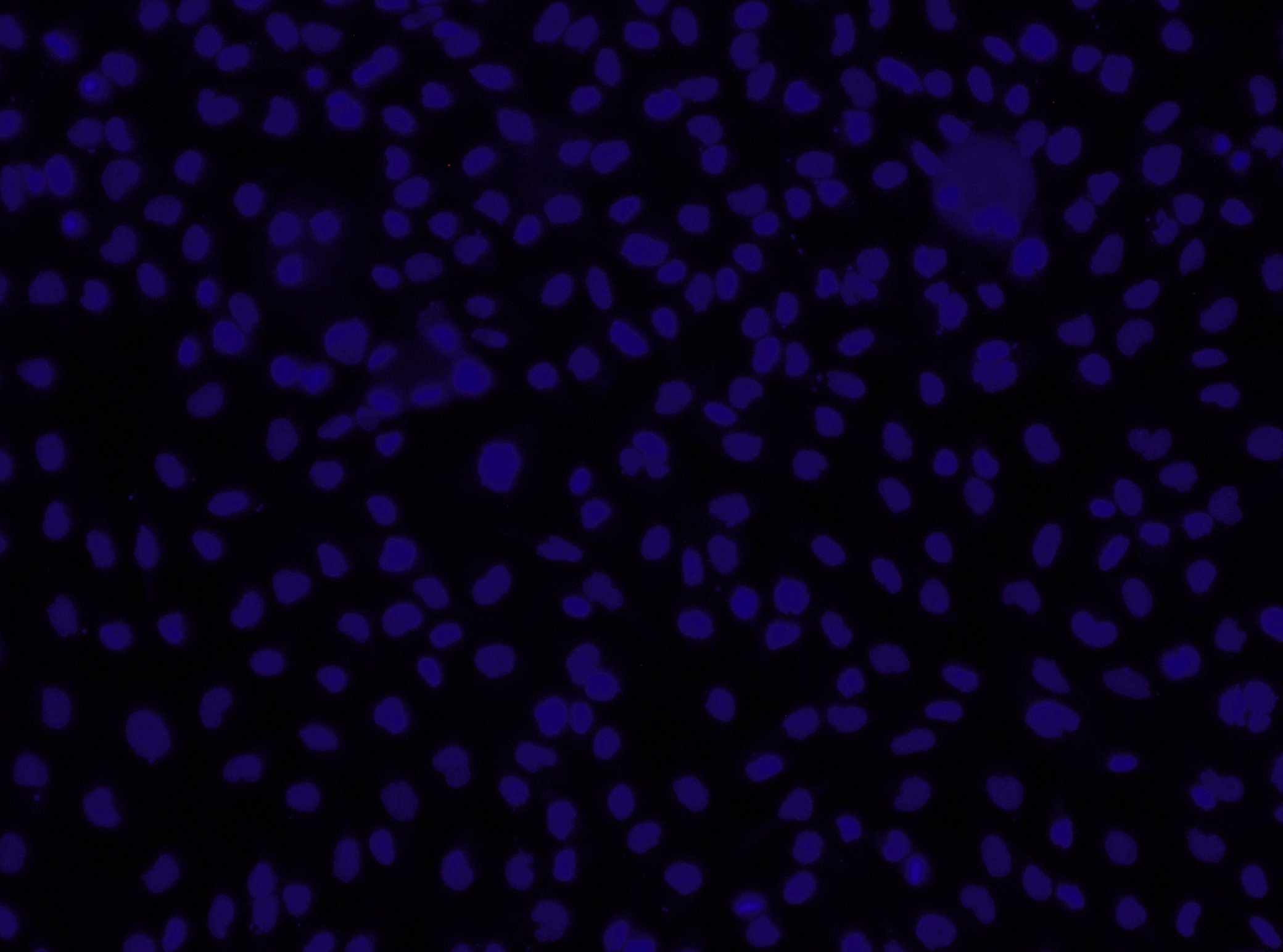

Supplement: Supplementary file 10 — EV Figure Source Data [file 44318_2026_766_MOESM10_ESM.zip › Figure EV2/Fig EV 2C/DMSO/dapi.jpg]

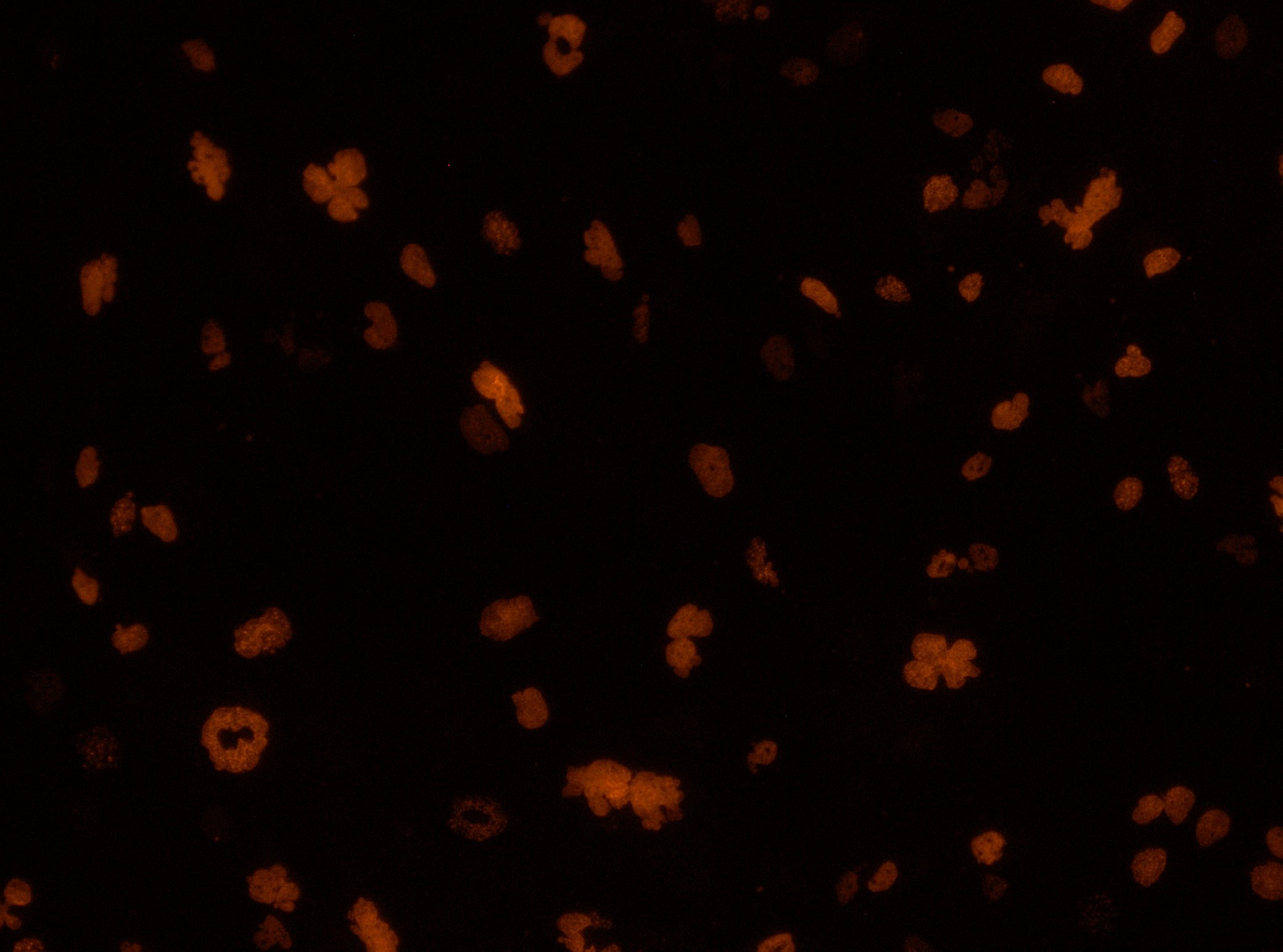

Supplement: Supplementary file 10 — EV Figure Source Data [file 44318_2026_766_MOESM10_ESM.zip › Figure EV2/Fig EV 2C/REV/edu.jpg]

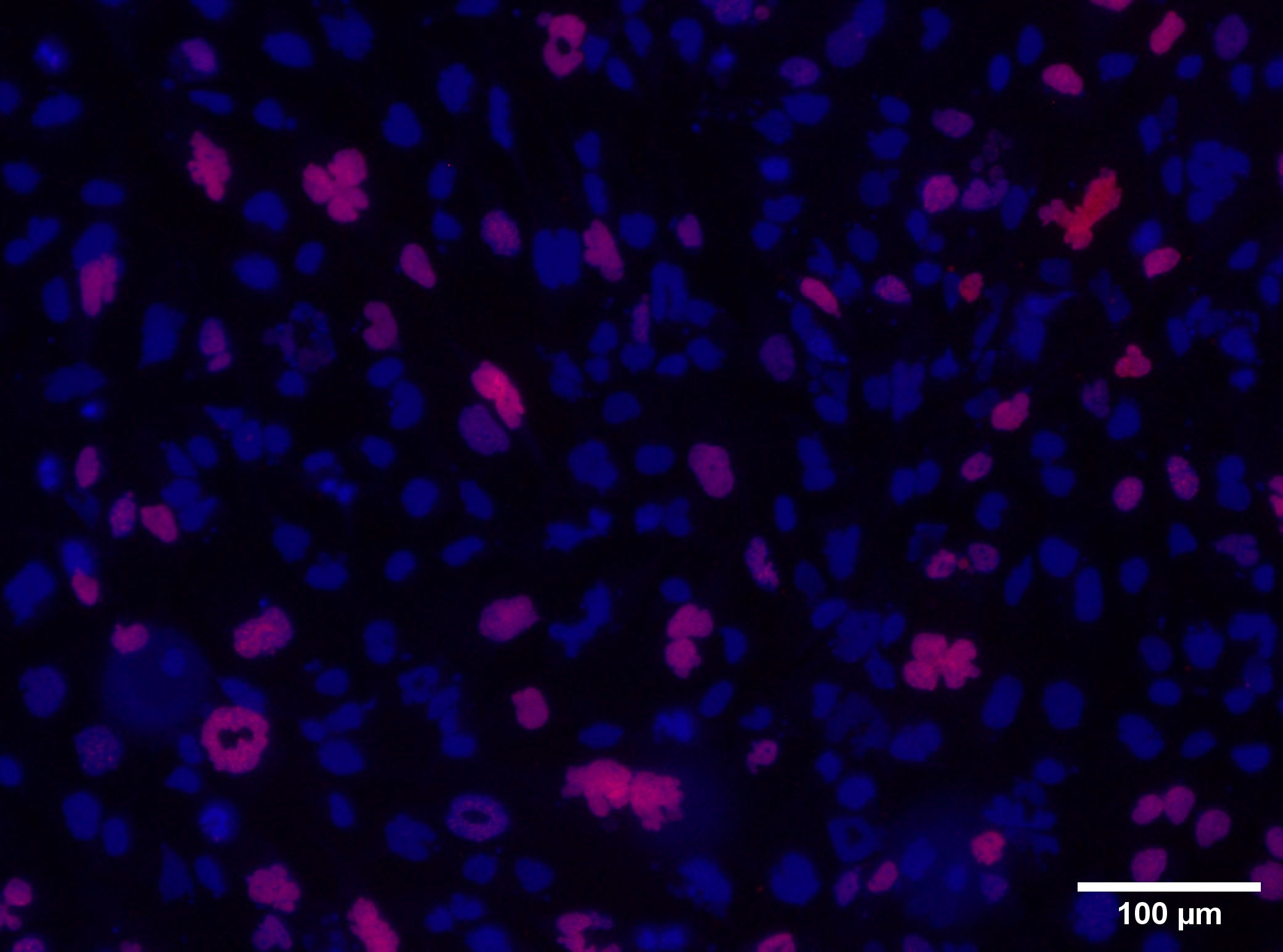

Supplement: Supplementary file 10 — EV Figure Source Data [file 44318_2026_766_MOESM10_ESM.zip › Figure EV2/Fig EV 2C/REV/Composite.jpg]

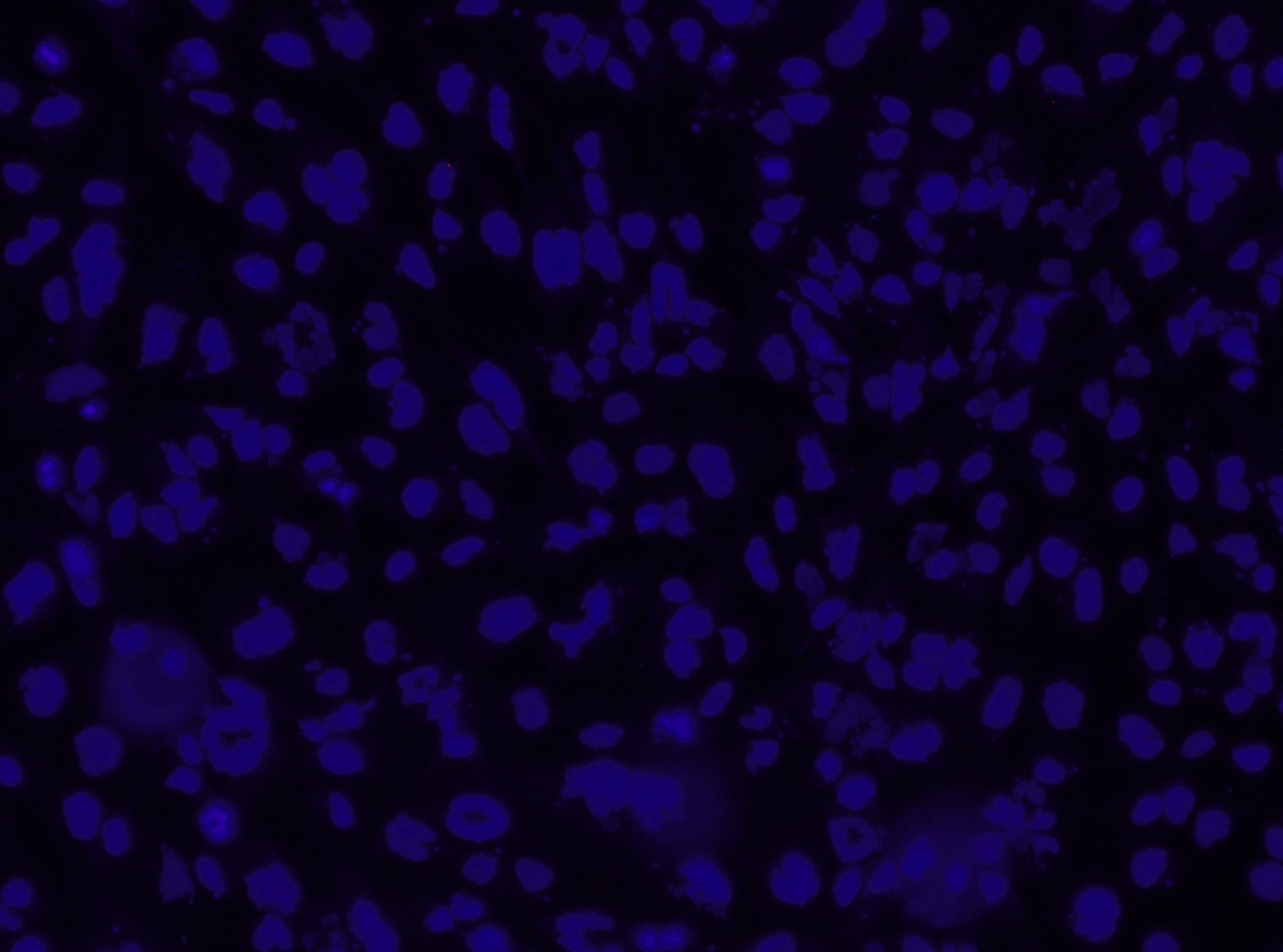

Supplement: Supplementary file 10 — EV Figure Source Data [file 44318_2026_766_MOESM10_ESM.zip › Figure EV2/Fig EV 2C/REV/dapi.jpg]

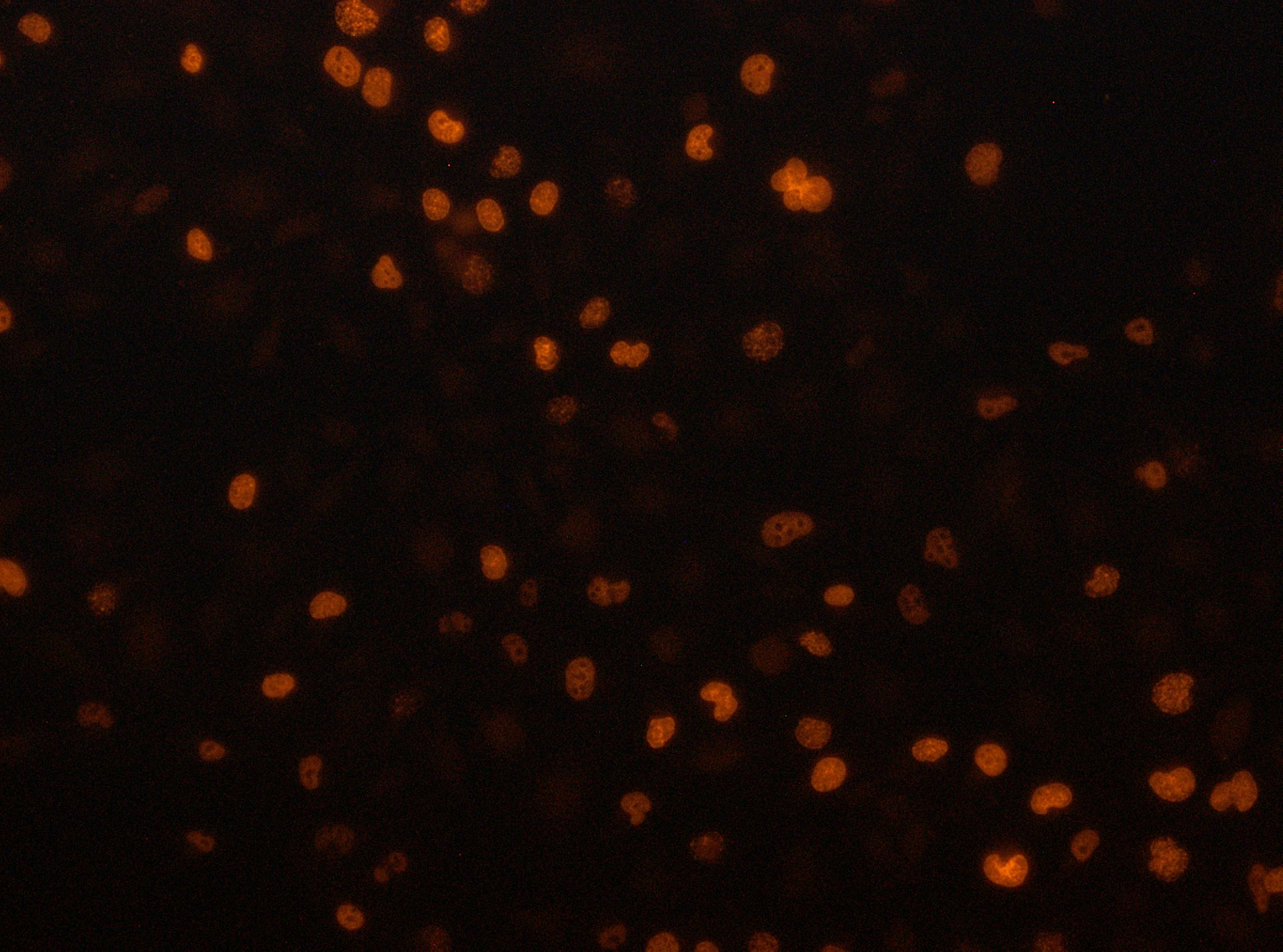

Supplement: Supplementary file 10 — EV Figure Source Data [file 44318_2026_766_MOESM10_ESM.zip › Figure EV2/Fig EV 2E/dmso/edu.jpg]

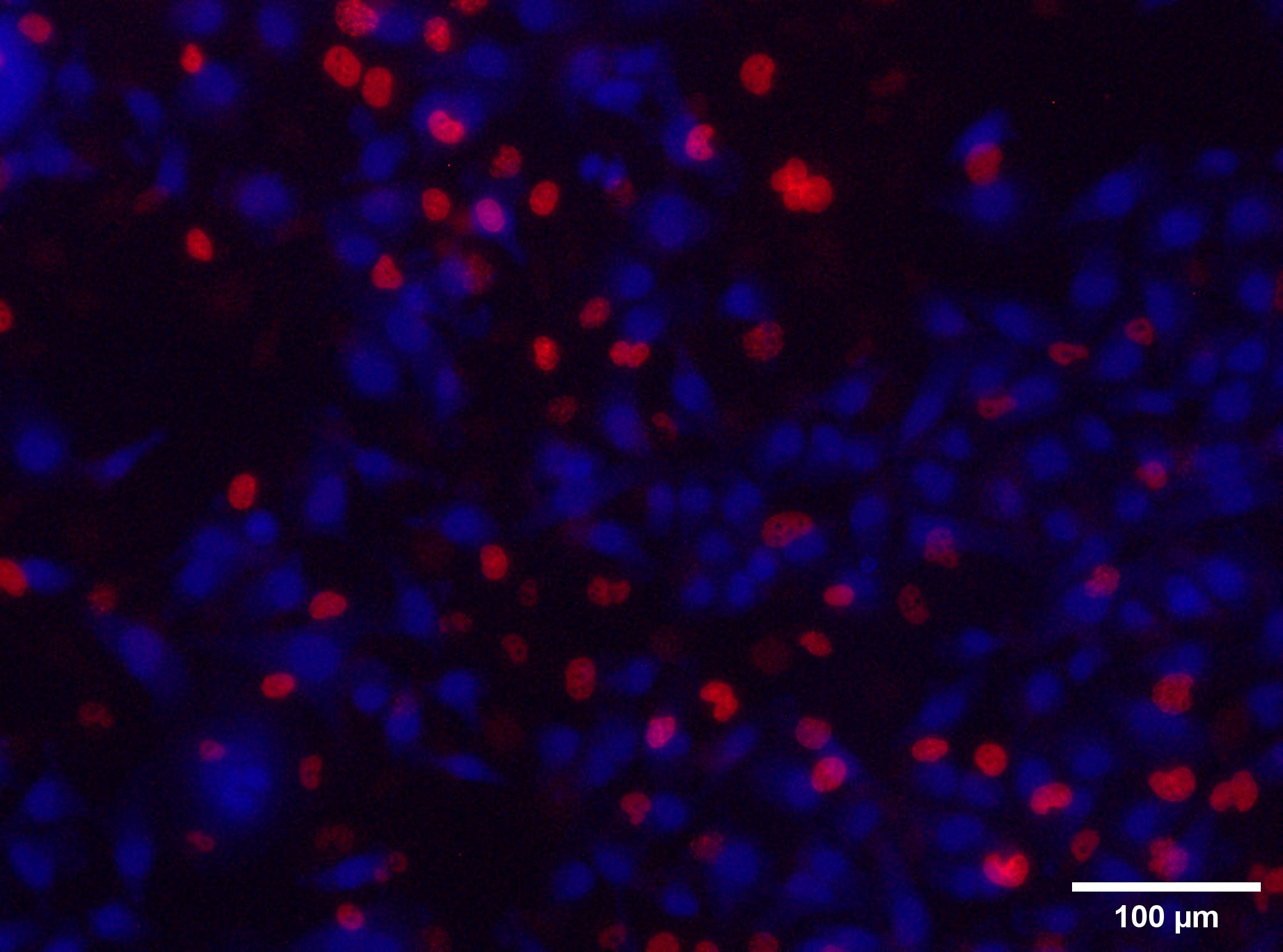

Supplement: Supplementary file 10 — EV Figure Source Data [file 44318_2026_766_MOESM10_ESM.zip › Figure EV2/Fig EV 2E/dmso/Composite.jpg]

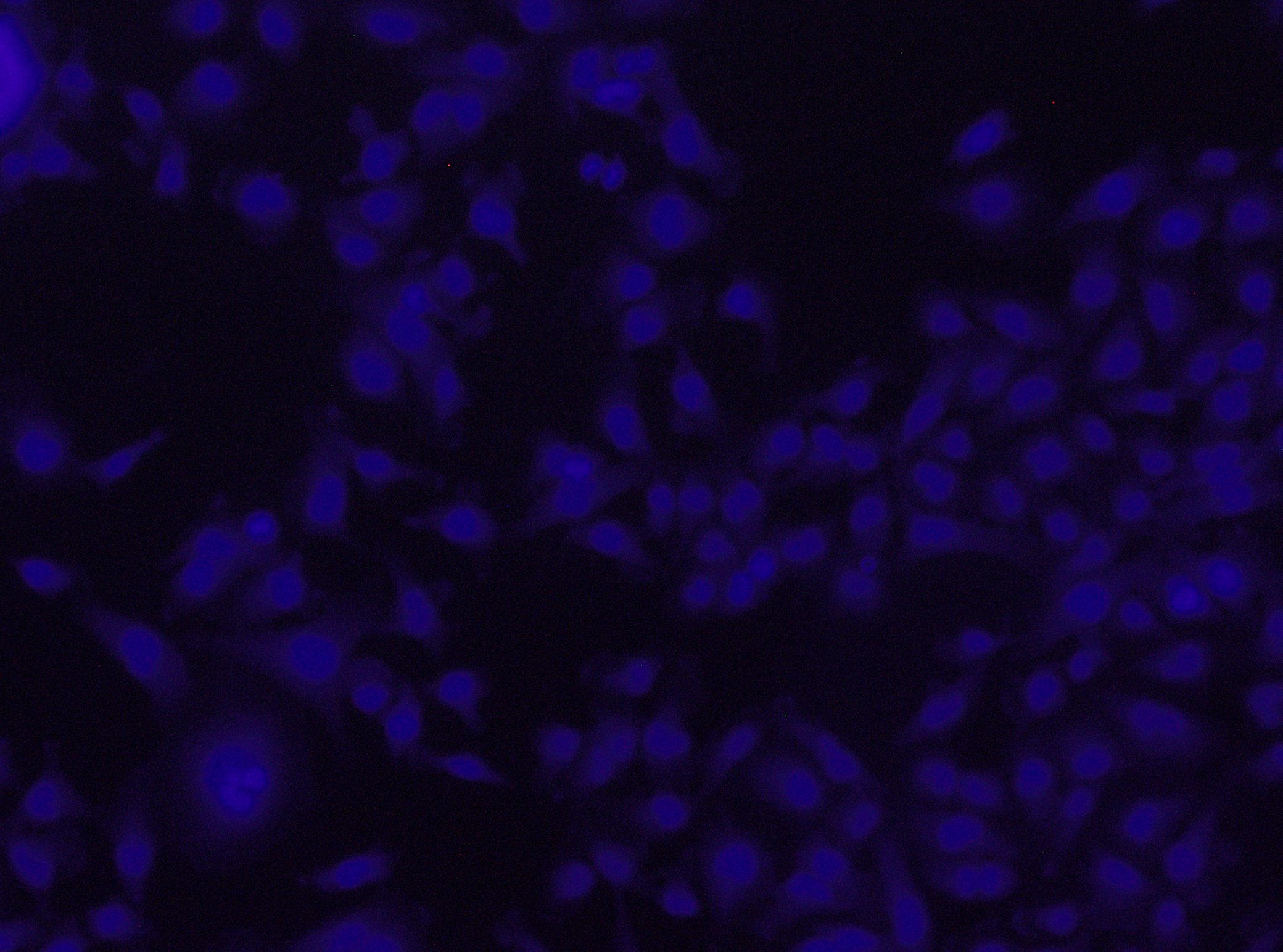

Supplement: Supplementary file 10 — EV Figure Source Data [file 44318_2026_766_MOESM10_ESM.zip › Figure EV2/Fig EV 2E/dmso/dapi.jpg]

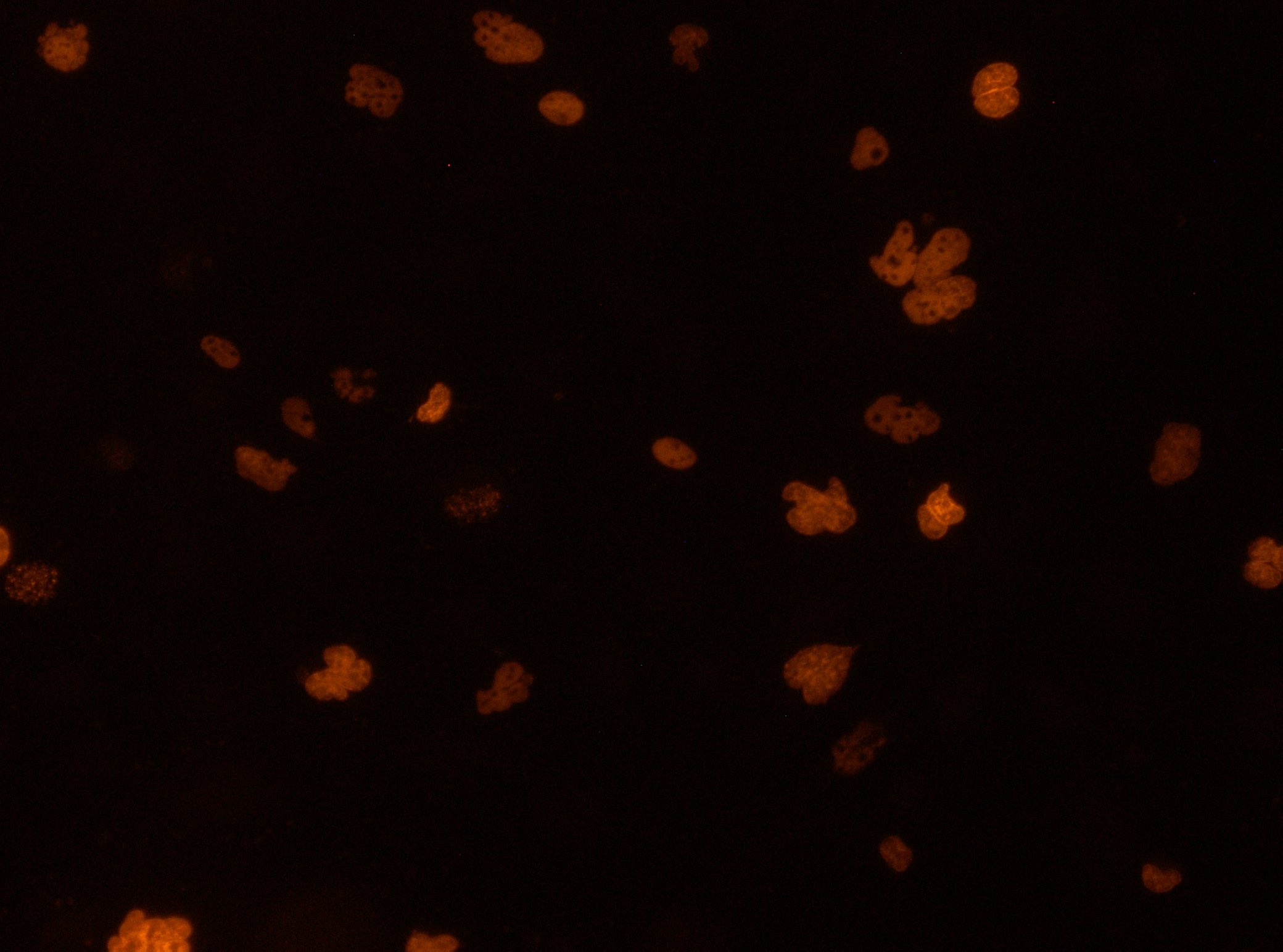

Supplement: Supplementary file 10 — EV Figure Source Data [file 44318_2026_766_MOESM10_ESM.zip › Figure EV2/Fig EV 2E/rev/edu.jpg]

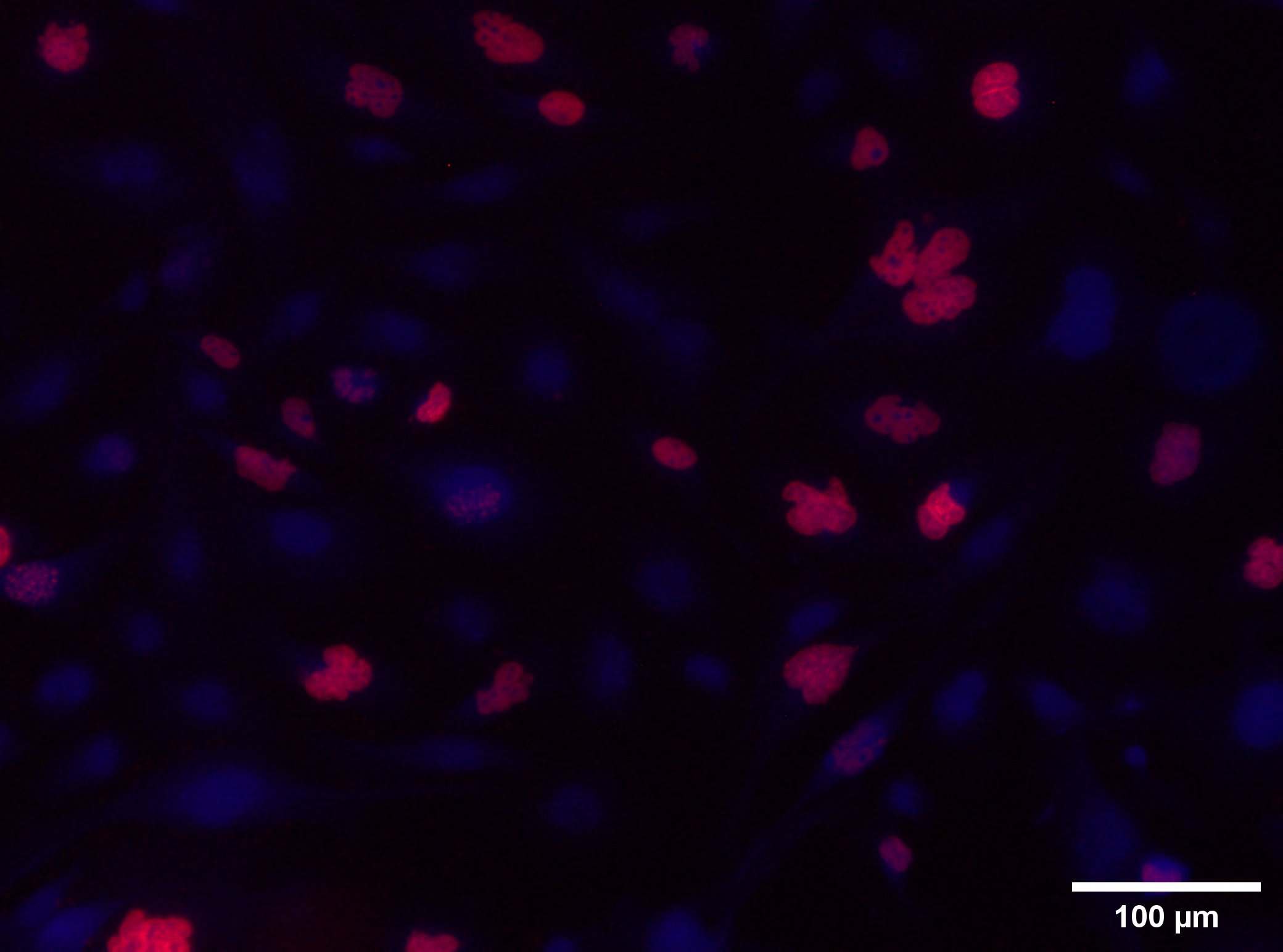

Supplement: Supplementary file 10 — EV Figure Source Data [file 44318_2026_766_MOESM10_ESM.zip › Figure EV2/Fig EV 2E/rev/Composite.jpg]

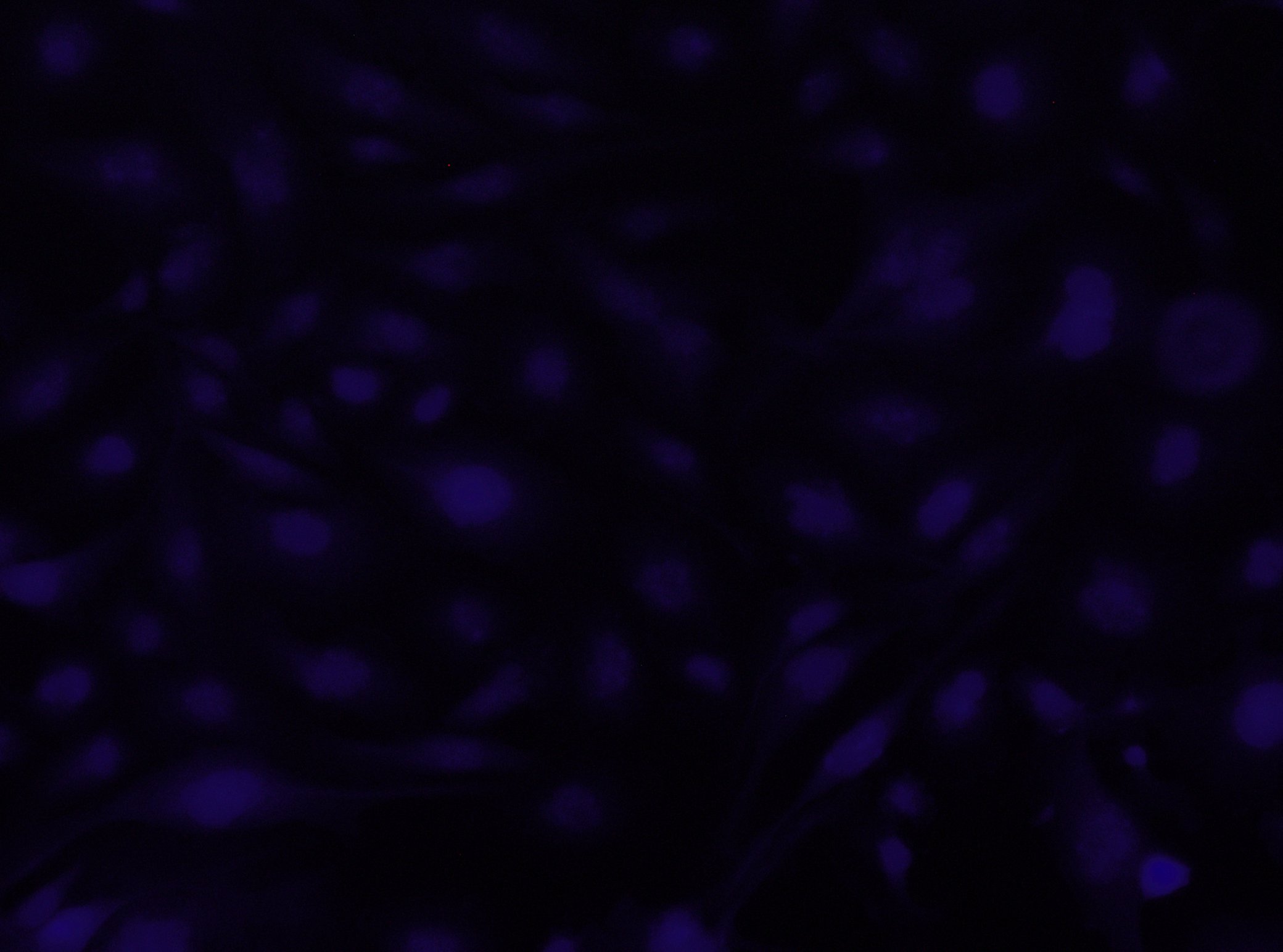

Supplement: Supplementary file 10 — EV Figure Source Data [file 44318_2026_766_MOESM10_ESM.zip › Figure EV2/Fig EV 2E/rev/dapi.jpg]

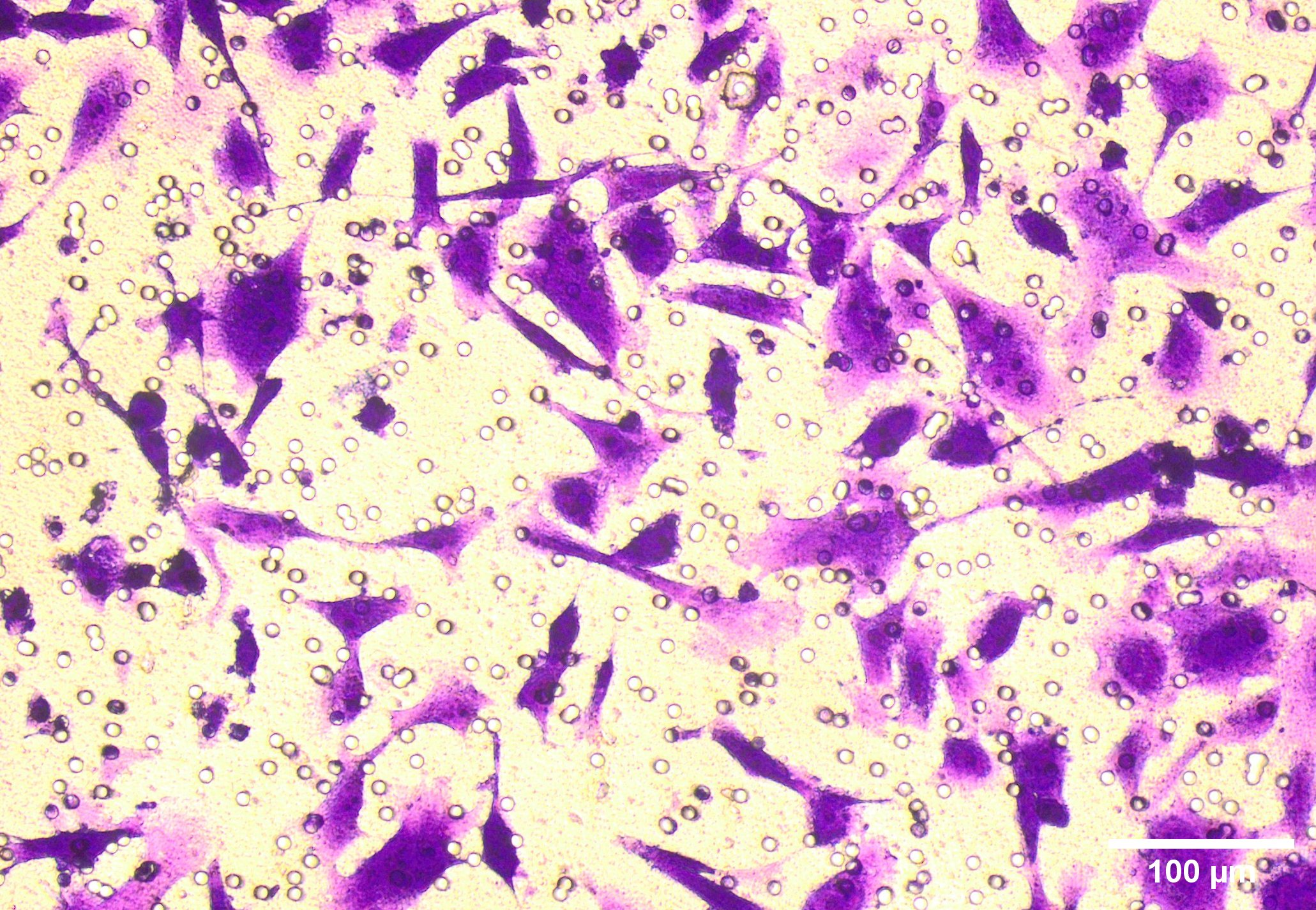

Supplement: Supplementary file 10 — EV Figure Source Data [file 44318_2026_766_MOESM10_ESM.zip › Figure EV2/Fig EV 2G/invasion/rev.jpg]
