# Supplementary material for: Chromosomal instability promotes cell migration and invasion via EFEMP1 secretion into extracellular vesicles
Source: EMBO J. 2026 Apr 13;45(10):3471–99. doi: 10.1038/s44318-026-00766-4 (PMC13187162; doi:10.1038/s44318-026-00766-4)
Supplement: Supplementary file 10 — EV Figure Source Data [file 44318_2026_766_MOESM10_ESM.zip › Figure EV7/Fig EV 7A/efemp1.pdf]

UCSC Genome Browser on Human (GRCh38/hg38)

Move <<< << < > >> >>> Zoom in 1.5x 3x 10x Base Zoom out 1.5x 3x 10x 100x

Multi-region chr2:55,851,836-55,903,228 51,393 bp. gene, chromosome range, search terms, help pages, see exampl Search Examples

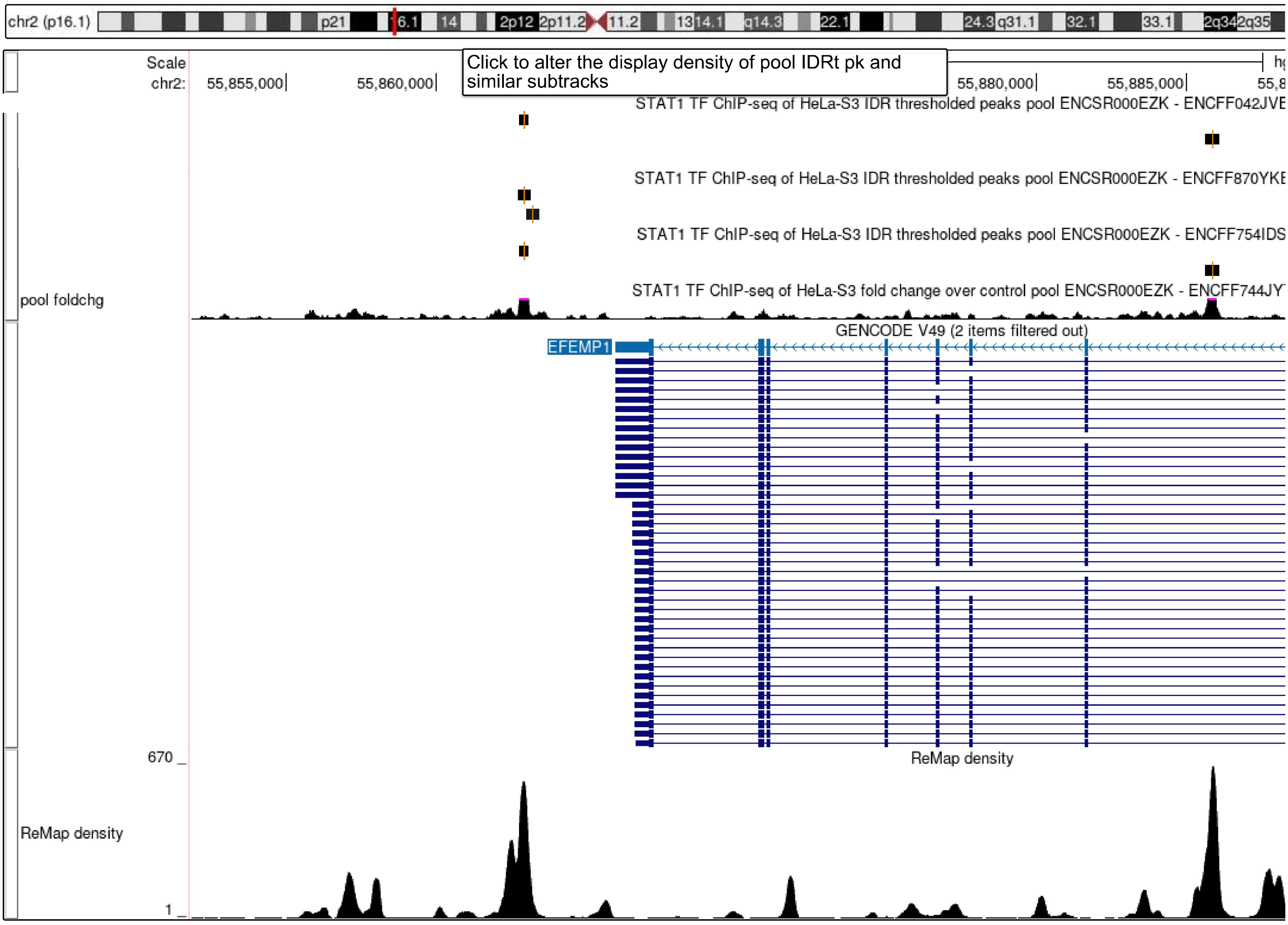

Collapse all Track search Highlight Hide all Add custom tracks Configure Reverse Resize Expand all

- Hub: Hub (TF ChIP-seq ENCSR000EZK) No Info Hide group Disconnect Refresh

STAT1 TF ChIP-seq of HeLa-S3 ENCSR000EZK full

- Mapping and Sequencing Hide group Refresh

Base Position dense | Assembly hide | Assembly Tracks hide | Centromeres hide | Chromosome Band hide | Clone Ends hide | Exome Probesets hide | Gap hide

GC Percent hide | GRC Incident hide | P14 GRC Patches pack | Hg19 Diff hide | INSDC hide | LiftOver & ReMap hide | LRG Regions hide | Mappability hide

Updated Problematic Regions show | Recomb Rate hide | RefSeq Acc hide | Restr Enzymes hide | Short Match hide

- Genes and Gene Predictions Hide group Refresh

Updated GENCODE V49 pack | NCBI RefSeq hide | CCDS hide | CRISPR Targets hide | GENCODE Archive hide | Updated GENCODE Versions hide | Updated HGNC hide | 19 IKMC Genes Mapped hide

LRG Transcripts hide | MANE hide | MGC/ORFeome Genes hide | Non-coding RNA hide | Other RefSeq hide | Pfam in GENCODE hide | Prediction Archive hide | Pseudogenes hide

RetroGenes V9 hide | TransMap V5 hide | UCSC Alt Events hide | UniProt hide

- Phenotypes, Variants, and Literature Hide group Refresh

OMIM show | AlphaMissense hide | CADD 1.6 hide | CADD 1.7 hide | Cancer Gene Expr hide | CIViC hide | ClinGen hide | ClinGen CNVs hide

ClinVar Variants hide | Constraint scores hide | Coriell CNVs hide | COSMIC hide | COSMIC Regions hide | COVID Data hide | DECIPHER hide | New Deleteriousness Predictions hide

Development Delay hide | Dosage Sensitivity hide | New G2P Project hide | GenCC hide | Gene Interactions hide | GeneReviews hide | GWAS Catalog hide | HGMD public hide

LOVD Variants hide | MITOMAP [No data-chr2] | Orphanet hid | Updated | | SNPedia | Updated Splicing Impact hide | TCGA Pan-Cancer hide

Click to alter the display density of pool IDRt pk and similar subtracks

UniProt Variants hide | Updated Variants in Papers hide

Variation

Hide group Refresh

dbSNP 155 hide | 1000 Genomes hide | Ancient Hominids hide | Updated Array Probesets hide | dbSNP Archive hide | dbVar Common Struct Var hide | DGV Struct Var hide | Genome In a Bottle hide

gnomAD Variants hide | New Long-read Variants hide | Platinum Genomes hide

Human Pangenome - HPRC

Hide group Refresh

Multiple Alignment hide | Pairwise Alignments hide | Rearrangements hide | Short Variants hide

RNA and Transcriptome

Hide group Refresh

Human ESTs hide | Human mRNAs hide | New Long-read Transcripts hide | Other ESTs hide | Other mRNAs hide | SIB Alt-Splicing hide | Spliced ESTs hide

Expression

Hide group Refresh

GTEx Gene V8 pack | GTEx RNA-Seq Coverage hide | Affy Archive hide | EPDnew Promoters hide | GNF Atlas 2 hide | GTEx Gene hide | GTEx Transcript hide | GWIPS-viz Riboseq hide

New MaveDB Experiments hide | miRNA Tissue Atlas hide | Single Cell Expression hide

Single Cell RNA-seq

Hide group Refresh

Regulation

Hide group Refresh

ENCODE cCREs hide | ENCODE Regulation hide | CpG Islands hide | FANTOM5 hide | GeneHancer hide | GTEx cis-eQTLs hide | Hi-C and Micro-C hide | JASPAR Transcription Factors hide

ORegAnno hide | RefSeq Func Elems hide | ReMap ChIP-seq full | VISTA Enhancers full

Comparative Genomics

Hide group Refresh

UCSC 100 Vertebrates hide | Zoonomia 241 Placent hide | UCSC 30 Primates hide | Primate Chain/Net hide | Placental Chain/Net hide | Vertebrate Chain/Net hide | CHM13 alignments hide | Hiller Lab 470 Mammals hide

New Unusually Conserved hide | Zoonomia+Primates 447 hide

Repeats

Hide group Refresh

RepeatMasker hide | Interrupted Rpts hide | Microsatellite hide | NuMTs Sequence hide | RepeatMasker Viz. hide | Segmental Dups hide | Self Alignment hide | Simple Repeats hide

WM + SDust hide

Refresh
