# Supplementary material for: Chromosomal instability promotes cell migration and invasion via EFEMP1 secretion into extracellular vesicles
Source: EMBO J. 2026 Apr 13;45(10):3471–99. doi: 10.1038/s44318-026-00766-4 (PMC13187162; doi:10.1038/s44318-026-00766-4)
Supplement: Supplementary file 10 — EV Figure Source Data [file 44318_2026_766_MOESM10_ESM.zip › Figure EV7/Fig EV 7C/ACTB.pdf]

Genomes | Genome Browser | Tools | Mirrors | Downloads | My Data | View | Help | About Us

UCSC Genome Browser on Human (GRCh38/hg38)

Move <<< << < > >> >>> Zoom in 1.5x 3x 10x Base Zoom out 1.5x 3x 10x 100x

Multi-region chr7:5,526,776-5,532,021 5,246 bp. gene, chromosome range, search terms, help pages, see exampl Search Examples

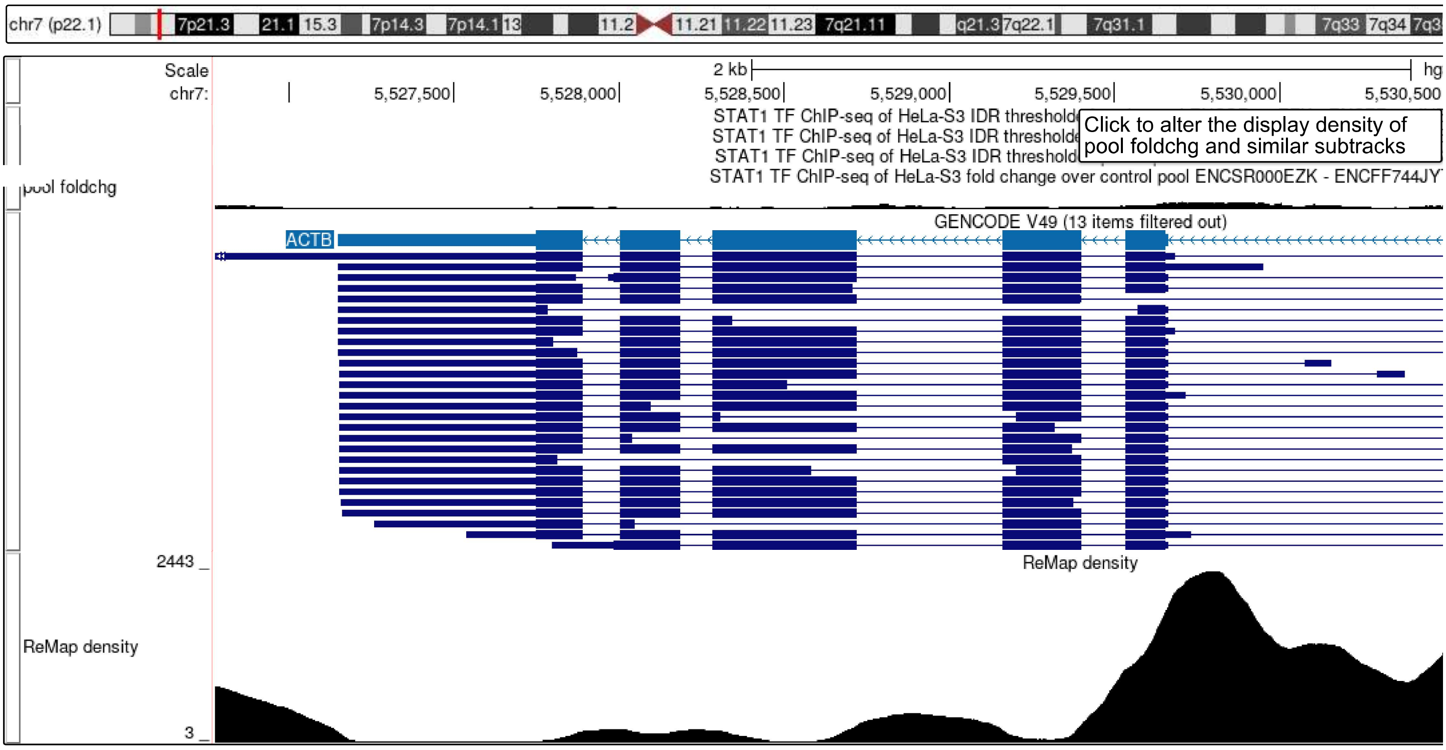

Collapse all | Track search | Highlight | Hide all | Add custom tracks | Configure | Reverse | Resize | Expand all

Hub: Hub (TF ChIP-seq ENCSR000EZK)

No Info | Hide group | Disconnect | Refresh

- STAT1 TF ChIP-seq of HeLa-S3 ENCSR000EZK — full

Mapping and Sequencing

Hide group | Refresh

- Base Position — dense
- Assembly — hide
- Assembly Tracks — hide
- Centromeres — hide
- Chromosome Band — hide
- Clone Ends — hide
- Exome Probesets — hide
- Gap — hide
- GC Percent — hide
- GRC Incident — hide
- P14 GRC Patches — pack
- Hg19 Diff — hide
- INSDC — hide
- LiftOver & ReMap — hide
- LRG Regions — hide
- Mappability — hide
- Updated Problematic Regions — show
- Recomb Rate — hide
- RefSeq Acc — hide
- Restr Enzymes — hide
- Short Match — hide

Genes and Gene Predictions

Hide group | Refresh

- Updated GENCODE V49 — pack
- NCBI RefSeq — hide
- CCDS — hide
- CRISPR Targets — hide
- GENCODE Archive — hide
- Updated GENCODE Versions — hide
- Updated HGNC — hide
- 19 IKMC Genes Mapped — hide
- LRG Transcripts — hide
- MANE — hide
- MGC/ORFeome Genes — hide
- Non-coding RNA — hide
- Other RefSeq — hide
- Pfam in GENCODE — hide
- Prediction Archive — hide
- Pseudogenes — hide
- RetroGenes V9 — hide
- TransMap V5 — hide
- UCSC Alt Events — hide
- UniProt — hide

Phenotypes, Variants, and Literature

Hide group | Refresh

- OMIM — show
- AlphaMissense — hide
- CADD 1.6 — hide
- CADD 1.7 — hide
- Cancer Gene Expr — hide
- CIViC — hide
- ClinGen — hide
- ClinGen CNVs — hide
- ClinVar Variants — hide
- Constraint scores — hide
- 19 Coriell CNVs — hide
- COSMIC — hide
- COSMIC Regions — hide
- COVID Data — hide
- DECIPHER — hide
- New Deleteriousness Predictions — hide
- Development Delay — hide
- Dosage Sensitivity — hide
- New G2P Project — hide
- GenCC — hide
- Gene Interactions — hide
- GeneReviews — hide
- GWAS Catalog — hide
- HGMD public — hide
- LOVD Variants — hide
- MITOMAP — [No data-chr7]
- Orphanet — hide
- Updated PanelApp — hide
- 19 REVEL Scores — hide
- SNPedia — hide
- Updated Splicing Impact — hide
- TCGA Pan-Cancer — hide

UniProt Variants hide
Updated Variants in Papers hide

Variation

Hide group Refresh

dbSNP 155 hide | 1000 Genomes hide | Ancient Hominids hide | Updated Array Probesets hide | dbSNP Archive hide | dbVar Common Struct Var hide | DGV Struct Var hide | Genome In a Bottle hide
gnomAD Variants hide | New Long-read Variants hide | Platinum Genomes hide

Click to alter the display density of pool foldchg and similar subtracks

Human Pangenome - HPRC

Hide group Refresh

Multiple Alignment hide | Pairwise Alignments hide | Rearrangements hide | Short Variants hide

RNA and Transcriptome

Hide group Refresh

Human ESTs hide | Human mRNAs hide | New Long-read Transcripts hide | Other ESTs hide | Other mRNAs hide | SIB Alt-Splicing hide | Spliced ESTs hide

Expression

Hide group Refresh

GTEx Gene V8 hide | GTEx RNA-Seq Coverage hide | Affy Archive hide | EPDnew Promoters hide | GNF Atlas 2 hide | GTEx Gene hide | GTEx Transcript hide | GWIPS-viz Riboseq hide
New MaveDB Experiments hide | miRNA Tissue Atlas hide | Single Cell Expression hide

Single Cell RNA-seq

Hide group Refresh

Regulation

Hide group Refresh

ENCODE cCREs hide | ENCODE Regulation hide | CpG Islands hide | FANTOM5 hide | GeneHancer hide | GTEx cis-eQTLs hide | Hi-C and Micro-C hide | JASPAR Transcription Factors hide
ORegAnno hide | RefSeq Func Elems hide | ReMap ChIP-seq full | VISTA Enhancers hide

Comparative Genomics

Hide group Refresh

UCSC 100 Vertebrates hide | Zoonomia 241 Placent hide | UCSC 30 Primates hide | Primate Chain/Net hide | Placental Chain/Net hide | Vertebrate Chain/Net hide | CHM13 alignments hide | Hiller Lab 470 Mammals hide
New Unusually Conserved hide | Zoonomia+Primates 447 hide

Repeats

Hide group Refresh

RepeatMasker hide | Interrupted Rpts hide | Microsatellite hide | NuMTs Sequence hide | RepeatMasker Viz. hide | Segmental Dups hide | Self Alignment hide | Simple Repeats hide
WM + SDust hide

Refresh
