# Supplementary material for: Chromosomal instability promotes cell migration and invasion via EFEMP1 secretion into extracellular vesicles
Source: EMBO J. 2026 Apr 13;45(10):3471–99. doi: 10.1038/s44318-026-00766-4 (PMC13187162; doi:10.1038/s44318-026-00766-4)
Supplement: Supplementary file 10 — EV Figure Source Data [file 44318_2026_766_MOESM10_ESM.zip › Figure EV7/Fig EV 7B/b2m.pdf]

UCSC Genome Browser on Human (GRCh38/hg38)

Move <<< << < > >> >>> Zoom in 1.5x 3x 10x Base Zoom out 1.5x 3x 10x 100x

Multi-region chr15:44,703,892-44,733,722 29,831 bp. gene, chromosome range, search terms, help pages, see exampl Search Examples

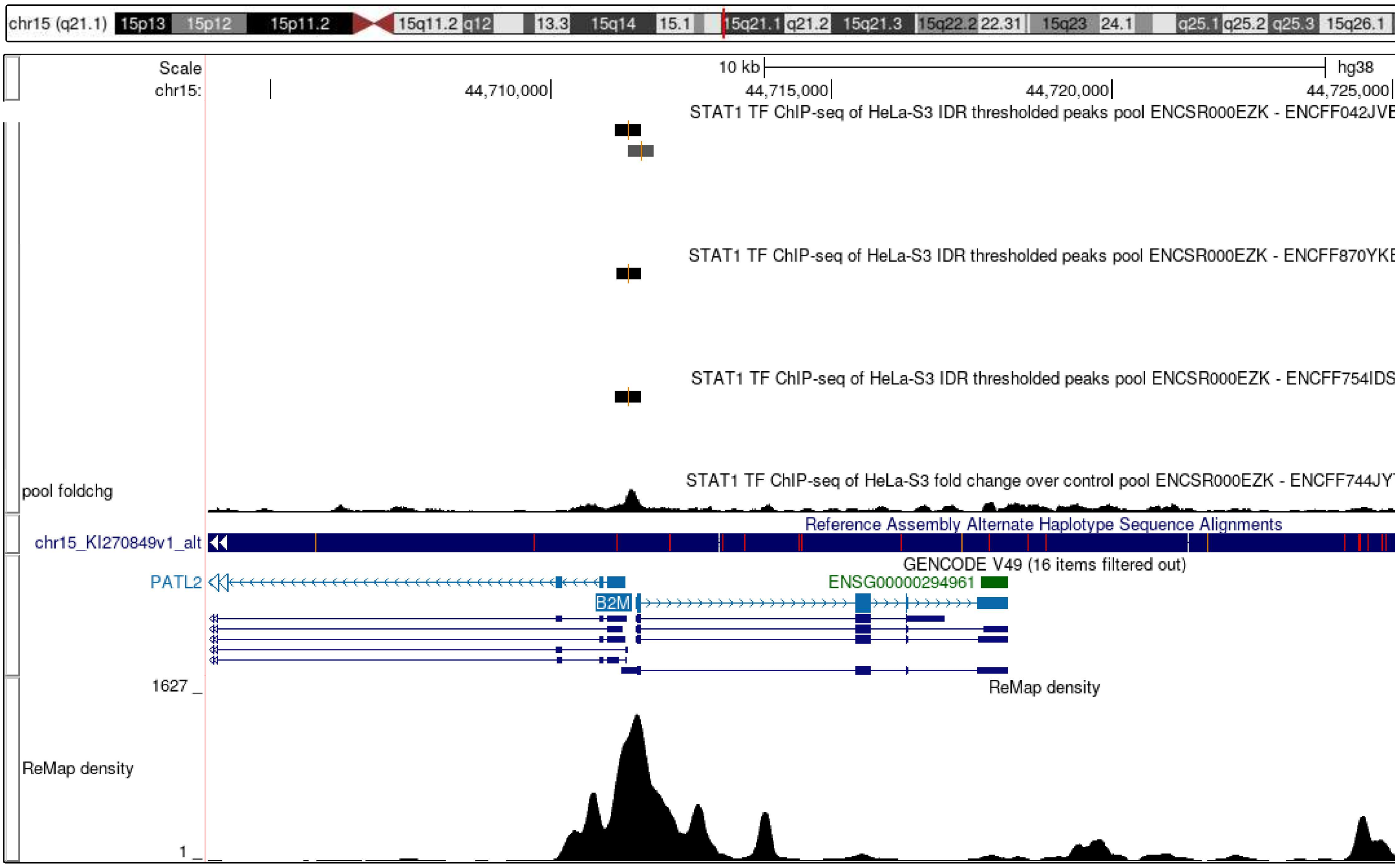

Collapse all | Track search | Highlight | Hide all | Add custom tracks | Configure | Reverse | Resize | Expand all

Hub: Hub (TF ChIP-seq ENCSR000EZK) No Info | Hide group | Disconnect | Refresh

STAT1 TF ChIP-seq of HeLa-S3 ENCSR000EZK — full

Mapping and Sequencing Hide group | Refresh

- Base Position — dense
- Assembly — hide
- Assembly Tracks — hide
- Centromeres — hide
- Chromosome Band — hide
- Clone Ends — hide
- Exome Probesets — hide
- Gap — hide
- GC Percent — hide
- GRC Incident — hide
- P14 GRC Patches — pack
- Hg19 Diff — hide
- INSDC — hide
- LiftOver & ReMap — hide
- LRG Regions — hide
- Mappability — hide
- Updated Problematic Regions — show
- Recomb Rate — hide
- RefSeq Acc — hide
- Restr Enzymes — hide
- Short Match — hide

Genes and Gene Predictions Hide group | Refresh

- Updated GENCODE V49 — pack
- NCBI RefSeq — hide
- CCDS — hide
- CRISPR Targets — hide
- GENCODE Archive — hide
- Updated GENCODE Versions — hide
- Updated HGNC — hide
- 19 IKMC Genes Mapped — hide
- LRG Transcripts — hide
- MANE — hide
- MGC/ORFeome Genes — hide
- Non-coding RNA — hide
- Other RefSeq — hide
- Pfam in GENCODE — hide
- Prediction Archive — hide
- Pseudogenes — hide
- RetroGenes V9 — hide
- TransMap V5 — hide
- UCSC Alt Events — hide
- UniProt — hide

Phenotypes, Variants, and Literature Hide group | Refresh

- OMIM — show
- AlphaMissense — hide
- CADD 1.6 — hide
- CADD 1.7 — hide
- Cancer Gene Expr — hide
- CIViC — hide
- ClinGen — hide
- ClinGen CNVs — hide
- ClinVar Variants — hide
- Constraint scores — hide
- 19 Coriell CNVs — hide
- COSMIC — hide
- COSMIC Regions — hide
- COVID Data — hide
- DECIPHER — hide
- New Deleteriousness Predictions — hide
- Development Delay — hide
- Dosage Sensitivity
- New G2P Project
- GenCC — hide
- Gene Interactions
- GeneReviews — hide
- GWAS Catalog
- HGMD public — hide

hide | hide | hide | hide

LOVD Variants hide
MITOMAP [No data-chr15]
Orphanet hide
Updated PanelApp hide
REVEL Scores hide
SNPedia hide
Updated Splicing Impact hide
TCGA Pan-Cancer hide
UniProt Variants hide
Updated Variants in Papers hide

Variation

Hide group Refresh

dbSNP 155 hide
1000 Genomes hide
Ancient Hominids hide
Updated Array Probesets hide
dbSNP Archive hide
dbVar Common Struct Var hide
DGV Struct Var hide
Genome In a Bottle hide
gnomAD Variants hide
New Long-read Variants hide
Platinum Genomes hide

Human Pangenome - HPRC

Hide group Refresh

Multiple Alignment hide
Pairwise Alignments hide
Rearrangements hide
Short Variants hide

RNA and Transcriptome

Hide group Refresh

Human ESTs hide
Human mRNAs hide
New Long-read Transcripts hide
Other ESTs hide
Other mRNAs hide
SIB Alt-Splicing hide
Spliced ESTs hide

Expression

Hide group Refresh

GTEx Gene V8 hide
GTEx RNA-Seq Coverage hide
Affy Archive hide
EPDnew Promoters hide
GNF Atlas 2 hide
GTEx Gene hide
GTEx Transcript hide
GWIPS-viz Riboseq hide
New MaveDB Experiments hide
miRNA Tissue Atlas hide
Single Cell Expression hide

Single Cell RNA-seq

Hide group Refresh

Regulation

Hide group Refresh

ENCODE cCREs hide
ENCODE Regulation hide
CpG Islands hide
FANTOM5 hide
GeneHancer hide
GTEx cis-eQTLs hide
Hi-C and Micro-C hide
JASPAR Transcription Factors hide
ORegAnno hide
RefSeq Func Elems hide
ReMap ChIP-seq full
VISTA Enhancers hide

Comparative Genomics

Hide group Refresh

UCSC 100 Vertebrates hide
Zoonomia 241 Placent hide
UCSC 30 Primates hide
Primate Chain/Net hide
Placental Chain/Net hide
Vertebrate Chain/Net hide
CHM13 alignments hide
Hiller Lab 470 Mammals hide
New Unusually Conserved hide
Zoonomia+Primates 447 hide

Repeats

Hide group Refresh

RepeatMasker hide
Interrupted Rpts hide
Microsatellite hide
NuMTs Sequence hide
RepeatMasker Viz. hide
Segmental Dups hide
Self Alignment hide
Simple Repeats hide
WM + SDust hide

Refresh
