# Supplementary figures and images for: Chromosomal instability promotes cell migration and invasion via EFEMP1 secretion into extracellular vesicles (part 2 of 2)
Source: EMBO J. 2026 Apr 13;45(10):3471–99. doi: 10.1038/s44318-026-00766-4 (PMC13187162; doi:10.1038/s44318-026-00766-4)

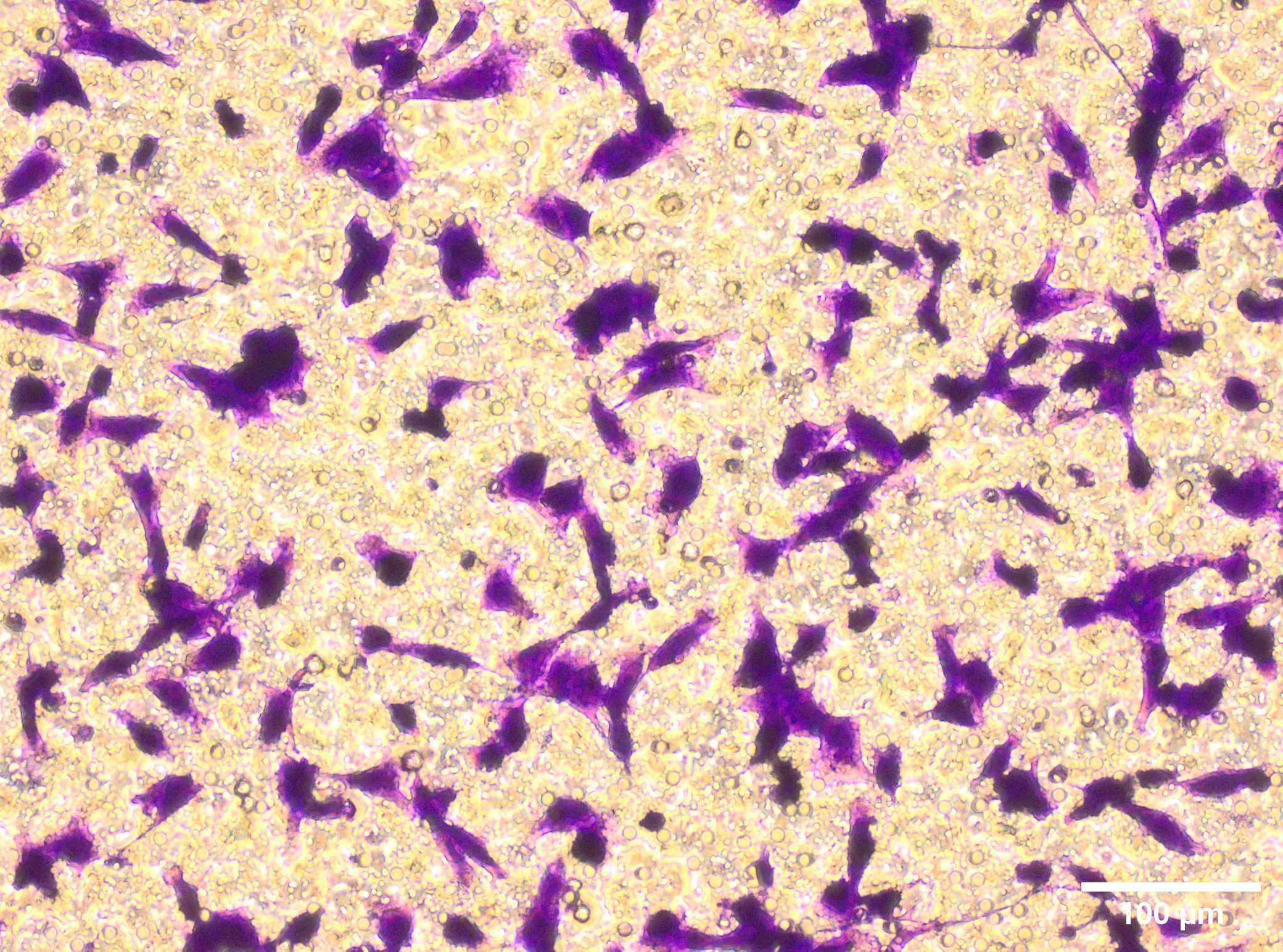

Supplement: Supplementary file 10 — EV Figure Source Data [file 44318_2026_766_MOESM10_ESM.zip › Figure EV2/Fig EV 2G/invasion/dmso.jpg]

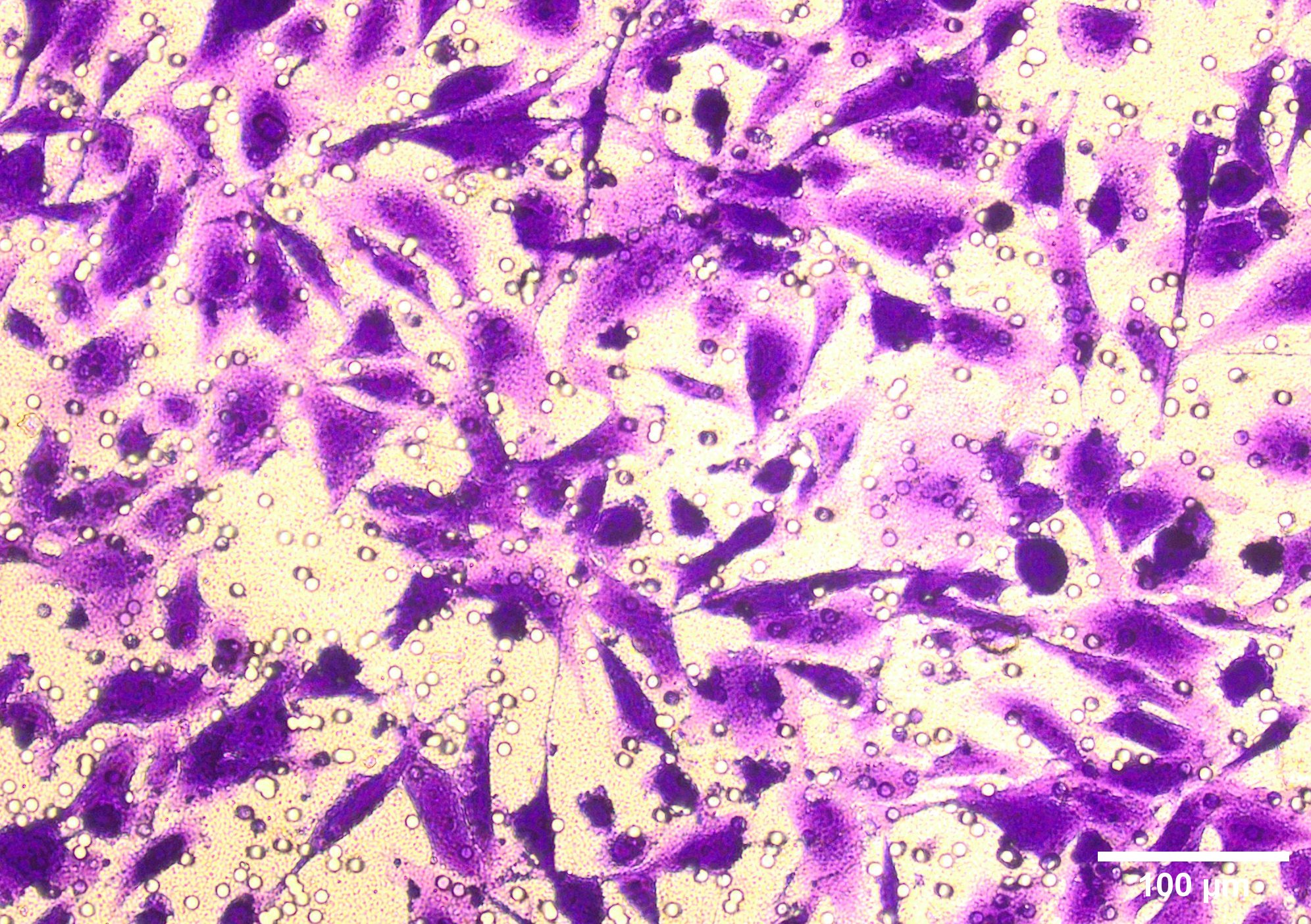

Supplement: Supplementary file 10 — EV Figure Source Data [file 44318_2026_766_MOESM10_ESM.zip › Figure EV2/Fig EV 2G/migration/rev.jpg]

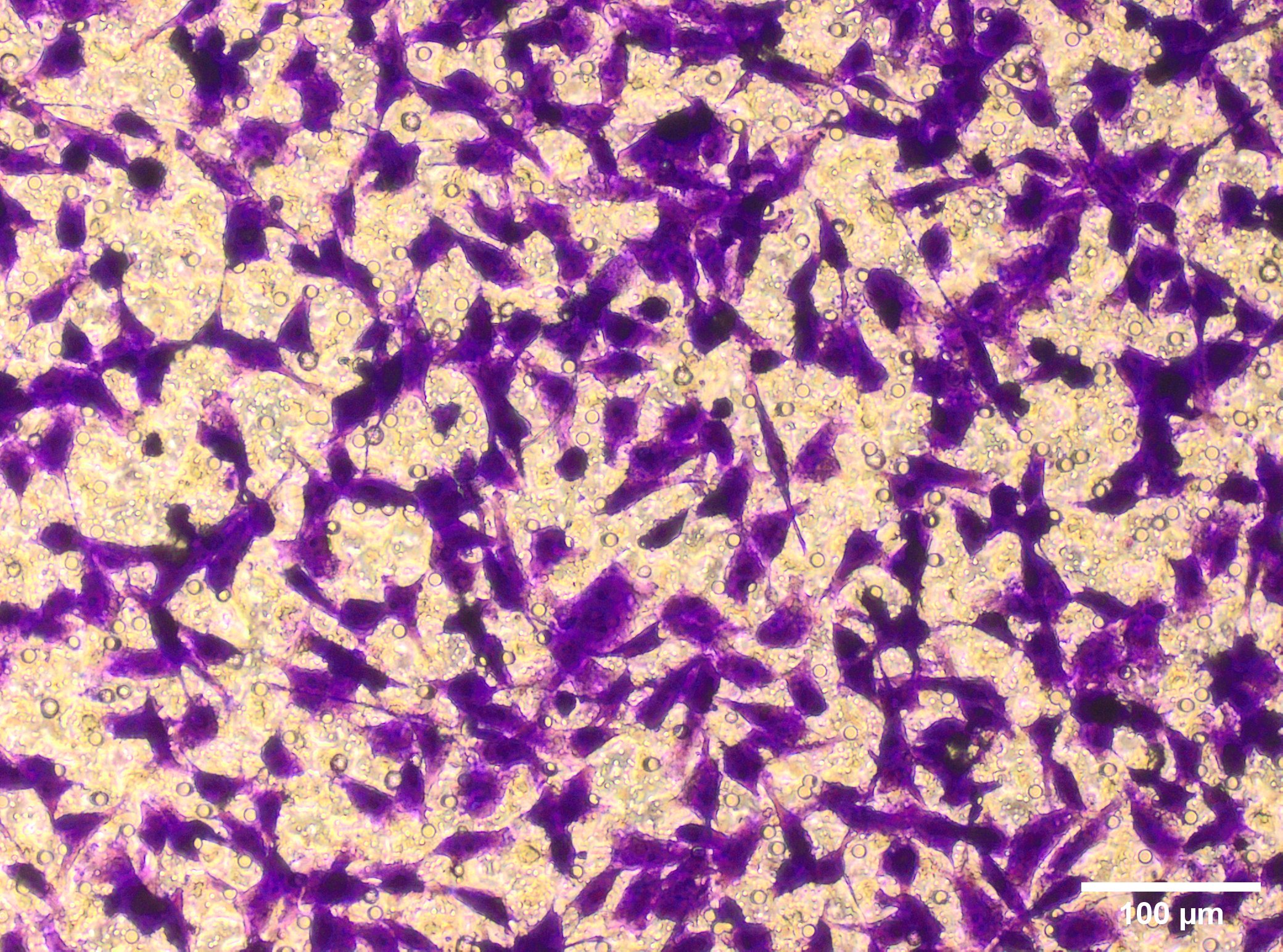

Supplement: Supplementary file 10 — EV Figure Source Data [file 44318_2026_766_MOESM10_ESM.zip › Figure EV2/Fig EV 2G/migration/dmso.jpg]

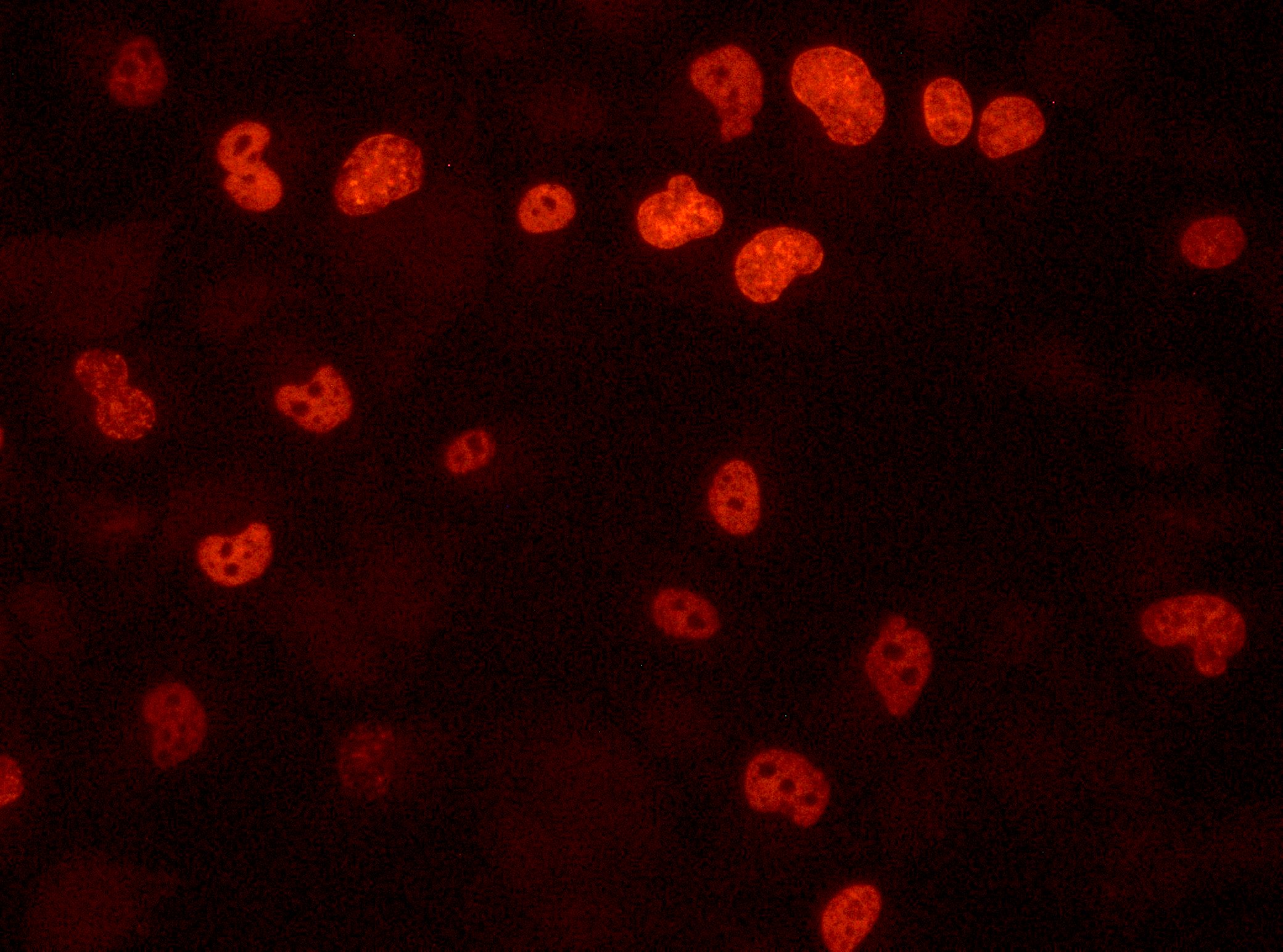

Supplement: Supplementary file 10 — EV Figure Source Data [file 44318_2026_766_MOESM10_ESM.zip › Figure EV2/Fig EV 2A/DMSO/edu.jpg]

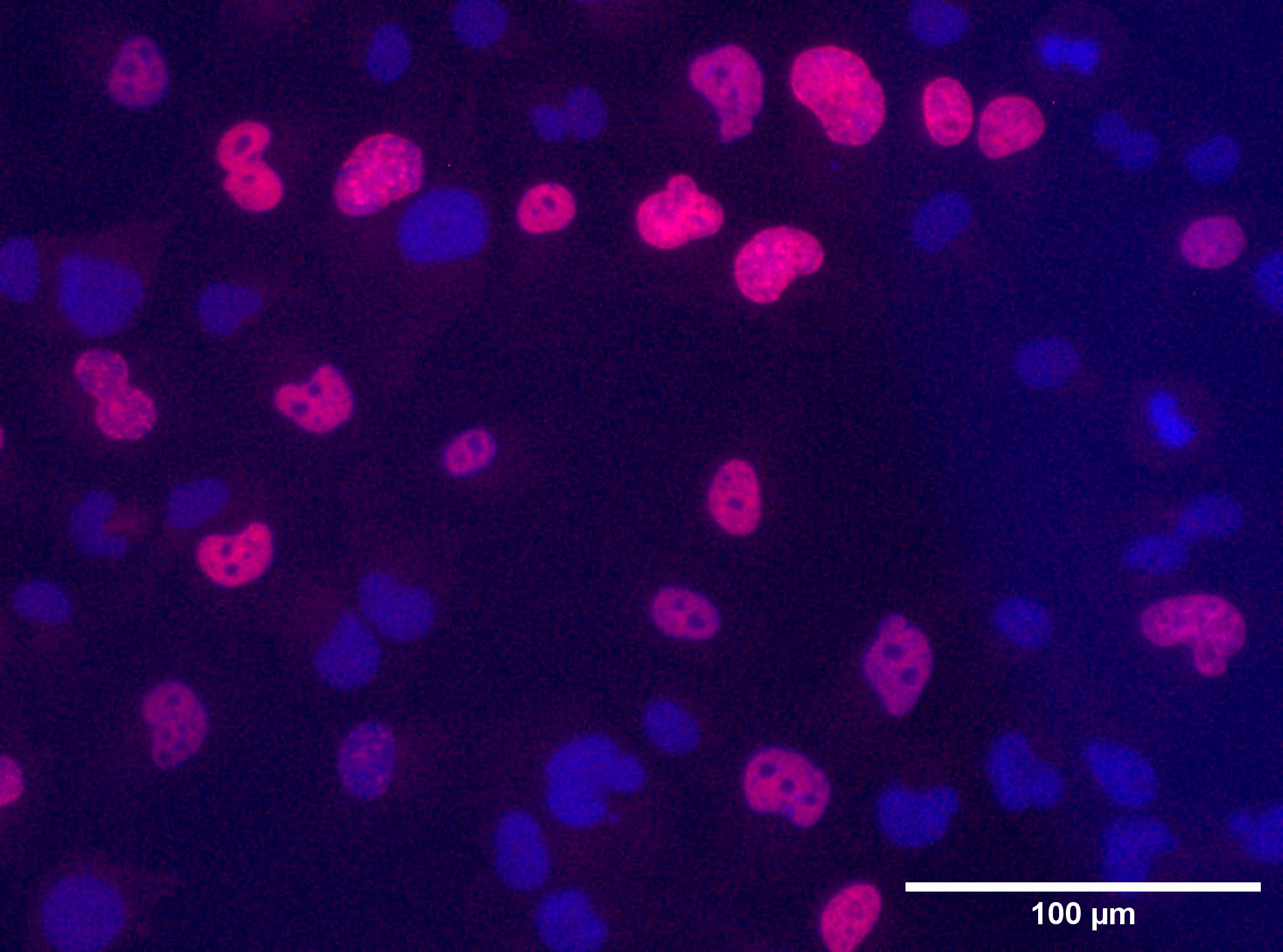

Supplement: Supplementary file 10 — EV Figure Source Data [file 44318_2026_766_MOESM10_ESM.zip › Figure EV2/Fig EV 2A/DMSO/Composite4.jpg]

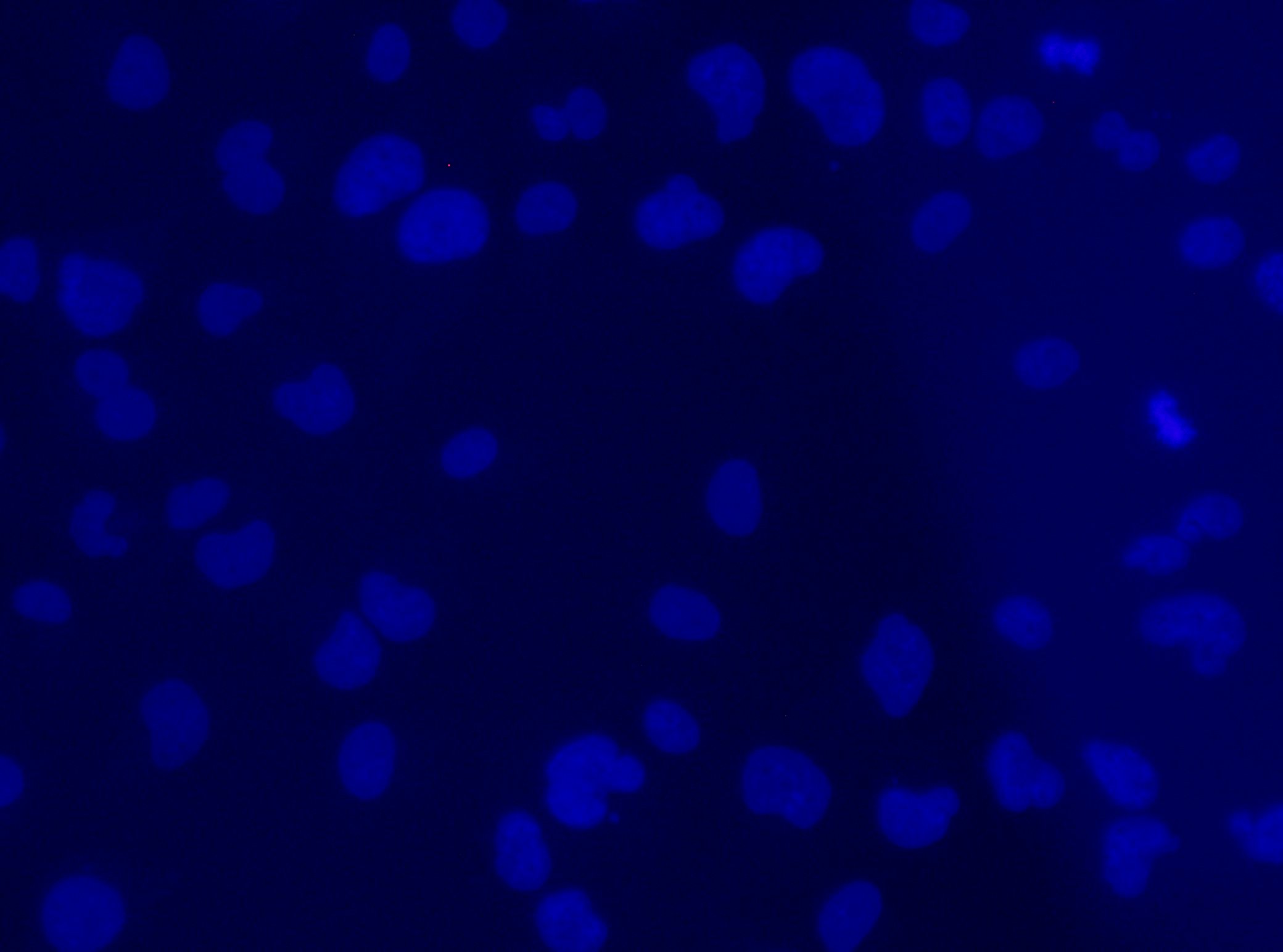

Supplement: Supplementary file 10 — EV Figure Source Data [file 44318_2026_766_MOESM10_ESM.zip › Figure EV2/Fig EV 2A/DMSO/dapi.jpg]

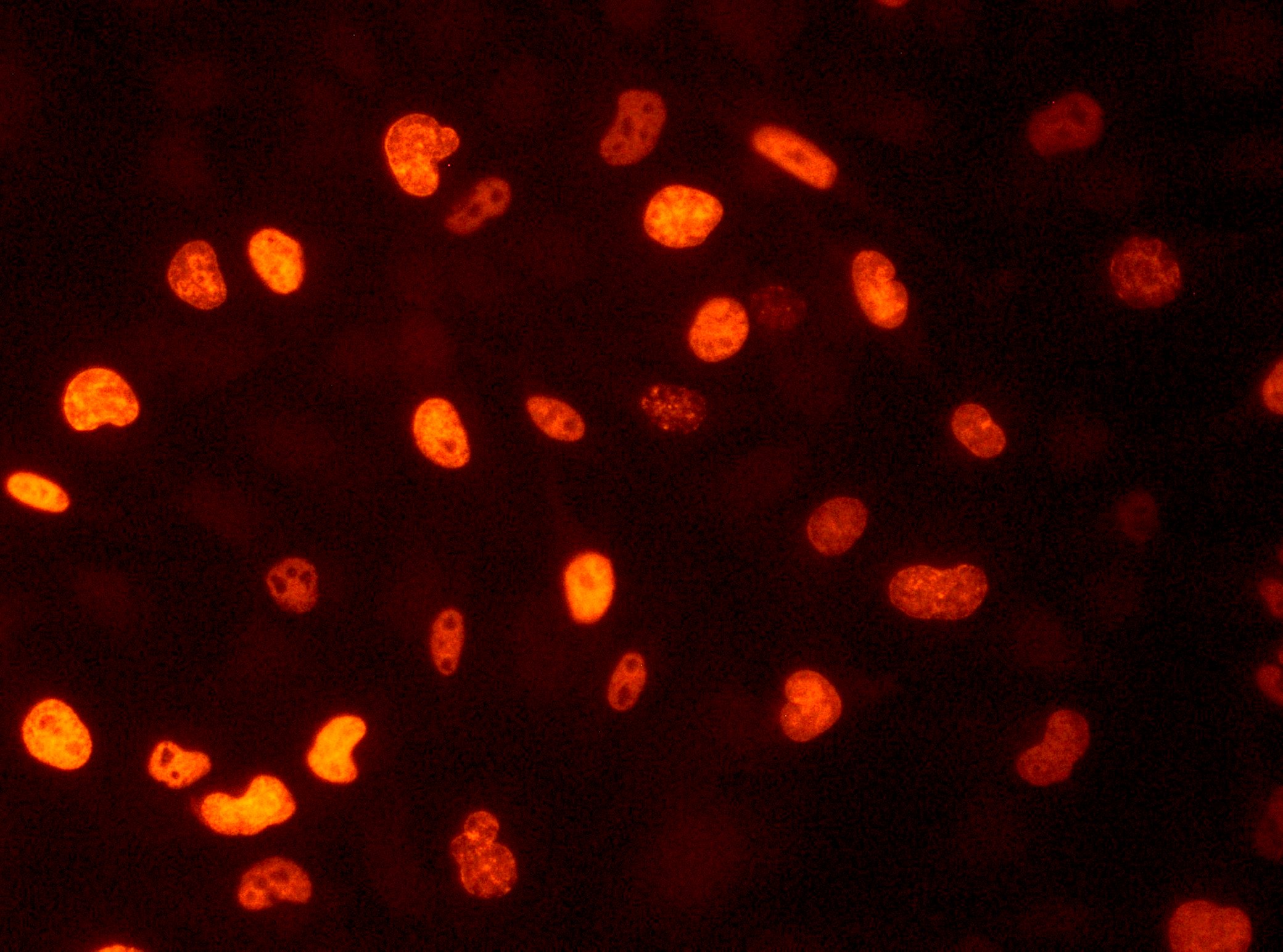

Supplement: Supplementary file 10 — EV Figure Source Data [file 44318_2026_766_MOESM10_ESM.zip › Figure EV2/Fig EV 2A/rev/edu.jpg]

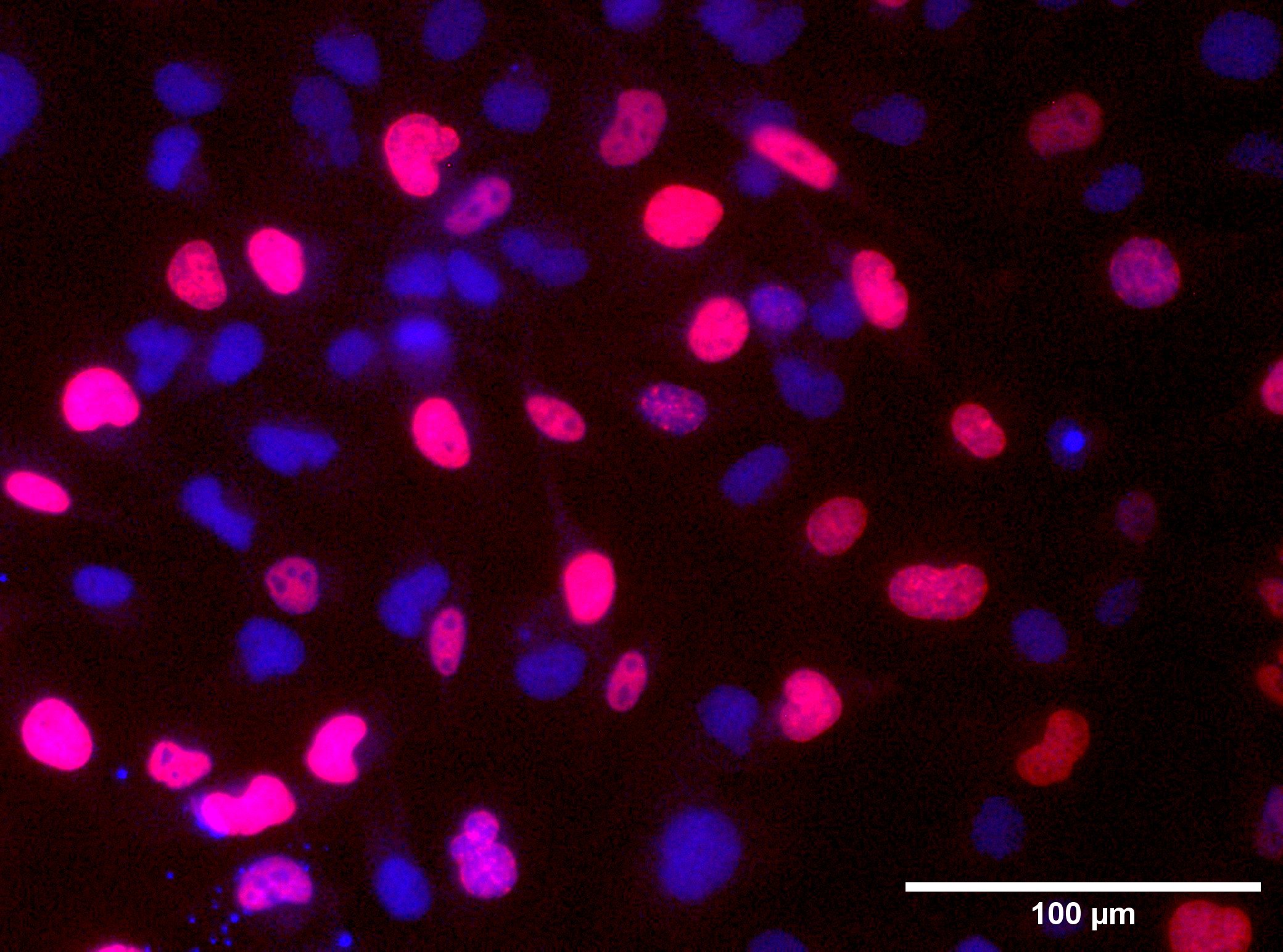

Supplement: Supplementary file 10 — EV Figure Source Data [file 44318_2026_766_MOESM10_ESM.zip › Figure EV2/Fig EV 2A/rev/Composite4.jpg]

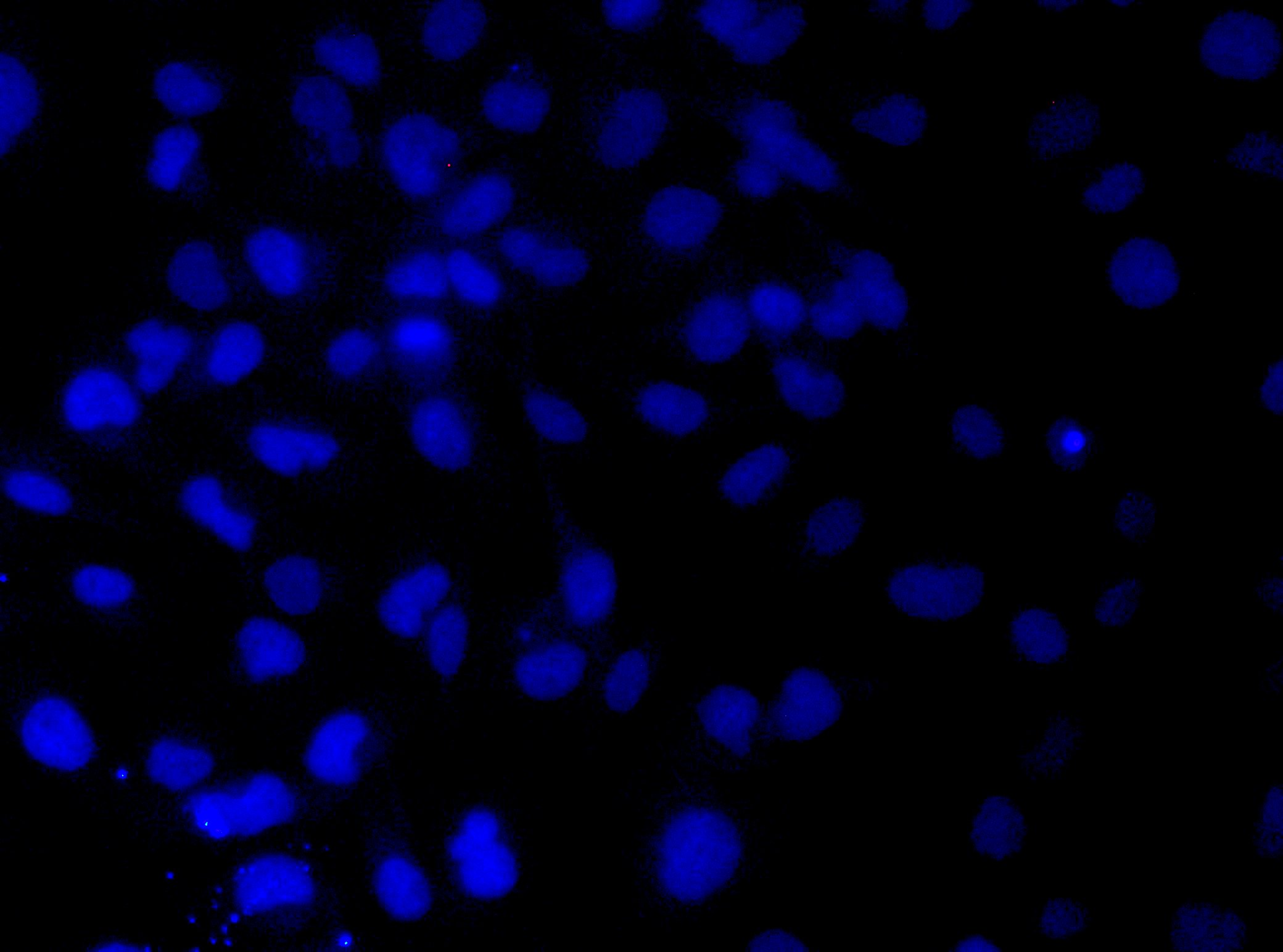

Supplement: Supplementary file 10 — EV Figure Source Data [file 44318_2026_766_MOESM10_ESM.zip › Figure EV2/Fig EV 2A/rev/dapi.jpg]

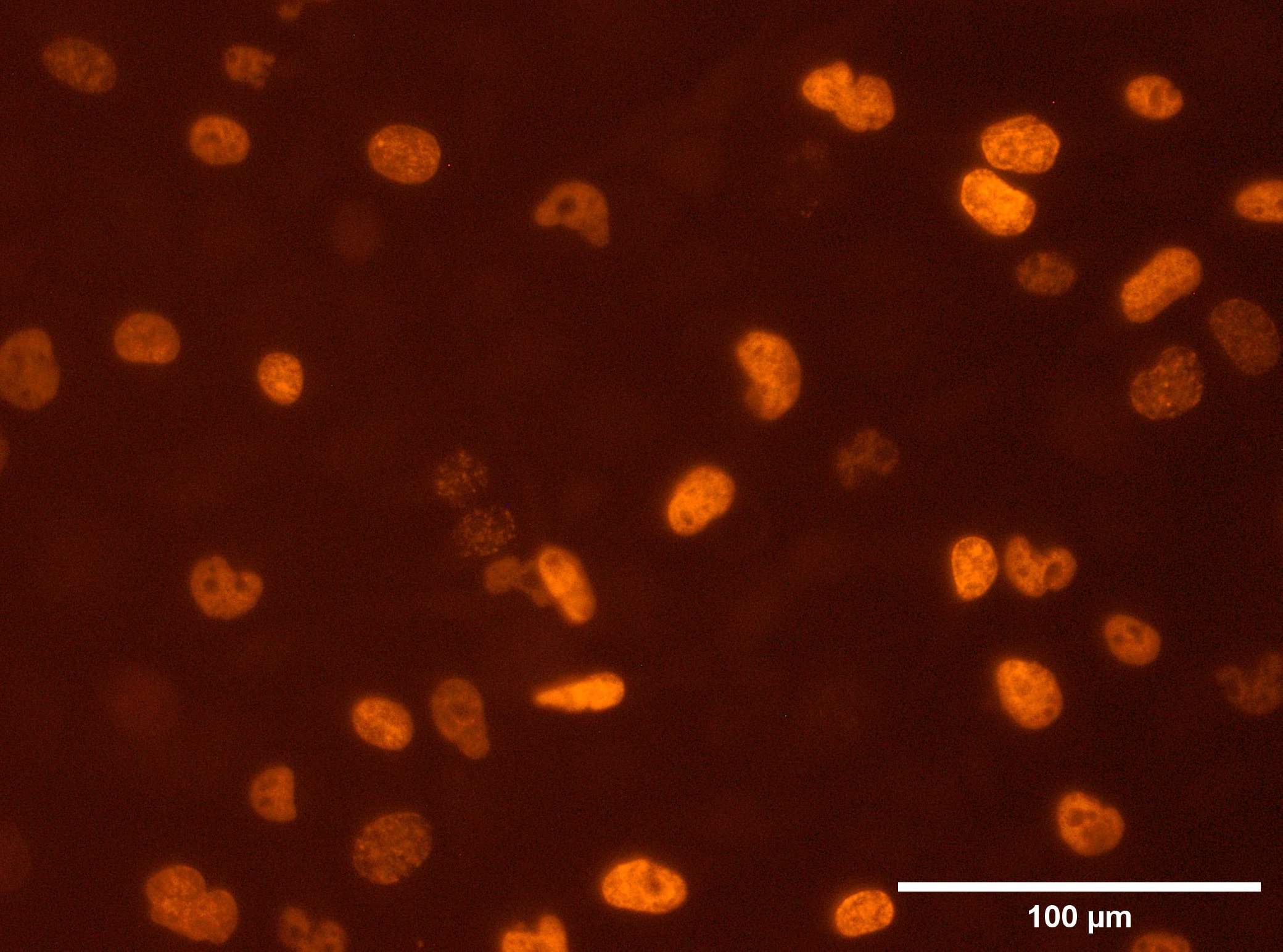

Supplement: Supplementary file 10 — EV Figure Source Data [file 44318_2026_766_MOESM10_ESM.zip › Figure EV2/Fig EV 2A/PBS/edu.jpg]

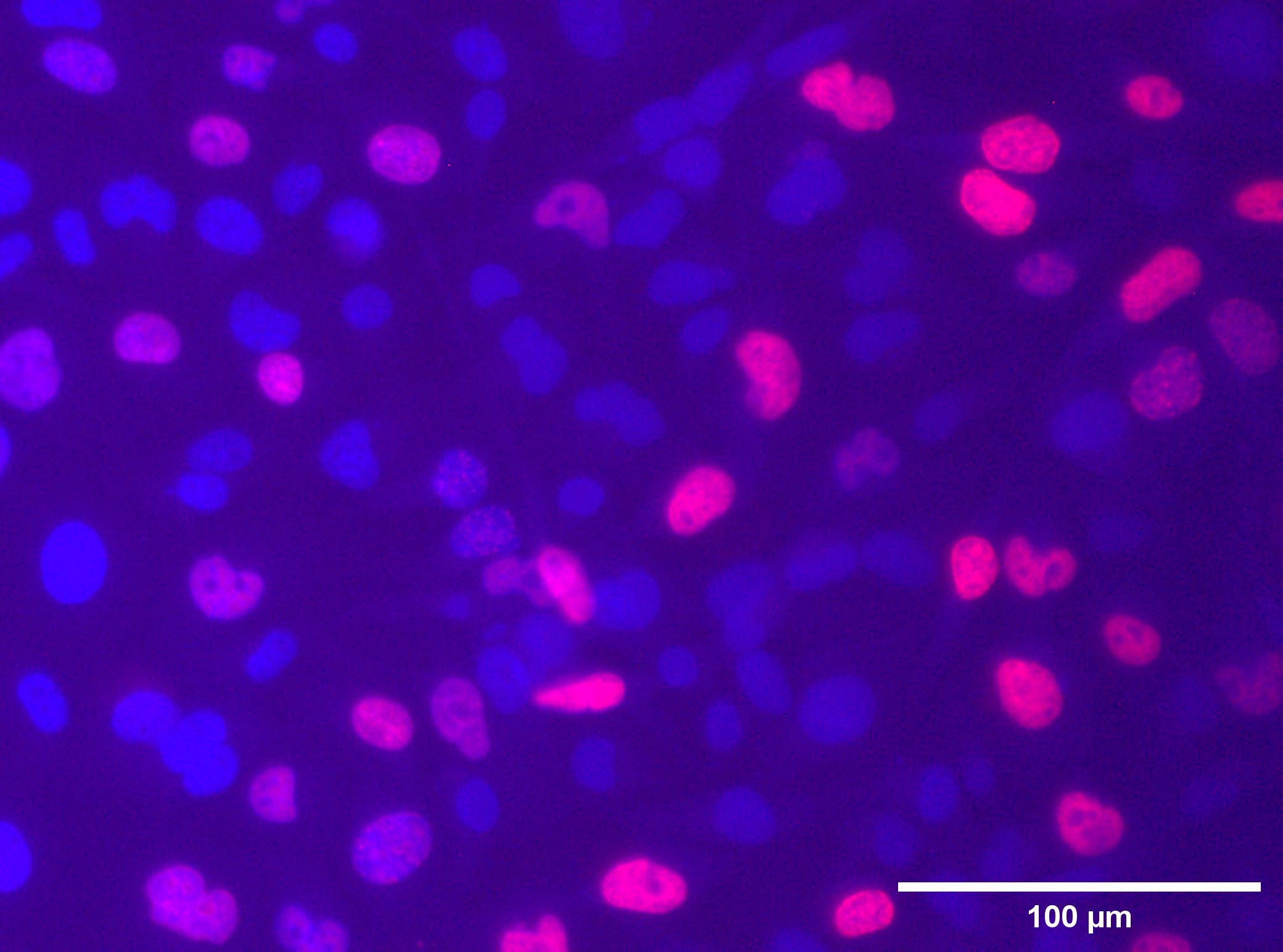

Supplement: Supplementary file 10 — EV Figure Source Data [file 44318_2026_766_MOESM10_ESM.zip › Figure EV2/Fig EV 2A/PBS/Composite.jpg]

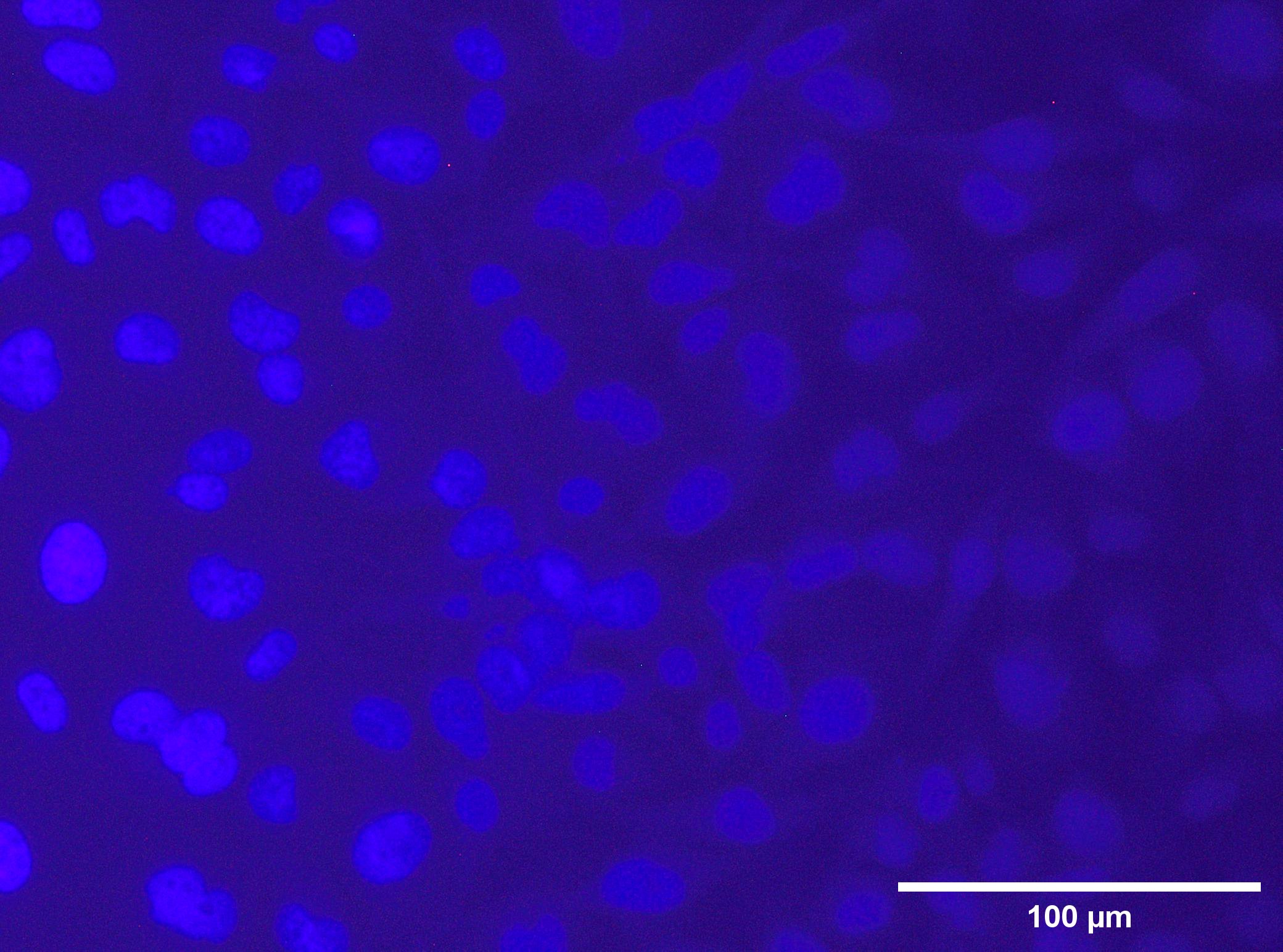

Supplement: Supplementary file 10 — EV Figure Source Data [file 44318_2026_766_MOESM10_ESM.zip › Figure EV2/Fig EV 2A/PBS/dapi.jpg]

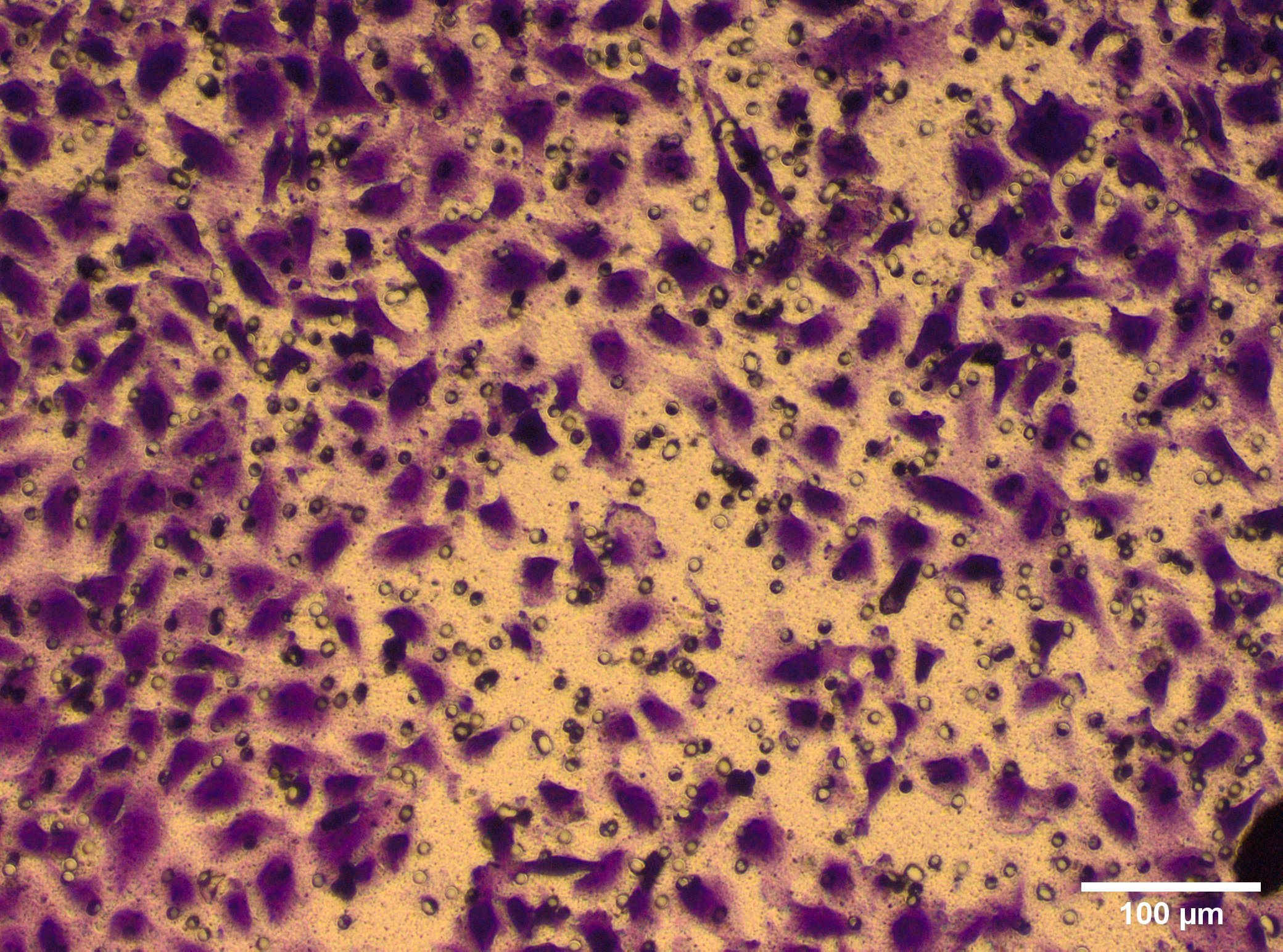

Supplement: Supplementary file 10 — EV Figure Source Data [file 44318_2026_766_MOESM10_ESM.zip › Figure EV2/Fig EV 2H/invasion/mda dmso.jpg]

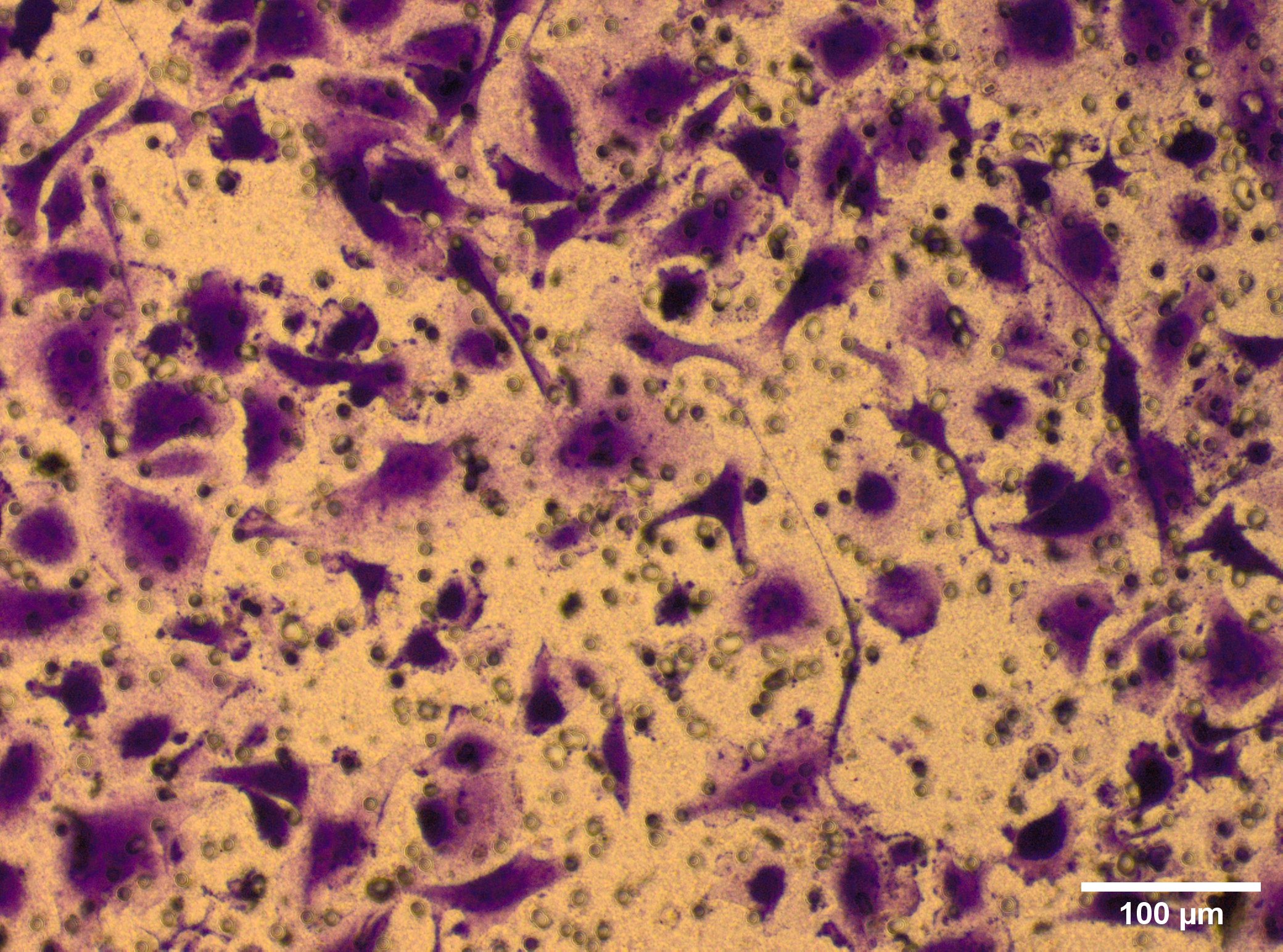

Supplement: Supplementary file 10 — EV Figure Source Data [file 44318_2026_766_MOESM10_ESM.zip › Figure EV2/Fig EV 2H/invasion/mda rev.jpg]

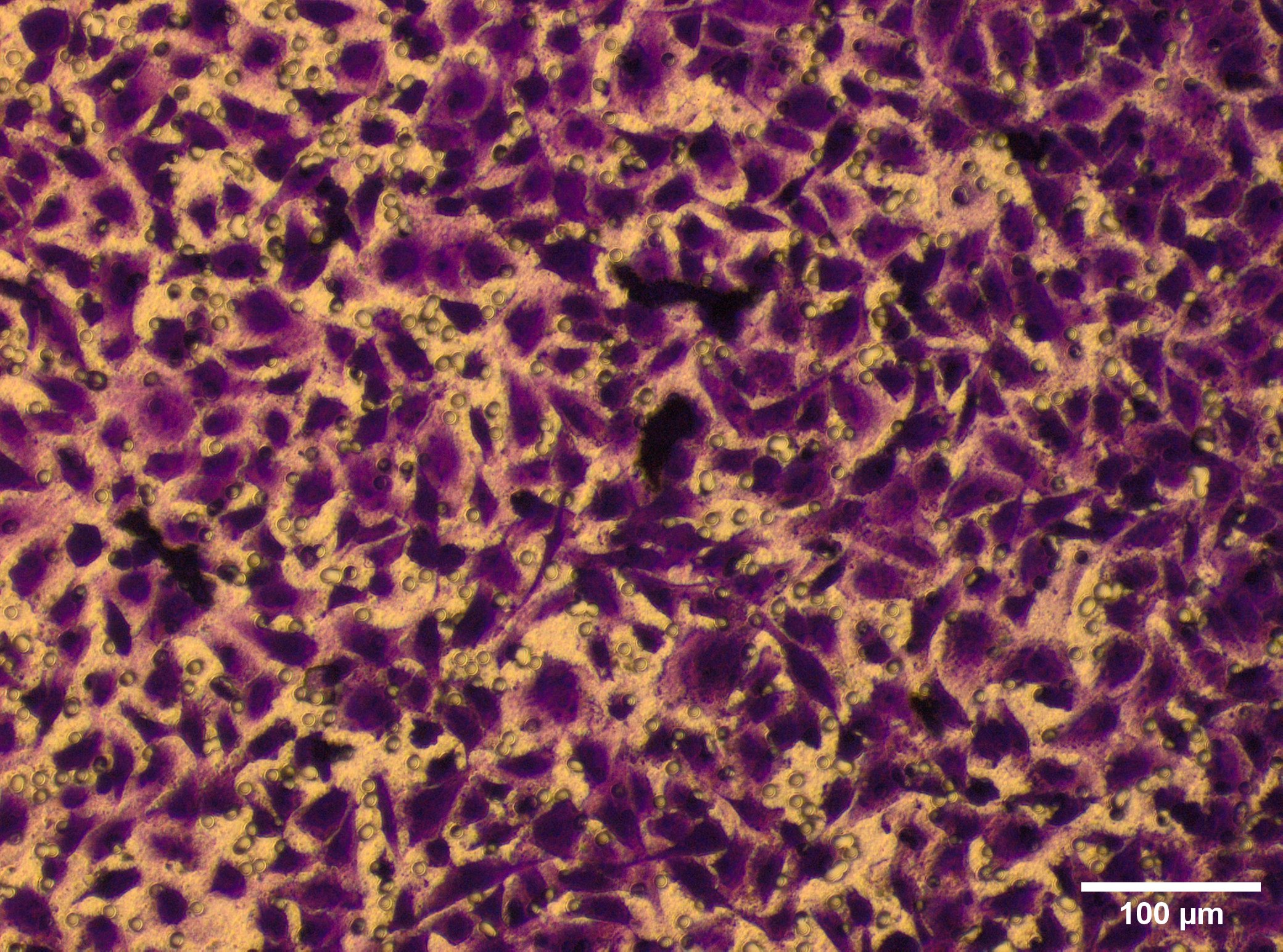

Supplement: Supplementary file 10 — EV Figure Source Data [file 44318_2026_766_MOESM10_ESM.zip › Figure EV2/Fig EV 2H/migration/mda dmso.jpg]

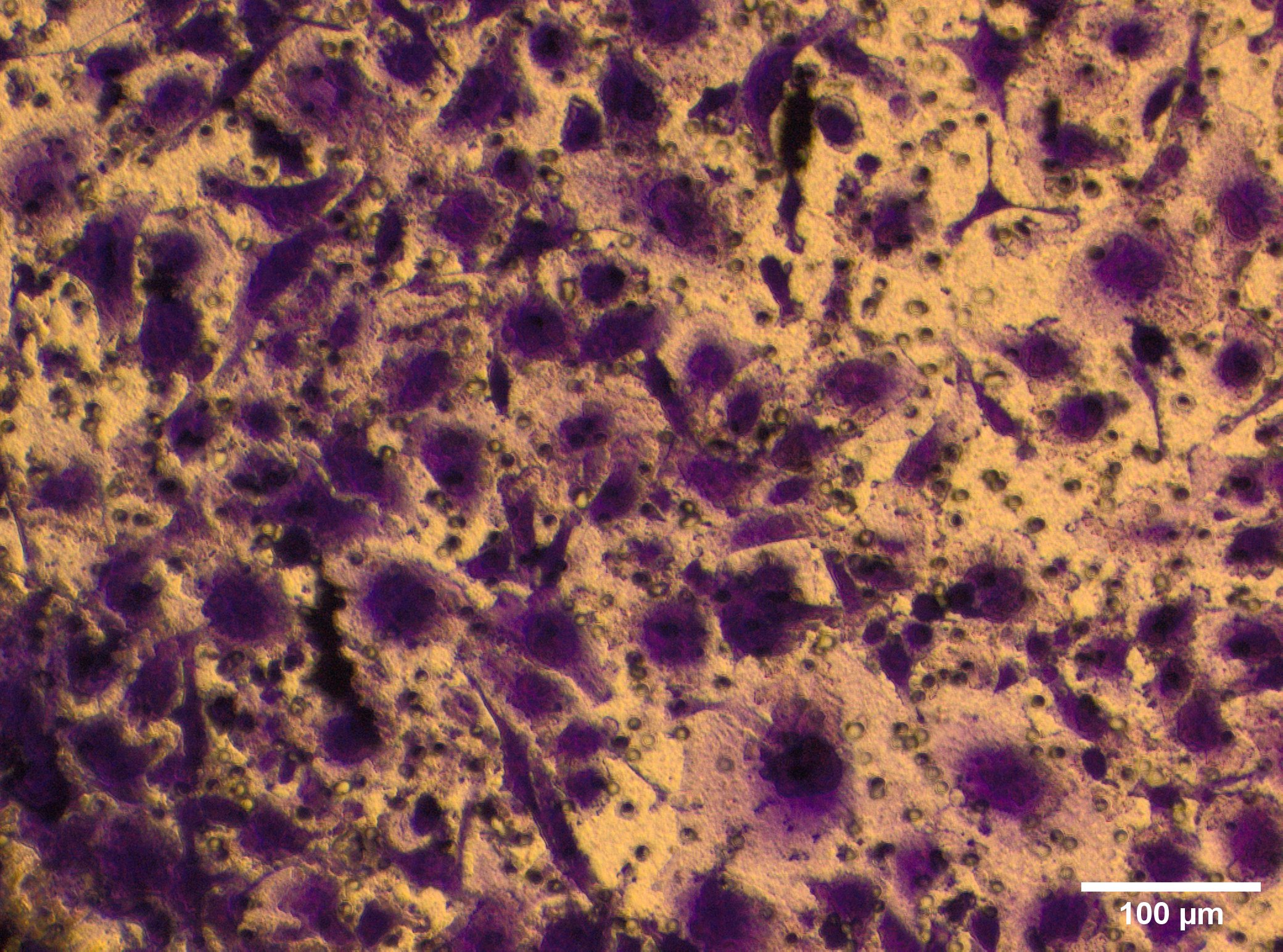

Supplement: Supplementary file 10 — EV Figure Source Data [file 44318_2026_766_MOESM10_ESM.zip › Figure EV2/Fig EV 2H/migration/mda rev.jpg]

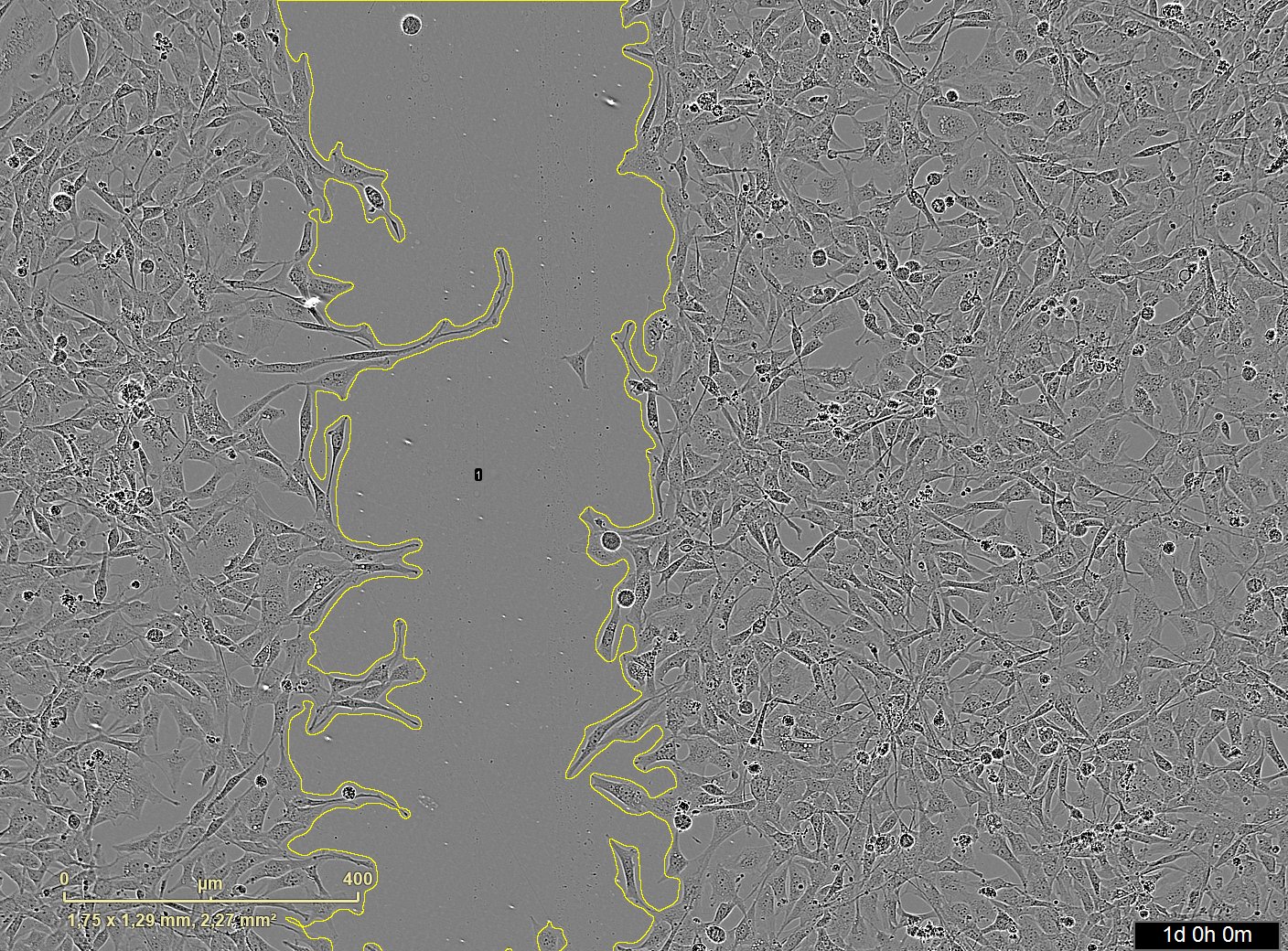

Supplement: Supplementary file 10 — EV Figure Source Data [file 44318_2026_766_MOESM10_ESM.zip › Figure EV4/Fig EV 4I/vector1_A1_1_01d00h00m-2.jpg]

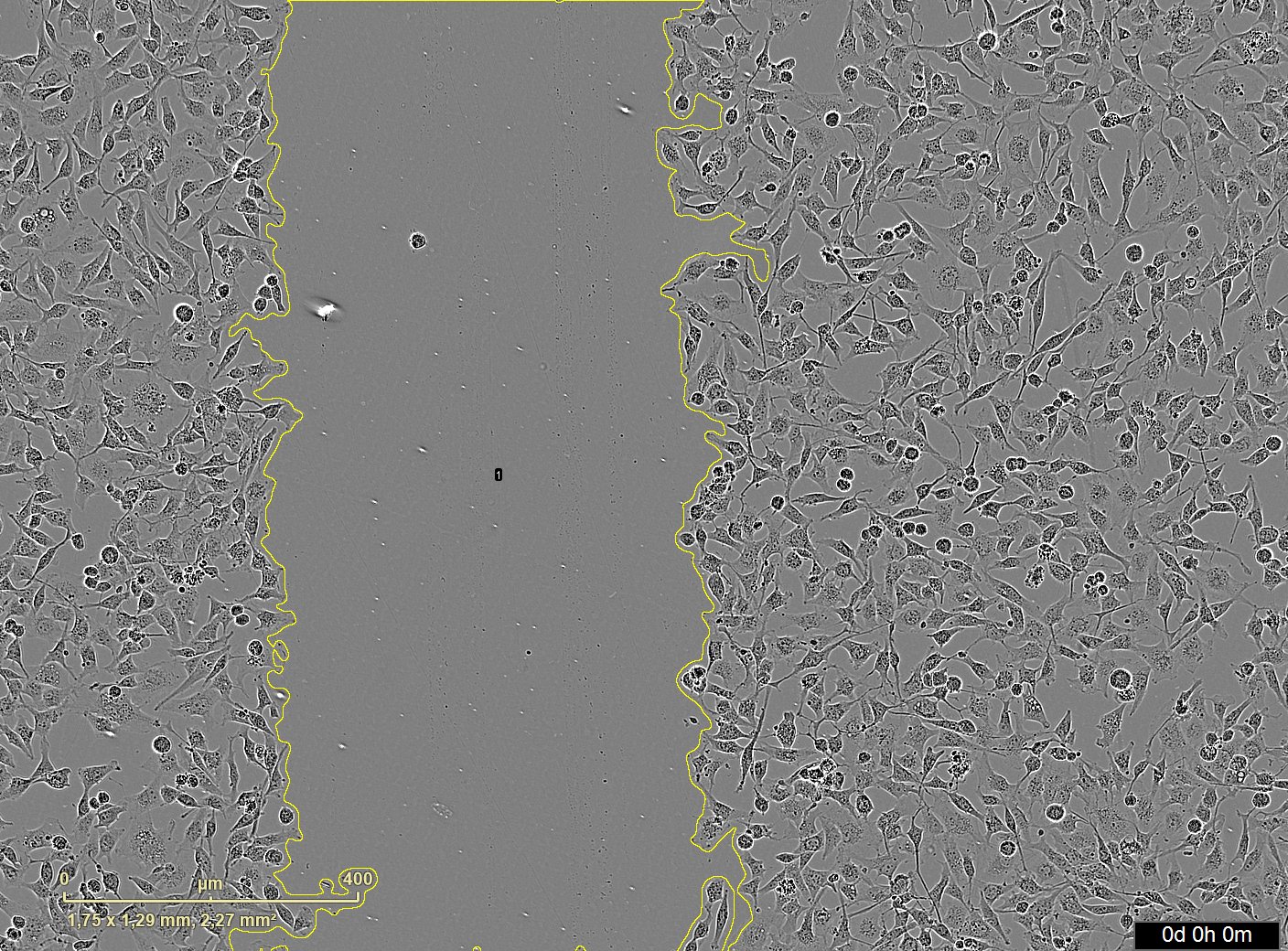

Supplement: Supplementary file 10 — EV Figure Source Data [file 44318_2026_766_MOESM10_ESM.zip › Figure EV4/Fig EV 4I/vector1_A1_1_00d00h00m-2.jpg]

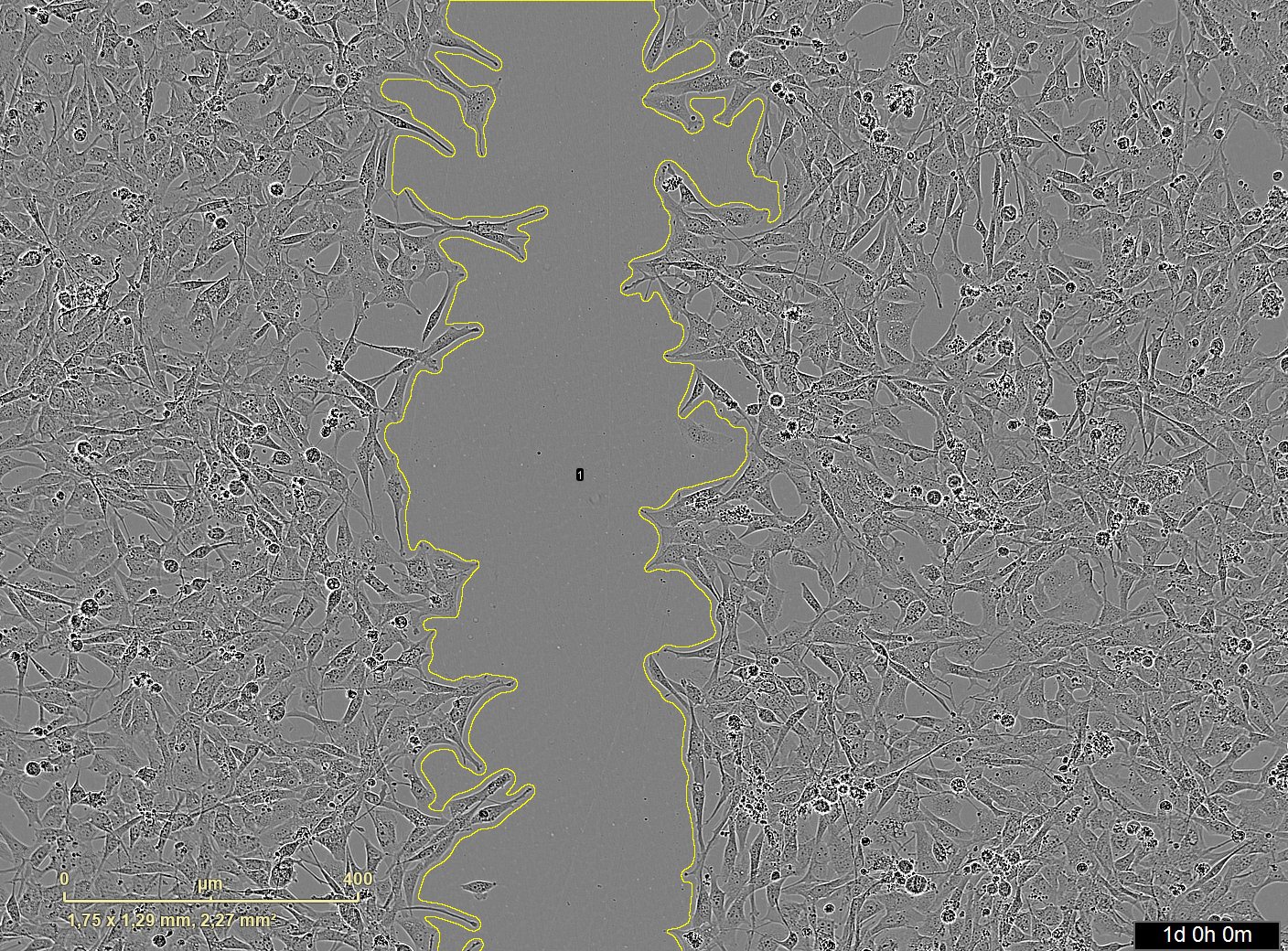

Supplement: Supplementary file 10 — EV Figure Source Data [file 44318_2026_766_MOESM10_ESM.zip › Figure EV4/Fig EV 4I/efemp1-2_B4_1_01d00h00m-3.jpg]

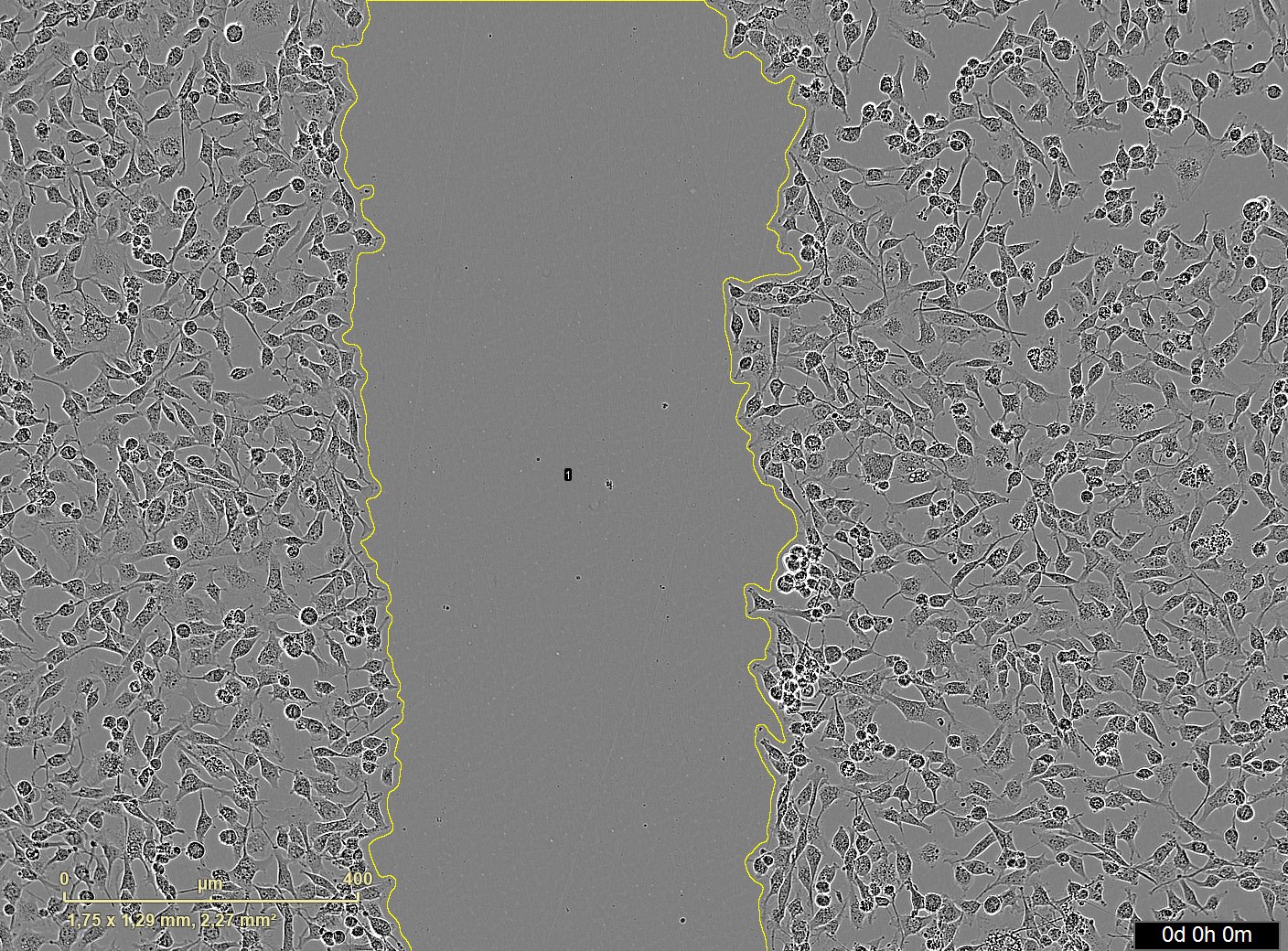

Supplement: Supplementary file 10 — EV Figure Source Data [file 44318_2026_766_MOESM10_ESM.zip › Figure EV4/Fig EV 4I/efemp1-2_B4_1_00d00h00m-2.jpg]

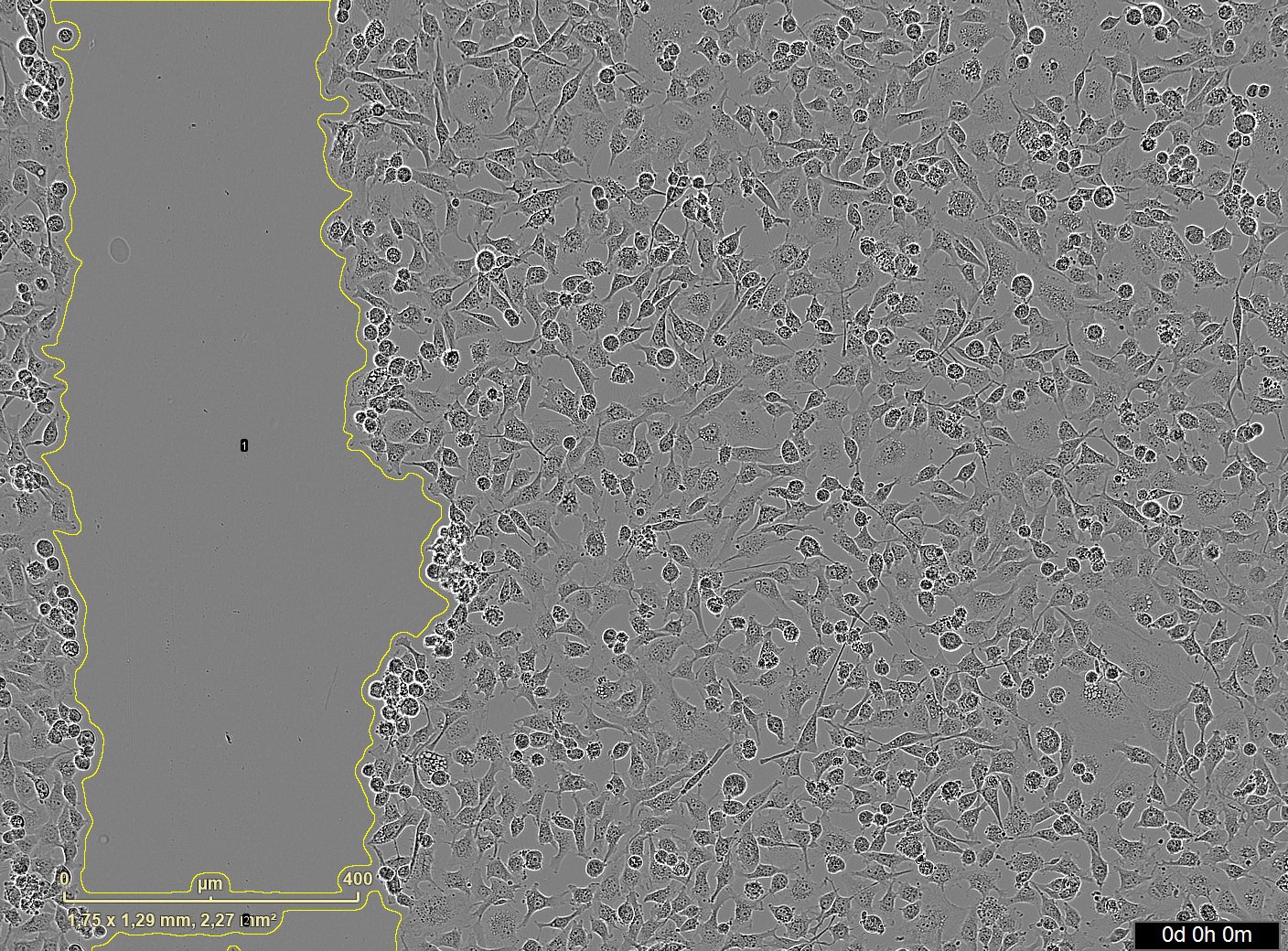

Supplement: Supplementary file 10 — EV Figure Source Data [file 44318_2026_766_MOESM10_ESM.zip › Figure EV4/Fig EV 4J/sh5 no1_A3_1_00d00h00m-3.jpg]

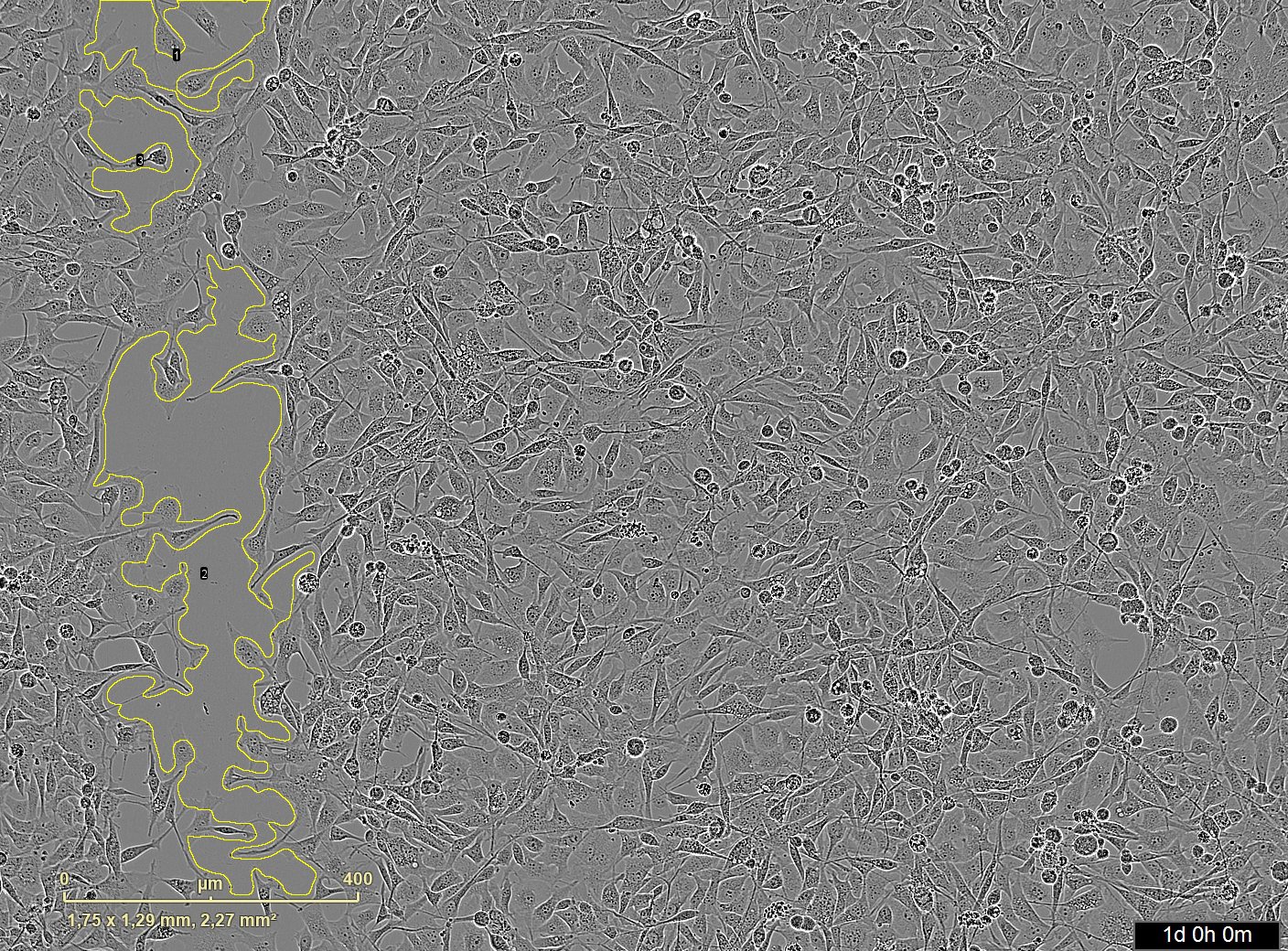

Supplement: Supplementary file 10 — EV Figure Source Data [file 44318_2026_766_MOESM10_ESM.zip › Figure EV4/Fig EV 4J/sh5 no1_A3_1_01d00h00m-3.jpg]

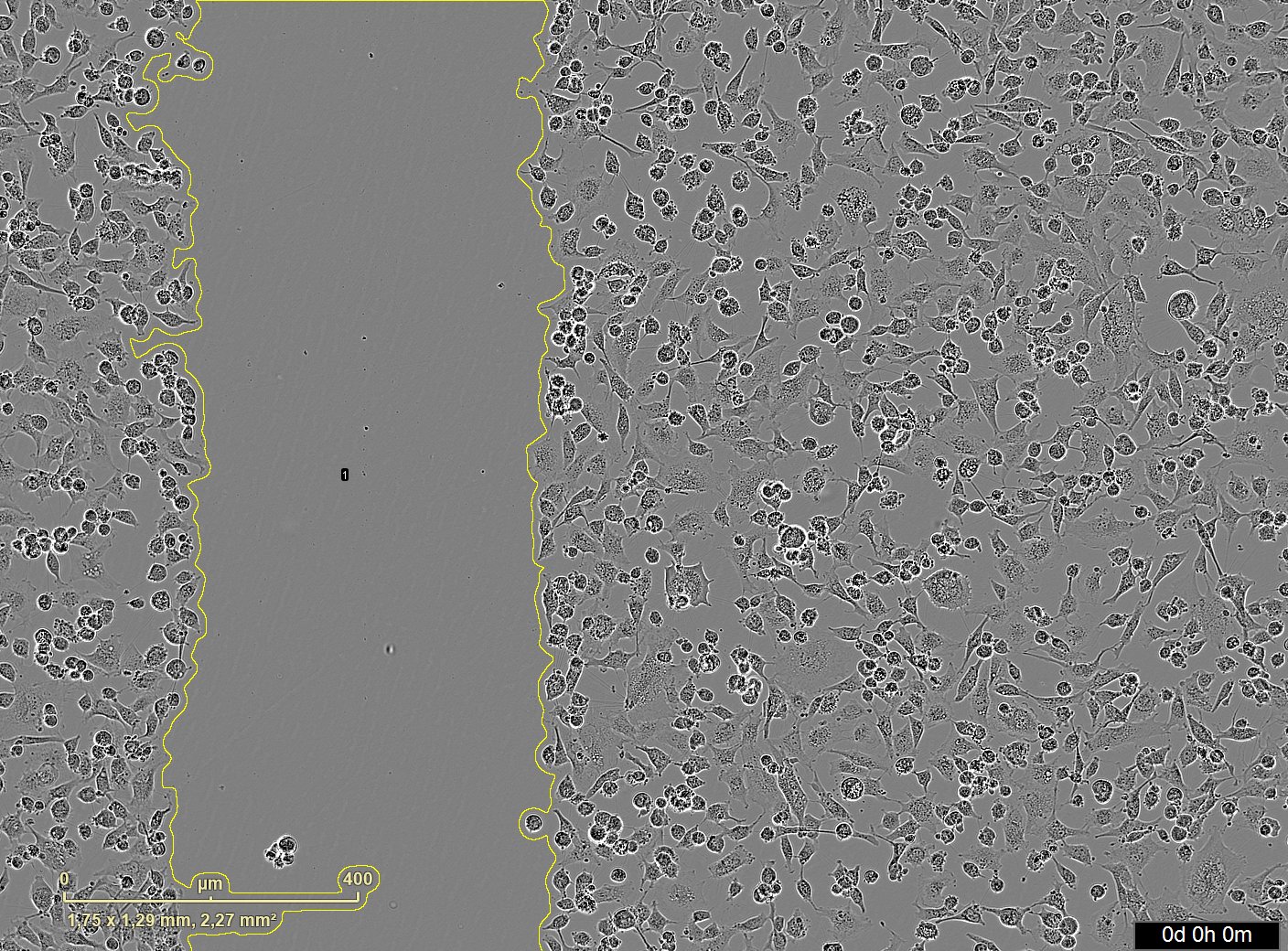

Supplement: Supplementary file 10 — EV Figure Source Data [file 44318_2026_766_MOESM10_ESM.zip › Figure EV4/Fig EV 4J/sh5+dox_C4_2_00d00h00m-2.jpg]

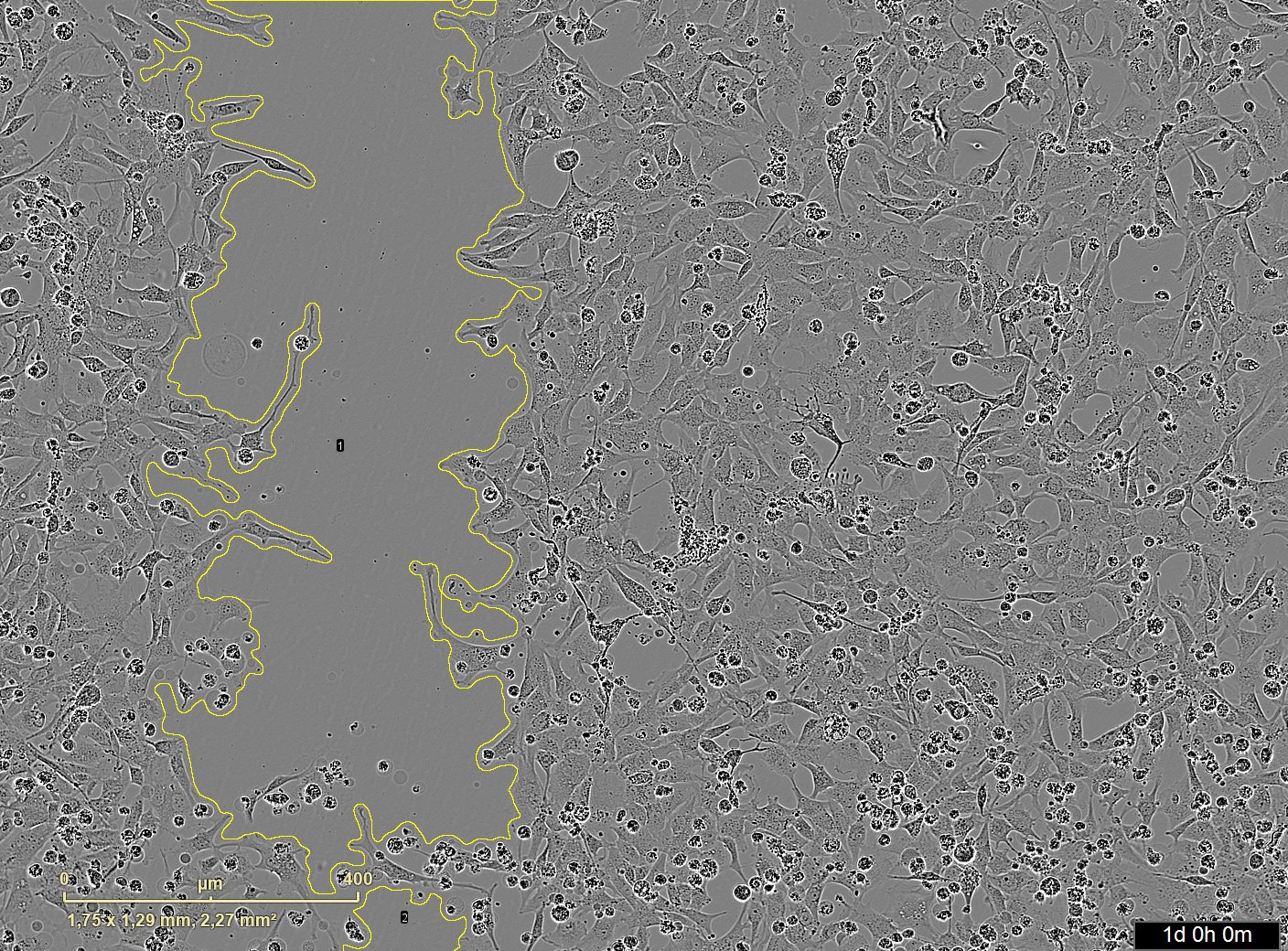

Supplement: Supplementary file 10 — EV Figure Source Data [file 44318_2026_766_MOESM10_ESM.zip › Figure EV4/Fig EV 4J/sh5+dox_C4_2_01d00h00m-3.jpg]

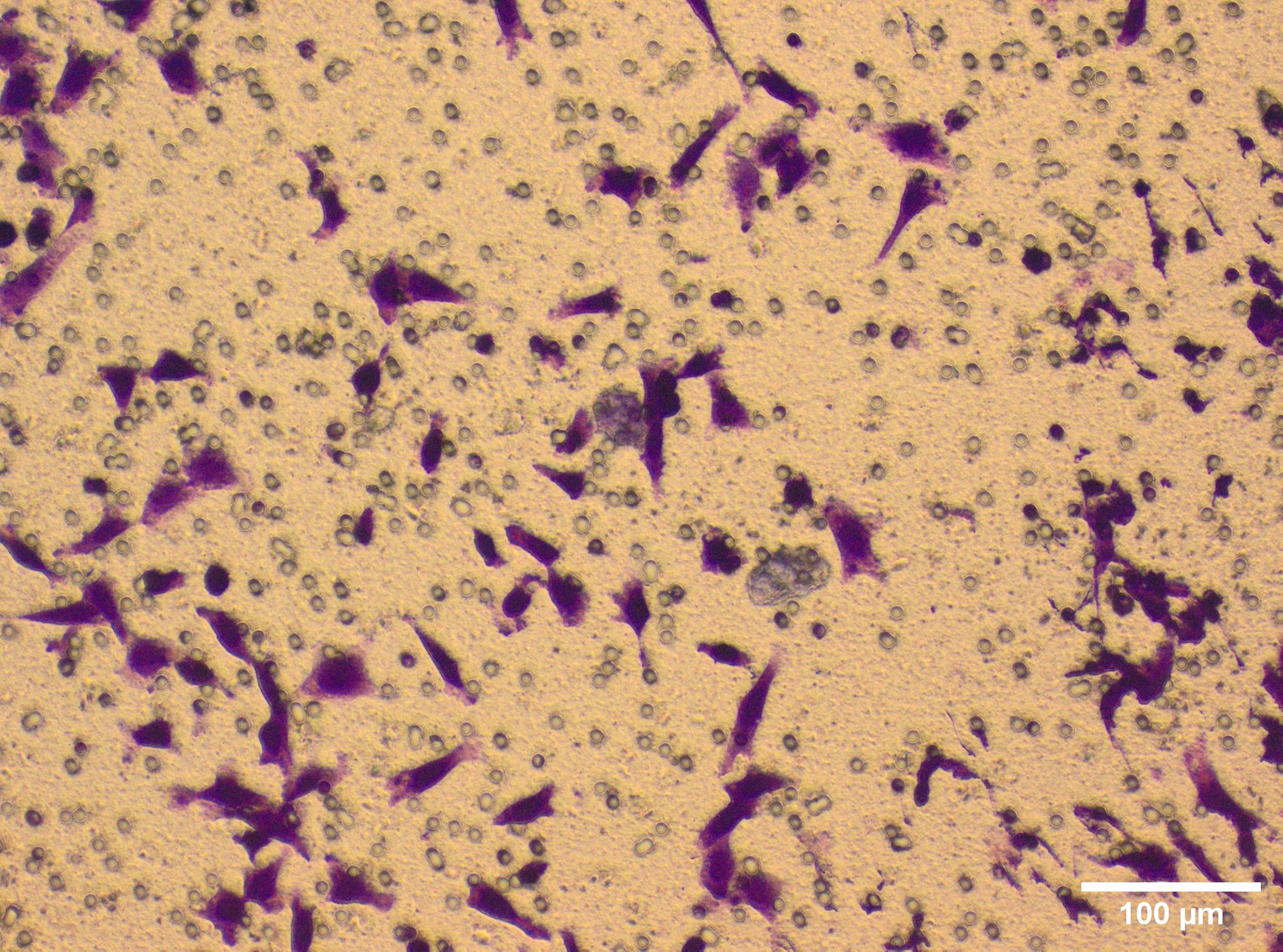

Supplement: Supplementary file 10 — EV Figure Source Data [file 44318_2026_766_MOESM10_ESM.zip › Figure EV4/Fig EV 4G/invasion/mda vector inva90Image_6553.jpg]

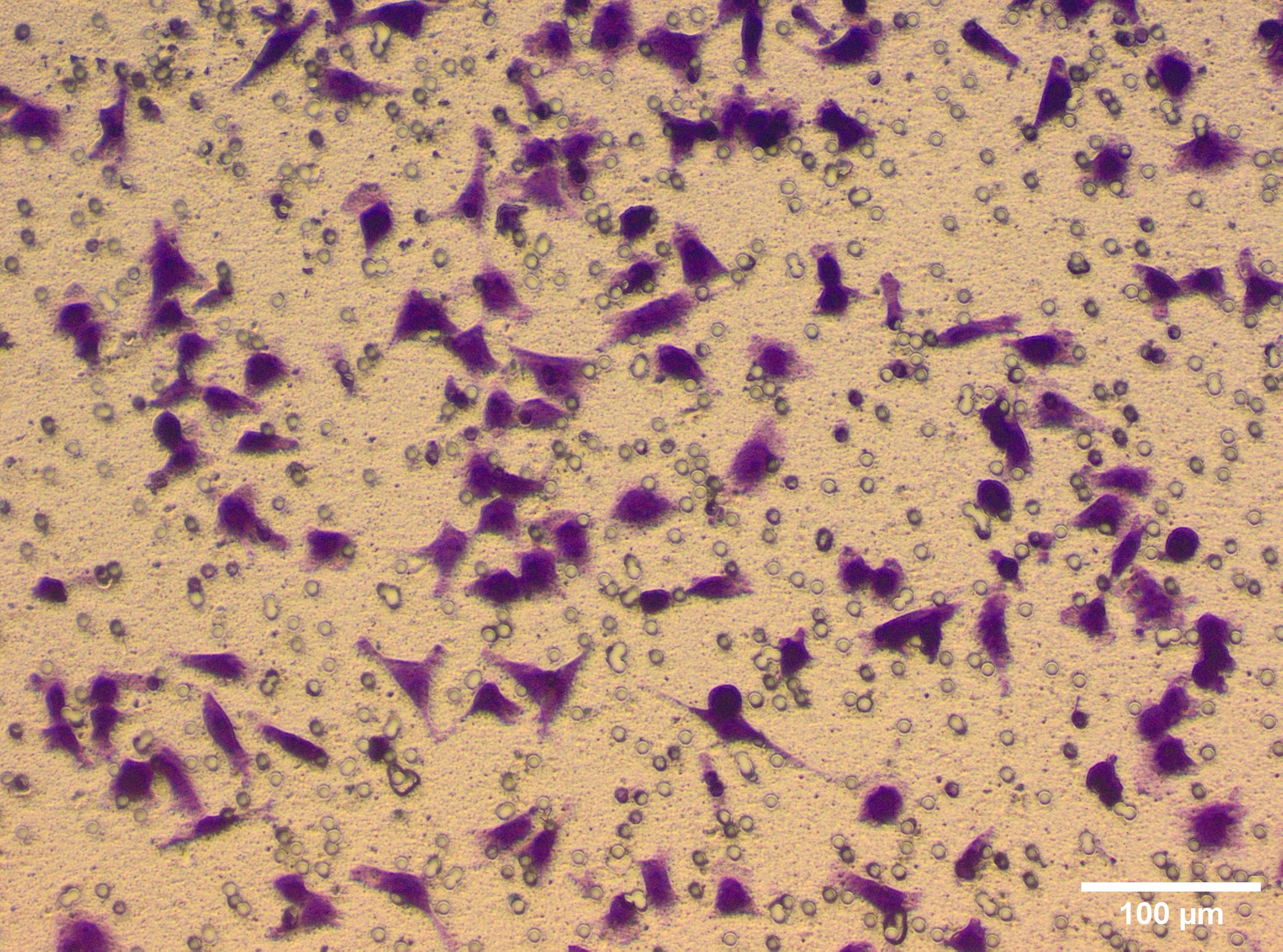

Supplement: Supplementary file 10 — EV Figure Source Data [file 44318_2026_766_MOESM10_ESM.zip › Figure EV4/Fig EV 4G/invasion/efemp1.jpg]

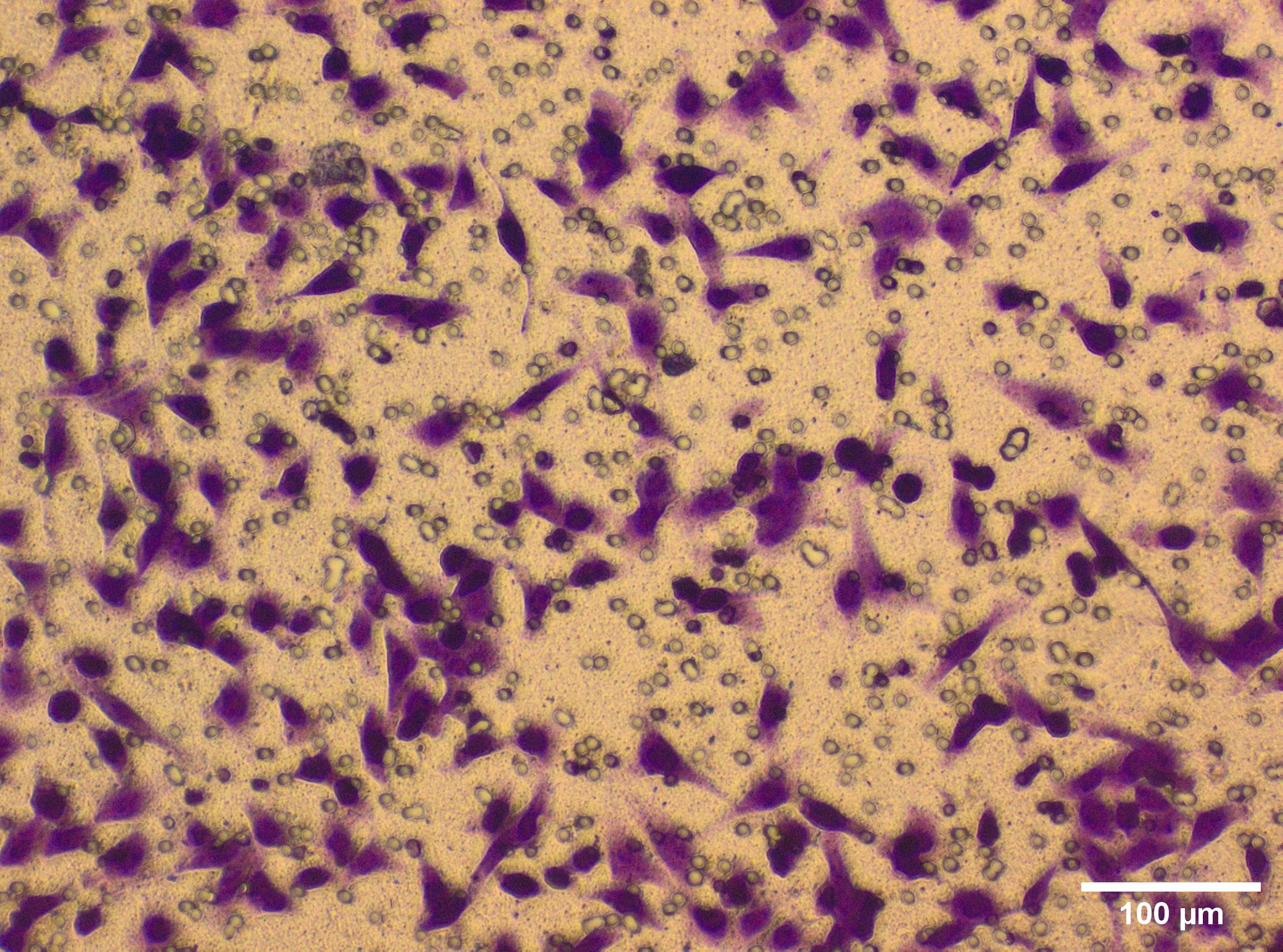

Supplement: Supplementary file 10 — EV Figure Source Data [file 44318_2026_766_MOESM10_ESM.zip › Figure EV4/Fig EV 4G/migration/vector.jpg]

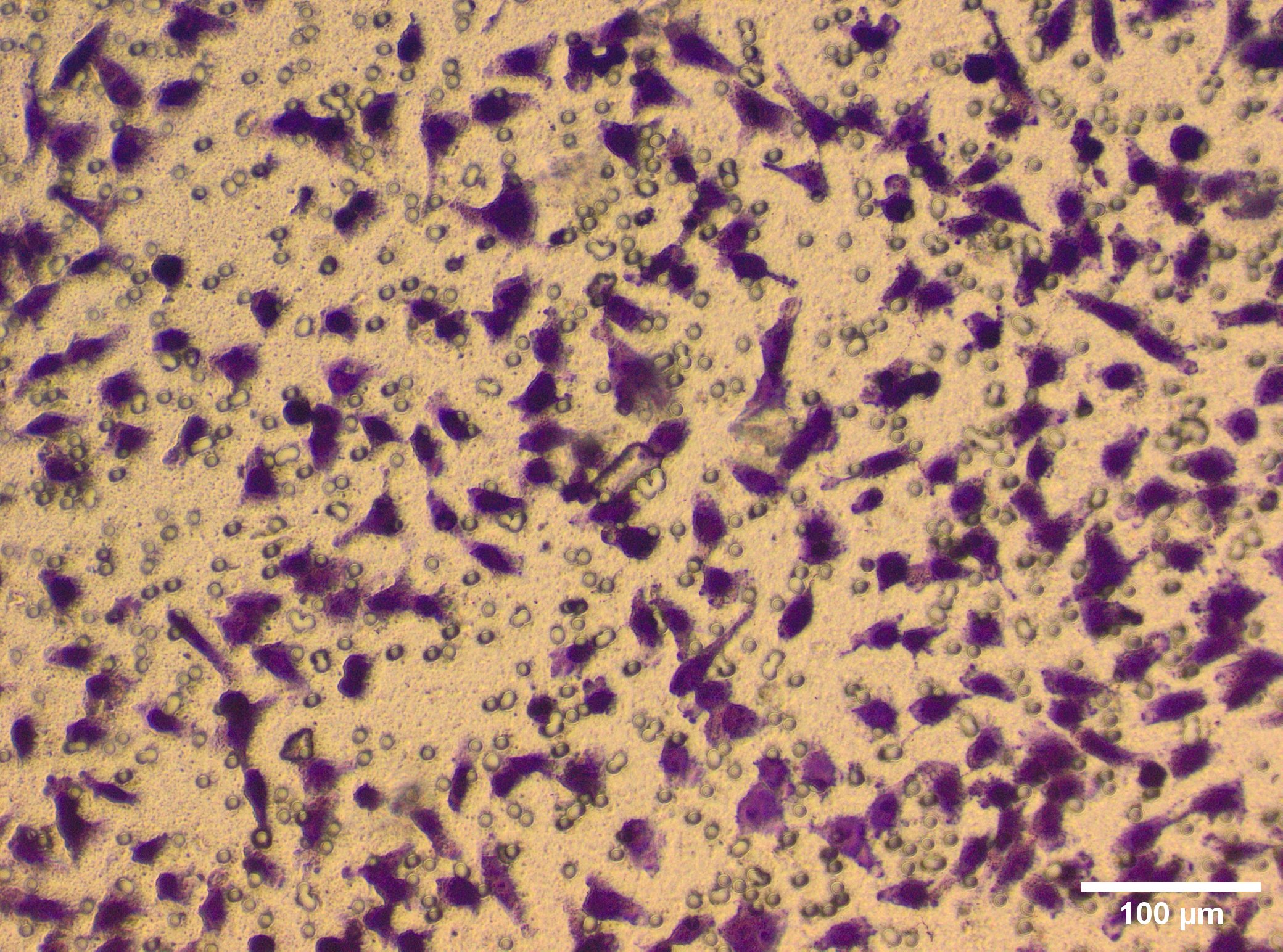

Supplement: Supplementary file 10 — EV Figure Source Data [file 44318_2026_766_MOESM10_ESM.zip › Figure EV4/Fig EV 4G/migration/mda efe mig244Image_6669.jpg]

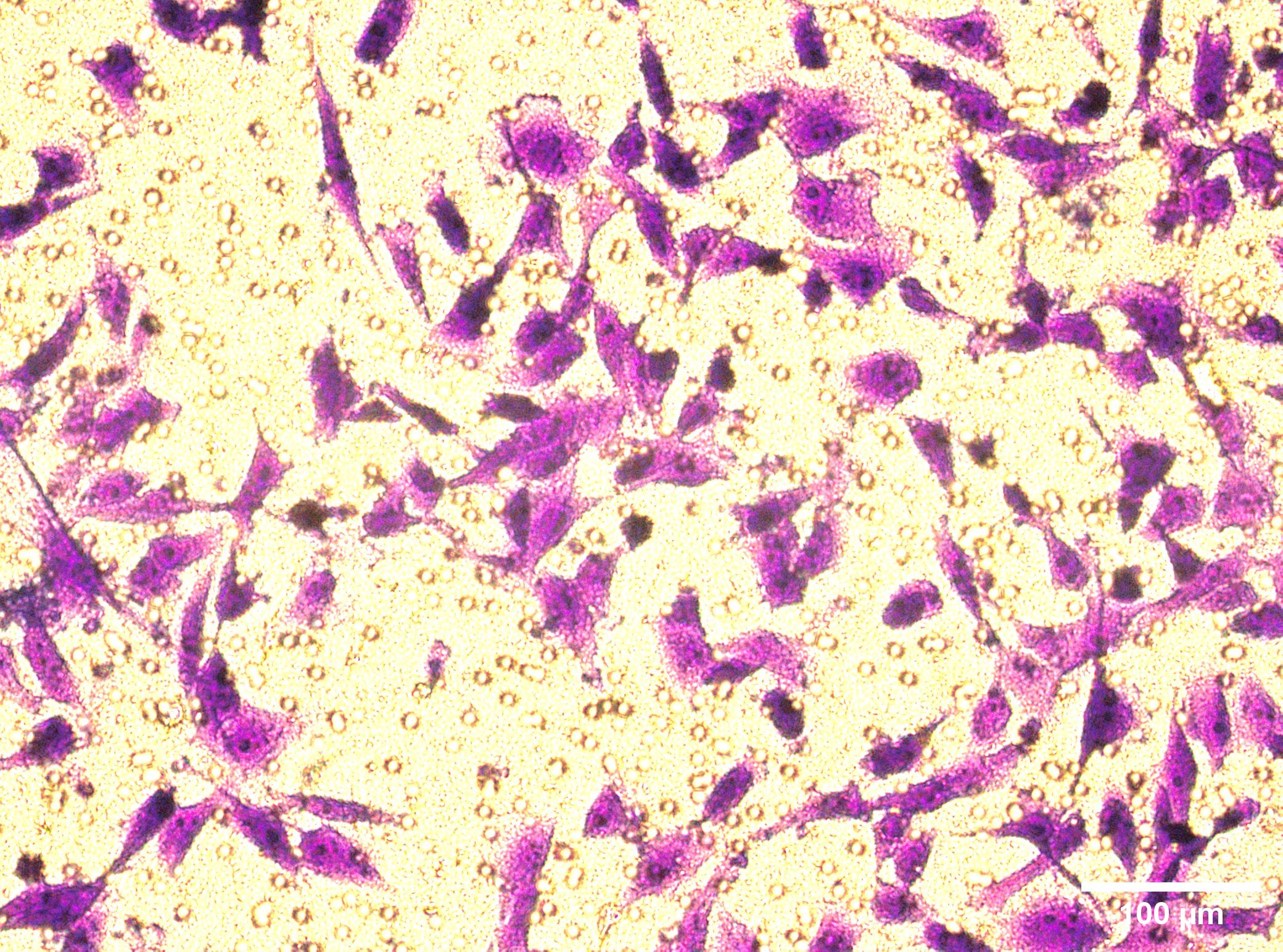

Supplement: Supplementary file 10 — EV Figure Source Data [file 44318_2026_766_MOESM10_ESM.zip › Figure EV4/Fig EV 4K/invasion/scramble dox.jpg]

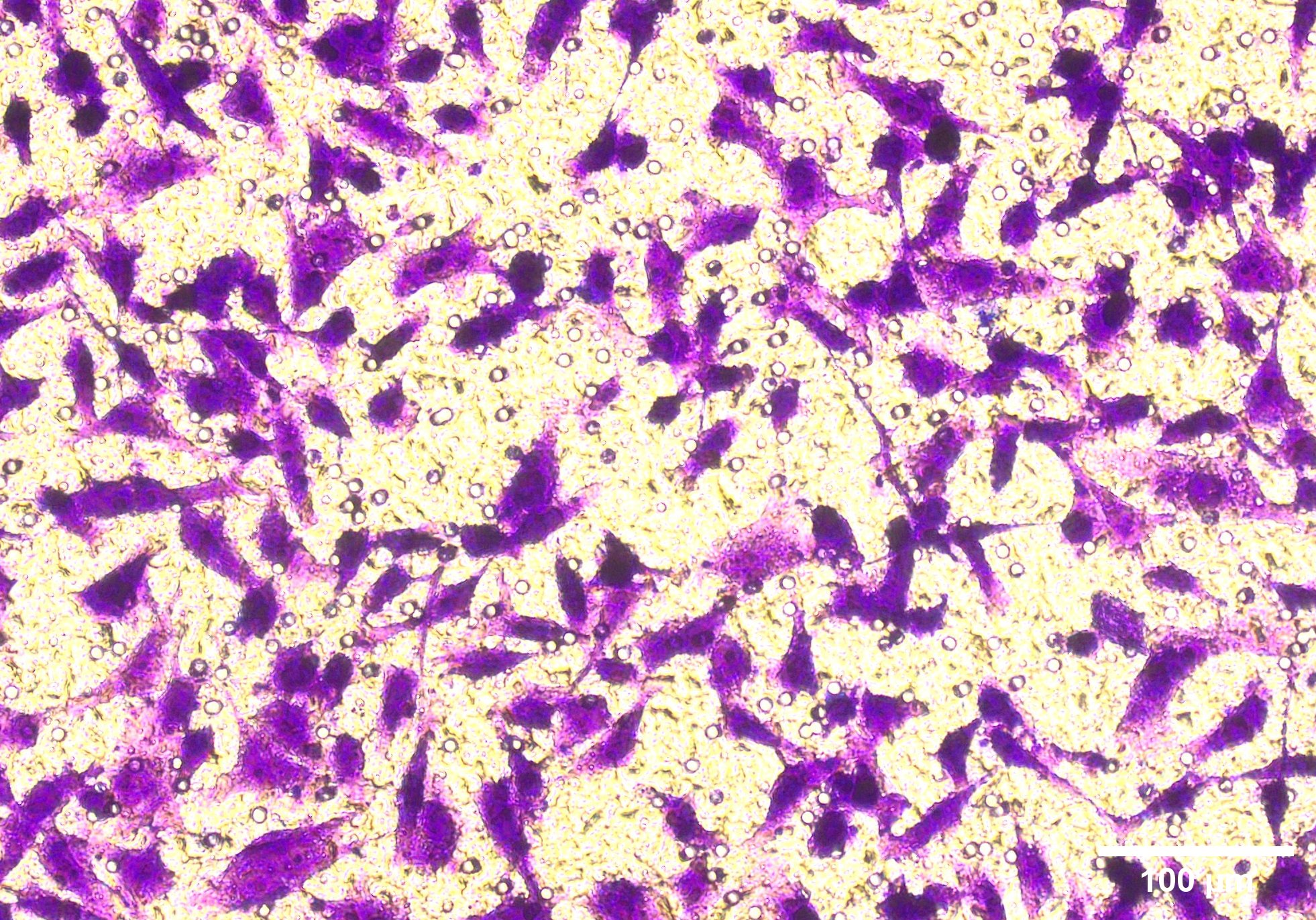

Supplement: Supplementary file 10 — EV Figure Source Data [file 44318_2026_766_MOESM10_ESM.zip › Figure EV4/Fig EV 4K/invasion/sh4 no invasion-1-bar.jpg]

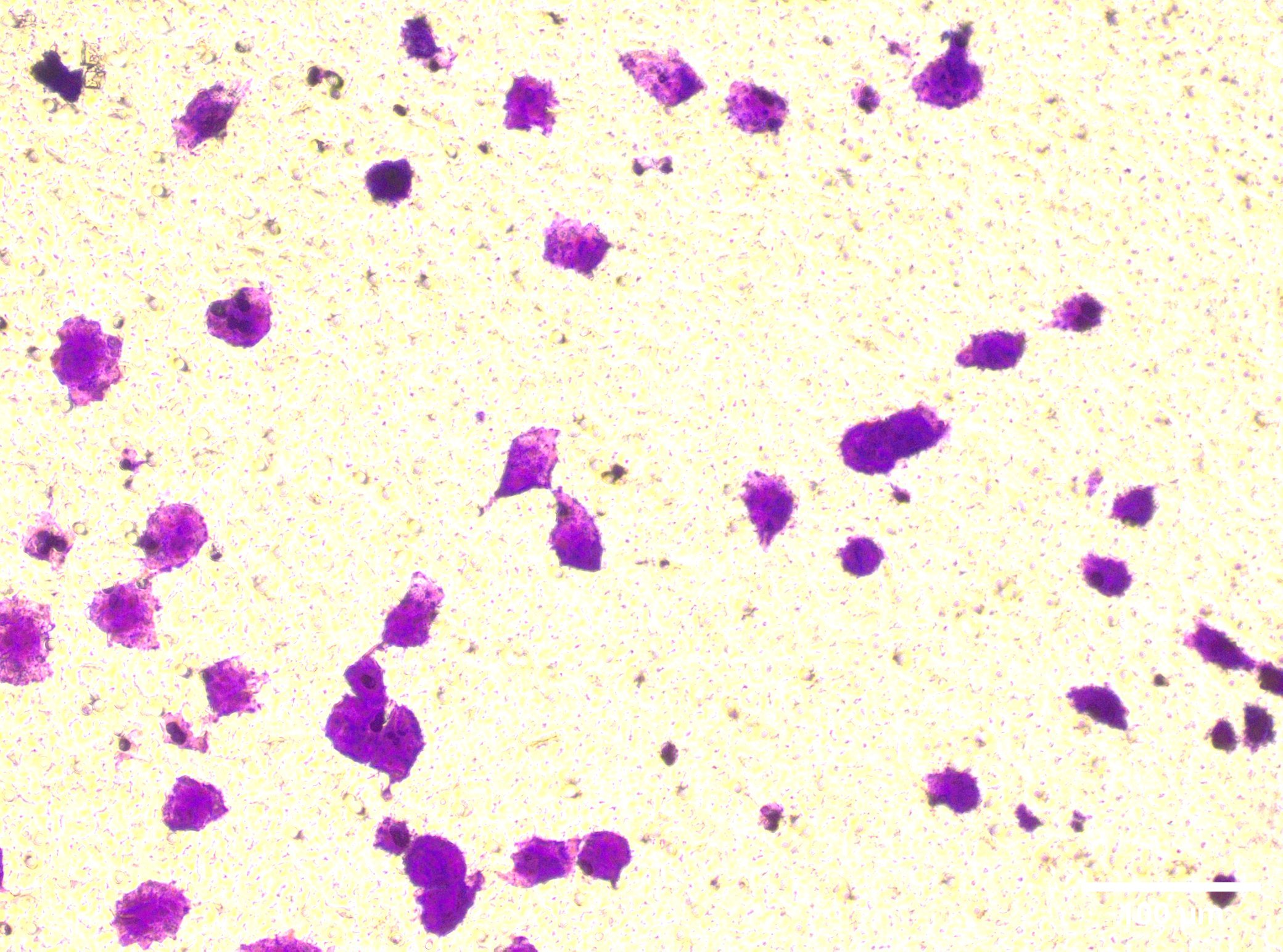

Supplement: Supplementary file 10 — EV Figure Source Data [file 44318_2026_766_MOESM10_ESM.zip › Figure EV4/Fig EV 4K/invasion/sh1 dox.jpg]

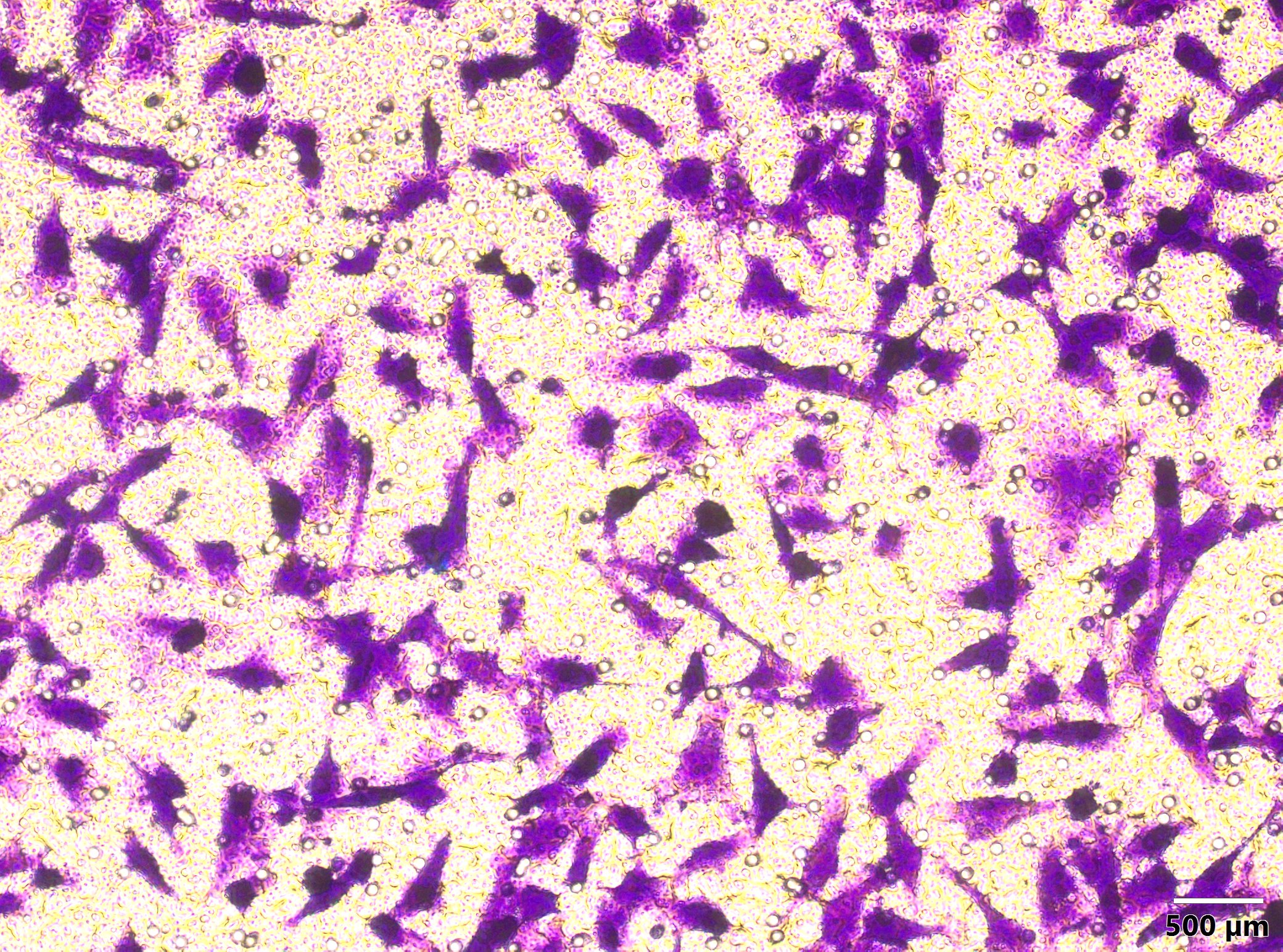

Supplement: Supplementary file 10 — EV Figure Source Data [file 44318_2026_766_MOESM10_ESM.zip › Figure EV4/Fig EV 4K/invasion/sh2 no.jpg]

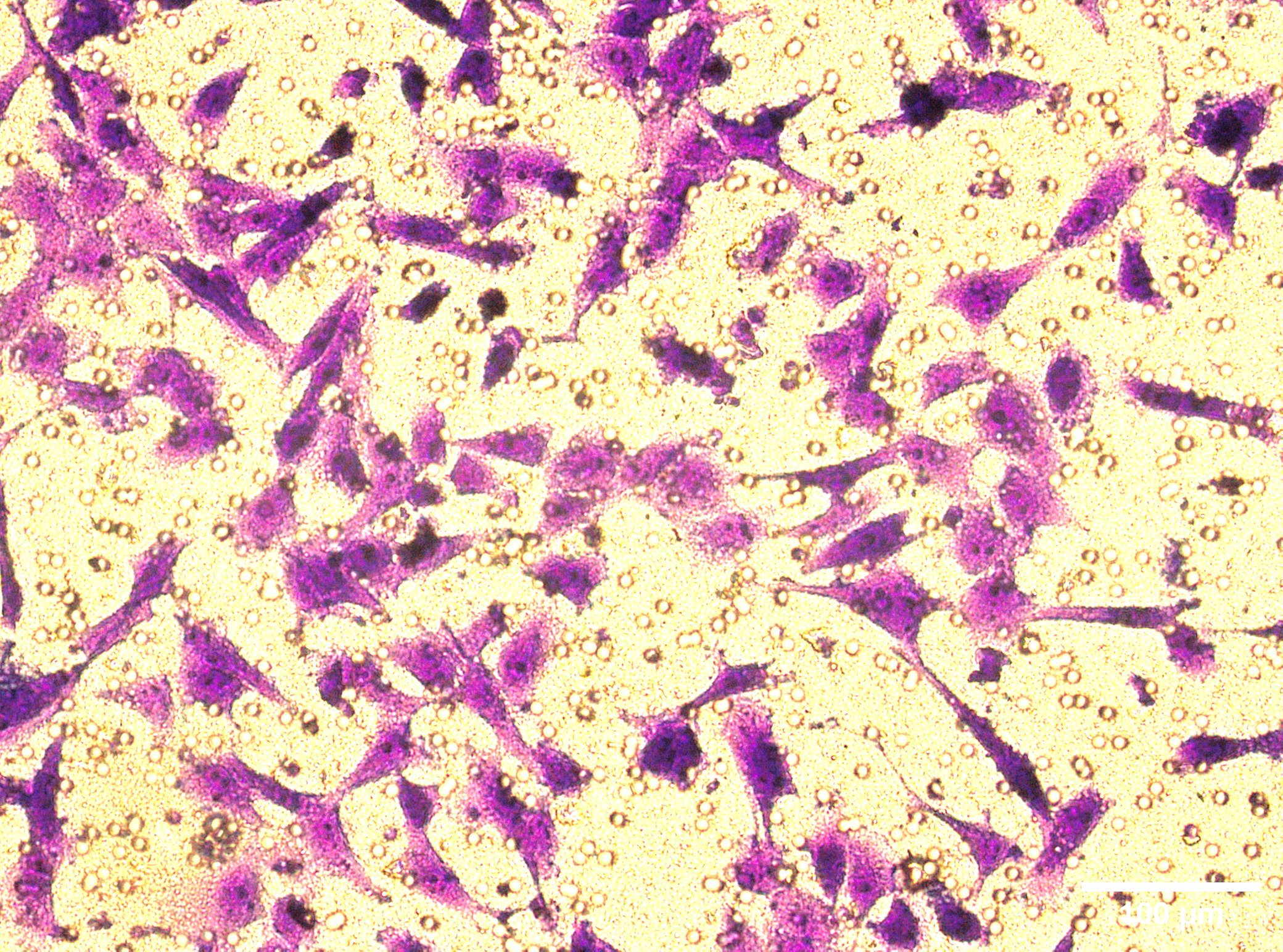

Supplement: Supplementary file 10 — EV Figure Source Data [file 44318_2026_766_MOESM10_ESM.zip › Figure EV4/Fig EV 4K/invasion/scramble no.jpg]

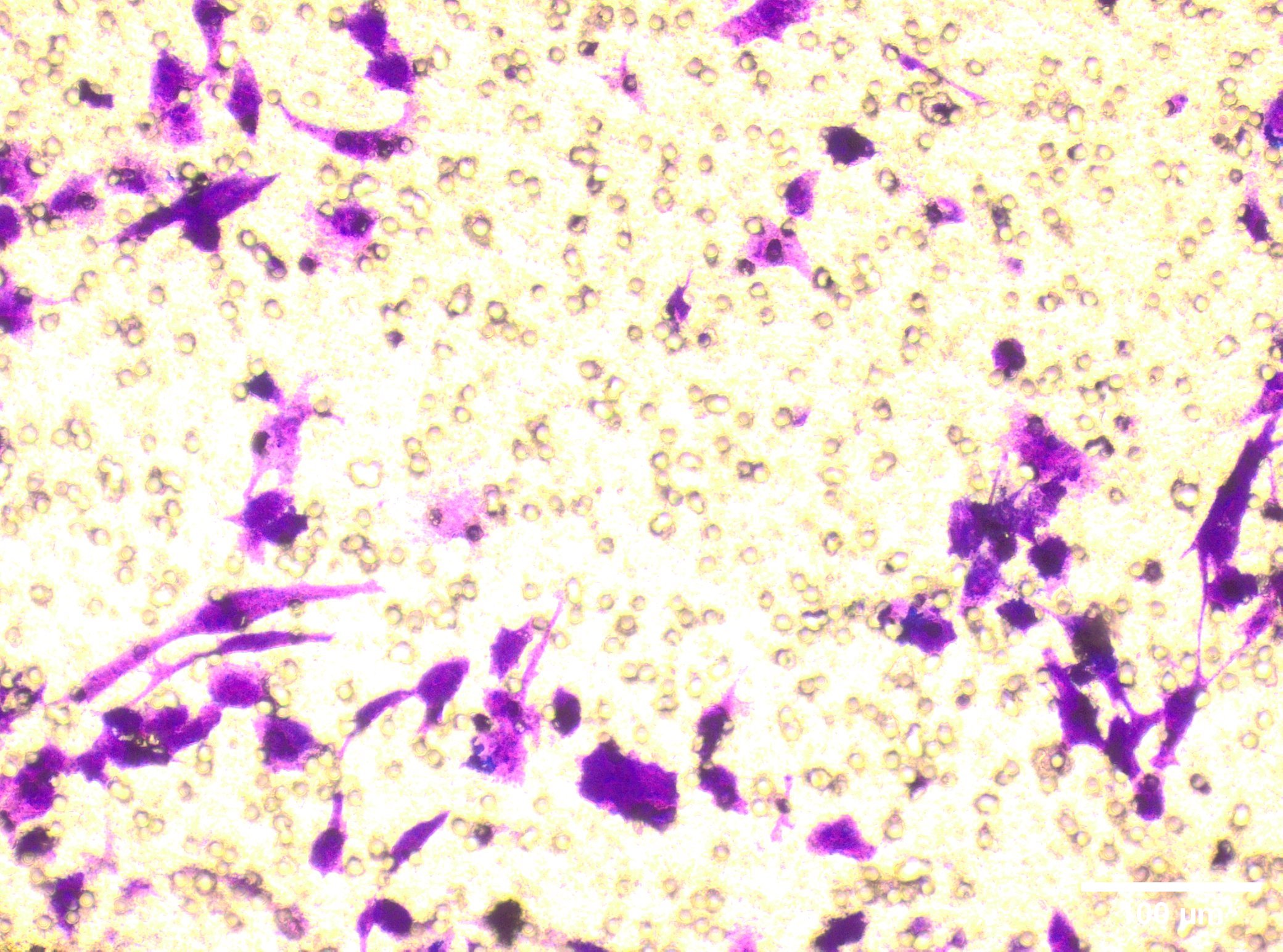

Supplement: Supplementary file 10 — EV Figure Source Data [file 44318_2026_766_MOESM10_ESM.zip › Figure EV4/Fig EV 4K/invasion/sh2 dox.jpg]

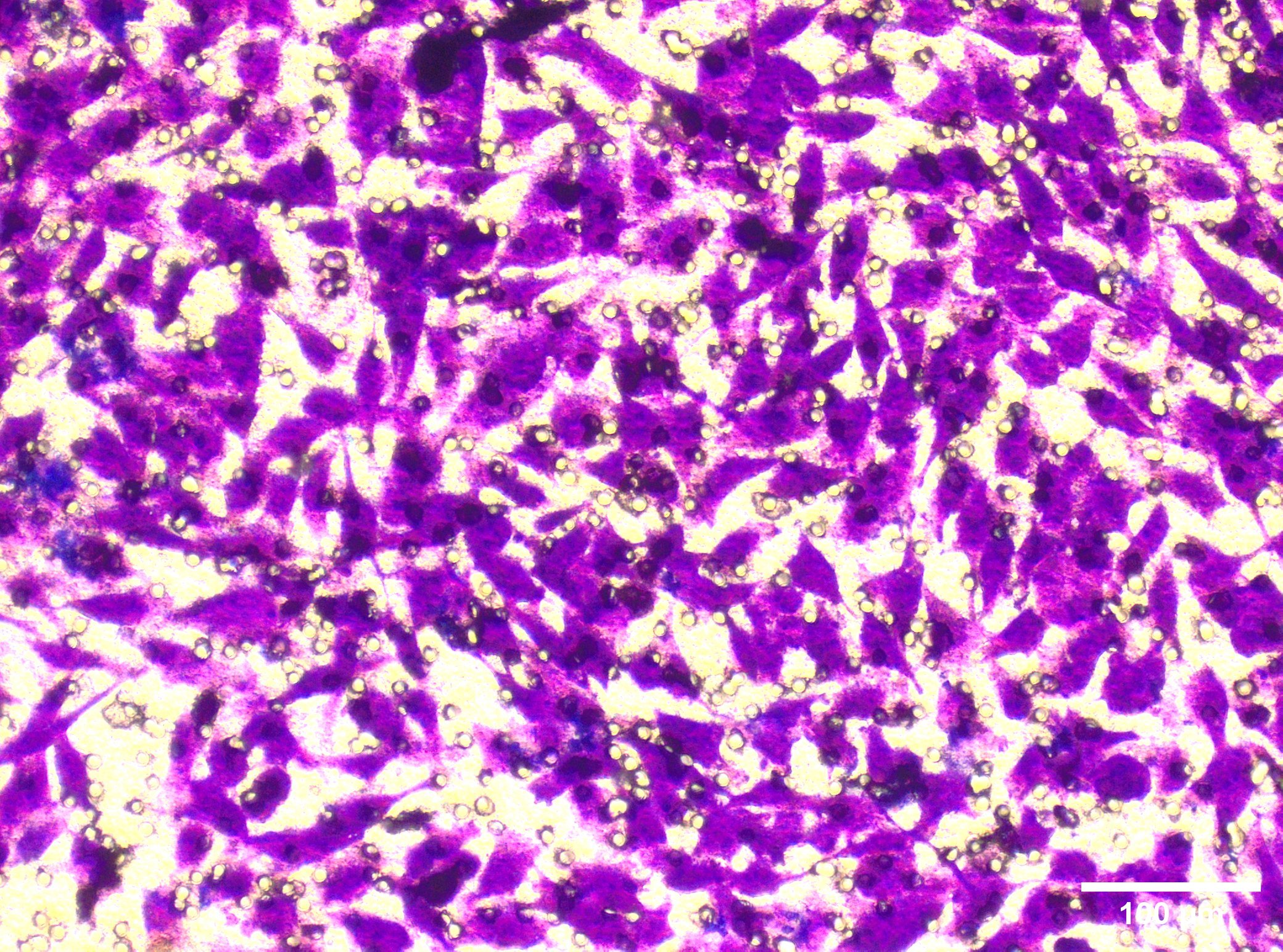

Supplement: Supplementary file 10 — EV Figure Source Data [file 44318_2026_766_MOESM10_ESM.zip › Figure EV4/Fig EV 4K/migation/sh1 no.jpg]

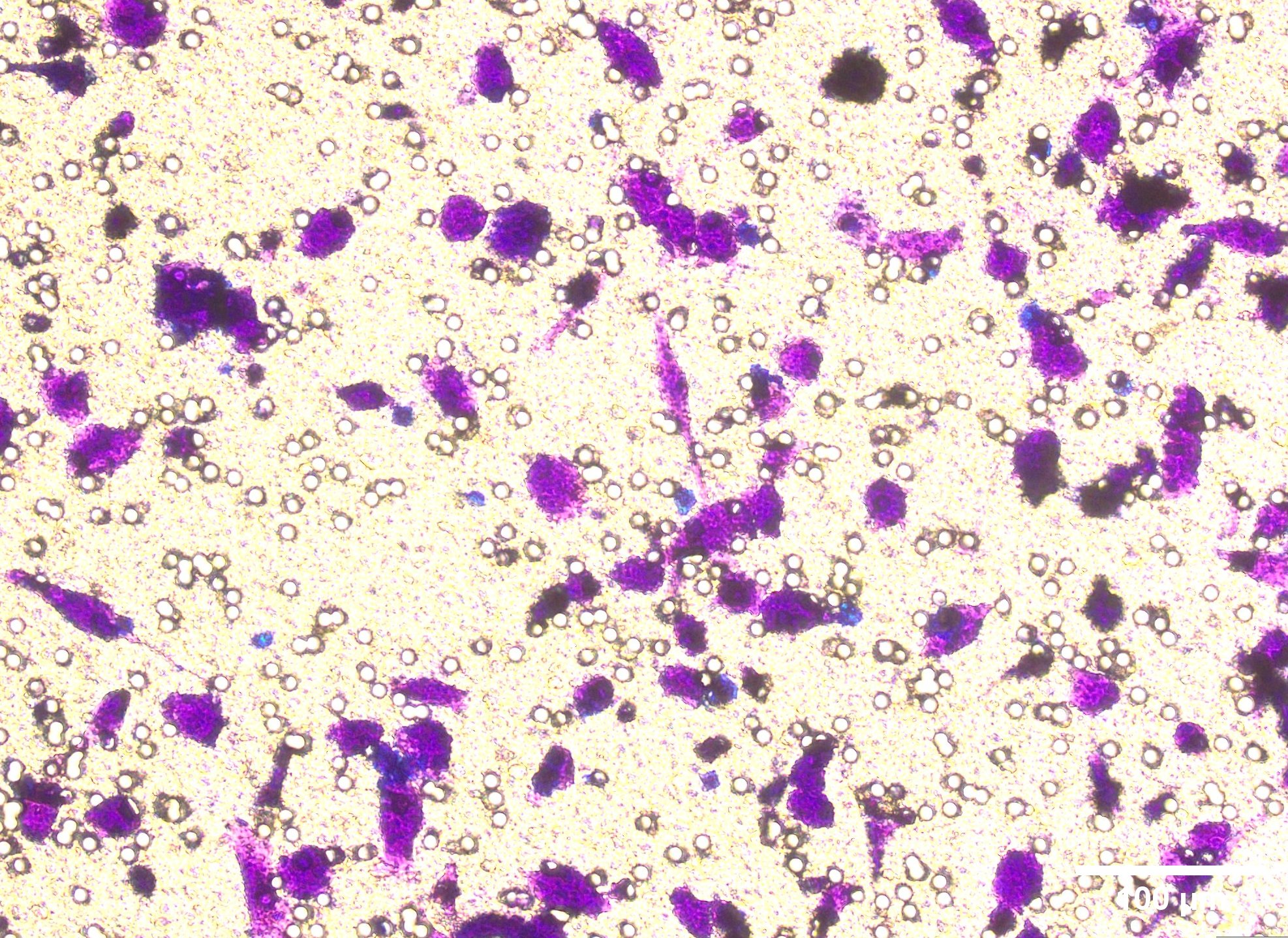

Supplement: Supplementary file 10 — EV Figure Source Data [file 44318_2026_766_MOESM10_ESM.zip › Figure EV4/Fig EV 4K/migation/sh1 dox.jpg]

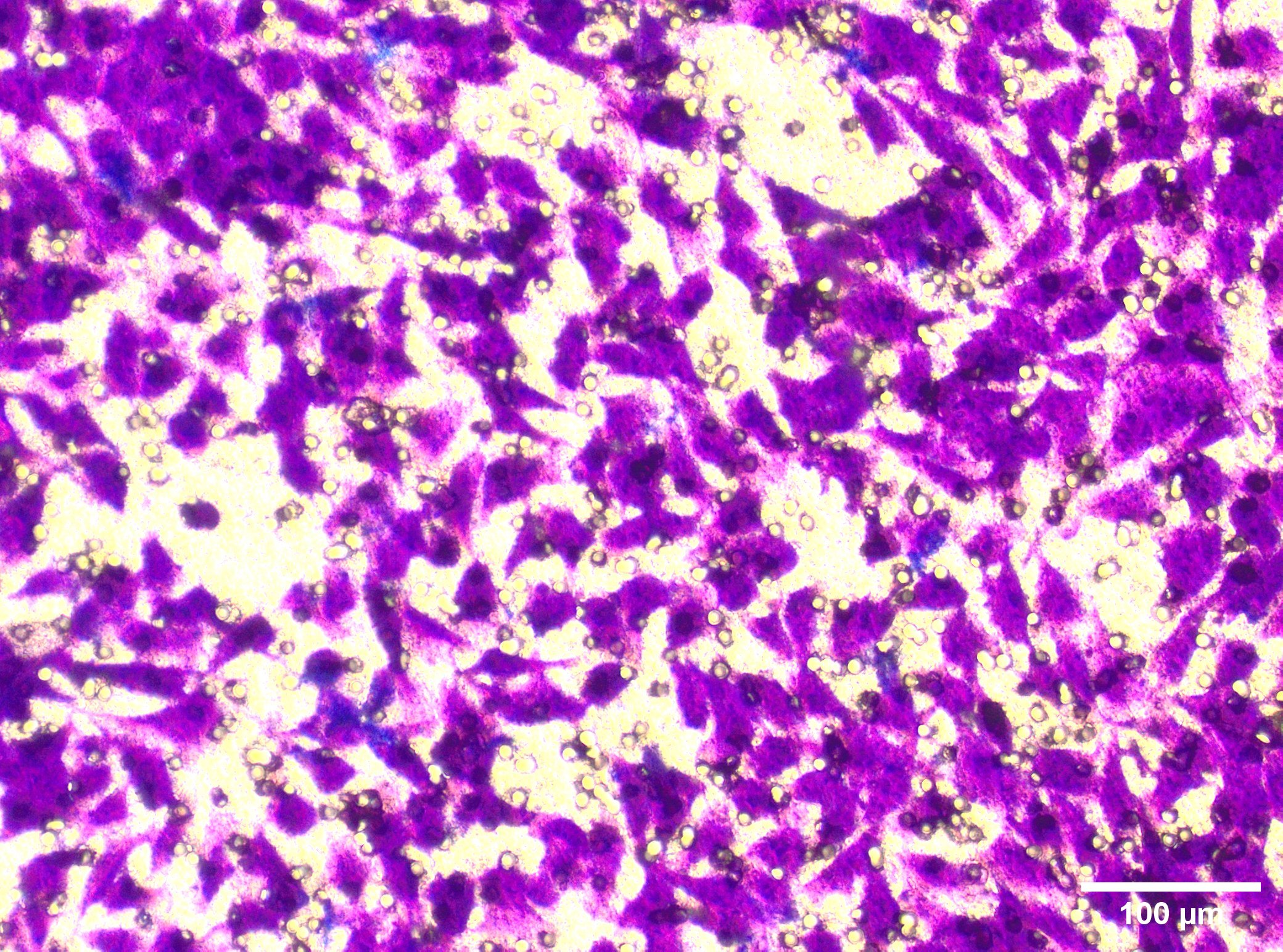

Supplement: Supplementary file 10 — EV Figure Source Data [file 44318_2026_766_MOESM10_ESM.zip › Figure EV4/Fig EV 4K/migation/sh2 no.jpg]

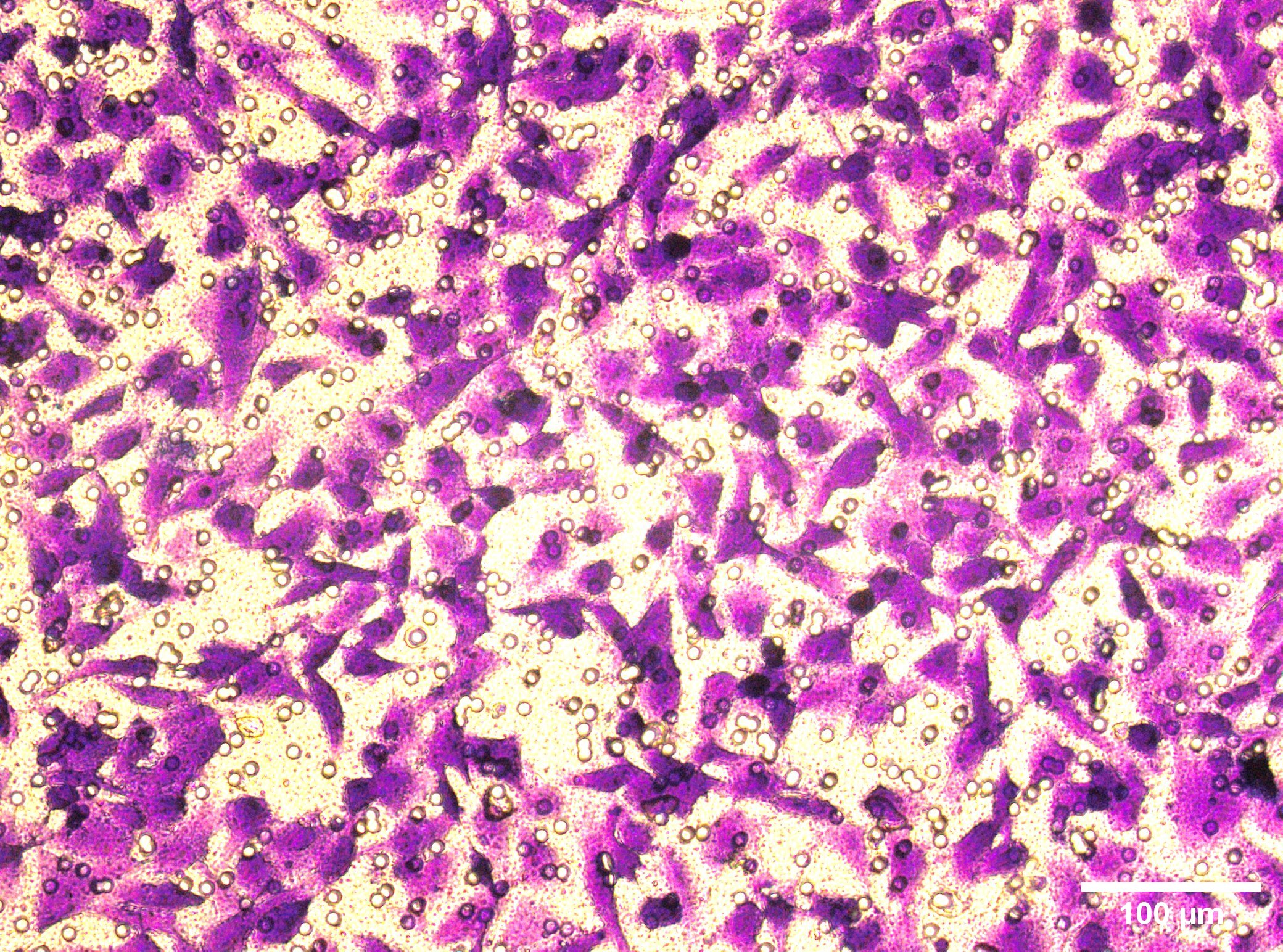

Supplement: Supplementary file 10 — EV Figure Source Data [file 44318_2026_766_MOESM10_ESM.zip › Figure EV4/Fig EV 4K/migation/scramble no.jpg]

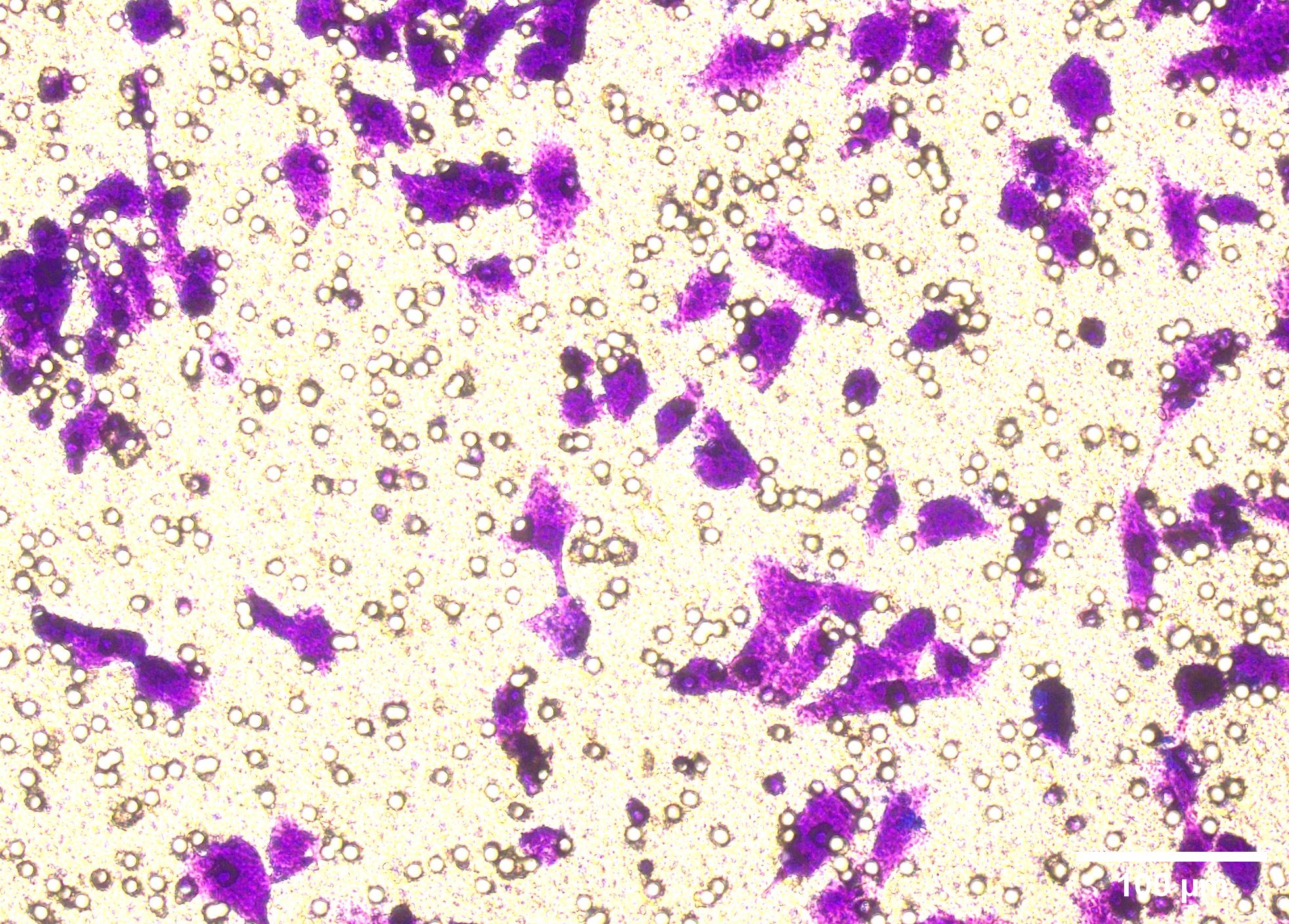

Supplement: Supplementary file 10 — EV Figure Source Data [file 44318_2026_766_MOESM10_ESM.zip › Figure EV4/Fig EV 4K/migation/sh2 dox.jpg]

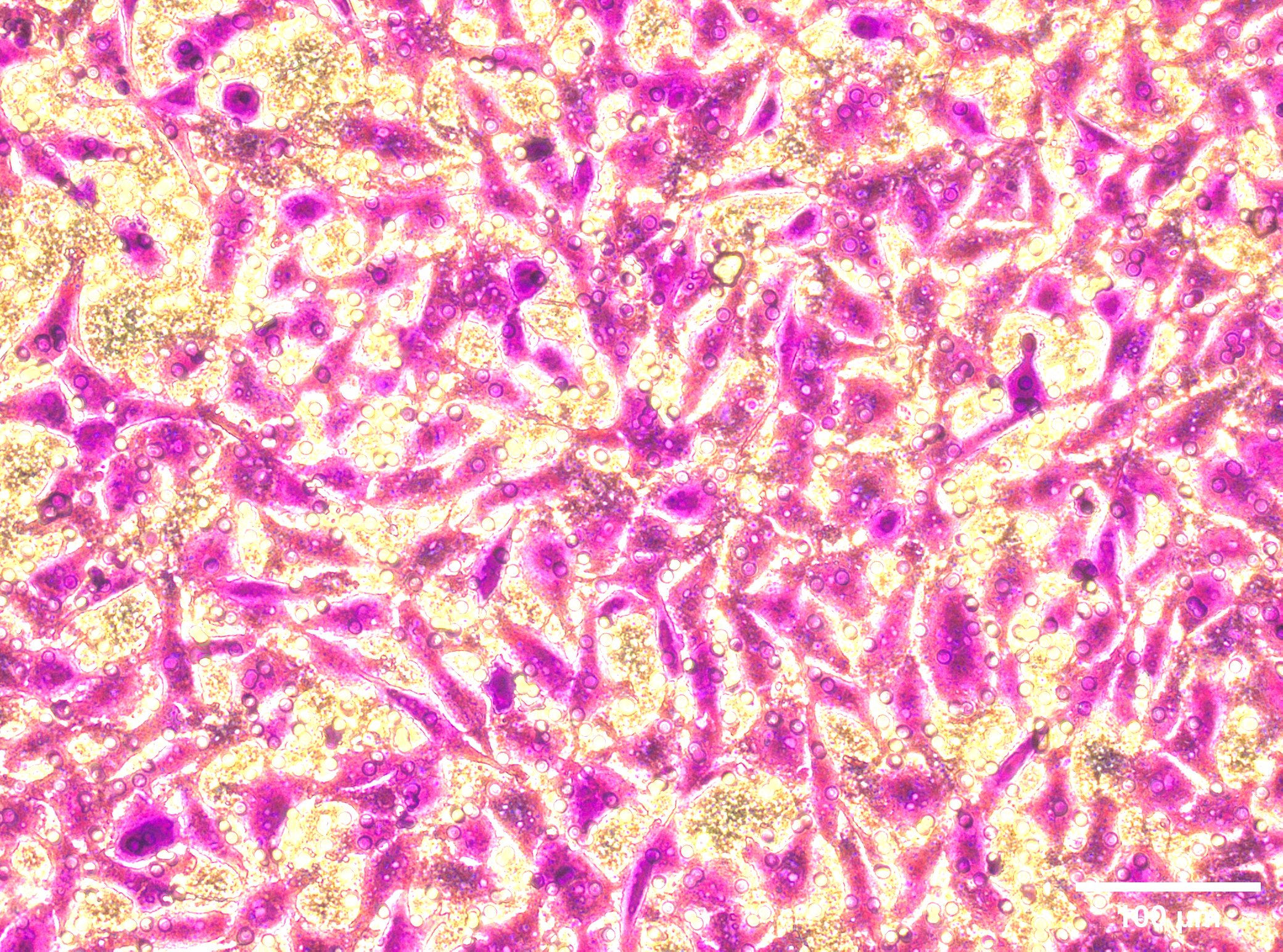

Supplement: Supplementary file 10 — EV Figure Source Data [file 44318_2026_766_MOESM10_ESM.zip › Figure EV4/Fig EV 4K/migation/scamble dox.jpg]

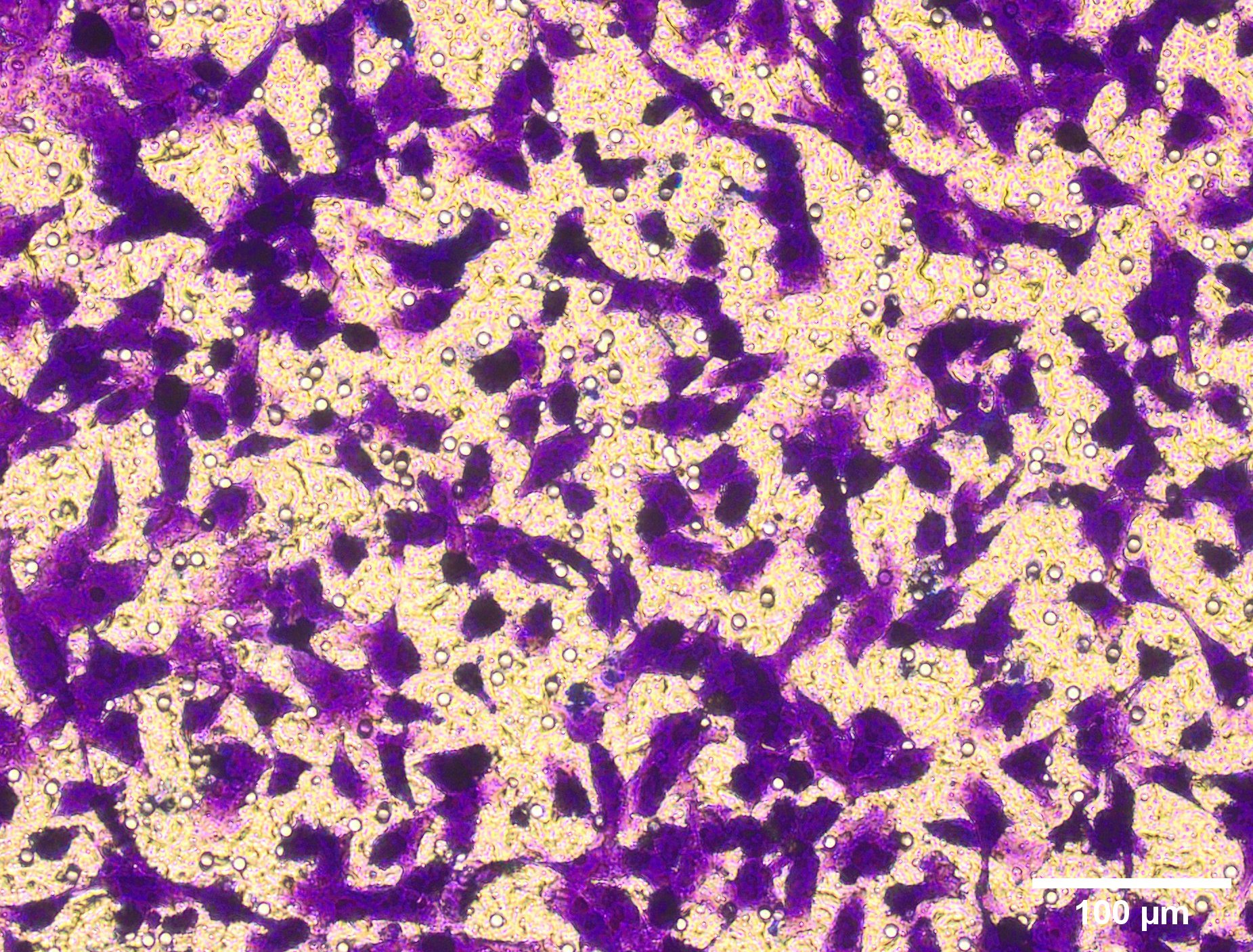

Supplement: Supplementary file 10 — EV Figure Source Data [file 44318_2026_766_MOESM10_ESM.zip › Figure EV4/Fig EV 4E/invasion/_10112efemp1 invasion-1.jpg]

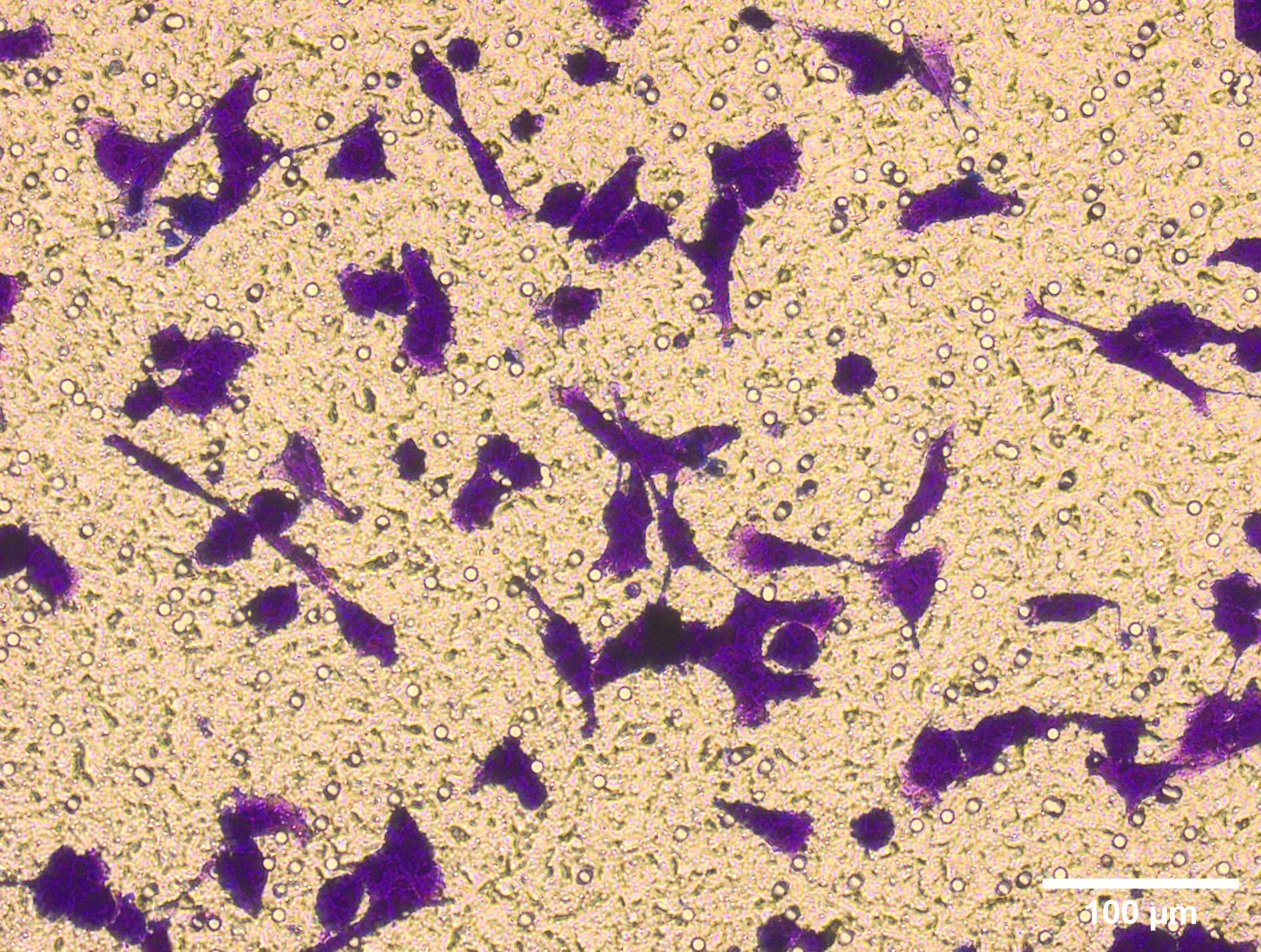

Supplement: Supplementary file 10 — EV Figure Source Data [file 44318_2026_766_MOESM10_ESM.zip › Figure EV4/Fig EV 4E/invasion/_10109vector invasion-1.jpg]

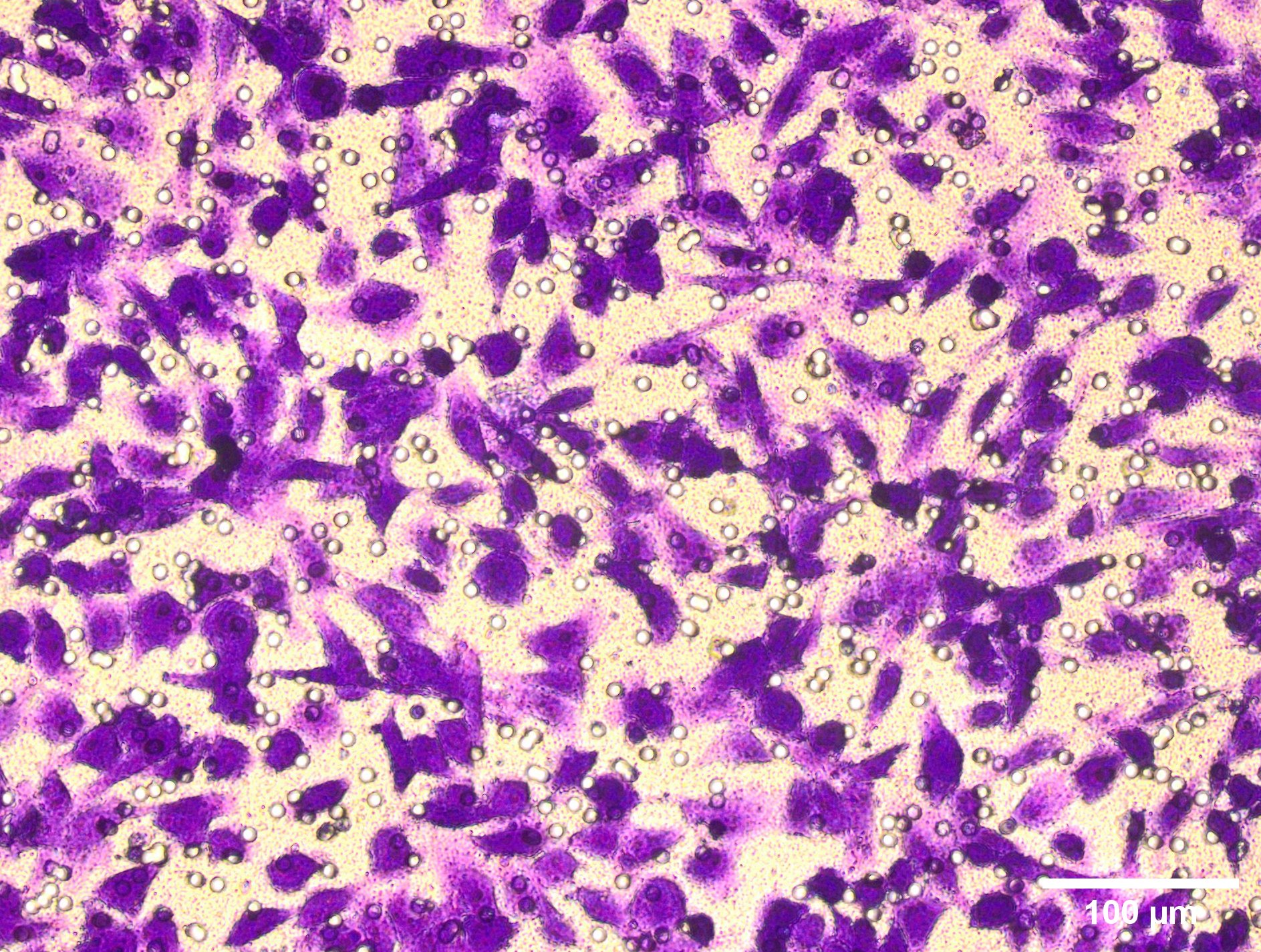

Supplement: Supplementary file 10 — EV Figure Source Data [file 44318_2026_766_MOESM10_ESM.zip › Figure EV4/Fig EV 4E/migration/_10099efemp1 migration-1.jpg]

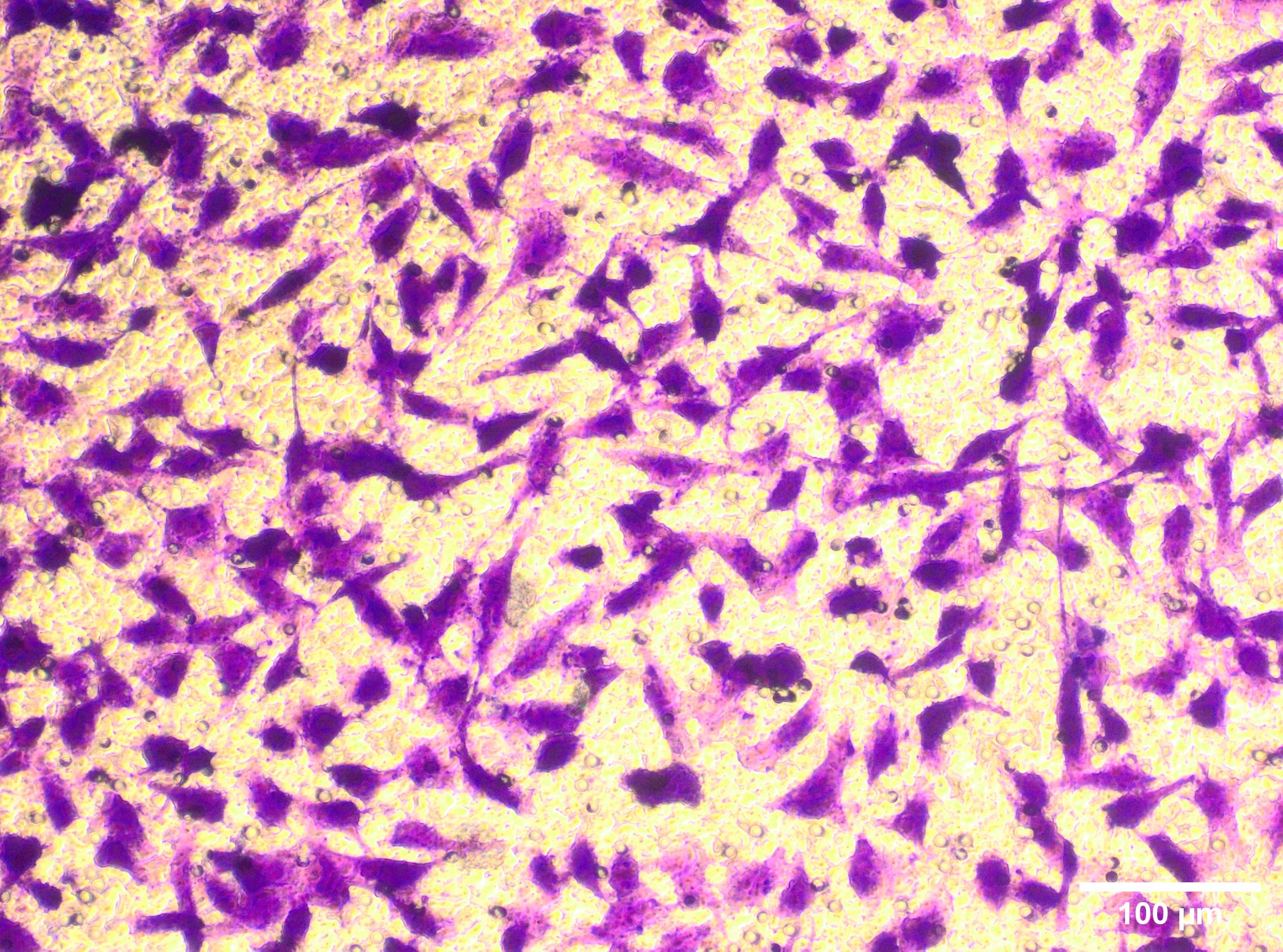

Supplement: Supplementary file 10 — EV Figure Source Data [file 44318_2026_766_MOESM10_ESM.zip › Figure EV4/Fig EV 4E/migration/Image_320vector migration.jpg]

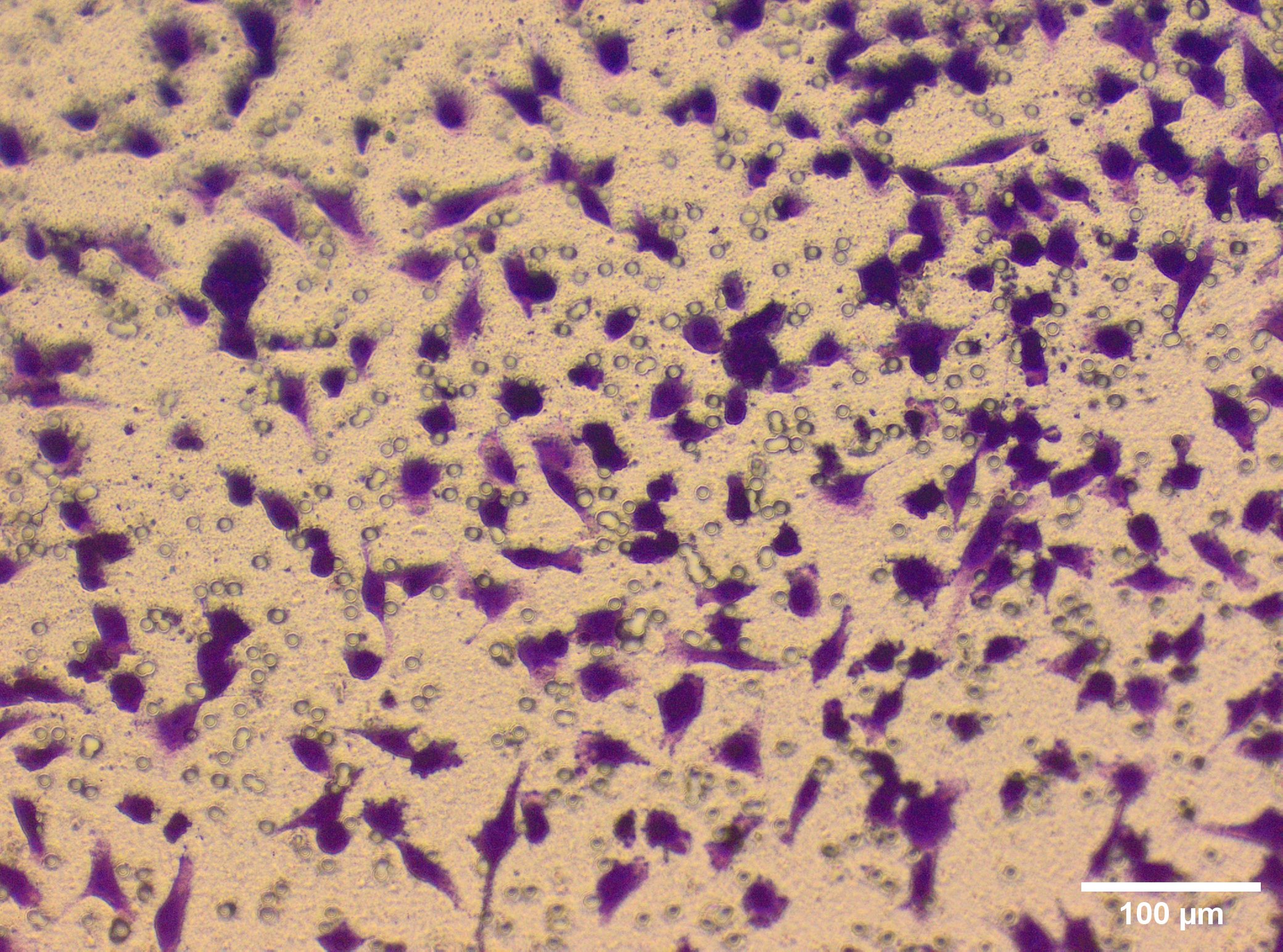

Supplement: Supplementary file 10 — EV Figure Source Data [file 44318_2026_766_MOESM10_ESM.zip › Figure EV4/Fig EV 4M/Invasion/mda scramble no.jpg]

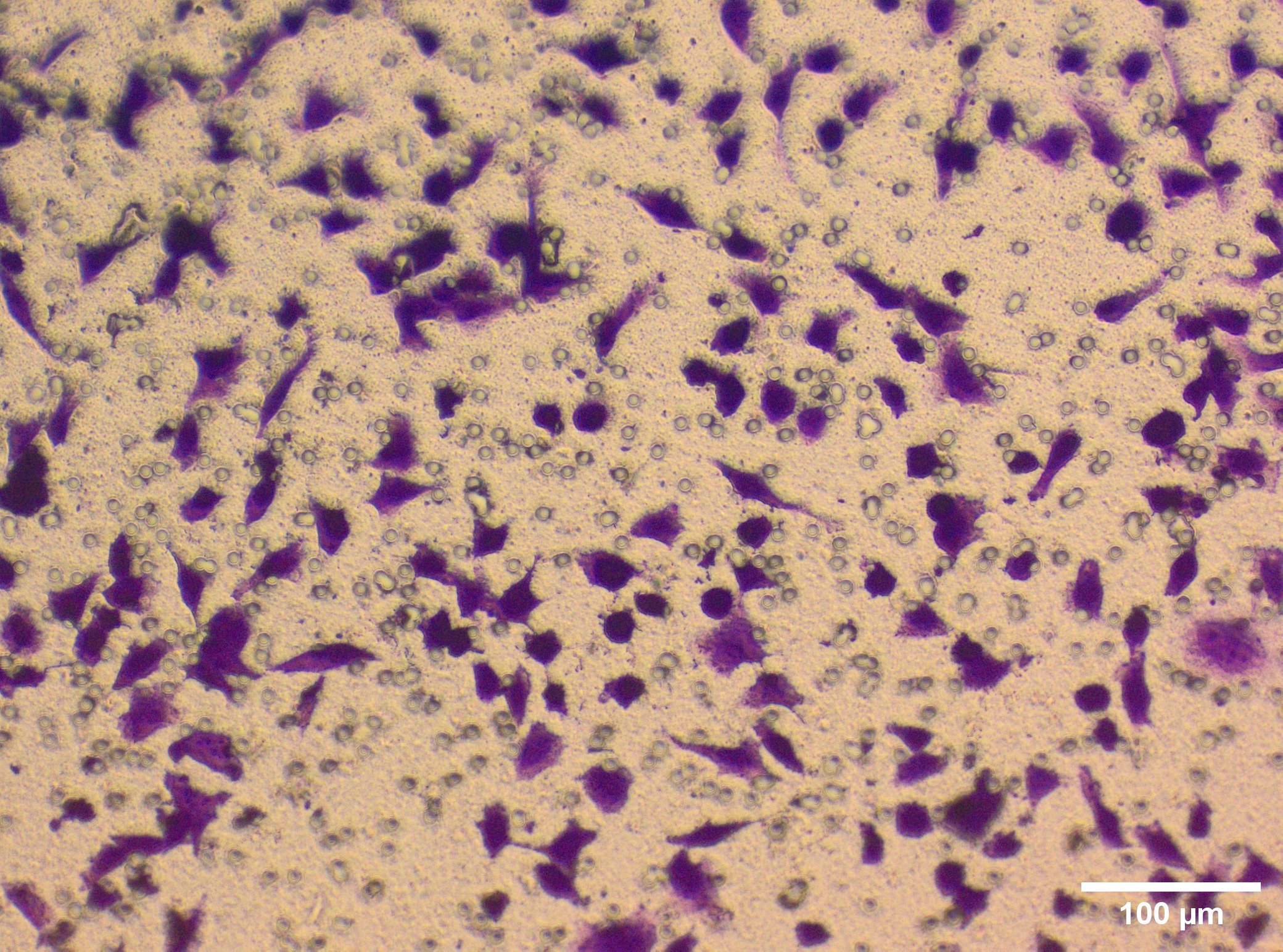

Supplement: Supplementary file 10 — EV Figure Source Data [file 44318_2026_766_MOESM10_ESM.zip › Figure EV4/Fig EV 4M/Invasion/mda sh5 no invaImage_6471.jpg]

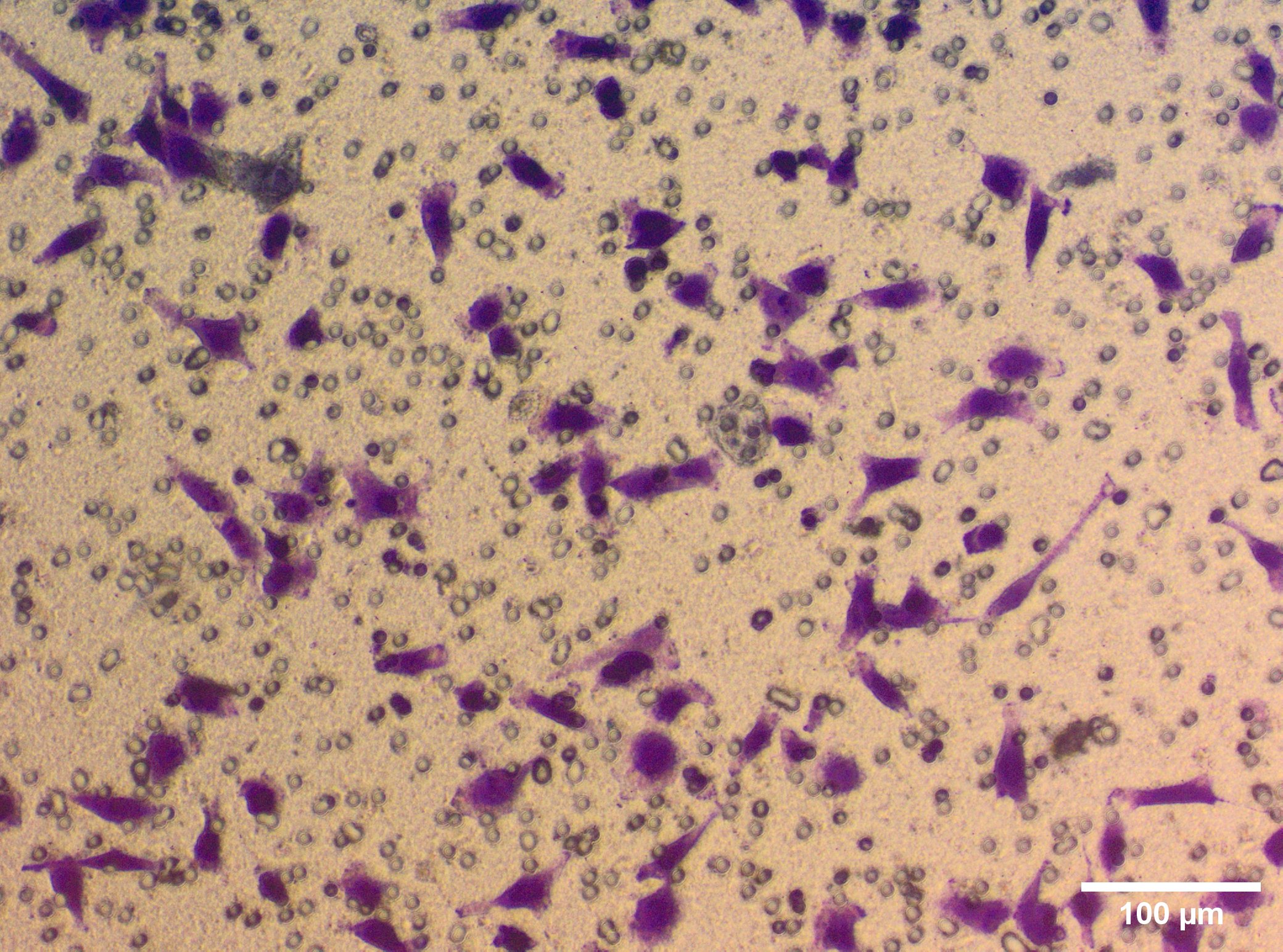

Supplement: Supplementary file 10 — EV Figure Source Data [file 44318_2026_766_MOESM10_ESM.zip › Figure EV4/Fig EV 4M/Invasion/mda sh2 dox.jpg]

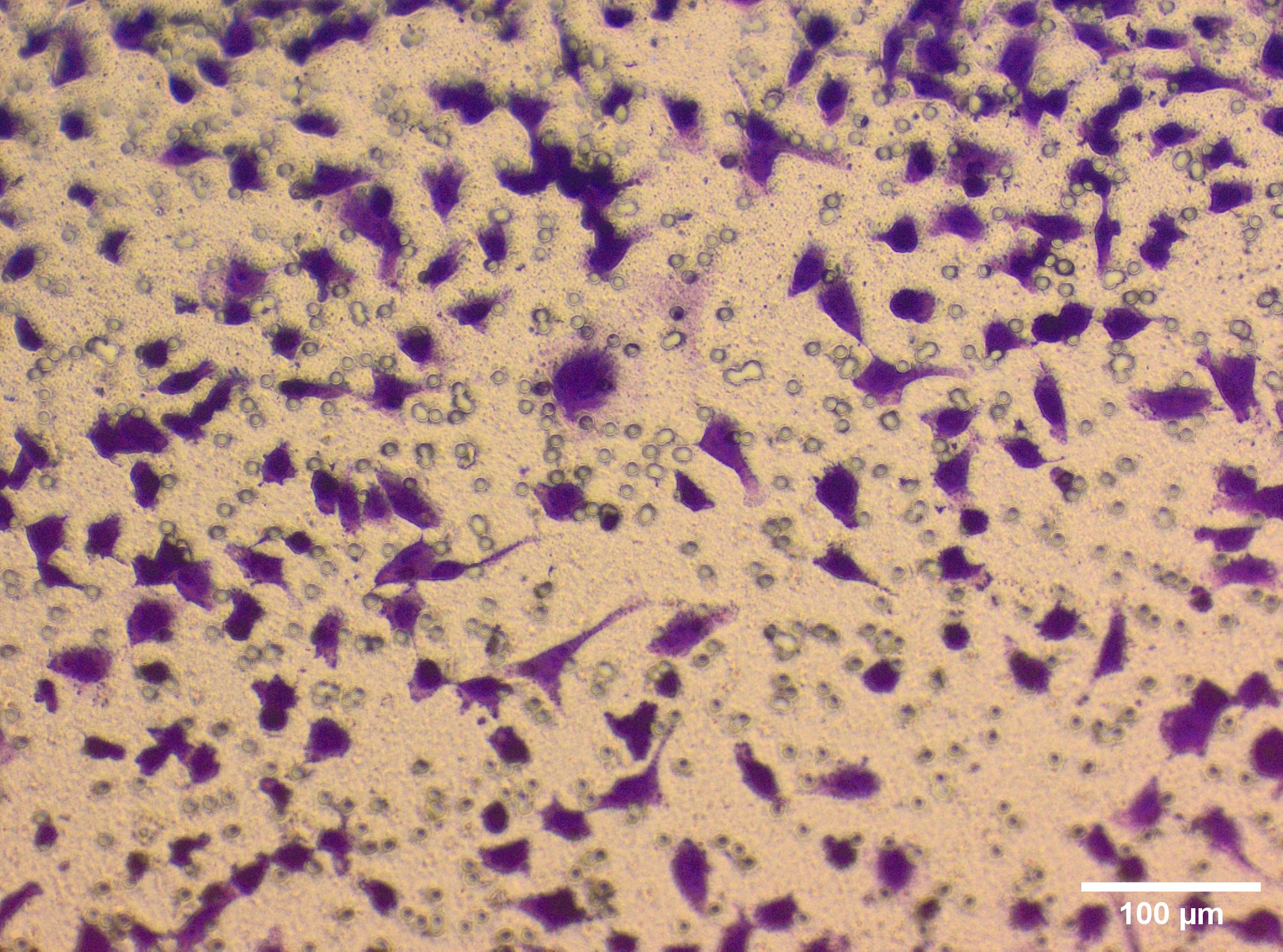

Supplement: Supplementary file 10 — EV Figure Source Data [file 44318_2026_766_MOESM10_ESM.zip › Figure EV4/Fig EV 4M/Invasion/mda scramble dox.jpg]

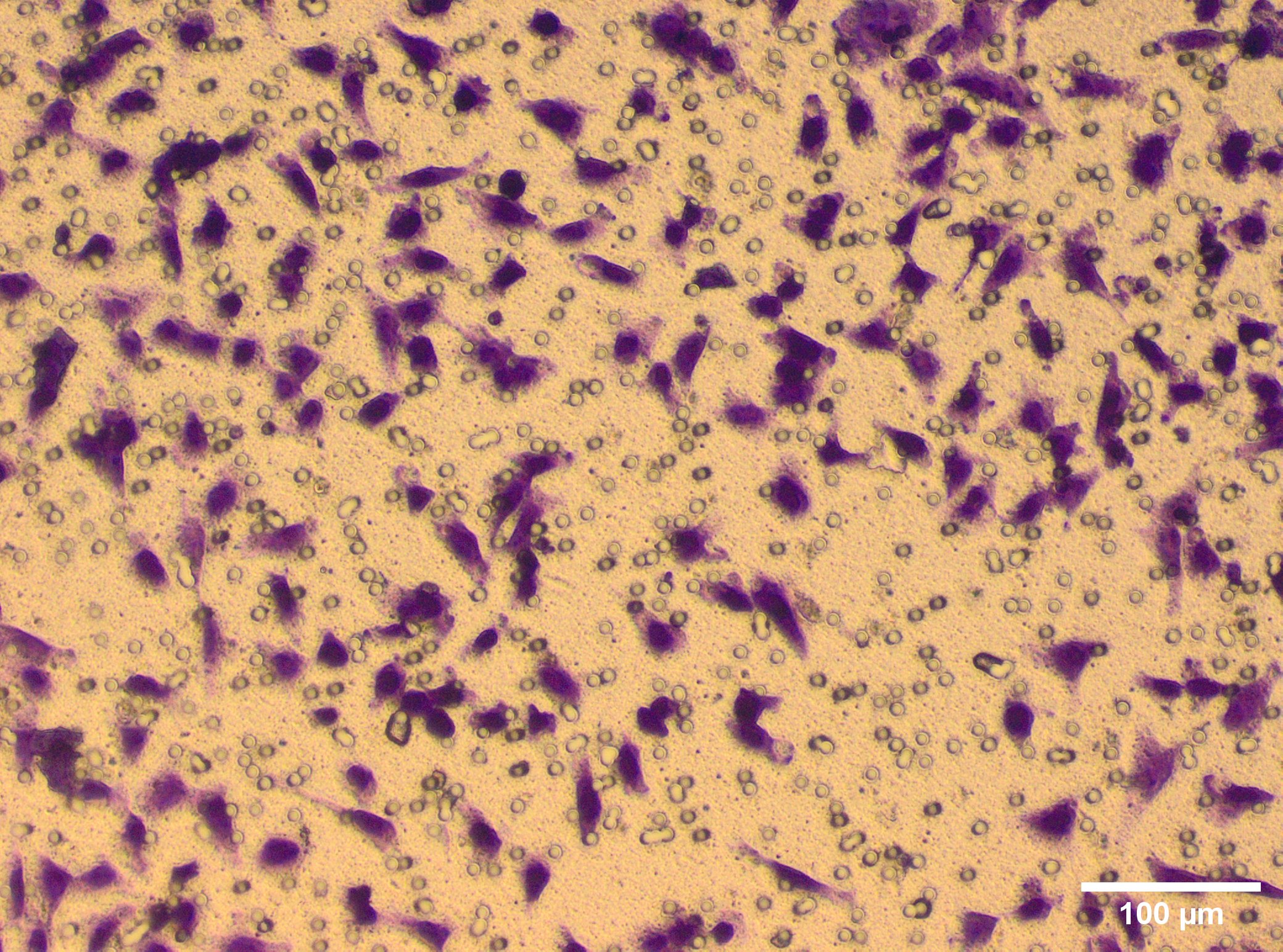

Supplement: Supplementary file 10 — EV Figure Source Data [file 44318_2026_766_MOESM10_ESM.zip › Figure EV4/Fig EV 4M/Invasion/mda sh1 no.jpg]

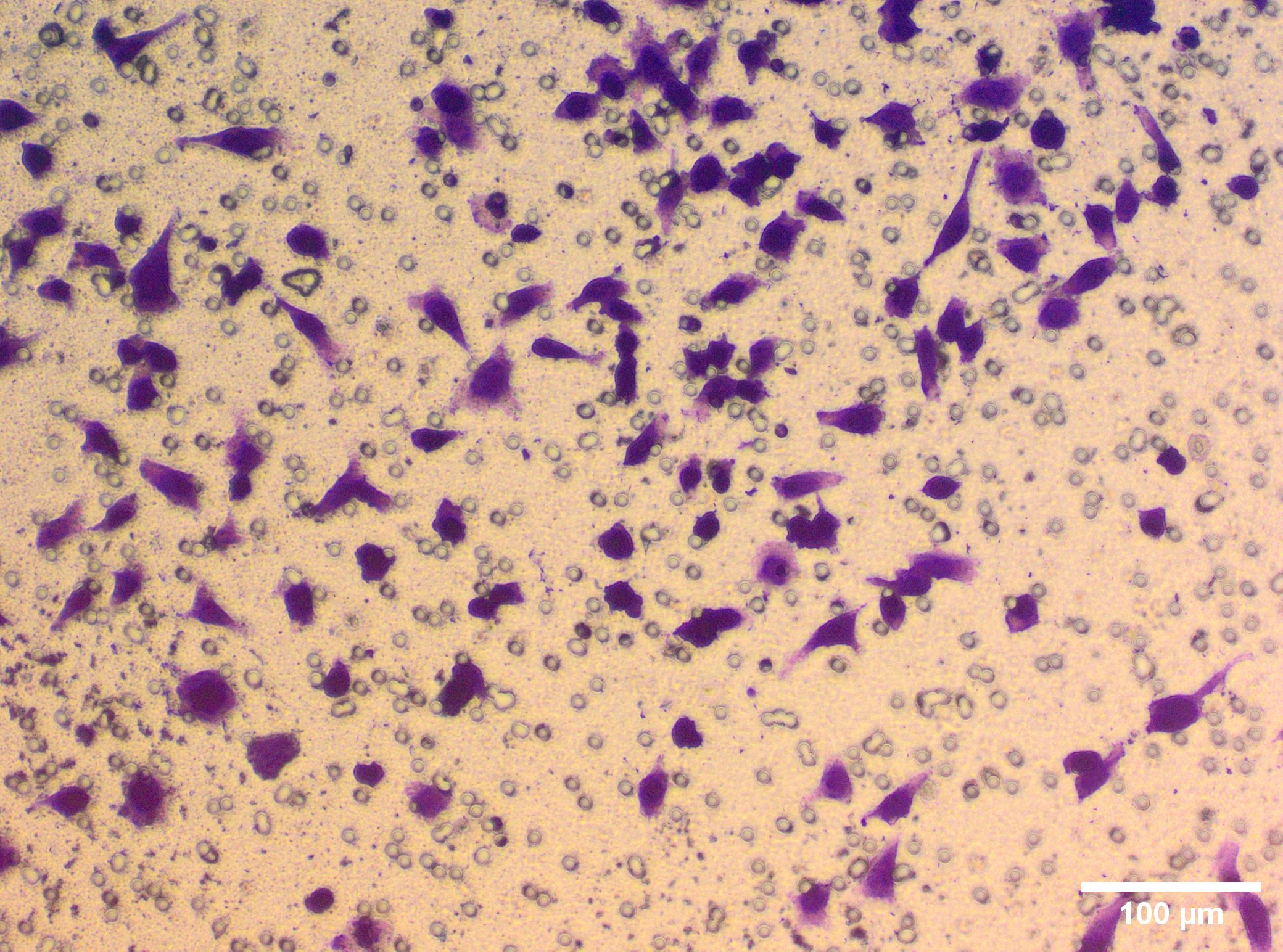

Supplement: Supplementary file 10 — EV Figure Source Data [file 44318_2026_766_MOESM10_ESM.zip › Figure EV4/Fig EV 4M/Invasion/mda sh1 dox.jpg]

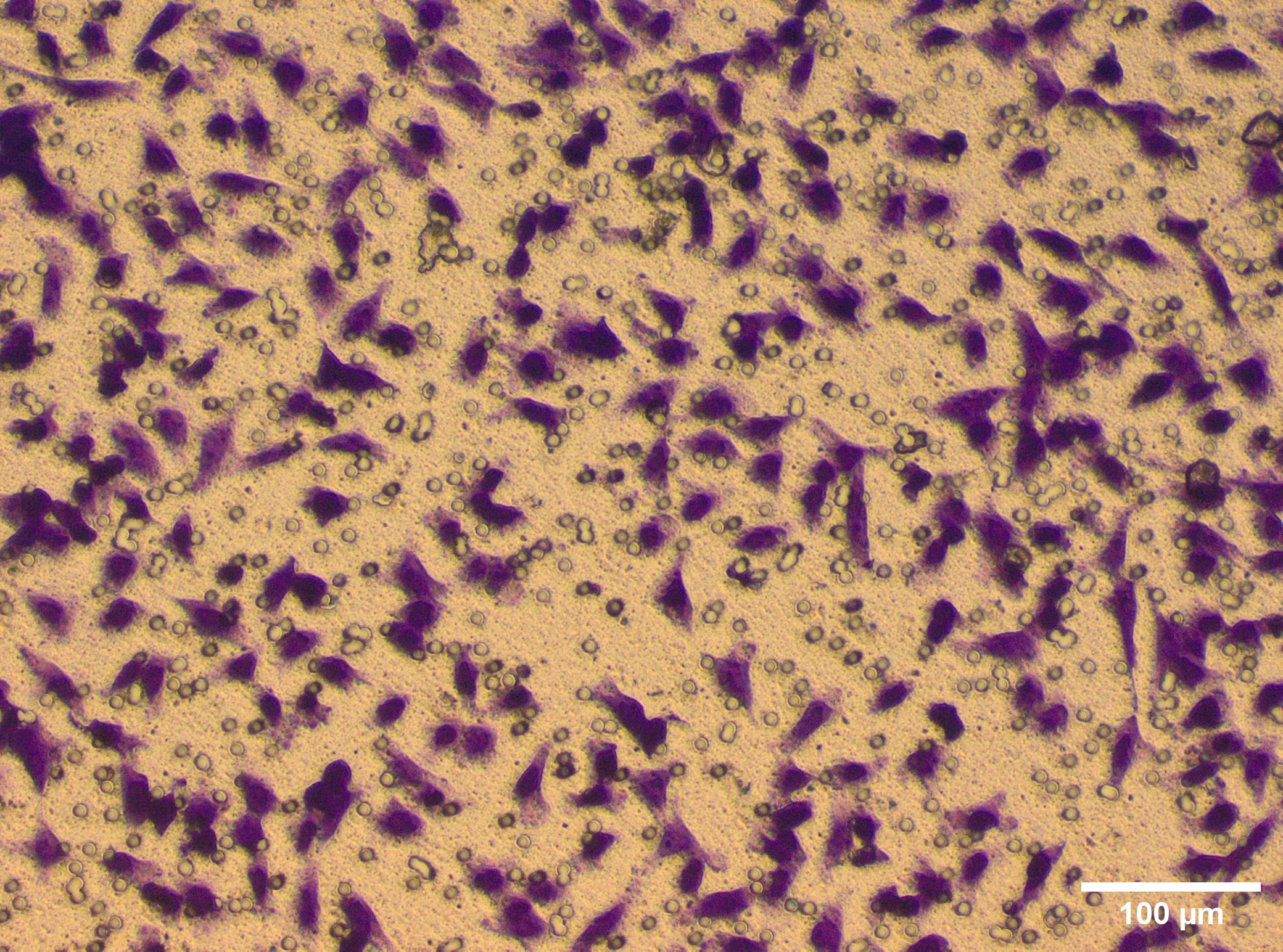

Supplement: Supplementary file 10 — EV Figure Source Data [file 44318_2026_766_MOESM10_ESM.zip › Figure EV4/Fig EV 4M/migration/MDA sh1 dox.jpg]

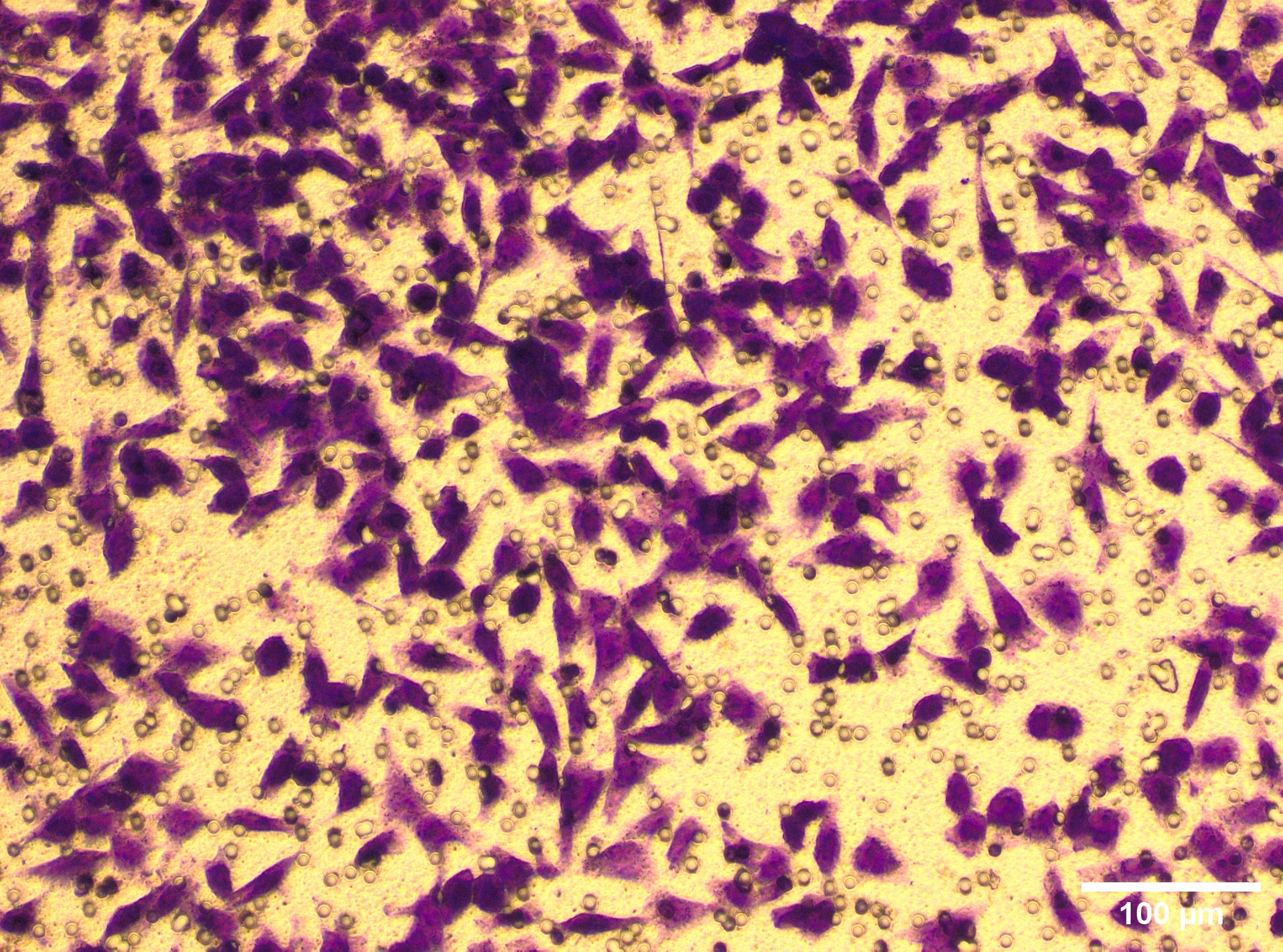

Supplement: Supplementary file 10 — EV Figure Source Data [file 44318_2026_766_MOESM10_ESM.zip › Figure EV4/Fig EV 4M/migration/mda scramble no.jpg]

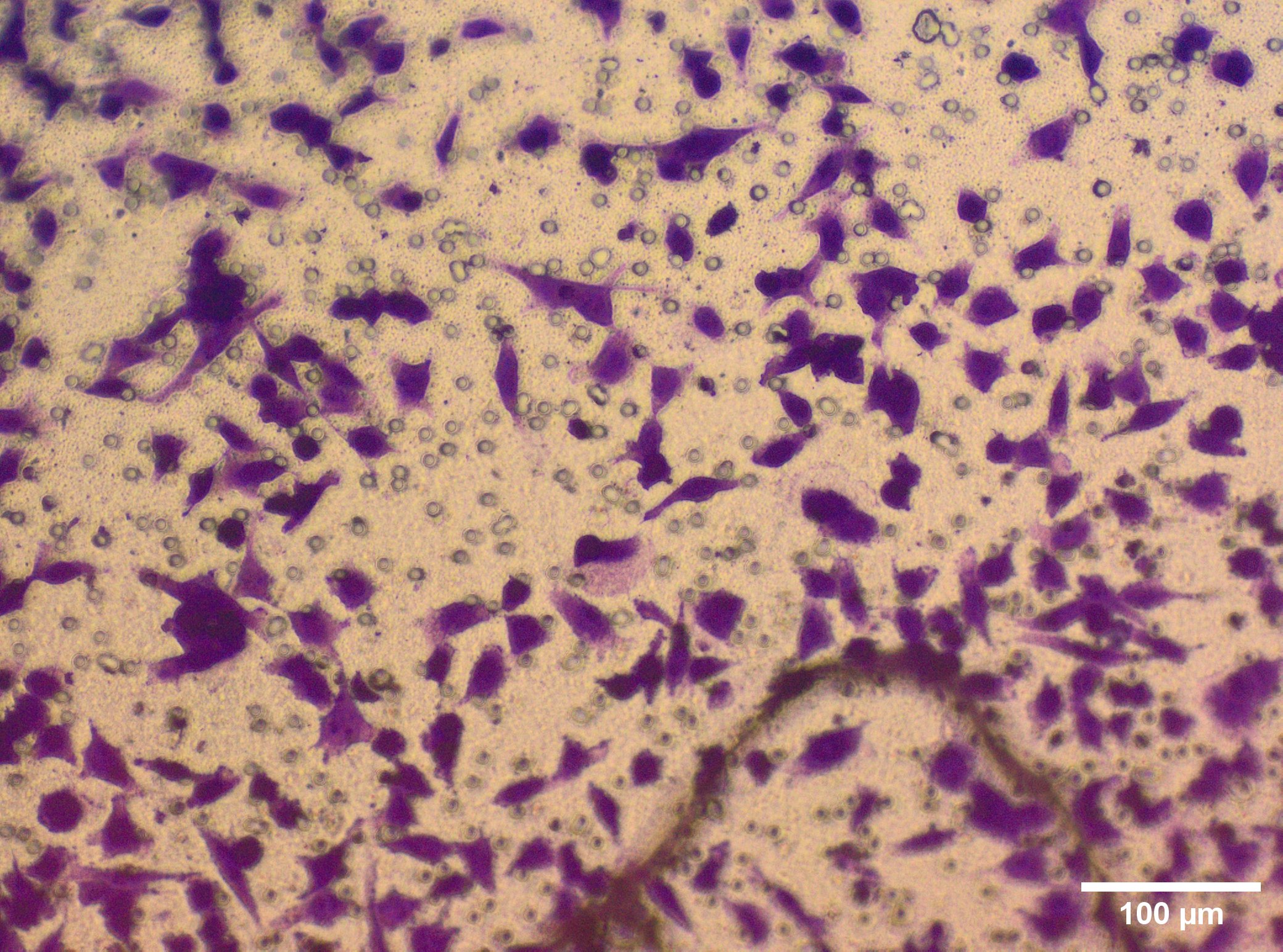

Supplement: Supplementary file 10 — EV Figure Source Data [file 44318_2026_766_MOESM10_ESM.zip › Figure EV4/Fig EV 4M/migration/mda sh2 no.jpg]

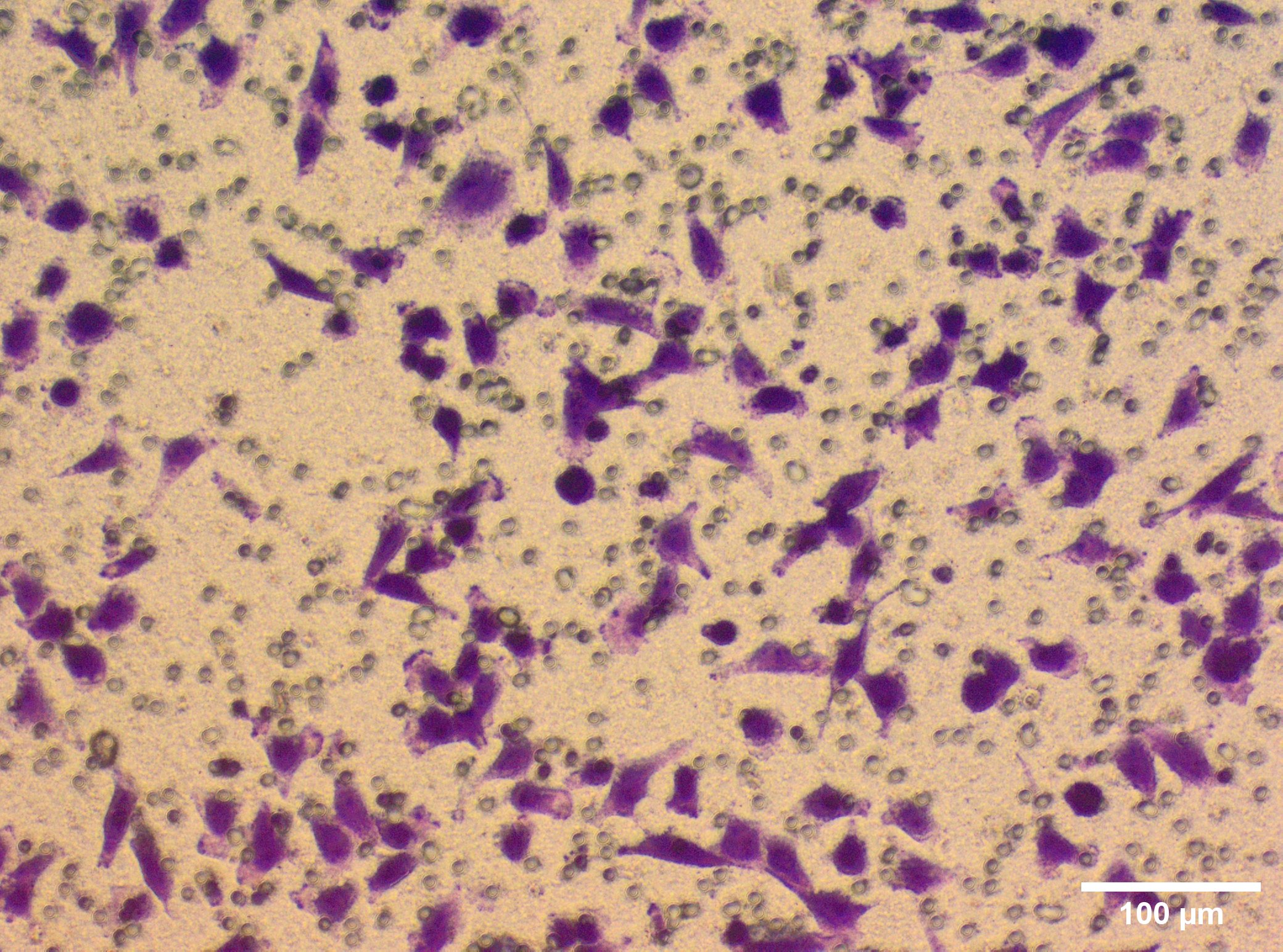

Supplement: Supplementary file 10 — EV Figure Source Data [file 44318_2026_766_MOESM10_ESM.zip › Figure EV4/Fig EV 4M/migration/mda sh2 dox.jpg]

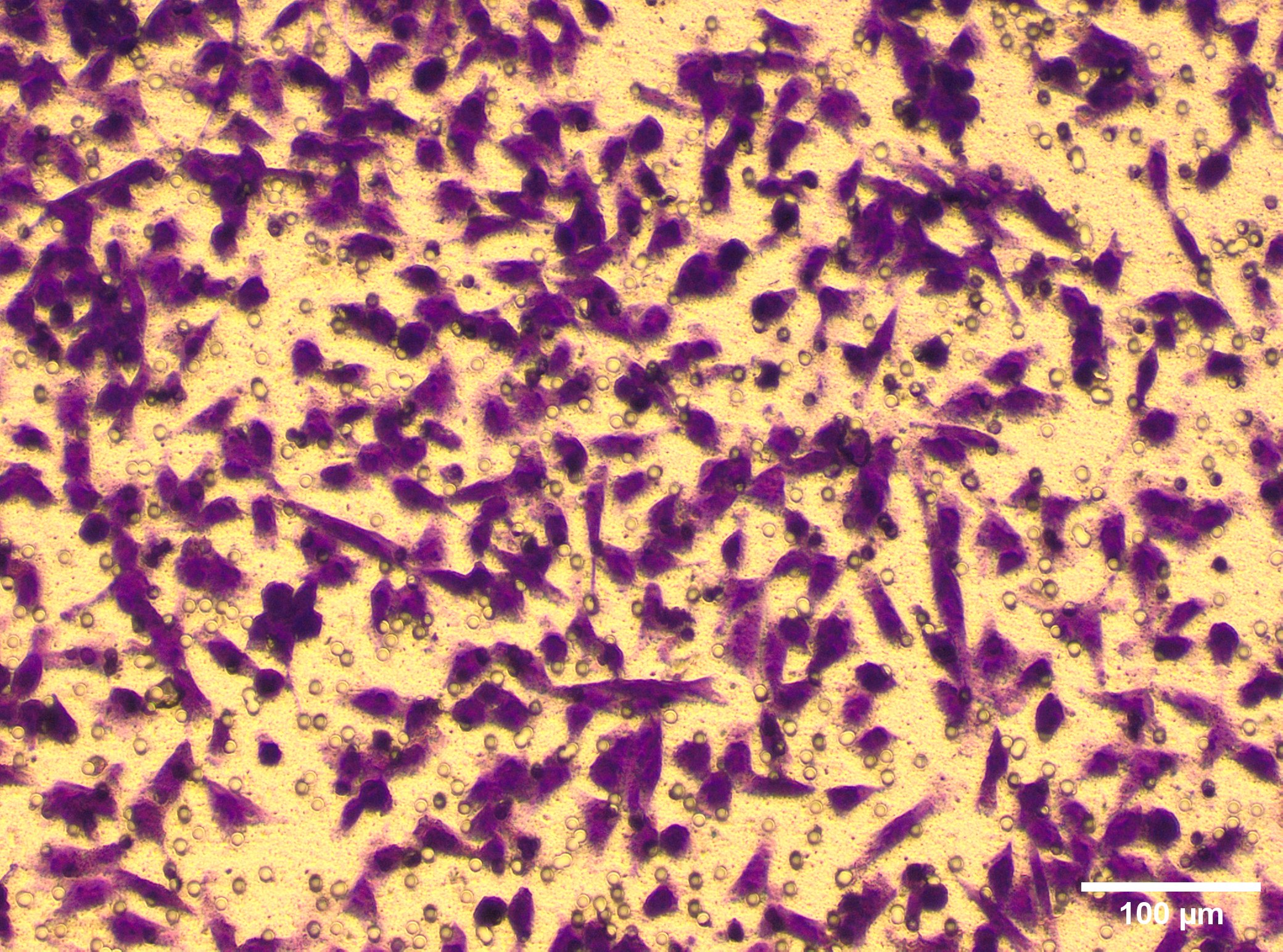

Supplement: Supplementary file 10 — EV Figure Source Data [file 44318_2026_766_MOESM10_ESM.zip › Figure EV4/Fig EV 4M/migration/mda scramble dox.jpg]

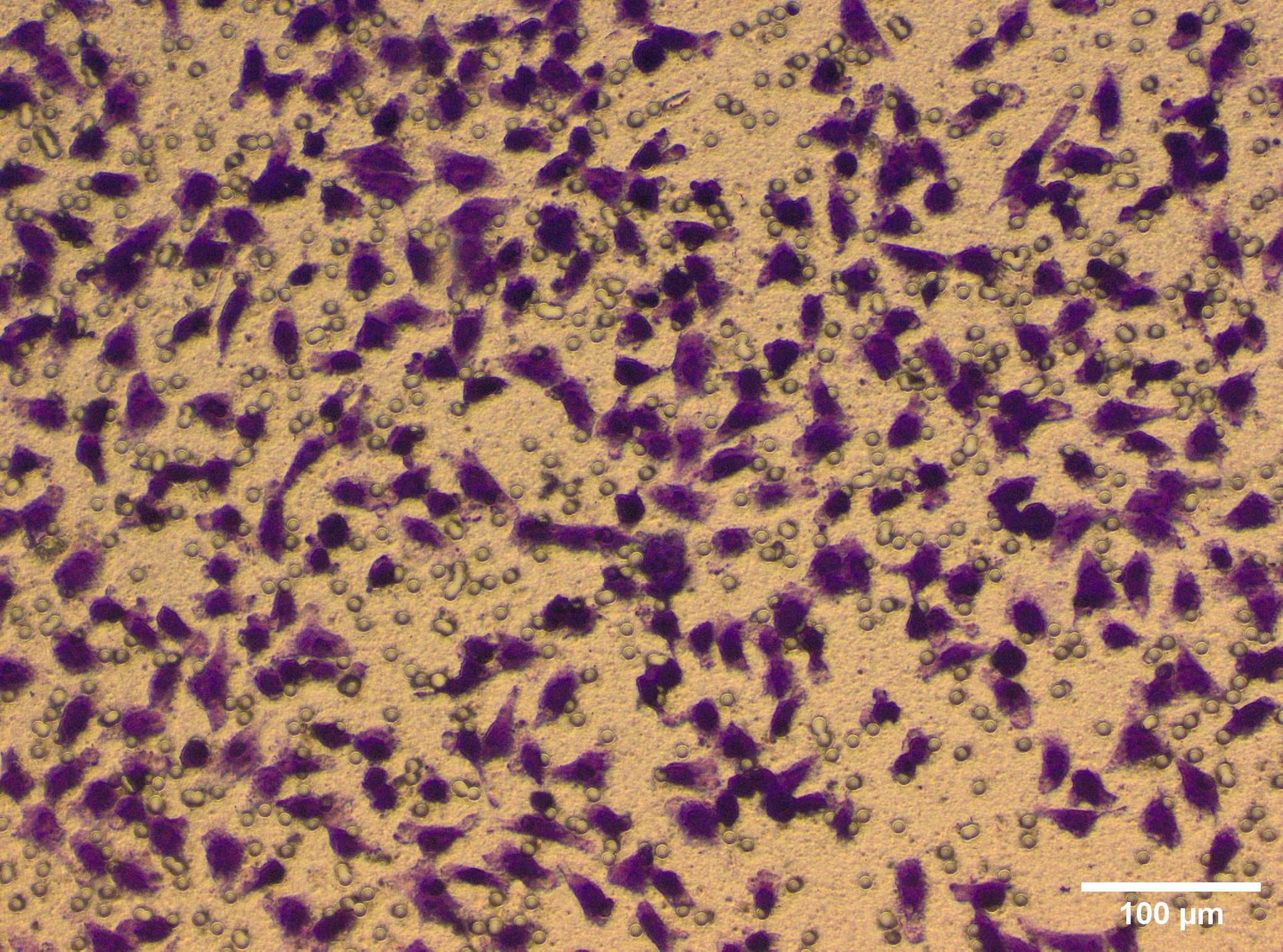

Supplement: Supplementary file 10 — EV Figure Source Data [file 44318_2026_766_MOESM10_ESM.zip › Figure EV4/Fig EV 4M/migration/MDA SH1 NO.jpg]

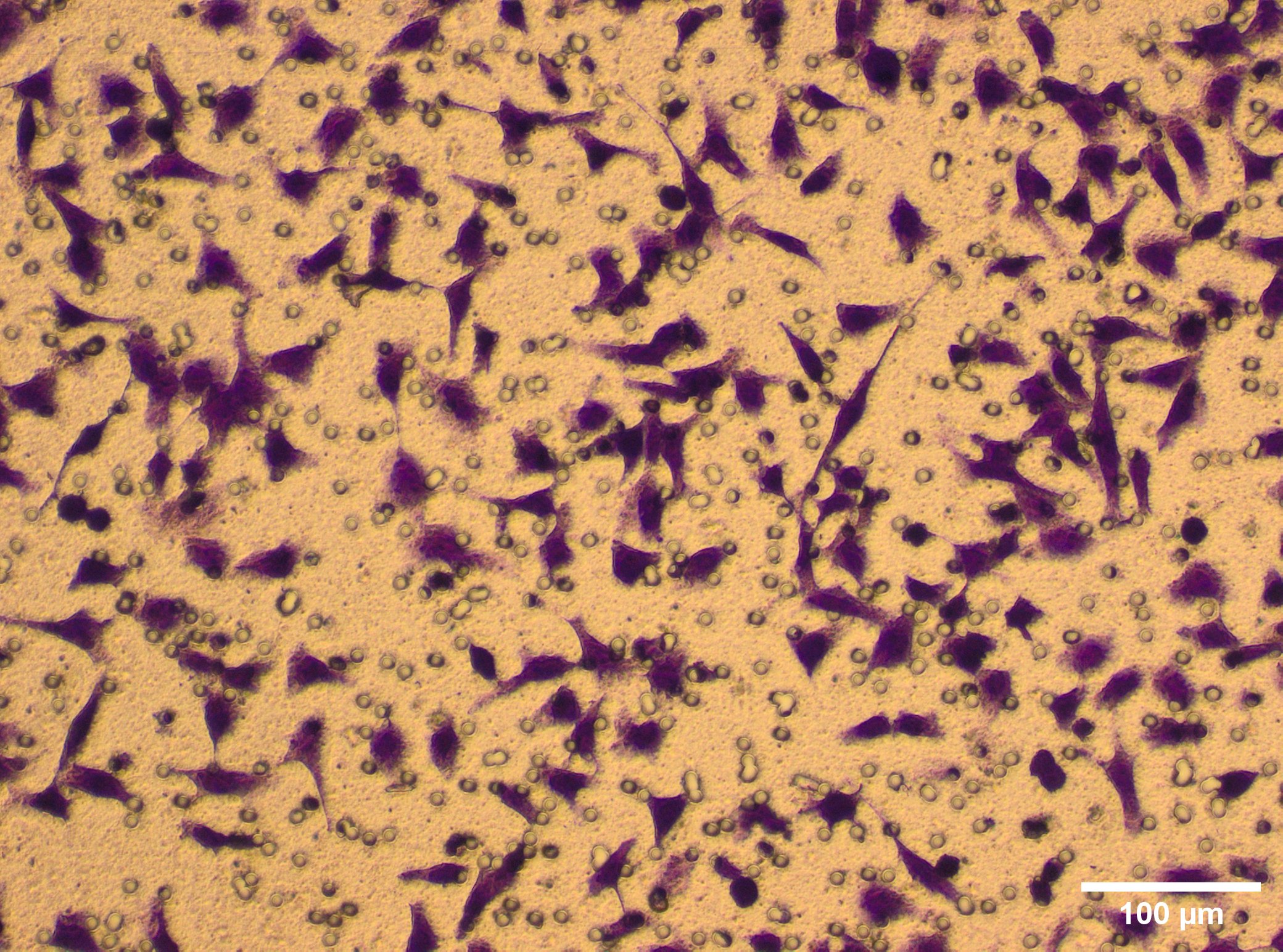

Supplement: Supplementary file 10 — EV Figure Source Data [file 44318_2026_766_MOESM10_ESM.zip › Figure EV5/Fig EV 5C/Invasion/vector.jpg]

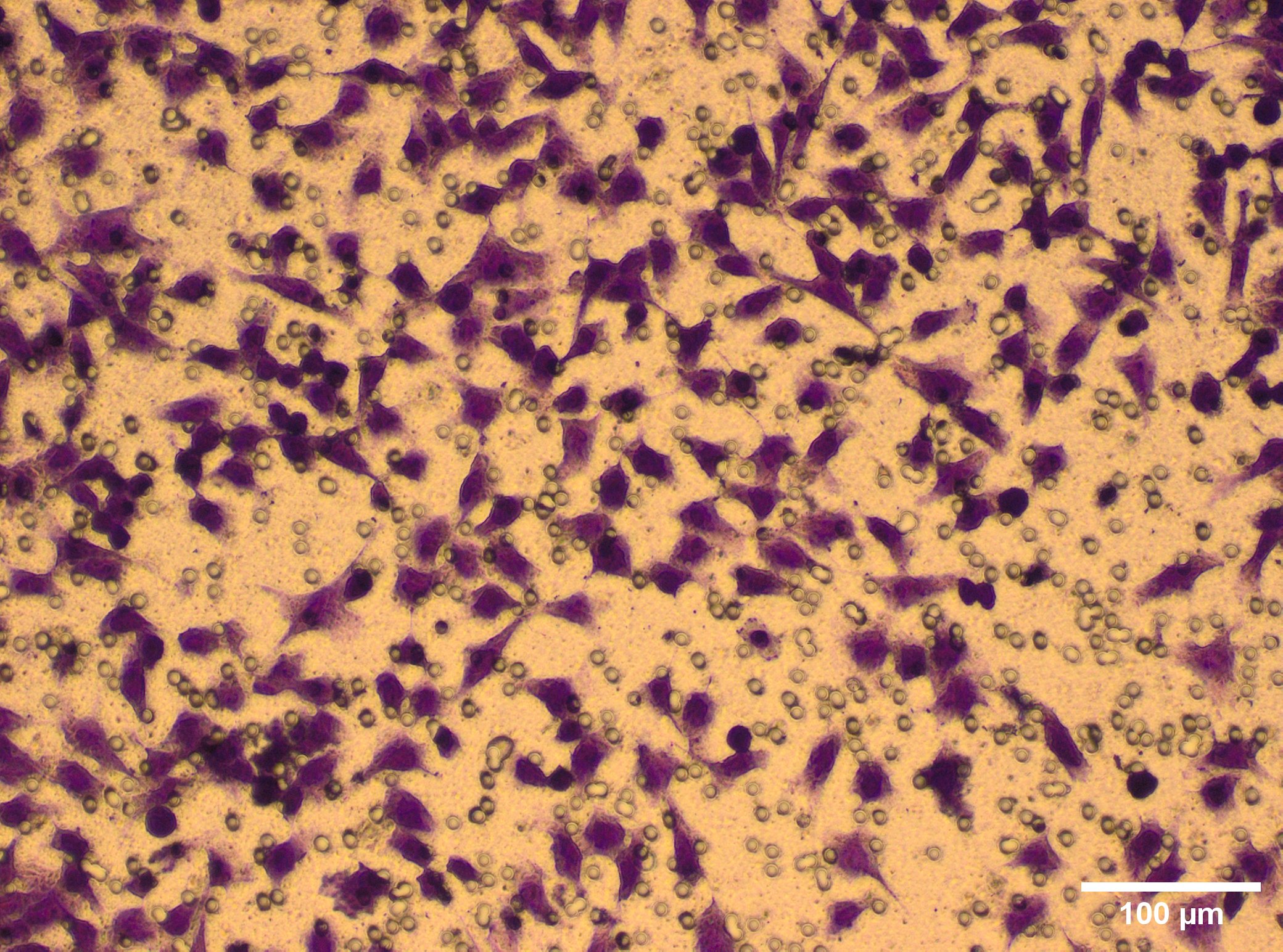

Supplement: Supplementary file 10 — EV Figure Source Data [file 44318_2026_766_MOESM10_ESM.zip › Figure EV5/Fig EV 5C/Invasion/efemp1.jpg]

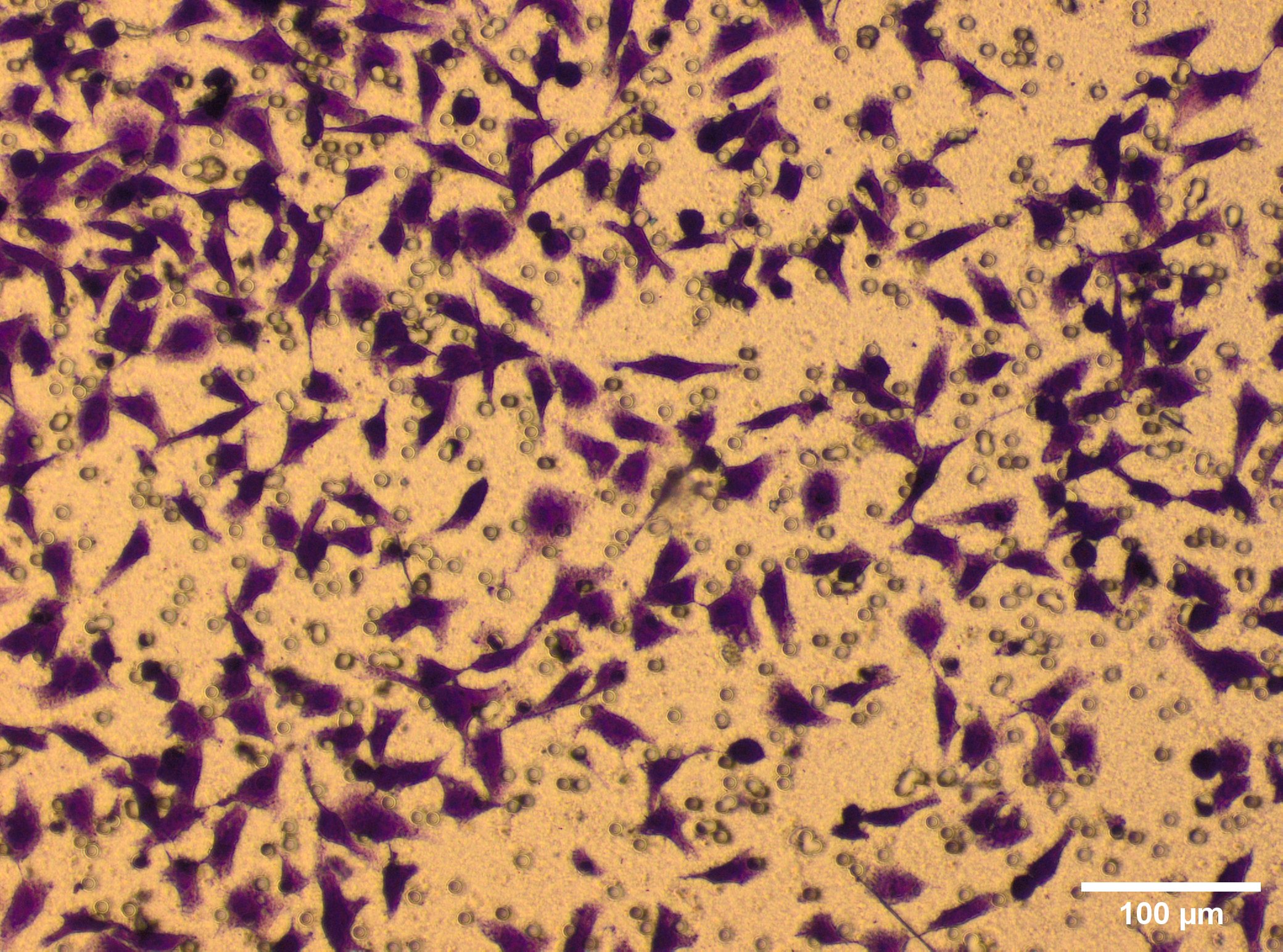

Supplement: Supplementary file 10 — EV Figure Source Data [file 44318_2026_766_MOESM10_ESM.zip › Figure EV5/Fig EV 5C/migration/vector.jpg]

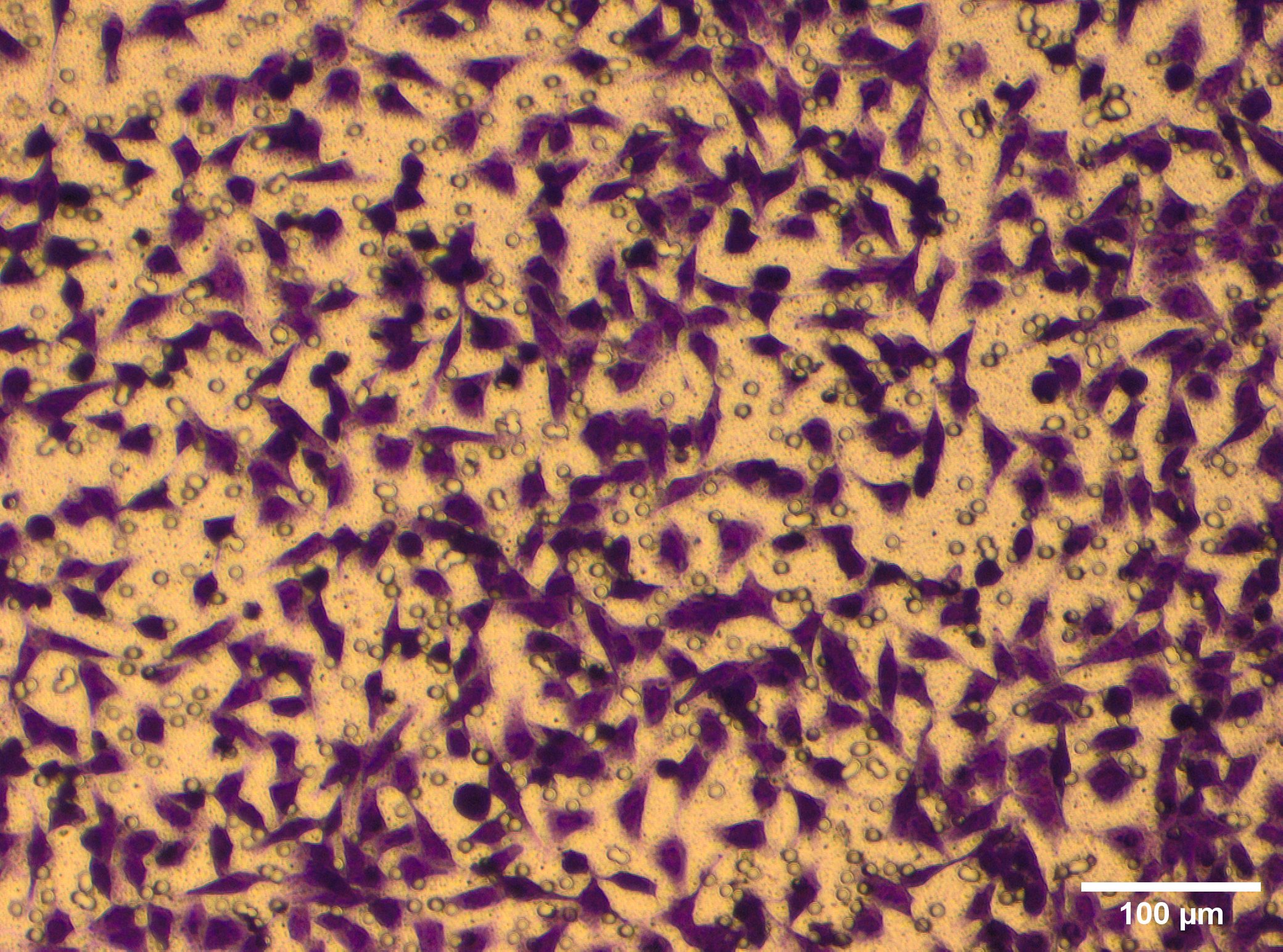

Supplement: Supplementary file 10 — EV Figure Source Data [file 44318_2026_766_MOESM10_ESM.zip › Figure EV5/Fig EV 5C/migration/efemp1.jpg]

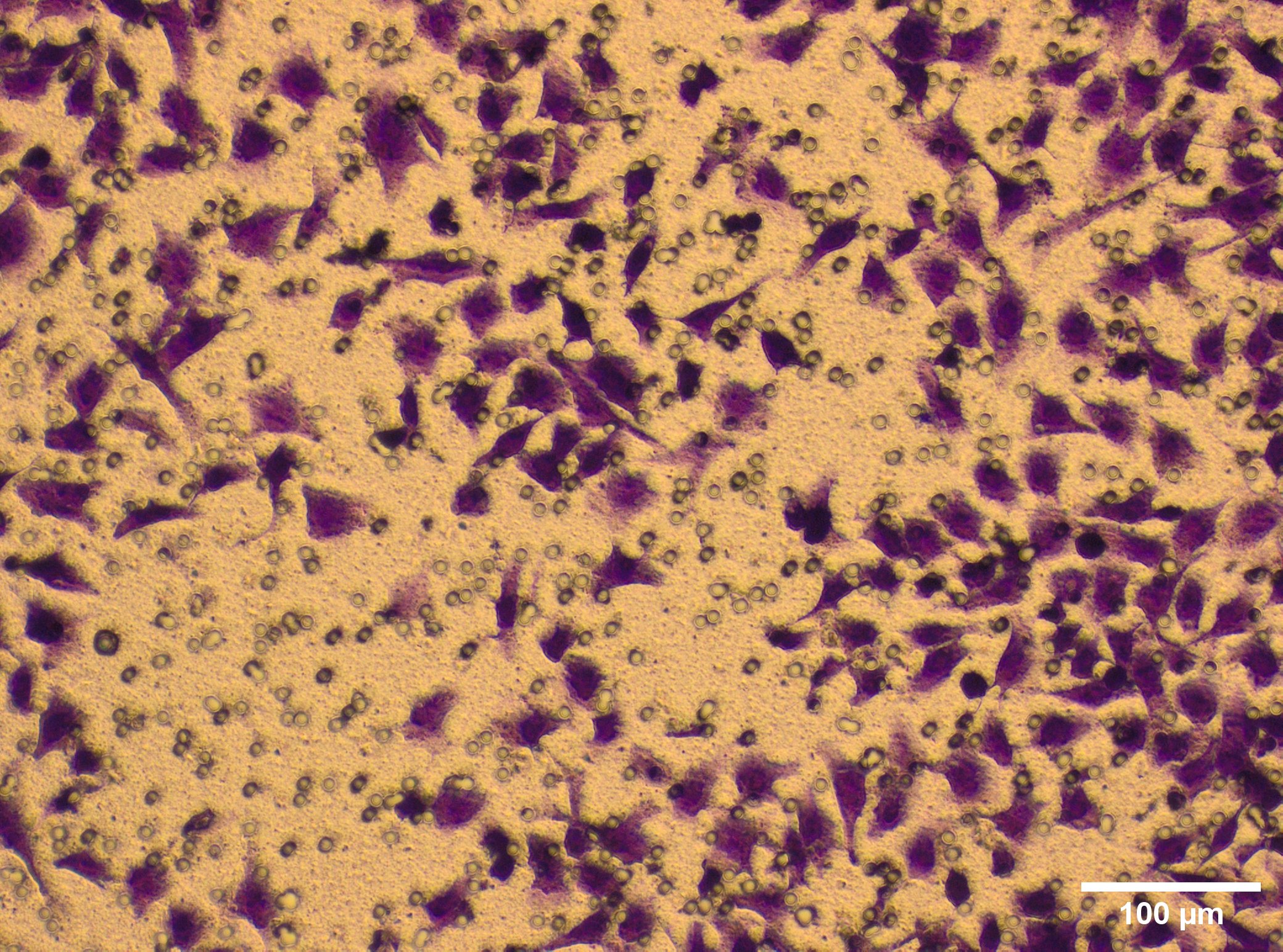

Supplement: Supplementary file 10 — EV Figure Source Data [file 44318_2026_766_MOESM10_ESM.zip › Figure EV5/Fig EV 5F/Invasion/sh2 no.jpg]

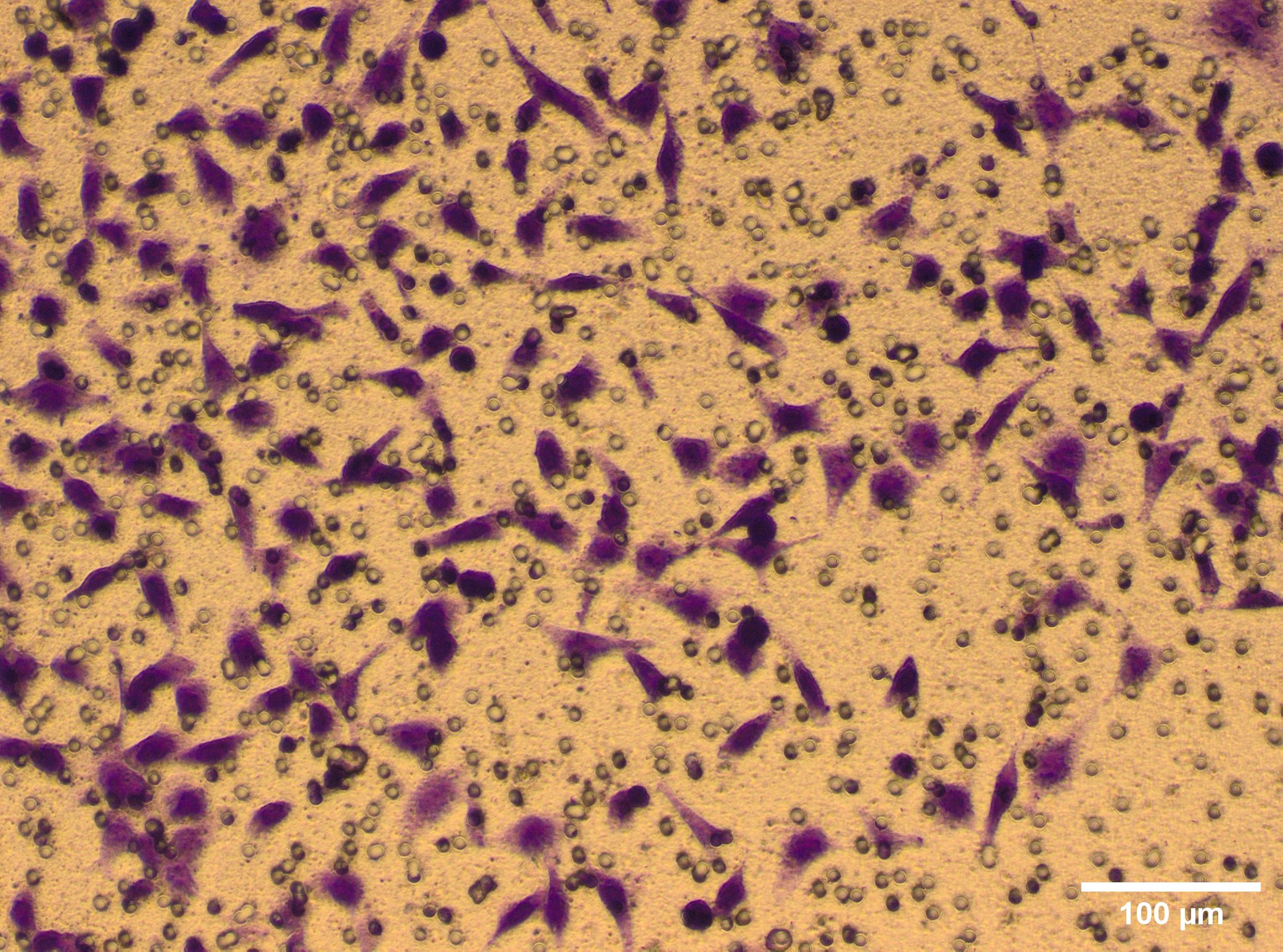

Supplement: Supplementary file 10 — EV Figure Source Data [file 44318_2026_766_MOESM10_ESM.zip › Figure EV5/Fig EV 5F/Invasion/sh2 dox.jpg]

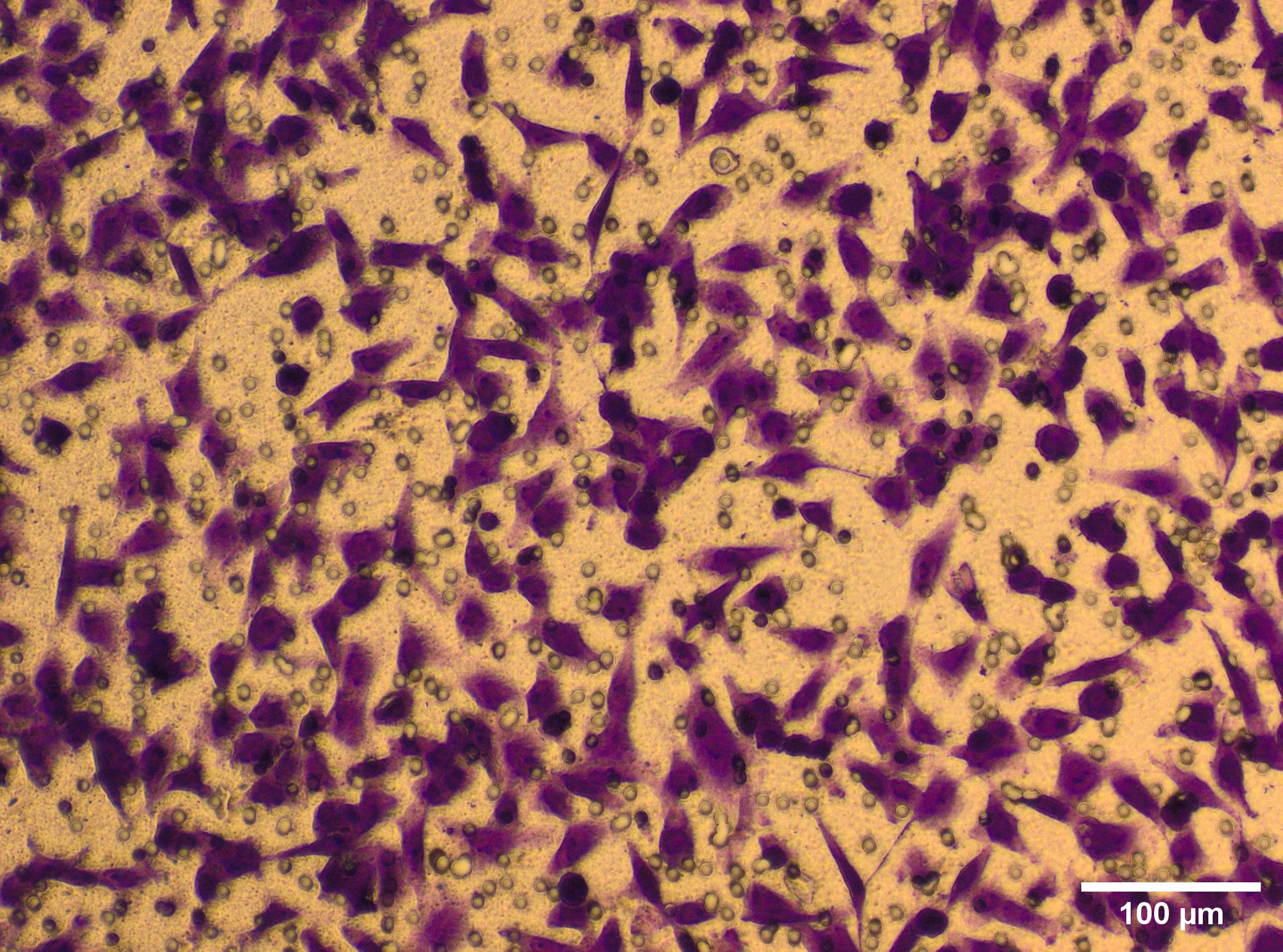

Supplement: Supplementary file 10 — EV Figure Source Data [file 44318_2026_766_MOESM10_ESM.zip › Figure EV5/Fig EV 5F/migration/sh2 no.jpg]

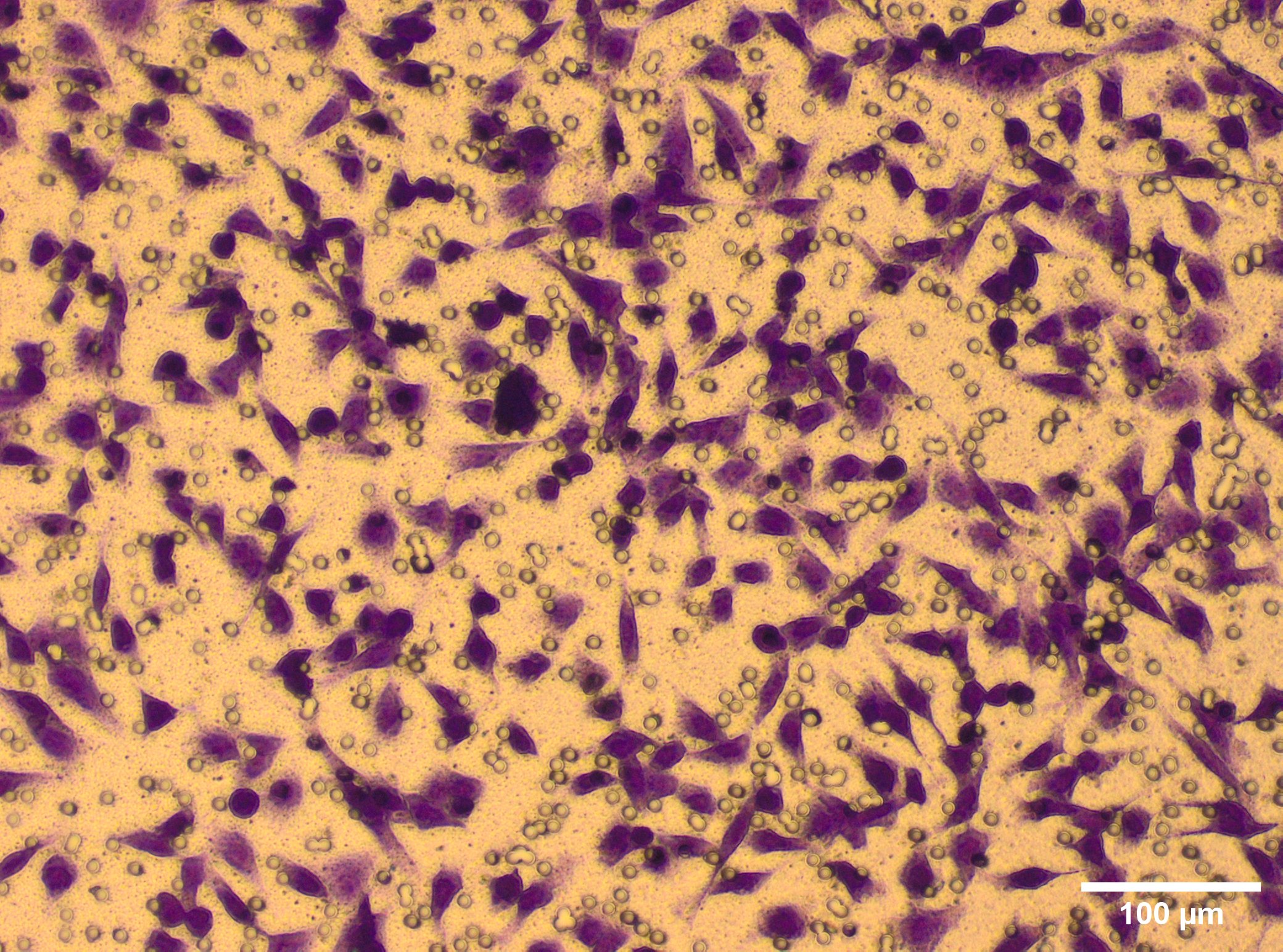

Supplement: Supplementary file 10 — EV Figure Source Data [file 44318_2026_766_MOESM10_ESM.zip › Figure EV5/Fig EV 5F/migration/sh2 dox.jpg]

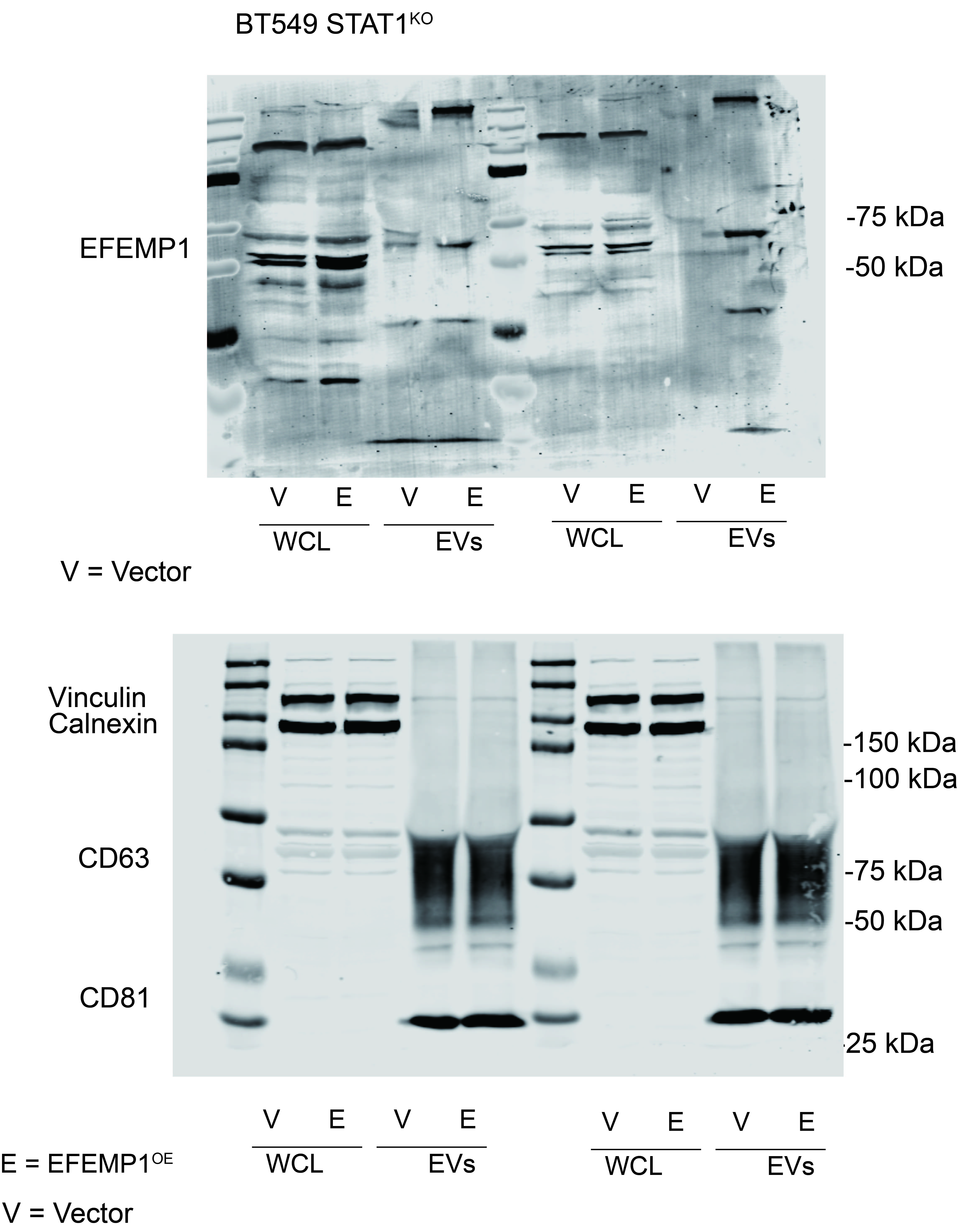

Supplement: Supplementary file 10 — EV Figure Source Data [file 44318_2026_766_MOESM10_ESM.zip › Figure EV7/Fig EV 7F.jpg]

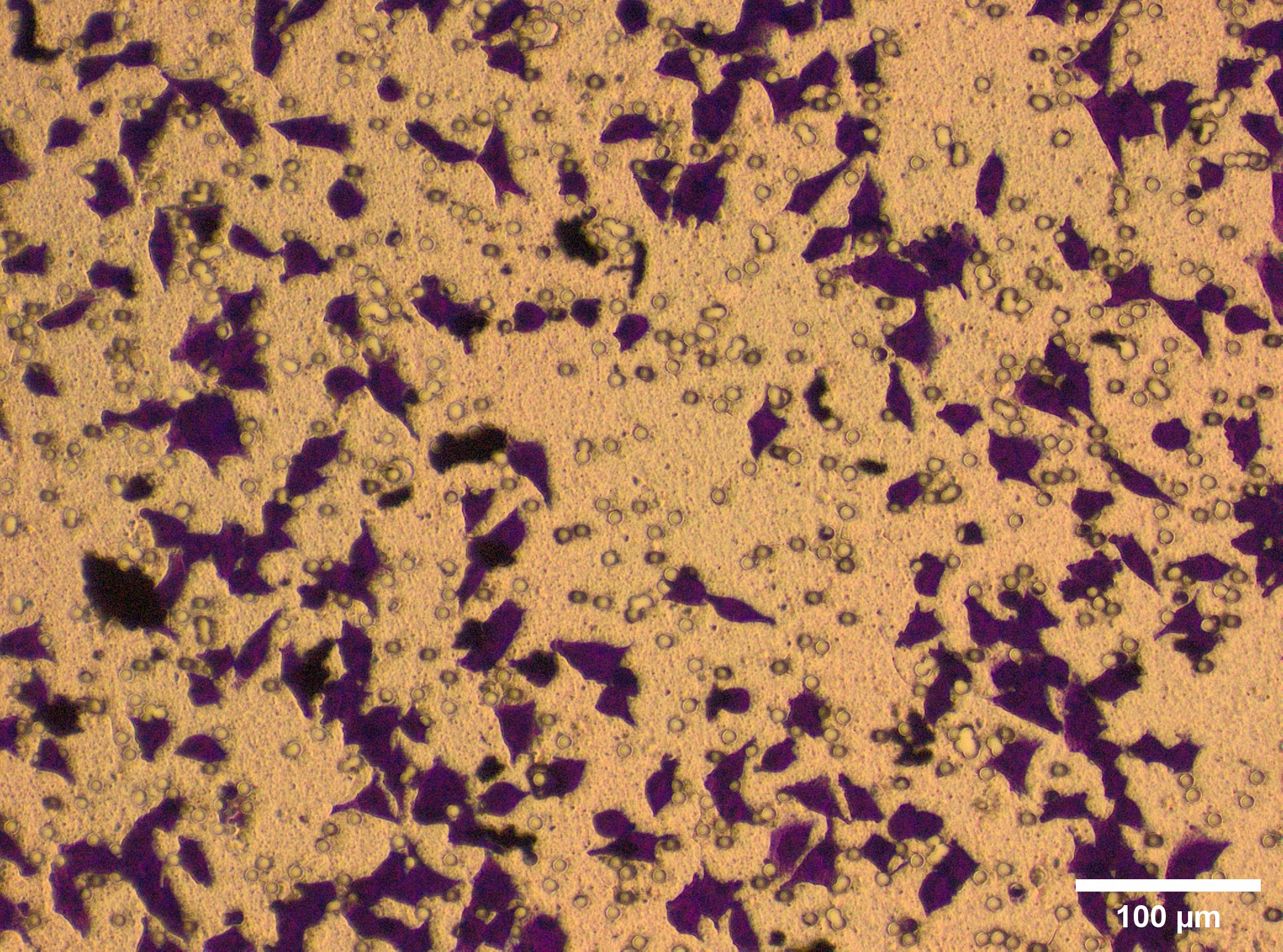

Supplement: Supplementary file 10 — EV Figure Source Data [file 44318_2026_766_MOESM10_ESM.zip › Figure EV7/Fig EV 7G/UC/uc vector migration.jpg]

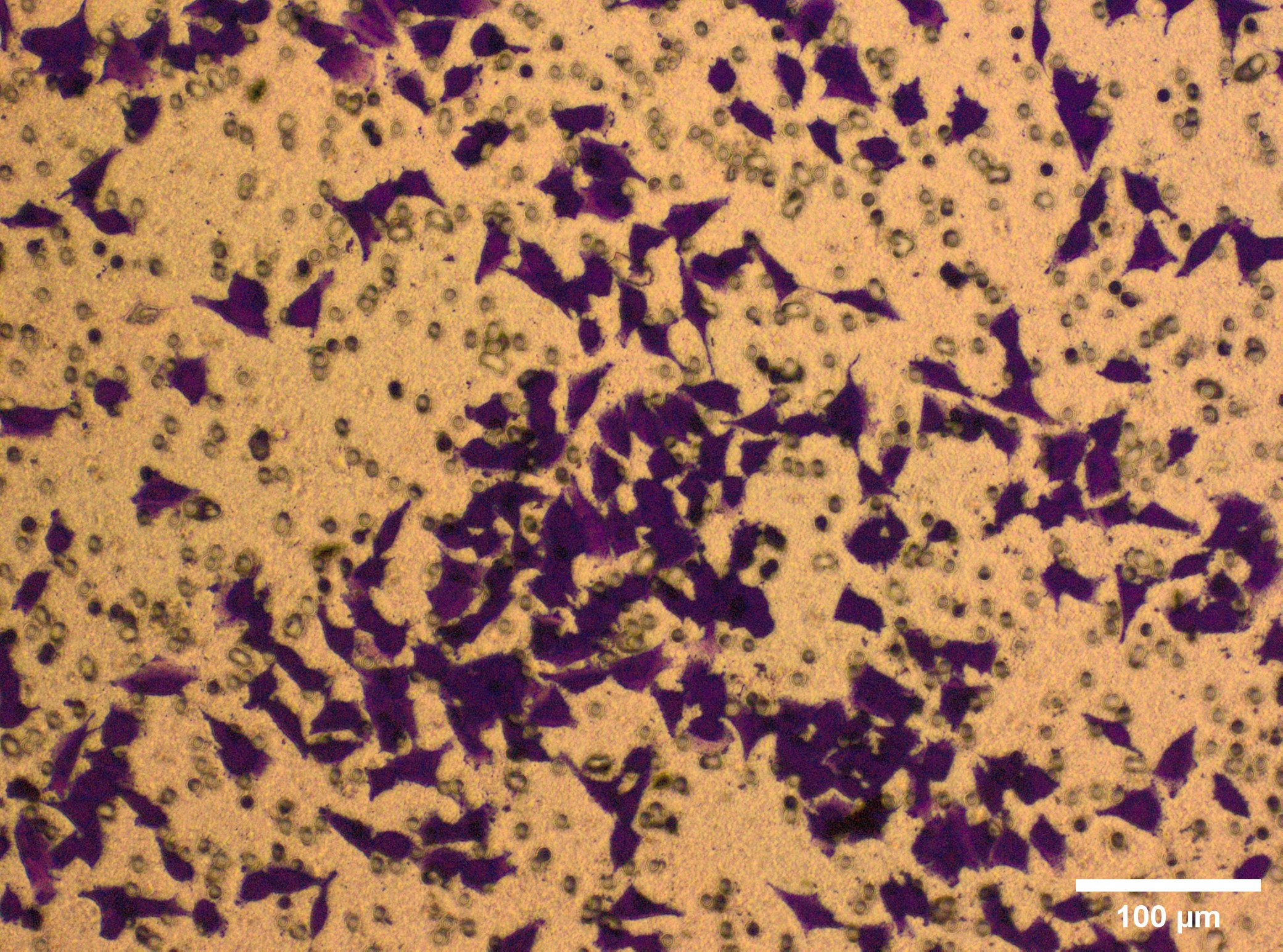

Supplement: Supplementary file 10 — EV Figure Source Data [file 44318_2026_766_MOESM10_ESM.zip › Figure EV7/Fig EV 7G/UC/uc efemp1 migration.jpg]

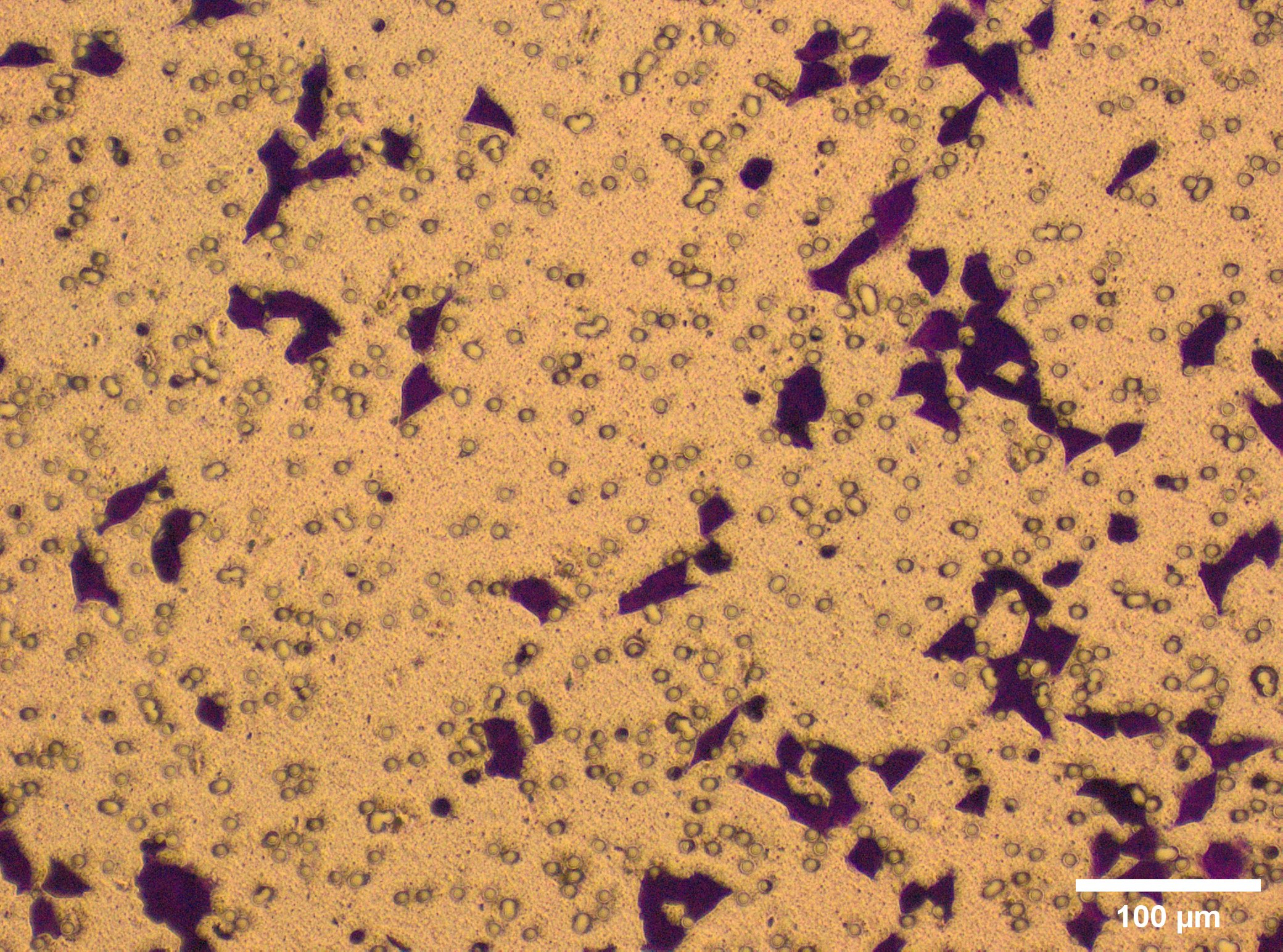

Supplement: Supplementary file 10 — EV Figure Source Data [file 44318_2026_766_MOESM10_ESM.zip › Figure EV7/Fig EV 7G/UC/uc vector invasion.jpg]

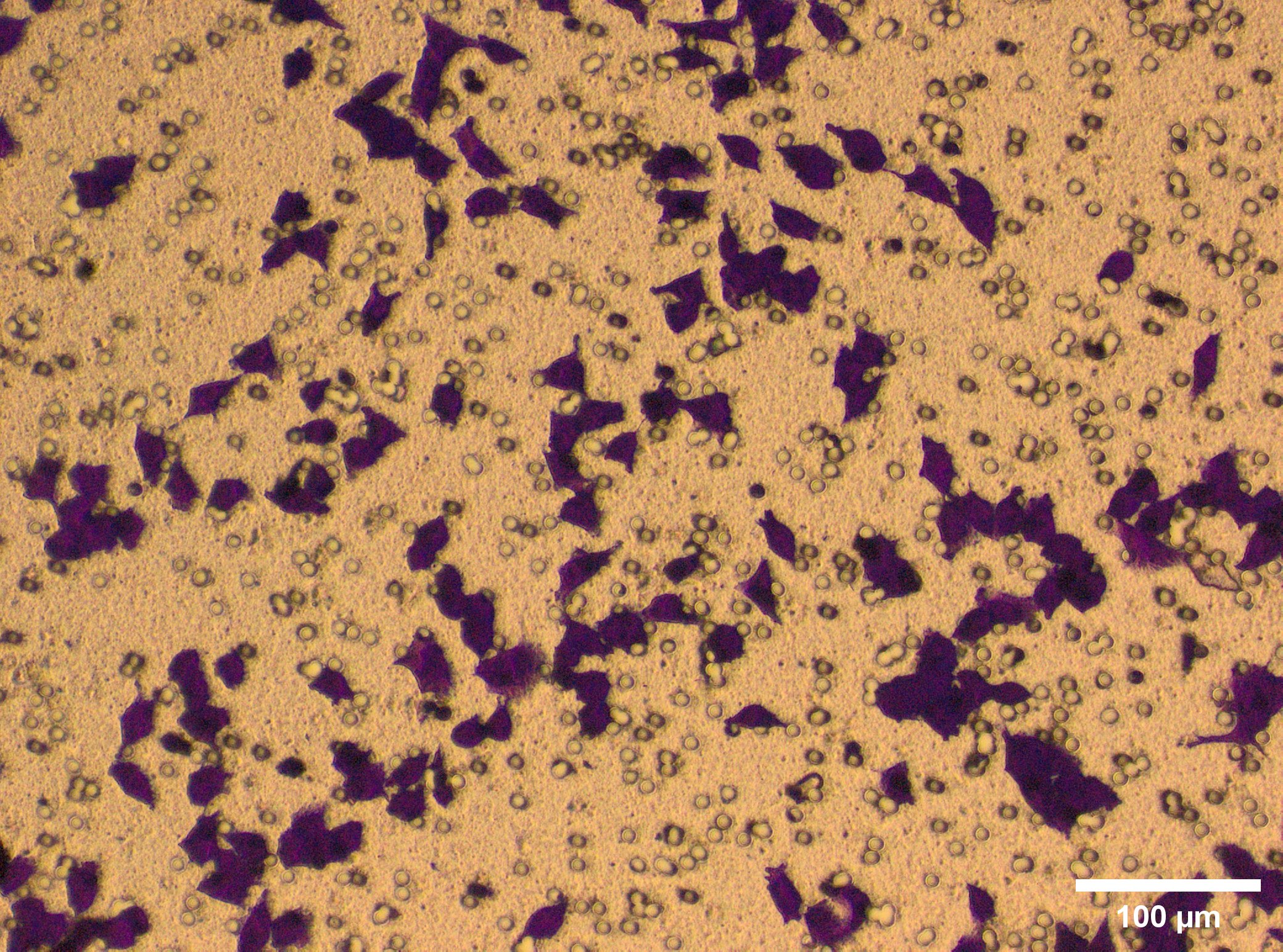

Supplement: Supplementary file 10 — EV Figure Source Data [file 44318_2026_766_MOESM10_ESM.zip › Figure EV7/Fig EV 7G/UC/uc efemp1 invasion.jpg]

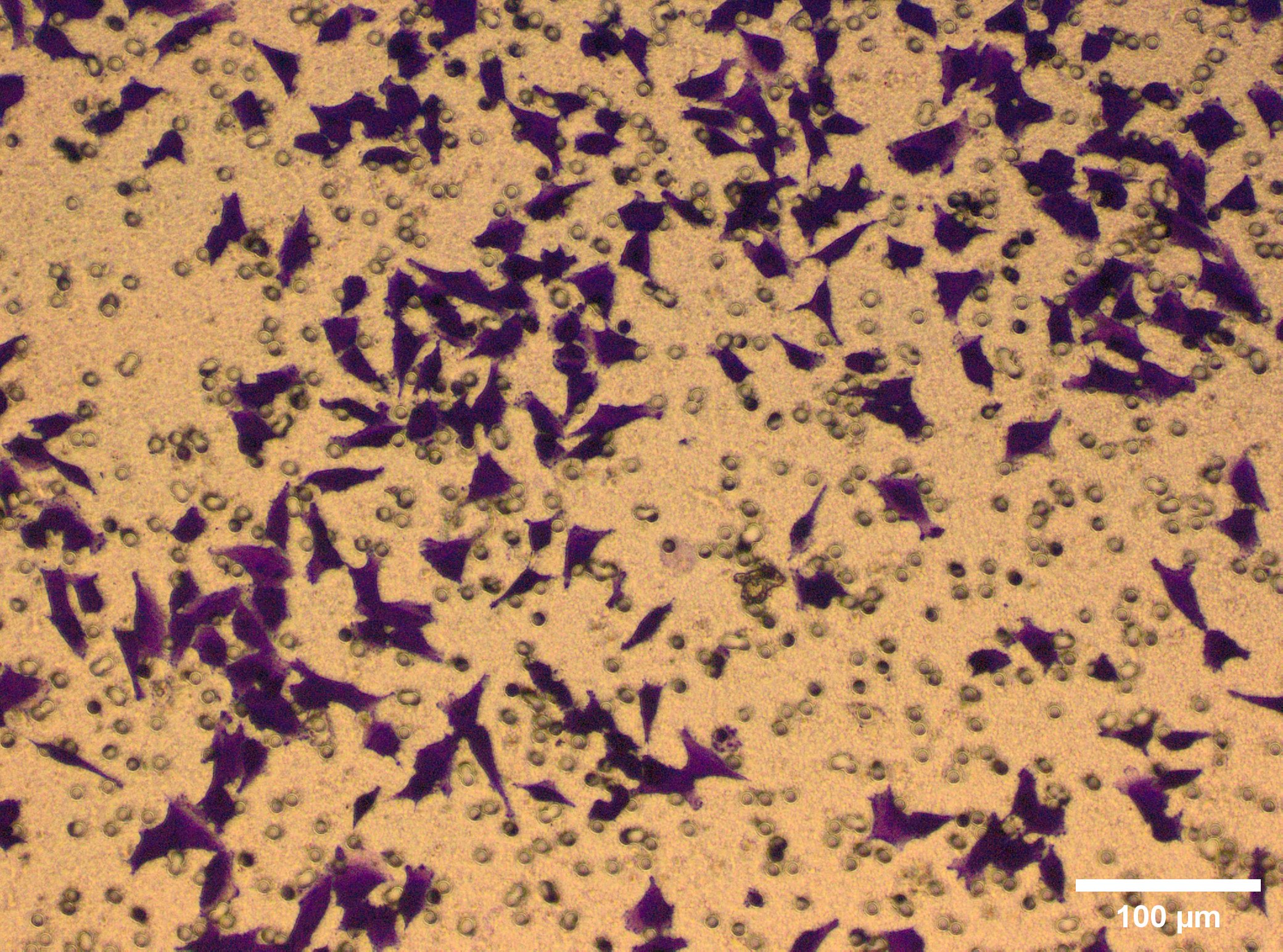

Supplement: Supplementary file 10 — EV Figure Source Data [file 44318_2026_766_MOESM10_ESM.zip › Figure EV7/Fig EV 7G/qEV/qev efemp1 migration.jpg]

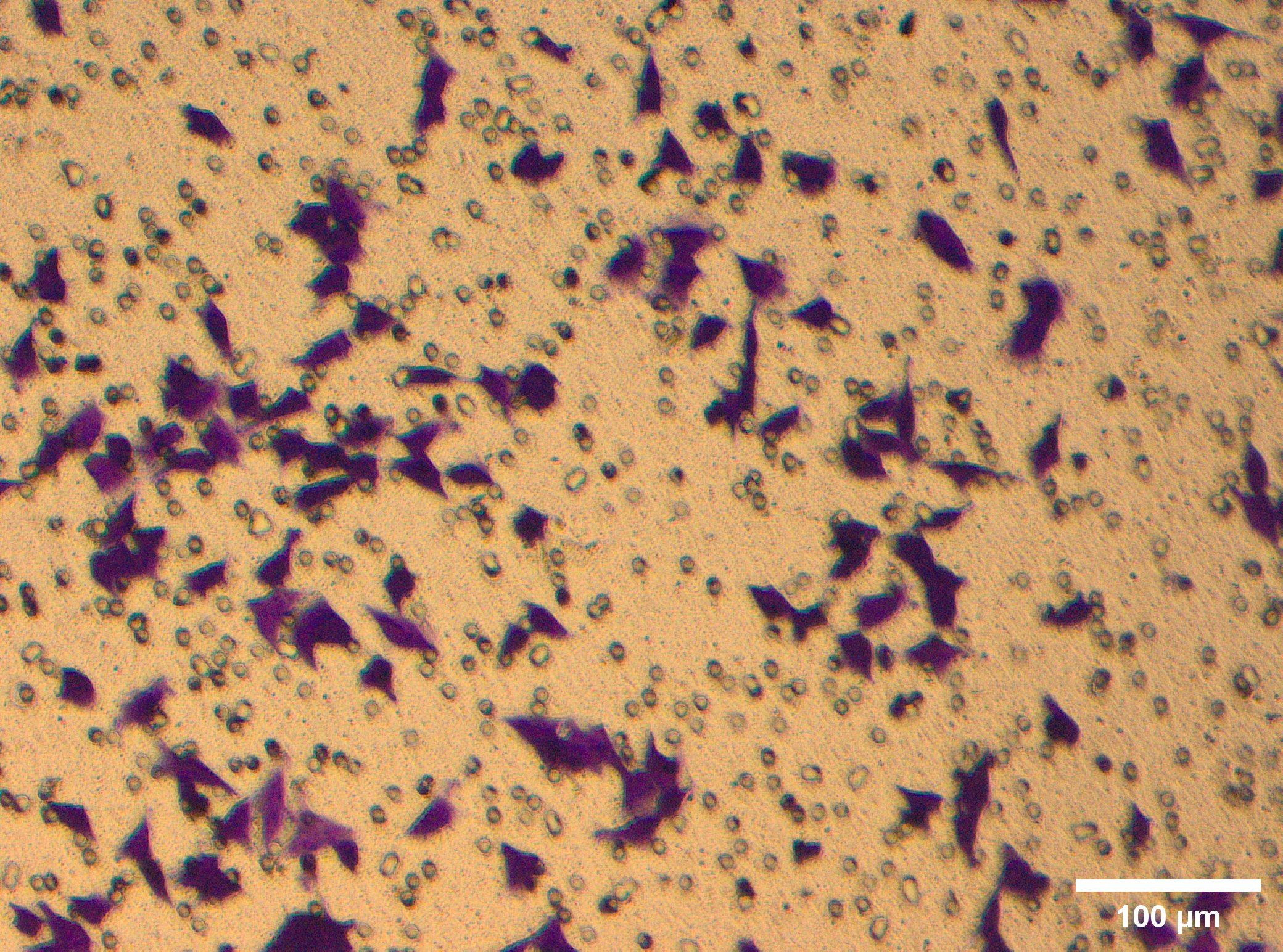

Supplement: Supplementary file 10 — EV Figure Source Data [file 44318_2026_766_MOESM10_ESM.zip › Figure EV7/Fig EV 7G/qEV/qev efemp1 invasion.jpg]

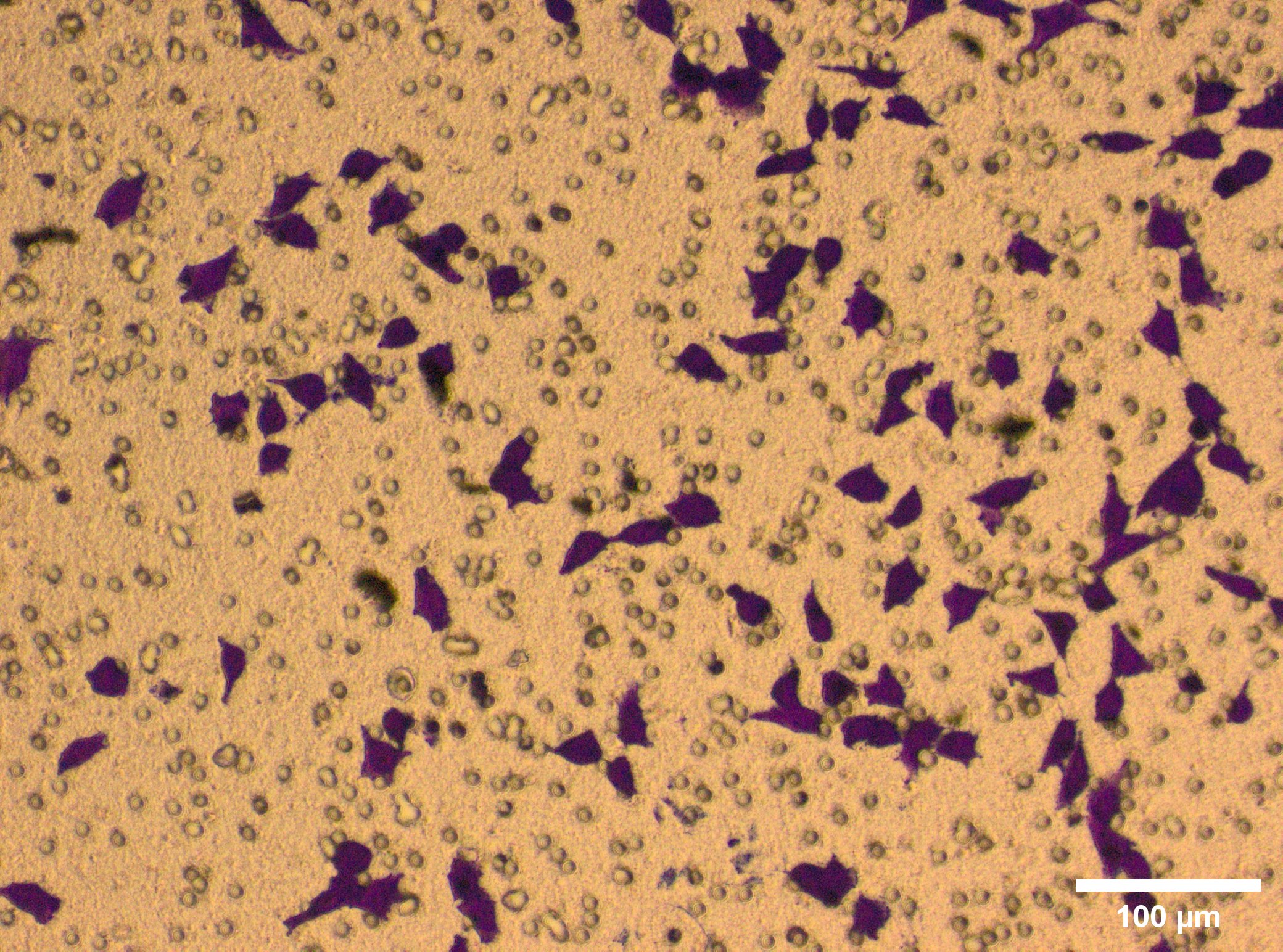

Supplement: Supplementary file 10 — EV Figure Source Data [file 44318_2026_766_MOESM10_ESM.zip › Figure EV7/Fig EV 7G/qEV/qev vector migration.jpg]

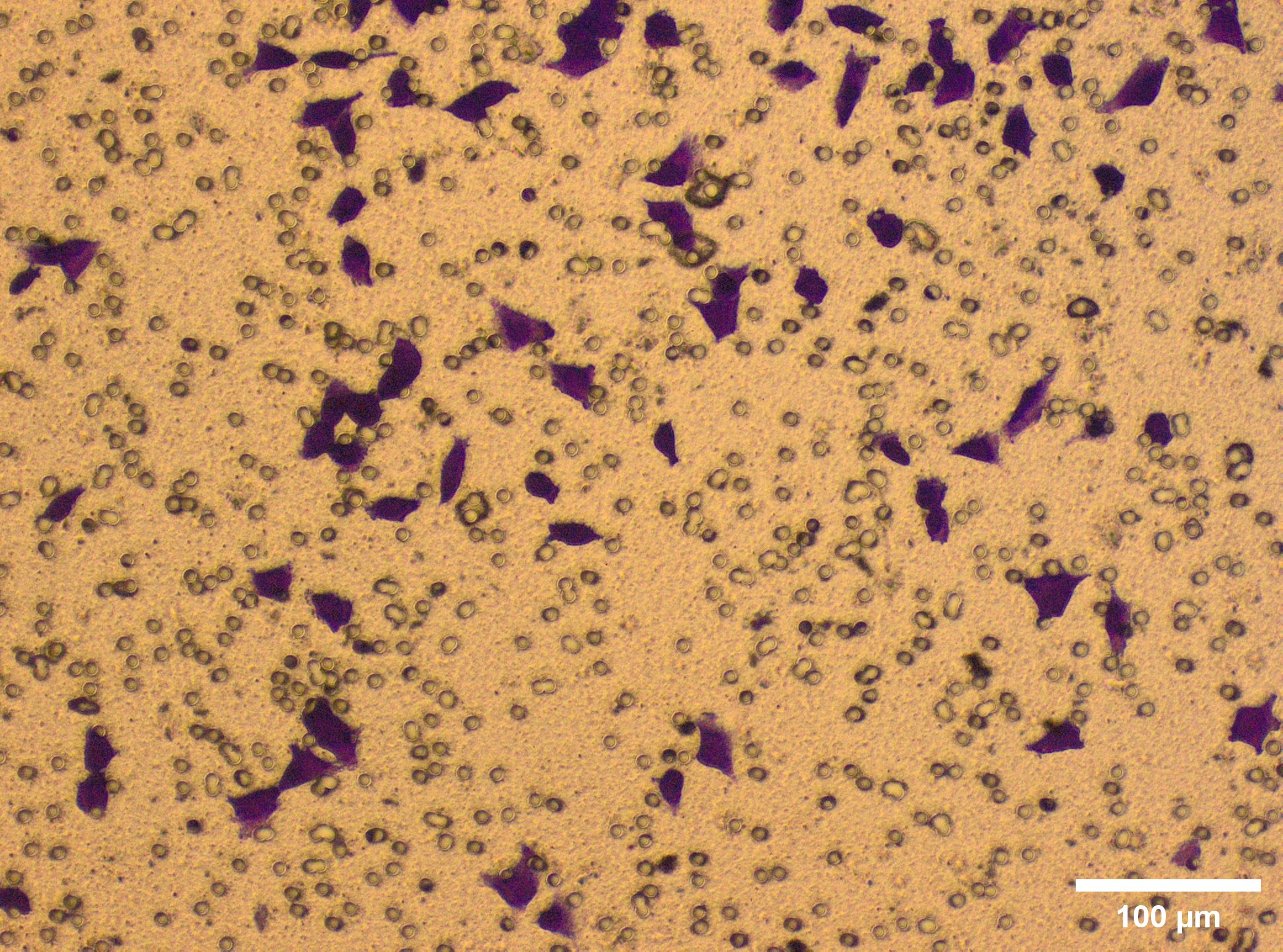

Supplement: Supplementary file 10 — EV Figure Source Data [file 44318_2026_766_MOESM10_ESM.zip › Figure EV7/Fig EV 7G/qEV/qev vector INvasionn.jpg]

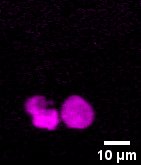

Supplement: Supplementary file 10 — EV Figure Source Data [file 44318_2026_766_MOESM10_ESM.zip › Figure EV8/Fig EV 8C/EFEMP1 OEX/Composite-13.jpg (red).jpg]

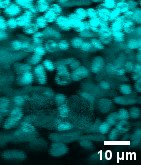

Supplement: Supplementary file 10 — EV Figure Source Data [file 44318_2026_766_MOESM10_ESM.zip › Figure EV8/Fig EV 8C/EFEMP1 OEX/Composite-13.jpg (green).jpg]

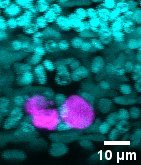

Supplement: Supplementary file 10 — EV Figure Source Data [file 44318_2026_766_MOESM10_ESM.zip › Figure EV8/Fig EV 8C/EFEMP1 OEX/Composite-13.jpg]

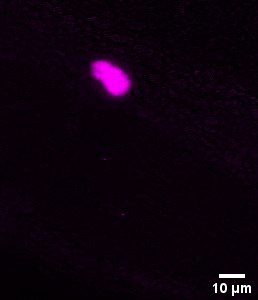

Supplement: Supplementary file 10 — EV Figure Source Data [file 44318_2026_766_MOESM10_ESM.zip › Figure EV8/Fig EV 8C/Control/Composite-1.jpg (red).jpg]

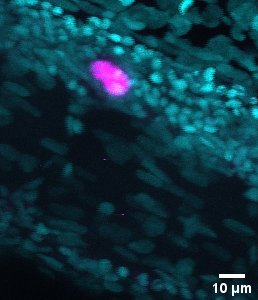

Supplement: Supplementary file 10 — EV Figure Source Data [file 44318_2026_766_MOESM10_ESM.zip › Figure EV8/Fig EV 8C/Control/Composite-1.jpg]

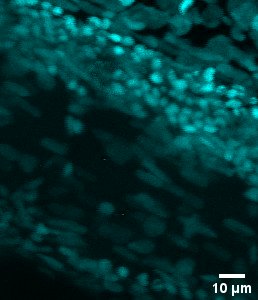

Supplement: Supplementary file 10 — EV Figure Source Data [file 44318_2026_766_MOESM10_ESM.zip › Figure EV8/Fig EV 8C/Control/Composite-1.jpg (green).jpg]

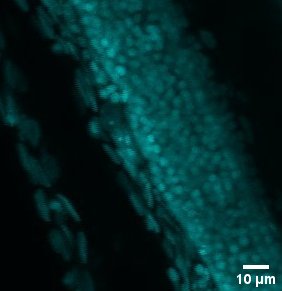

Supplement: Supplementary file 10 — EV Figure Source Data [file 44318_2026_766_MOESM10_ESM.zip › Figure EV8/Fig EV 8B/EFEMP1 KD/MAX_C1-20221122sq_complete.lif.jpg]

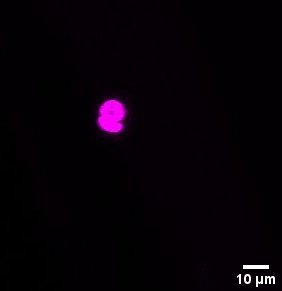

Supplement: Supplementary file 10 — EV Figure Source Data [file 44318_2026_766_MOESM10_ESM.zip › Figure EV8/Fig EV 8B/EFEMP1 KD/MAX_C2-20221122sq_complete.lif.jpg]

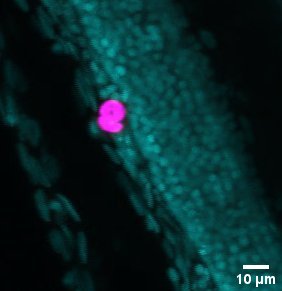

Supplement: Supplementary file 10 — EV Figure Source Data [file 44318_2026_766_MOESM10_ESM.zip › Figure EV8/Fig EV 8B/EFEMP1 KD/Composite-1.jpg]

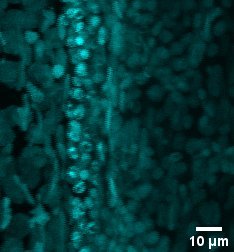

Supplement: Supplementary file 10 — EV Figure Source Data [file 44318_2026_766_MOESM10_ESM.zip › Figure EV8/Fig EV 8B/Control/MAX_C1-20221122sq_complete.lif - group2-1-1-1.jpg]

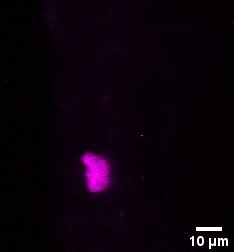

Supplement: Supplementary file 10 — EV Figure Source Data [file 44318_2026_766_MOESM10_ESM.zip › Figure EV8/Fig EV 8B/Control/MAX_C2-20221122sq_complete.lif - group2-1-1-1.jpg]

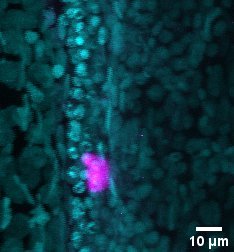

Supplement: Supplementary file 10 — EV Figure Source Data [file 44318_2026_766_MOESM10_ESM.zip › Figure EV8/Fig EV 8B/Control/Composite-2.jpg]
