# Supplementary material for: Chromosomal instability promotes cell migration and invasion via EFEMP1 secretion into extracellular vesicles
Source: EMBO J. 2026 Apr 13;45(10):3471–99. doi: 10.1038/s44318-026-00766-4 (PMC13187162; doi:10.1038/s44318-026-00766-4)
Supplement: Supplementary file 12 — Expanded View Figures [file 44318_2026_766_MOESM12_ESM.pdf]

# Expanded View Figures

**Figure EV1. Biophysical characterization of EVs.**

(**A**) Transmission electron microscopy (TEM) images showing EV morphology when isolated from BT549 treated cells. Scale bar: 200 nm. (**B**) Ponceau S staining of ultracentrifuge inputs and SEC fractions. (**C**, **D**) Nanoparticle tracking analysis of EVs isolated from BT549 cells (**C**) and MDA-MB-231 cells (**D**), to determine EV size distribution and EV concentrations. (**E**) Immunofluorescence images of BT549 cells labelled for PKH26 and F-actin incubated for 4 h with indicated EV preparations, compared to DMSO controls. Nuclei were counterstained with DAPI. (**F**) Quantification of PKH26 fluorescence intensity (RFU) in BT549 cells in the presence or absence of 500 nM REV-induced EVs. $^*p < 0.05$. RFU, relative fluorescence units. Error bars represent the standard deviation (SD) of the mean. $N = 3$ independent experiments; each data point represents the mean of the replicates; Statistical significance was determined with a paired two-tailed t-test. $p = 0.0165$; $^*p < 0.05$. Source data are available online for this figure.

© The Author(s)

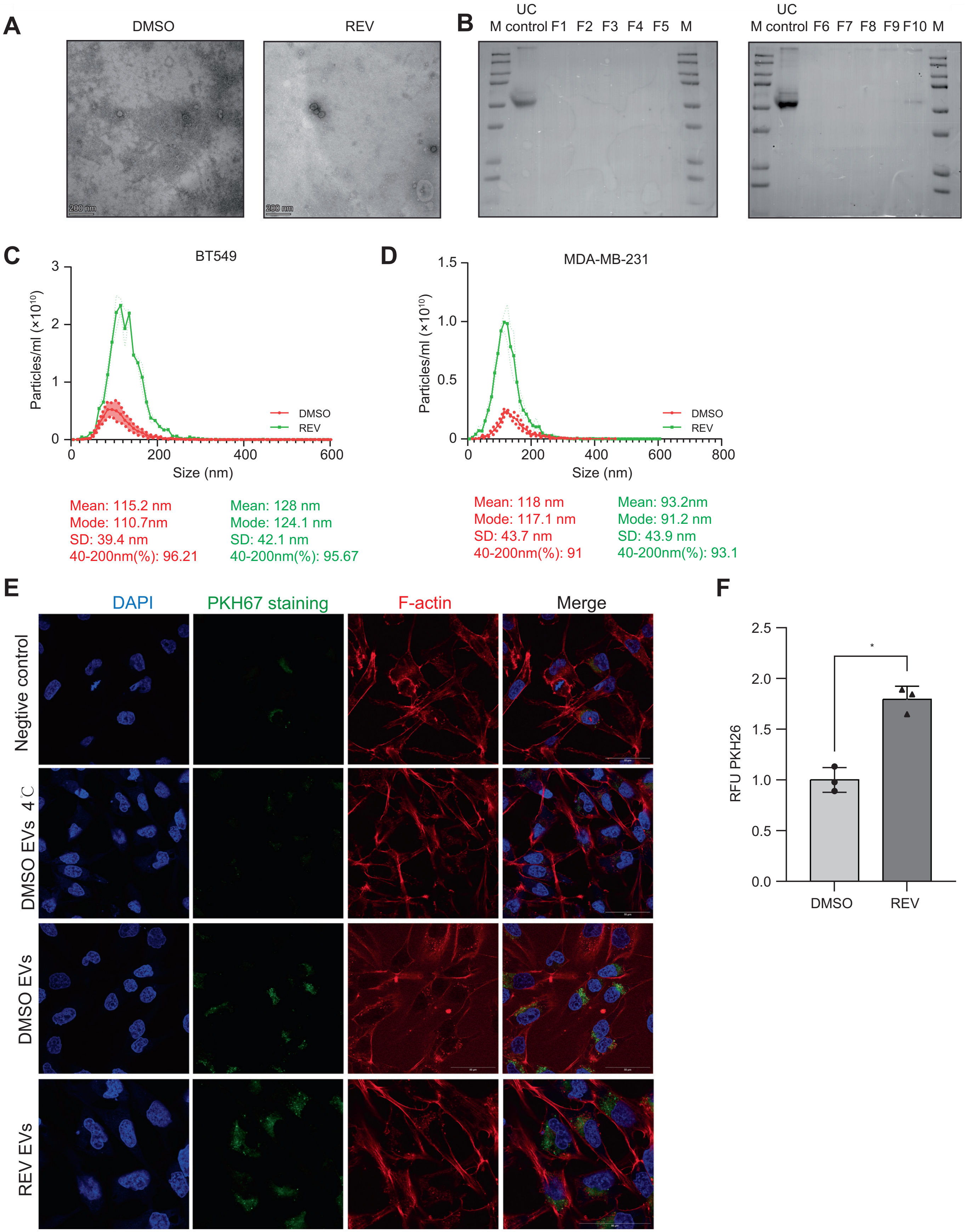
A
DMSO
REV
200 nm
200 nm
B
UC
M control F1 F2 F3 F4 F5 M
UC
M control F6 F7 F8 F9 F10 M
C
BT549
Particles/ml (×10^10)
Size (nm)
DMSO
REV
Mean: 115.2 nm
Mode: 110.7nm
SD: 39.4 nm
40-200nm(%): 96.21
Mean: 128 nm
Mode: 124.1 nm
SD: 42.1 nm
40-200nm(%): 95.67
D
MDA-MB-231
Particles/ml (×10^10)
Size (nm)
DMSO
REV
Mean: 118 nm
Mode: 117.1 nm
SD: 43.7 nm
40-200nm(%): 91
Mean: 93.2nm
Mode: 91.2 nm
SD: 43.9 nm
40-200nm(%): 93.1
E
DAPI
PKH67 staining
F-actin
Merge
Negtive control
DMSO EVs 4℃
DMSO EVs
REV EVs
F
RFU PKH26
DMSO
REV
*

 © The Author(s)

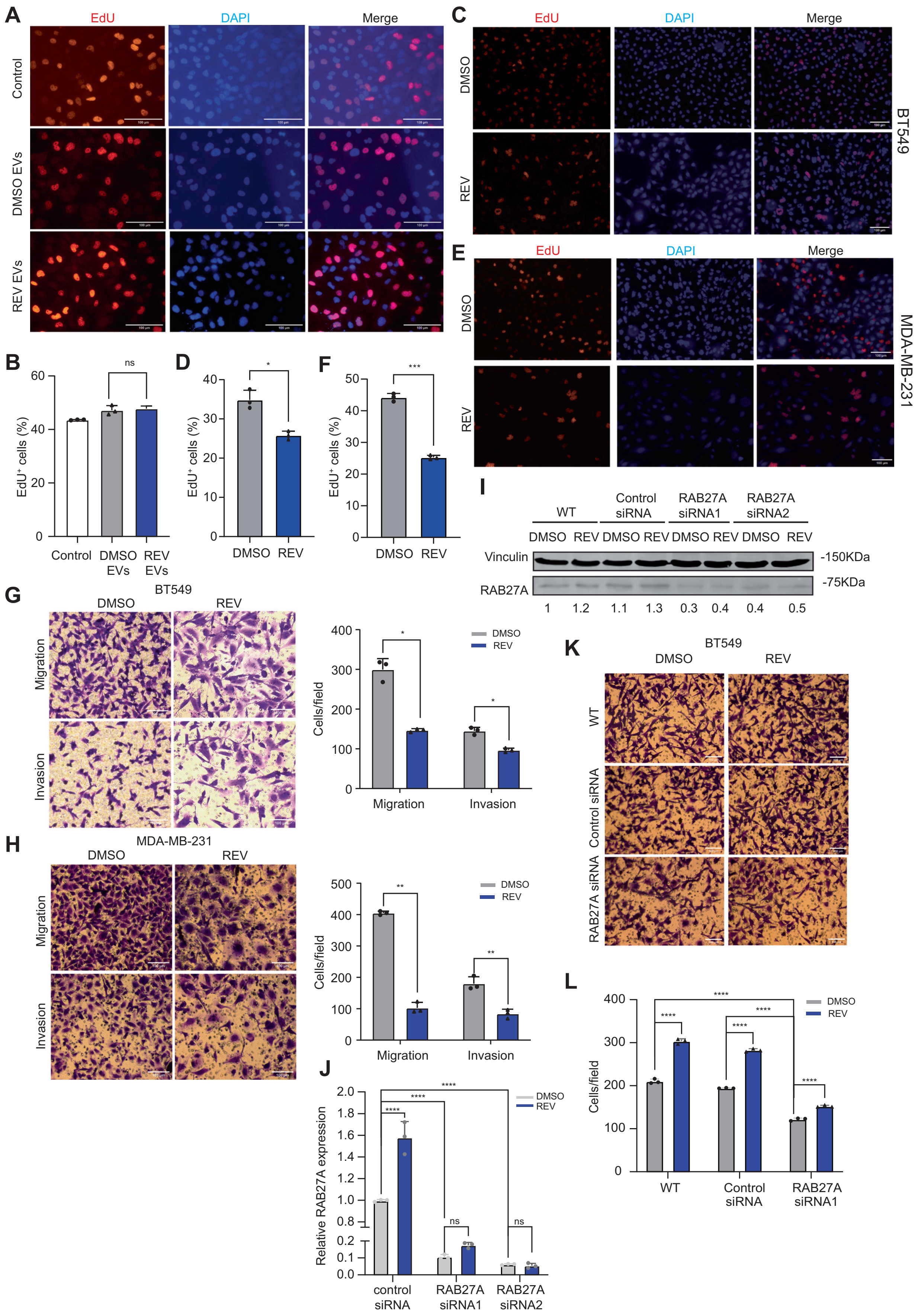
A
EdU
DAPI
Merge
Control
DMSO EVs
REV EVs
B
EdU+ cells (%)
Control
DMSO EVs
REV EVs
ns
C
DMSO
REV
BT549
D
E
MDA-MB-231
F
G
BT549
Migration
Invasion
Cells/field
H
MDA-MB-231
I
WT
Control siRNA
RAB27A siRNA1
RAB27A siRNA2
Vinculin
RAB27A
-150KDa
-75KDa
1 1.2 1.1 1.3 0.3 0.4 0.4 0.5
J
Relative RAB27A expression
control siRNA
RAB27A siRNA1
RAB27A siRNA2
K
L

**Figure EV2. CIN drives pro-migratory EVs and requires RAB27A in recipient cells.**

(**A**, **B**) Representative images of EdU incorporation in MDA-MB-231 cells treated with EVs from PBS, DMSO or REV treated cells (**A**) and quantification for three biological replicates (**B**). Error bars represent the standard deviation (SD) of the mean. $N = 3$ independent experiments; each data point represents the mean of the replicates; Statistical significance was determined with a one-way ANOVA. (**C**, **D**) Representative immunofluorescence images of EdU incorporation in BT549 EV-donor cells treated with DMSO or REV prior to EV isolation (**C**), quantified for three biological replicates (**D**) Error bars represent the standard deviation (SD) of the mean. $N = 3$ independent experiments; each data point represents the mean of the replicates; Statistical significance was determined with a paired two-tailed t-test. $p = 0.0405$, $^{*}p < 0.05$. (**E**, **F**) Representative immunofluorescence images of EdU incorporation in MDA-MB-231 EV-donor cells treated with DMSO or REV prior to EV isolation (**E**), quantified for three biological replicates (**F**). Error bars represent the standard deviation (SD) of the mean. $N = 3$ independent experiments; each data point represents the mean of within-experiment replicates; Statistical significance was determined with a paired two-tailed t-test. $p = 0.0004$, $^{***}p < 0.001$. (**G**, **H**) Migration and invasion of BT549 $p = 0.0132$, $^{*}p < 0.05$. (**G**) and MDA-MB-231 $p = 0.0180$, $^{*}p < 0.05$. (**H**) cells treated with DMSO ($CIN^{LOW}$) or REV ($CIN^{HIGH}$) using trans-well assays. Statistical significance was determined using two-sided T-tests ($^{*}p < 0.05$, $^{**}p < 0.01$, $^{***}p < 0.001$, $^{****}p < 0.0001$). Experiments were performed as biological triplicates. Scale bar: 100 µm. Error bars represent the standard deviation (SD) of the mean. $N = 3$ independent experiments; each data point represents the mean of the replicates; Statistical significance was determined with a paired two-tailed t-test. (**I**) Western blot quantification of RAB27A knockdown efficacy in BT549 cells. (**J**) qPCR quantification of RAB27A knockdown efficacy in BT549 cells: Statistical tests were done using two-way ANOVA ($n = 3$; ***, $^{**}p < 0.01$, ***, $p < 0.001$, $^{****}p < 0.0001$). (**K**, **L**) Representative images (**K**) and quantification (**L**) of siRNA-mediated RAB27A knockdown in BT549 recipient cells reduces the increases in migration induced by reversine-derived ($CIN^{HIGH}$) EVs. Scale bar: 100 µm. Error bars represent the standard deviation (SD) of the mean. $N = 3$ independent experiments; each data point represents the mean of the replicates; statistical significance was determined with a two-way ANOVA. Source data are available online for this figure.

 © The Author(s)

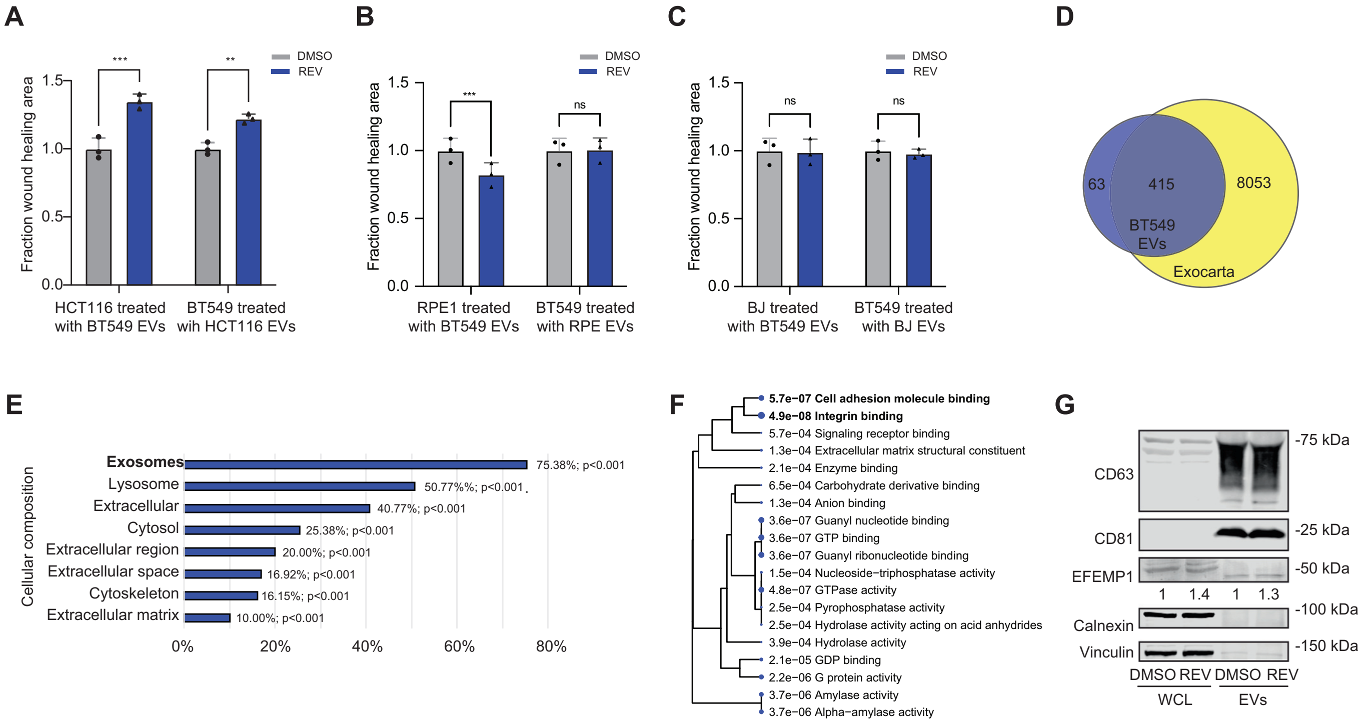


**Figure EV3. Reciprocal EV transfer assays, EV proteomics annotation, and EFEMP1 enrichment in CIN$^{HIGH}$ EVs.**

(**A**) Quantification of scratch-wound migration of HCT116 colon cancer cells treated with BT549-derived EVs generated under 250 nM REV, and the reciprocal condition in which BT549 cells were treated with HCT116-derived EVs at the same doses. Statistical significance was assessed by two-sided t-tests ($n = 3$ biological replicates); $p < 0.01$. Error bars represent the standard deviation (SD) of the mean. (**B**) Quantification of scratch-wound migration in RPE1 fibroblasts treated with BT549-derived EVs generated under 250 nM REV, and the reciprocal condition in which BT549 cells were treated with RPE1-derived EVs at the same doses. Statistical significance was assessed by two-sided t-tests ($n = 3$ biological replicates); $p < 0.01$. Error bars represent the standard deviation (SD) of the mean. (**C**) Quantification of scratch-wound migration in BJ fibroblasts treated with BT549-derived EVs generated under 250 nM REV, and the reciprocal condition in which BT549 cells were treated with BJ-derived EVs at the same doses. Statistical significance was assessed by two-sided t-tests ($n = 3$ biological replicates); $p < 0.01$. Error bars represent the standard deviation (SD) of the mean. (**D**) Venn diagram of peptides enriched in EVs isolated from BT549 cells compared to Exocarta database. (**E**) Cellular component analysis of differentially expressed proteins using Funrich 3.1.4. software. (**F**) Gene Ontology (GO) analysis on the proteins enriched in CIN$^{HIGH}$ EVs compared to CIN- EVs. (**G**) EFEMP1 levels in BT549 cell lysates and BT549 EVs treated with DMSO (CIN$^{LOW}$) or REV (CIN$^{HIGH}$) detected by Western blot. Source data are available online for this figure.

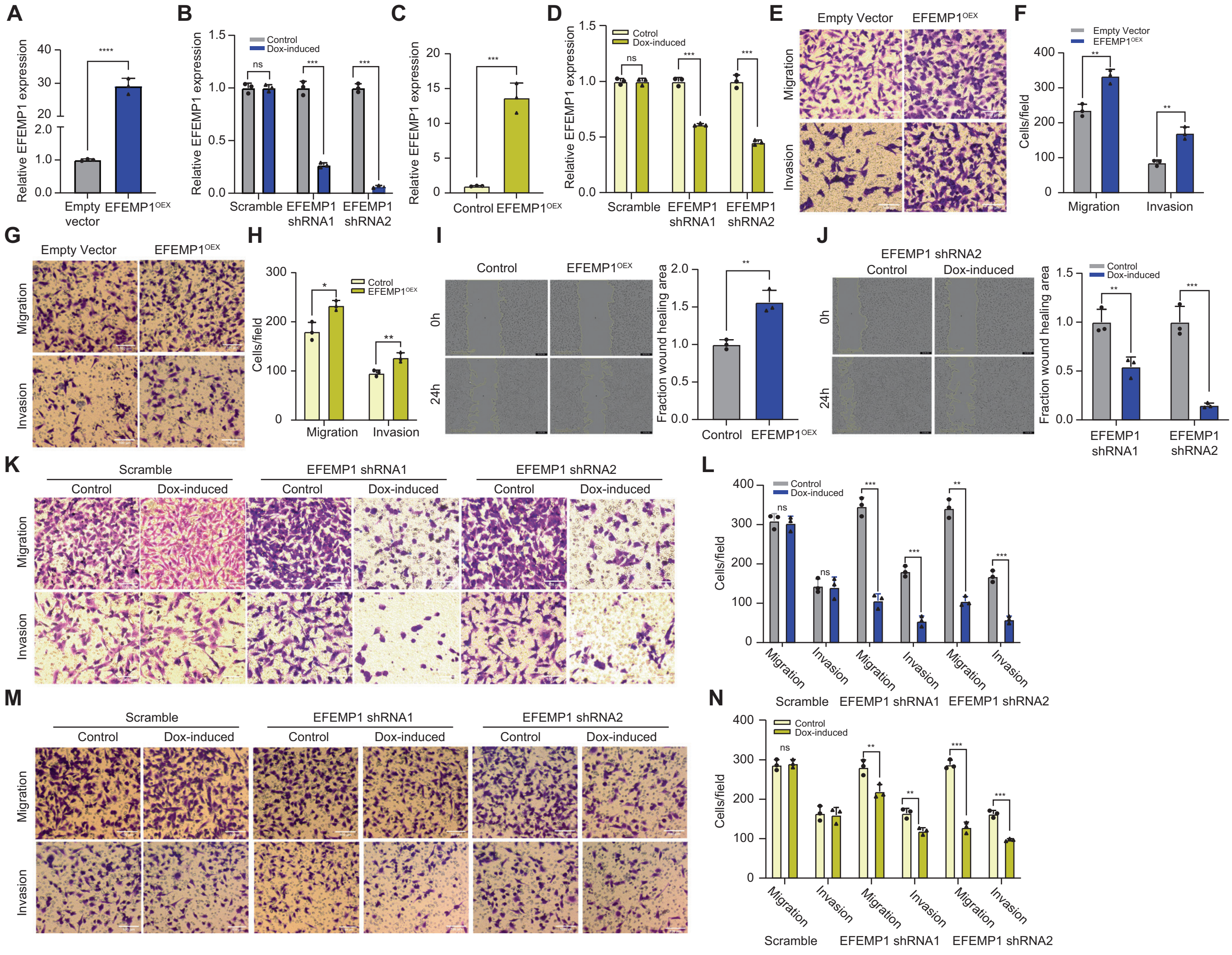


**Figure EV4. EFEMP1 enhances migration and invasion of BT549 and MDA-MB-231 cells.**

(**A**) qPCR quantification of EFEMP1 knockdown efficacy in BT549 cells. Statistical tests were done using a two-sided T-test ($n = 3$; ***, $p < 0.001$). (**B**) qPCR quantification of EFEMP1 overexpression in BT549 cells. Statistical significance was assessed by a two-sided T-test ($n = 3$; ****, $p < 0.0001$). (**C**) qPCR quantification of EFEMP1 knockdown efficiency in MDA-MB-231 cells. Statistical analysis using a two-sided T-test ($n = 3$; ***, $p < 0.001$). (**D**) qPCR quantification of EFEMP1 overexpression. Statistical significance assessed by a two-sided T-test ($n = 3$; ****, $p < 0.0001$). (**E**, **F**) Representative images (**E**) and quantification (**F**) of cell migration and invasion of BT549 cells with EFEMP1 overexpression assessed by trans-well assays. Statistical significance was determined by a two-sided T-test ($n = 3$ biological replicates), *$p < 0.05$, **$p < 0.01$, ***$p < 0.001$, ****$p < 0.0001$. Scale bar: 100 μm. (**G**, **H**) Representative images (**G**) and quantification (**H**) of cell migration and invasion of MDA-MB-231 cells with EFEMP1 overexpression assessed by trans-well assays. Statistical significance was determined by a two-sided T-test ($n = 3$ biological replicates), *$p < 0.05$, **$p < 0.01$, ***$p < 0.001$, ****$p < 0.0001$. Scale bar: 100 μm. (**I**) Representative images (left panel) and quantification (right panel) of scratch assays to quantify migration of BT549 cells with EFEMP1 knockdown. Statistical significance was determined by a two-sided T-test ($n = 3$ biological replicates), *$p < 0.05$, **$p < 0.01$, ***$p < 0.001$, ****$p < 0.0001$. (**J**) Representative images (left panel) and quantification (right panel) of scratch assays to quantify migration of BT549 cells with EFEMP1 overexpression. Statistical significance was determined by a two-sided T-test ($n = 3$ biological replicates), *$p < 0.05$, **$p < 0.01$, ***$p < 0.001$, ****$p < 0.0001$. (**K**, **L**) Representative images (**K**) and quantification (**L**) of cell migration and invasion of BT549 cells with EFEMP1 knockdown assessed by trans-well assays. Statistical significance was determined by a two-sided T-test ($n = 3$ biological replicates), *$p < 0.05$, **$p < 0.01$, ***$p < 0.001$, ****$p < 0.0001$. Scale bar: 100 μm. (**M**, **N**) Representative images (**M**) and quantification (**N**) of cell migration and invasion of MDA-MB-231 cells with EFEMP1 knockdown (two shRNAs) assessed by trans-well assays. Statistical significance was determined by a two-sided T-test ($n = 3$ biological replicates), *$p < 0.05$, **$p < 0.01$, ***$p < 0.001$, ****$p < 0.0001$. Source data are available online for this figure.


© The Author(s)

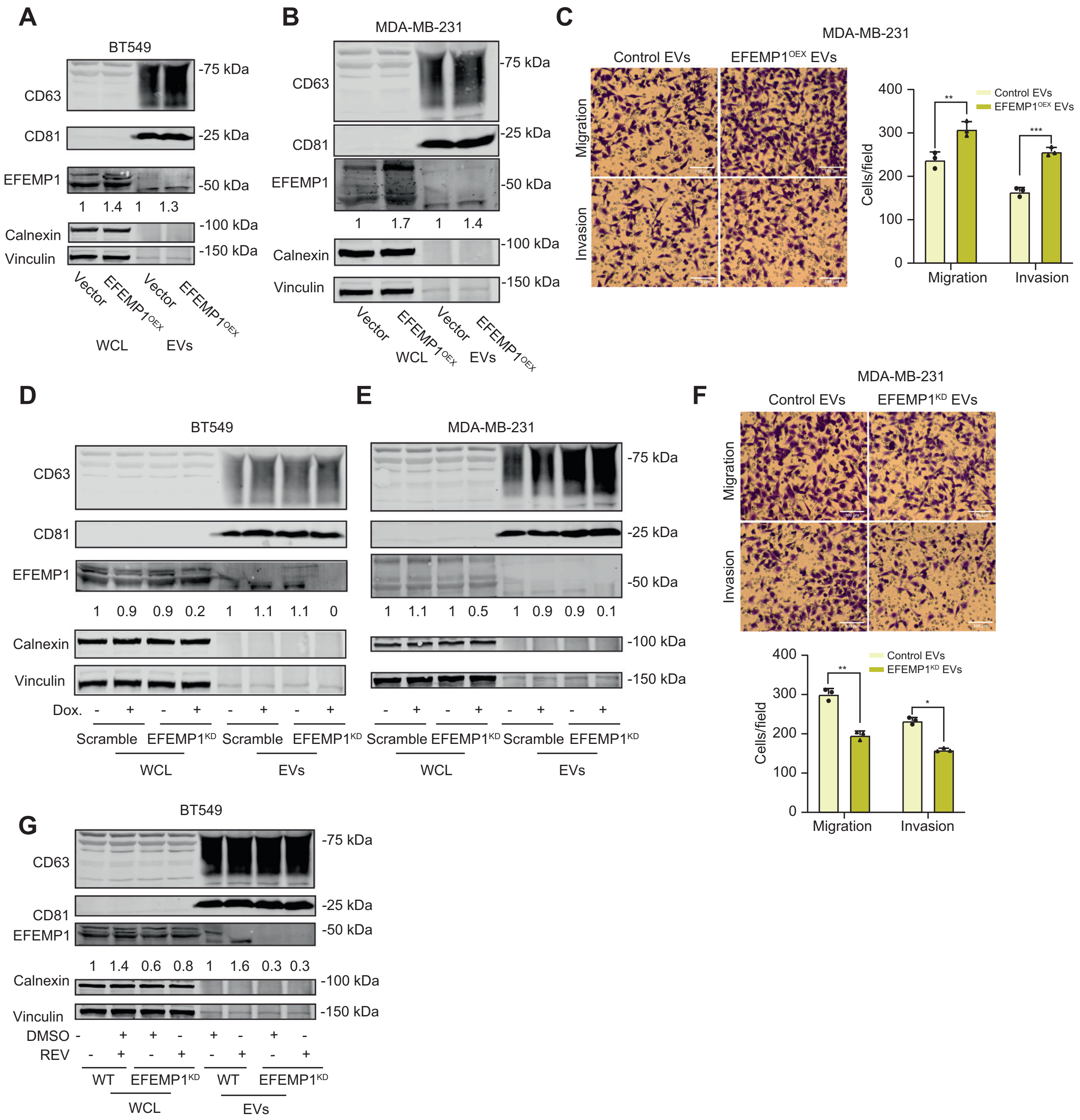


**Figure EV5. EFEMP1 and EV marker expression and EV-mediated migration/invasion in BT549 and MDA-MB-231 cells.**

(**A**) CD63, CD81, EFEMP1, Calnexin and Vinculin protein levels in whole cell lysates and EVs from BT549 and BT549 EFEMP1$^{OEX}$ cells determined by Western blot. (**B**) CD63, CD81, EFEMP1, Calnexin and Vinculin protein levels in whole cell lysates and EVs from MDA-MB-231 cells with or without EFEMP1 overexpression determined by Western blot. (**C**) Representative images (left panel) and quantification (right panel) of cell migration and invasion of MDA-MB-231 cells treated with EFEMP1$^{OEX}$ EVs assessed by trans-well assays. Statistical significance was determined by a two-sided T-test ($n = 3$ biological replicates). Scale bar: 100 µm. (**D**) CD63, CD81, EFEMP1, Calnexin and Vinculin protein levels in whole cell lysates and EVs from BT549 scramble and EFEMP1 knockdown (EFEMP1$^{KD)}$ cells determined by Western blot. (**E**) CD63, CD81, EFEMP1, Calnexin and Vinculin protein levels in whole cell lysates and EVs from MDA-MB-231 control and EFEMP1$^{KD}$ cells determined by Western blot. (**F**) Representative images (upper panel) and quantification (bottom panel) of cell migration and invasion of MDA-MB-231 cells treated with EFEMP1$^{KD}$ EVs assessed by transwell assays. Statistical significance was determined by a two-sided T-test ($n = 3$ biological replicates), $*p < 0.05$, $**p < 0.01$, $***p < 0.001$, $****p < 0.0001$. Scale bar: 100 µm. (**G**) CD63, CD81, EFEMP1, Calnexin and vinculin protein levels in whole cell lysates and EVs from DMSO- or REV-treated BT549 scramble and EFEMP1$^{KD}$ cells determined by Western blot. Source data are available online for this figure.

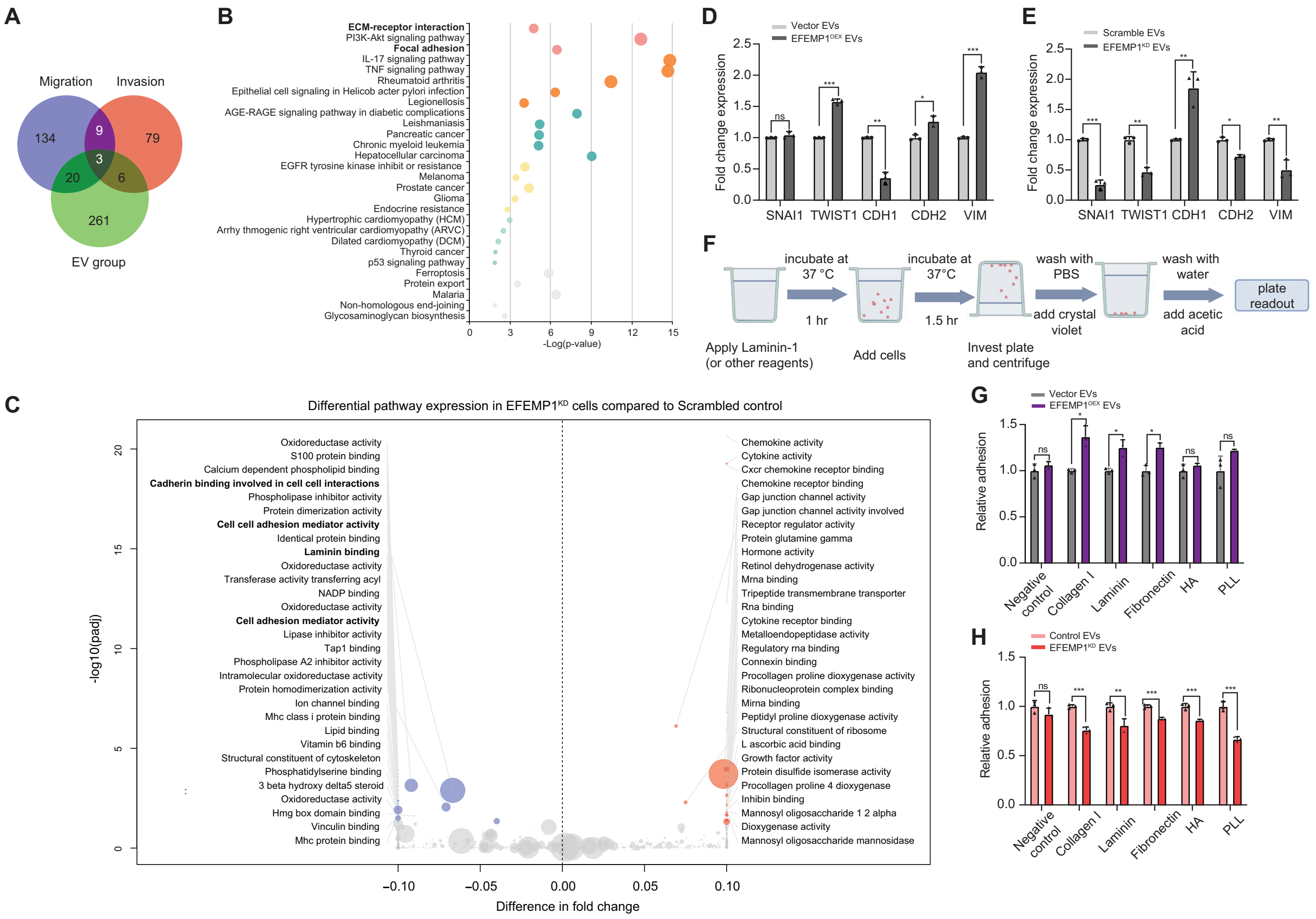


**Figure EV6. EFEMP1 modulates cell adhesion in a paracrine manner.**

(**A**) Venn diagram of RNA sequencing analysis illustrating the downregulation of 29 genes associated with migration and invasion in BT549 cells treated with EFEMP1$^{KD}$ EVs. The gene sets for migration (166 genes) and invasion (97 genes) were derived from the Cancer Single-cell State Atlas, underscoring the significant role of EFEMP1 in regulating cellular migration and invasion patterns (http://biocc.hrbmu.edu.cn/CancerSEA/goDownload). (**B**) KEGG pathway analysis of the 290 deregulated genes in EFEMP1$^{KD}$ cells. (**C**) Molecular Functions (MF) pathway analysis of the 290 deregulated genes in EFEMP1$^{KD}$ cells. (**D**) qPCR quantification of SNAI1, TWIST, CHD1, CDH2, VIM in vector EVs and EFEMP1$^{OEX}$ EVs treated BT549 cells: Statistical tests were done using a two-sided T-test ($n = 3$; ***, **$p < 0.01$, ***$p < 0.001$). (**E**) qPCR quantification of SNAI1, TWIST, CHD1, CDH2, VIM in scramble EVs and EFEMP1$^{KD}$ EVs treated BT549 cells: Statistical tests were done using a two-sided T-test ($n = 3$; ***, **$p < 0.01$, ***$p < 0.001$). (**F**) Schematic outline of the cell adhesion assay. Image created using a licenced BioRender account. (**G**) Quantification of cell adhesion of BT549 cells treated with EVs-derived from EFEMP1$^{KD}$ cells. (**H**) Quantification of cell adhesion of BT549 cells treated with EVs-derived from EFEMP1$^{OEX}$ cells. Source data are available online for this figure.

 © The Author(s)

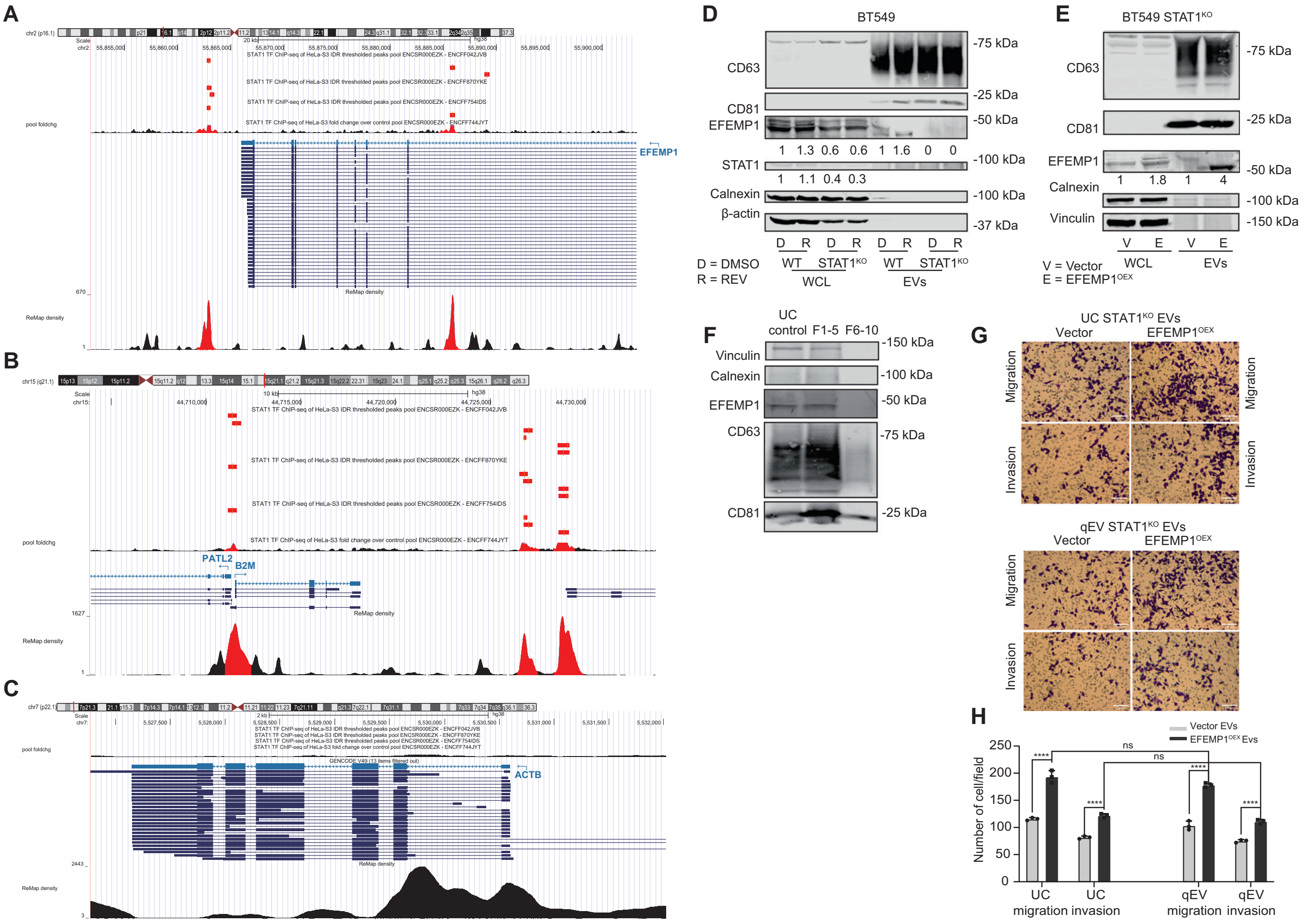


**Figure EV7. EFEMP1 and EV marker expression in BT549 WT and BT549 STAT1KO cells.**

(**A**) ChIP-seq-identified STAT1 binding motifs at the human EFEMP1 locus from public ENCODE data. (**B**) ChIP-seq-identified STAT1 binding motifs at the human B2M locus from public ENCODE data. (**C**) ChIP-seq-identified STAT1 binding motifs at the human ACTB locus from public ENCODE data. (**D**) CD63, CD81, EFEMP1, Calnexin and beta-Actin protein levels in whole cell lysates and EVs from BT549 and STAT$^{KO}$ BT549 cells treated with DMSO or REV determined by Western blot. (**E**) CD63, CD81, EFEMP1, Calnexin and Vinculin protein levels in whole cell lysates and EVs from STAT$^{KO}$ BT549 cells treated with or without EFEMP1 overexpression determined by Western blot. (**F**) Western blots of EV-protein isolates from STAT$^{KO}$ BT549 cells treated with or without EFEMP1 overexpression fractionated by SEC with fractions 1–5 and fractions 6–10 pooled. (**G**, **H**) Representative images (**G**) and quantification (**H**) of cell migration and invasion of BT549 cells co-cultured with EVs isolated from STAT1$^{KO}$ BT549 cells by ultracentrifugation (UC) or size-exclusion chromatography (qEV), treated with vector or EFEMP1$^{OEX}$, assessed by transwell assays. Statistical significance was determined by a two-way ANOVA ($n = 3$ biological replicates), *$p < 0.05$, **$p < 0.01$, ***$p < 0.001$, ****$p < 0.0001$. Scale bar: 100 µm. Source data are available online for this figure.

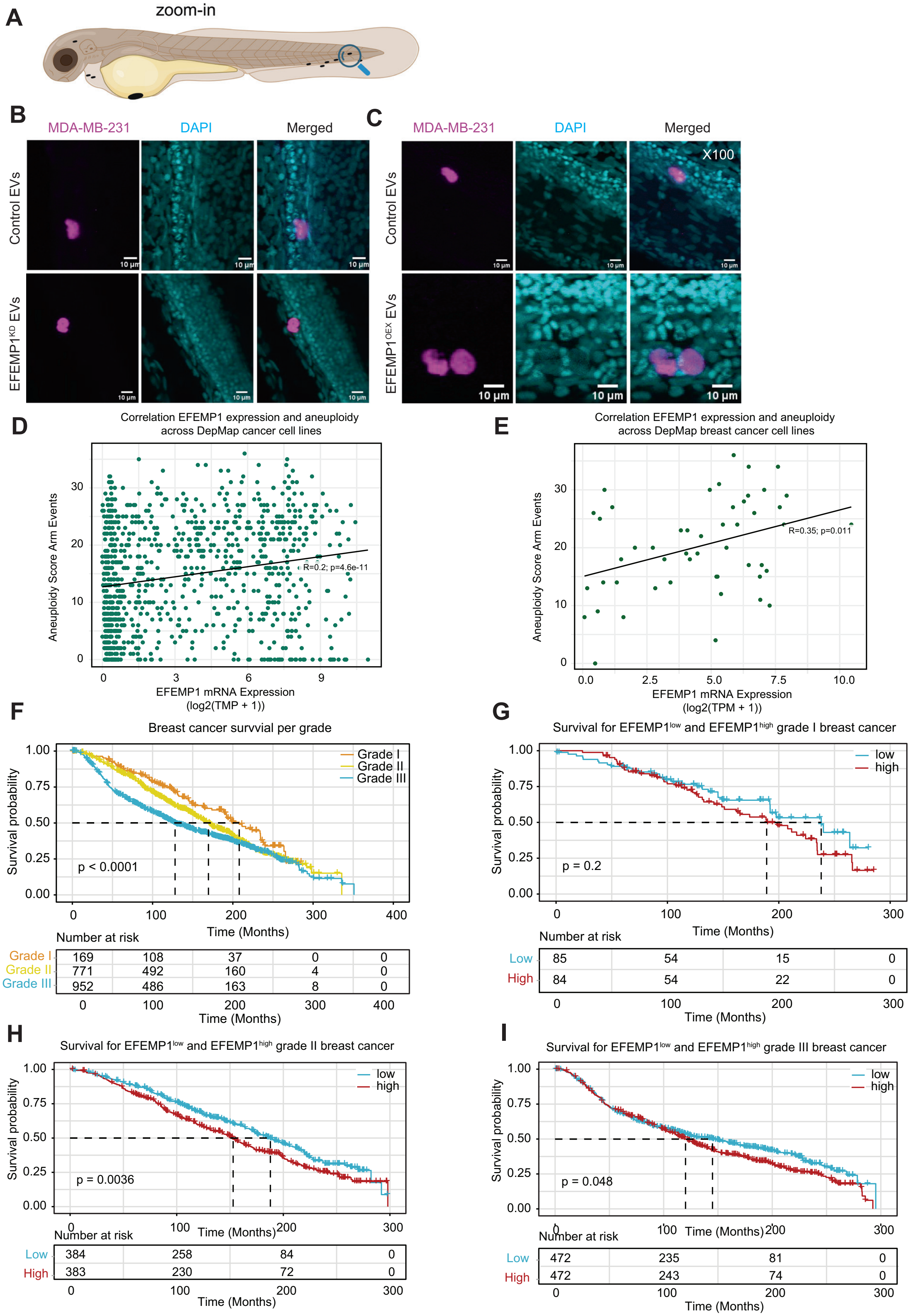
A
zoom-in
B
MDA-MB-231
DAPI
Merged
Control EVs
EFEMP1KD EVs
10 µm
C
MDA-MB-231
DAPI
Merged
X100
Control EVs
EFEMP1OEX EVs
10 µm
D
Correlation EFEMP1 expression and aneuploidy across DepMap cancer cell lines
Aneuploidy Score Arm Events
R=0.2; p=4.6e-11
EFEMP1 mRNA Expression (log2(TMP + 1))
E
Correlation EFEMP1 expression and aneuploidy across DepMap breast cancer cell lines
Aneuploidy Score Arm Events
R=0.35; p=0.011
EFEMP1 mRNA Expression (log2(TPM + 1))
F
Breast cancer survvial per grade
Survival probability
Grade I
Grade II
Grade III
p < 0.0001
Time (Months)
Number at risk
Grade I 169 108 37 0 0
Grade II 771 492 160 4 0
Grade III 952 486 163 8 0
Time (Months)
G
Survival for EFEMP1low and EFEMP1high grade I breast cancer
Survival probability
low
high
p = 0.2
Time (Months)
Number at risk
Low 85 54 15 0
High 84 54 22 0
Time (Months)
H
Survival for EFEMP1low and EFEMP1high grade II breast cancer
Survival probability
low
high
p = 0.0036
Number at risk
Time (Months)
Low 384 258 84 0
High 383 230 72 0
Time (Months)
I
Survival for EFEMP1low and EFEMP1high grade III breast cancer
Survival probability
low
high
p = 0.048
Number at risk
Time (Months)
Low 472 235 81 0
High 472 243 74 0
Time (Months)


© The Author(s)

**Figure EV8. High resolution imaging confirms MDA-MB-231 spreading to the tail of xenografted zebrafish embryos, and EFEMP1 and STAT1 expression patterns in breast cancer progression and prognosis.**

(**A**) Schematic overview of a zebrafish embryo showing the region where migrated MDA-MB-231 cells were monitored. Image created using a licenced BioRender account. (**B**, **C**) Representative single optical section images of zebrafish embryo tails with mCherry-H2B labelled MDA-MB-231 cells 1 dpt. All nuclei were labelled with DAPI Scale bar: 10 µm. (**D**, **E**) Scatter plots showing the correlation between EFEMP1 expression and aneuploidy score in DepMap included cancer cell lines (**D**) or DepMap included breast cancer cell lines (**E**). (**F**) Kaplan–Meier survival curves for TGCA-included breast cancer patients, showing overall survival for grade 3 (blue) ($n$ = 952), grade 2 (yellow) ($n$ = 771) and grade 1 (tangerine) ($n$ = 169) breast cancer. (**G**–**I**) Kaplan–Meier survival curves for TGCA-included breast cancer patients stratified for low or high EFEMP1 expression per grade (grade I, **G**; grade II, **H**, grade III, **I**). Significant differences between EFEMP1 expression groups were tested using a Log-rank Test. Source data are available online for this figure.
